# Supplementary material for: EMULSION: Transparent and flexible multiscale stochastic models in human, animal and plant epidemiology
Source: PLoS Comput Biol. 2019 Sep 13;15(9):e1007342. doi: 10.1371/journal.pcbi.1007342 (PMC6760811; doi:10.1371/journal.pcbi.1007342)
Supplement: S5 Text — Differences between original and modified models (resp. “hybrid_SIR_ages _metapop.yaml” and “hybrid_VSIR_ages_metapop.yaml” provided in S1 File) are presented side-by-side and highlighted (in bold; red: parts removed, green: parts added; yellow: parts modified; dashes: skipped identical lines). (PDF) [file pcbi.1007342.s005.pdf]

hybrid_SIR_ages_metapop.yaml

```
# general information
model_name: hybrid_SIR_ages_metapop

model_info:
  abstract: 'This model is a simple discrete-time, stochastic,
  hybrid SIR model (with individuals grouped automatically), with
  demography based on age groups, at the metapopulation scale, with
  data-based trade movements.'
  author: 'Sebastien Picault (sebastien.picault@inra.fr)'
---
  # specify the date where simulation starts (to connect with recorded data)
  origin: 'January 1, 2018'

---
      - R:
          name: 'Resistant'
          desc: 'healthy again and resistant to infection'
          fillcolor: 'midnightblue'
    # list of transitions between states
    transitions:
      - {from: S, to: I, rate: 'transmission_I * total_I / total_population'}
      - {from: I, to: R, rate: 'recovery'}
  age_group:
    desc: 'The state machine which defines the evolution of age groups.'
---
  init_prevalent_pop:
    desc: 'initial proportion of infected populations'
    value: 0.1

```

hybrid_VSIR_ages_metapop.yaml

```
# general information
model_name: hybrid_VSIR_ages_metapop

model_info:
  abstract: 'This model is a simple discrete-time, stochastic,
  hybrid SIR model (with individuals grouped automatically), with
  demography based on age groups, at the metapopulation scale, with
  data-based trade movements. Vaccination is introduced for a targeted
  group and a specific period of the year'
  author: 'Sebastien Picault (sebastien.picault@inra.fr)'
---
  # specify the date where simulation starts (to connect with recorded data)
  origin: 'January 1, 2018'
  calendars:
    # this calendar defines the vaccination period
    vaccinal:
      period: {days: 365}
      events:
        spring: {begin: 'March 21', end: 'June, 21'}

---
      - R:
          name: 'Resistant'
          desc: 'healthy again and resistant to infection'
          fillcolor: 'midnightblue'
      - V:
          name: 'Vaccinated'
          desc: 'temporarily immune to infection'
          fillcolor: 'orange'
    # list of transitions between states
    transitions:
      - {from: S, to: I, rate: 'transmission_I * total_I / total_population'}
      - {from: I, to: R, rate: 'recovery'}
      # vaccinate Juveniles in spring
      - {from: S, to: V, when: 'spring', cond: is_J, rate:
        'vaccination'}
      # vaccinal protection gradually fades out
      - {from: V, to: S, rate: 'waning'}
  age_group:
    desc: 'The state machine which defines the evolution of age groups.'
---
  init_prevalent_pop:
    desc: 'initial proportion of infected populations'
    value: 0.1
  vaccination:
    desc: 'rate at which individuals are vaccintated (/day)'
    value: 0.1
  waning:
    desc: 'rate at which vaccinal protection is lost (/day)'
    value: '1/100'

```
