# Supplementary material for: EMULSION: Transparent and flexible multiscale stochastic models in human, animal and plant epidemiology
Source: PLoS Comput Biol. 2019 Sep 13;15(9):e1007342. doi: 10.1371/journal.pcbi.1007342 (PMC6760811; doi:10.1371/journal.pcbi.1007342)
Supplement: S2 File — This zip file is a clone of EMULSION public Git repository. To install from this file rather than from PyPI, go to EMULSION documentation page: https://sourcesup.renater.fr/www/emulsion-public/pages/Install.html and follow Git-based installation instructions. (ZIP) [file pcbi.1007342.s010.zip › S2_file/models/features/img/hybrid_SIR_aggreg.html]

Emulsion Plot: hybrid\_SIR\_aggreg
