# Supplementary material for: EMULSION: Transparent and flexible multiscale stochastic models in human, animal and plant epidemiology
Source: PLoS Comput Biol. 2019 Sep 13;15(9):e1007342. doi: 10.1371/journal.pcbi.1007342 (PMC6760811; doi:10.1371/journal.pcbi.1007342)
Supplement: S2 File — This zip file is a clone of EMULSION public Git repository. To install from this file rather than from PyPI, go to EMULSION documentation page: https://sourcesup.renater.fr/www/emulsion-public/pages/Install.html and follow Git-based installation instructions. (ZIP) [file pcbi.1007342.s010.zip › S2_file/doc/_modules/emulsion/tools/calendar.html]

emulsion.tools.calendar — EMULSION (Epidemiological Multi-Level Simulation framework)

# Source code for emulsion.tools.calendar

```
""".. module:: emulsion.tools.calendar

Classes and functions for the definition of Emulsion calendars.
"""

# [HEADER]


from   sortedcontainers      import SortedDict


#  ______                    _   _
# |  ____|                  | | (_)
# | |__  __  _____ ___ _ __ | |_ _  ___  _ __  ___
# |  __| \ \/ / __/ _ \ '_ \| __| |/ _ \| '_ \/ __|
# | |____ >  < (_|  __/ |_) | |_| | (_) | | | \__ \
# |______/_/\_\___\___| .__/ \__|_|\___/|_| |_|___/
#                     | |
#                     |_|

[docs]class InvalidIntervalException(Exception):
    """Exception raised when trying to insert an inconsistent event in the
    calendar. An event is considered inconsistent if the begin date is
    posterior to the send date in a non-periodic calendar.

    """

[docs]    def __init__(self, begin, end):
        """Create the exception with the incorrect *begin* and *end* dates."""
        super().__init__()
        self.value = (begin, end)

def __str__(self):
        return 'Invalid dates interval {}'.format(self.value)

#  ______                _   _
# |  ____|              | | (_)
# | |__ _   _ _ __   ___| |_ _  ___  _ __  ___
# |  __| | | | '_ \ / __| __| |/ _ \| '_ \/ __|
# | |  | |_| | | | | (__| |_| | (_) | | | \__ \
# |_|   \__,_|_| |_|\___|\__|_|\___/|_| |_|___/

[docs]def date_in(begin, end, period=None):
    """Build a function which tests if a date belongs to the specified
    interval (from *begin* to *end*). If a *period* is specified, the
    test relies upon the periodicity, otherwise dates are considered
    absolute.

    Parameters
    ----------
    begin: ``datetime.datetime``
        the date when the interval begins
    end: ``datetime.datetime``
        the date when the interval ends
    period: ``datetime.timedelta``
        the duration of the period after which the calendar cycles

    Returns
    -------
    lambda:
        a function which maps a date (``datetime.datetime``) to a
        ``bool`` to indicate whether or not the date belongs to the
        *begin*-*end* interval.

    Raises
    ------
    InvalidIntervalexception
        if *begin* is posterior to *end* in a non-periodic calendar
    """
    if period is not None:
        begin, end = begin % period, end % period
        if begin <= end:
            def test_date(date):
                """Check that the specified date belongs to the event interval,
                considering the periodicity.

                """
                return begin <= (date % period) <= end
        else:
            def test_date(date):
                """Check that the specified date belongs to the event interval,
                considering the periodicity and the fact that the end
                date appears before the begin date in a civil year.

                """
                return (date % period) <= end or (date % period) >= begin
    else:
        if begin <= end:
            def test_date(date):
                """Check that the specified date belongs to the event interval,
                considered as absolute (no periodicity).

                """
                return begin <= date <= end
        else:
            raise InvalidIntervalException(begin, end)
    return test_date

#  ______               _    _____      _                _
# |  ____|             | |  / ____|    | |              | |
# | |____   _____ _ __ | |_| |     __ _| | ___ _ __   __| | __ _ _ __
# |  __\ \ / / _ \ '_ \| __| |    / _` | |/ _ \ '_ \ / _` |/ _` | '__|
# | |___\ V /  __/ | | | |_| |___| (_| | |  __/ | | | (_| | (_| | |
# |______\_/ \___|_| |_|\__|\_____\__,_|_|\___|_| |_|\__,_|\__,_|_|

[docs]class EventCalendar:
    """The EventCalendar class is intended to handle events and
    periods of time. Dates/times are given to the calendar in a
    human-readable format, and converted to integers (simulation
    steps).

    """

[docs]    def __init__(self, calendar_name, step_duration, origin,
                 period=None, **initial_dates):
        """Initialize the calendar using the specified information.

        Parameters
        ----------
        calendar_name: string
            the name of the calendar
        step_duration: ``datetime.timedelta``
            actual duration of one simulation step
        origin: ``datetime.datetime``
            date/time value of the beginning of the calendar
        period: ``datetime.timedelta``
            if the calendar is periodic, actual duration of the
            period, None otherwise
        initial_dates: dict
             initial dictionary of events (event name as key, dict as
             value with either begin/end dates or one single date if
             punctual event)

        """
        self.calendar_name = calendar_name
        self.step = 0
        self.step_duration = step_duration
        self.origin = origin
        self.current_date = self.origin
        self.events = SortedDict()
        self.description = SortedDict()
        self.period = None if period is None\
          else period // self.step_duration
        for name, value in initial_dates.items():
            self.add_event(name, value)


[docs]    def increment(self, steps=1):
        """Advance the current date by the specified number of
        simulation steps.

        Parameters
        ----------
        steps: int
            number of steps to 'add' to the current date
        """
        self.current_date += self.step_duration * steps


[docs]    def step_to_date(self, step):
        """Return the date when the specified step begins.

        Parameters
        ----------
        step: int
            the time step to convert to a date

        Returns
        -------
        d: ``datetime.datetime``
            the corresponding date
        """
        return self.origin + self.step_duration * step


[docs]    def date_to_step(self, date):
        """Return the step corresponding the specified date.

        Parameters
        ----------
        date: ``datetime.datetime``
            the date to convert into time steps

        Returns
        -------
        t: int
            the time step during which the specified date occurs (the
            date occurs between the returned time step *t* and time
            step *t+1*)

        """
        return (date - self.origin) // self.step_duration


[docs]    def add_event(self, name, begin_end):
        """Add the specified event to the calendar. An event is characterized
        by its *name* and a *begin_end* tuple indicating the begin and
        end dates.

        Parameters
        ----------
        name: str
            the name of the event
        begin_end: tuple
           a tuple composed of the begin date and the end date
           (possibly identical for handling punctual events)

        """
        begin_date, end_date = begin_end
        begin = self.date_to_step(begin_date)
        end = self.date_to_step(end_date)
        if end < begin and self.period is None:
            raise InvalidIntervalException(begin, end)
        self.description[name] = begin_end
        self.events[name] = date_in(begin, end, self.period)


[docs]    def get_events(self):
        """Return the list of events contained in the current calendar.

        Returns
        -------
        l: list
            the list of all event names
        """
        return list(self.description.keys())

def __getitem__(self, name):
        return self.events[name]
```

# EMULSION

Epidemiological Multi-Level Simulation Framework

### Navigation

- 1. Installation
- 2. Getting started with EMULSION
- 3. Modelling principles
- 4. Modelling language (basics)
- 5. Modelling language (advanced)
- 6. Feature examples
- 7. Information
- 8. License
- 9. High-level functions for model designers
- 10. emulsion package

### Related Topics

- Documentation overview
  - Module code

### Quick search

©2016, INRA and Univ. Lille.
|
Powered by Sphinx 1.8.5
& Alabaster 0.7.10
