# Supplementary material for: EMULSION: Transparent and flexible multiscale stochastic models in human, animal and plant epidemiology
Source: PLoS Comput Biol. 2019 Sep 13;15(9):e1007342. doi: 10.1371/journal.pcbi.1007342 (PMC6760811; doi:10.1371/journal.pcbi.1007342)
Supplement: S2 File — This zip file is a clone of EMULSION public Git repository. To install from this file rather than from PyPI, go to EMULSION documentation page: https://sourcesup.renater.fr/www/emulsion-public/pages/Install.html and follow Git-based installation instructions. (ZIP) [file pcbi.1007342.s010.zip › S2_file/doc/_modules/emulsion/tools/graph.html]

emulsion.tools.graph — EMULSION (Epidemiological Multi-Level Simulation framework)

# Source code for emulsion.tools.graph

```
"""A Python implementation of the EMuLSion framework (Epidemiologic
MUlti-Level SImulatiONs).

Tools aimed at handling graphs for the state machines
"""

# [HEADER]


from   collections               import OrderedDict

from   emulsion.tools.state      import EmulsionEnum


EdgeTypes = EmulsionEnum('EdgeTypes', ['TRANSITION', 'PRODUCTION'], module=__name__)
EdgeTypes.linestyle = 'solid'
EdgeTypes.PRODUCTION.linestyle = 'dashed'

[docs]class MultiDiGraph:
    """An oriented multigraph (two nodes can be linked by several
    edges). Replacement for MultiDiGraph in networkx with a
    reproducible order in accessing edges and nodes.

    """

[docs]    def __init__(self, **attributes):
        """Create an instance of MultiDiGraph. If keywords arguments
        are specified, they are considered attributes of the whole
        graph.

        """
        self.graph = dict(**attributes)
        self.node = OrderedDict()
        self.edge = OrderedDict()
        self._edge_counter = OrderedDict()


[docs]    def add_node(self, node_id, **attributes):
        """Add the specified node to the graph. Node attributes can be
        specified. If the node is already present, updated the
        attributes.

        """
        if node_id not in self.node:
            self.node[node_id] = dict()
        self.node[node_id].update(attributes)


[docs]    def add_edge(self, from_id, to_id, type_id=EdgeTypes.TRANSITION,
                 key=None, **attributes):
        """Add the specified edge to the graph. If nodes are not
        already created, they are automatically added. Edge attributes
        can be specified. Since this graph allows multiple edges
        between pairs of nodes, two calls to this method lead to two
        distinct edges (even if the attributes are the same), unless a
        key is specified. The key is used to identify edges between a
        given pair of nodes. By default, keys are consecutive
        integers.

        """
        self.add_node(from_id)
        self.add_node(to_id)
        if from_id not in self.edge:
            self.edge[from_id] = OrderedDict()
            self._edge_counter[from_id] = OrderedDict()
        if to_id not in self.edge[from_id]:
            self.edge[from_id][to_id] = OrderedDict()
            self._edge_counter[from_id][to_id] = 0
        if key is None:
            edge_key = self._edge_counter[from_id][to_id]
            self._edge_counter[from_id][to_id] += 1
        else:
            edge_key = key
        attributes['type_id'] = type_id
        self.edge[from_id][to_id][edge_key] = dict(**attributes)


[docs]    def edges(self):
        """Return a list of edge tuples."""
        return [(from_id, to_id)
                for from_id in self.edge
                for to_id in self.edge[from_id]]


[docs]    def edges_from(self, from_id, type_id=EdgeTypes.TRANSITION):
        """Return a list of tuples (to_id, attributes) corresponding
        to all edges going out of the `from_id` node.

        """
        if from_id not in self.edge:
            return []
        return [(state, attribs)
                for state in self.edge[from_id]
                for attribs in self.edge[from_id][state].values()
                if (type_id is None) or (attribs['type_id'] == type_id)]
```

# EMULSION

Epidemiological Multi-Level Simulation Framework

### Navigation

- 1. Installation
- 2. Getting started with EMULSION
- 3. Modelling principles
- 4. Modelling language (basics)
- 5. Modelling language (advanced)
- 6. Feature examples
- 7. Information
- 8. License
- 9. High-level functions for model designers
- 10. emulsion package

### Related Topics

- Documentation overview
  - Module code

### Quick search

©2016, INRA and Univ. Lille.
|
Powered by Sphinx 1.8.5
& Alabaster 0.7.10
