# Supplementary material for: EMULSION: Transparent and flexible multiscale stochastic models in human, animal and plant epidemiology
Source: PLoS Comput Biol. 2019 Sep 13;15(9):e1007342. doi: 10.1371/journal.pcbi.1007342 (PMC6760811; doi:10.1371/journal.pcbi.1007342)
Supplement: S2 File — This zip file is a clone of EMULSION public Git repository. To install from this file rather than from PyPI, go to EMULSION documentation page: https://sourcesup.renater.fr/www/emulsion-public/pages/Install.html and follow Git-based installation instructions. (ZIP) [file pcbi.1007342.s010.zip › S2_file/doc/_modules/emulsion/tools/parallel.html]

emulsion.tools.parallel — EMULSION (Epidemiological Multi-Level Simulation framework)

# Source code for emulsion.tools.parallel

```
"""A Python implementation of the EMuLSion framework (Epidemiologic
MUlti-Level SImulatiONs).

Tools for parallel computing.
"""

# [HEADER]


import multiprocessing           as     mp

from   emulsion.tools.simulation import MultiSimulation, SensitivitySimulation

[docs]def job_dist(total_task, workers):
    """ Return a distribution of each worker need to do.

    """
    nb = total_task // workers
    rest = total_task % workers
    return [nb+1 if i <= rest-1 else nb for i in range(workers)]


[docs]def job(target_simulation_class, proc, **others):
    """ Simple job for a simple processes

    """
    simu = target_simulation_class(proc=proc, **others)
    simu.run()
    simu.write_dot()

# simu.counts_to_csv()

[docs]def parallel_multi(target_simulation_class=MultiSimulation, nb_simu=None,
                   nb_proc=1, **others):
    """ Parallel loop for distributing tasks in different processes

    """
    list_proc = []
    list_nb_simu = job_dist(nb_simu, nb_proc)

    for i in range(nb_proc):
        others['nb_simu'] = list_nb_simu[i]
        others['start_id'] = sum(list_nb_simu[:i])
        proc = mp.Process(target=job, args=(target_simulation_class, i),
                          kwargs=others)
        list_proc.append(proc)
        proc.start()

    for proc in list_proc:
        proc.join()


[docs]def parallel_sensi(target_simulation_class=SensitivitySimulation, nb_proc=1,
                   **others):
    """ Parallel loop for distributing sensitivity tasks in different processes

    """
    list_proc = []
    list_nb_simu = job_dist(len(others['df']), nb_proc)

    for i in range(nb_proc):
        others['nb_multi'] = list_nb_simu[i]
        others['start_id'] = sum(list_nb_simu[:i])
        proc = mp.Process(target=job, args=(target_simulation_class, i),
                          kwargs=others)
        list_proc.append(proc)
        proc.start()

    for proc in list_proc:
        proc.join()
```

# EMULSION

Epidemiological Multi-Level Simulation Framework

### Navigation

- 1. Installation
- 2. Getting started with EMULSION
- 3. Modelling principles
- 4. Modelling language (basics)
- 5. Modelling language (advanced)
- 6. Feature examples
- 7. Information
- 8. License
- 9. High-level functions for model designers
- 10. emulsion package

### Related Topics

- Documentation overview
  - Module code

### Quick search

©2016, INRA and Univ. Lille.
|
Powered by Sphinx 1.8.5
& Alabaster 0.7.10
