# Supplementary material for: EMULSION: Transparent and flexible multiscale stochastic models in human, animal and plant epidemiology
Source: PLoS Comput Biol. 2019 Sep 13;15(9):e1007342. doi: 10.1371/journal.pcbi.1007342 (PMC6760811; doi:10.1371/journal.pcbi.1007342)
Supplement: S2 File — This zip file is a clone of EMULSION public Git repository. To install from this file rather than from PyPI, go to EMULSION documentation page: https://sourcesup.renater.fr/www/emulsion-public/pages/Install.html and follow Git-based installation instructions. (ZIP) [file pcbi.1007342.s010.zip › S2_file/doc/_modules/emulsion/tools/view.html]

emulsion.tools.view — EMULSION (Epidemiological Multi-Level Simulation framework)

# Source code for emulsion.tools.view

```
"""A Python implementation of the EMuLSion framework (Epidemiologic
MUlti-Level SImulatiONs).

Tools for data / map visualization.
"""

# [HEADER]

import numpy                as np
import matplotlib.pyplot    as plt
import matplotlib.animation as animation

[docs]def show_img(value, cmap='hot', save=None, colbar=True, **kwargs):
    """Display the specified value as an image. A special color map
    name can be specified, as well as the presence of a colorbar and
    additional keyword arguments passed to ``imshow``. If a filename is
    provided in the ``save`` parameter, the image is stored in that
    file.

    """
    plt.imshow(value, cmap=plt.get_cmap(cmap), interpolation='none', **kwargs)
    if colbar:
        plt.colorbar()
    if save:
        plt.savefig(save)
    plt.show()


[docs]def show_contour(value, cmap='hot', save=None, colbar=False, **kwargs):
    """Display the specified value as a contour map with values. A
    special color map name can be specified, as well as the presence
    of a colorbar and additional keyword arguments passed to
    ``contourf``. If a filename is provided in the ``save`` parameter, the
    image is stored in that file.

    """
    plt.contourf(value, cmap=cmap, **kwargs)
    cont = plt.contour(value, colors='black', alpha=0.75)
    plt.clabel(cont, fmt='%2.1f', colors='black', fontsize=6)
    plt.axes().set_aspect('equal')
    if colbar:
        plt.colorbar()
    if save:
        plt.savefig(save)
    plt.show()


[docs]def show_histo(value, xlabel, facecol='green', save=None):
    """Display the distribution of the specified ``value`` array using
    ``xlabel`` for the legend. A specific face color can be used. If a
    filename is provided in the ``save`` parameter, the image is
    stored in that file.

    """
    plt.hist(np.ravel(value), 50, facecolor=facecol, alpha=0.75)
    plt.xlabel(xlabel)
    plt.ylabel('Nb cells')
    plt.title('Histogram of {}'.format(xlabel))
    plt.grid(True)
    if save:
        plt.savefig(save)
    plt.show()


[docs]def build_animation(values, title, unit=None, filename=None,
                    cmap='coolwarm', framerate=10, resolution=100,
                    writer='imagemagick', **kwargs):
    """Create an animation based on the ``values`` list, with the
    specified title. A special color map name can be specified, as
    well as the presence of a colorbar and additional keyword
    arguments passed to ``imshow``. If a specified unit is given, each
    frame is marked with its number and the corresponding unit. If a
    filename is provided in the ``save`` parameter, the animation is
    stored in that file.

    """
    ims = []
    fig = plt.figure(title)
    ax = fig.add_subplot(111)

    for img_num, value in enumerate(values):
        frame = plt.imshow(value, cmap=plt.get_cmap(cmap),
                           animated=True, **kwargs)
        if unit:
            text = ax.annotate("{} {:03}".format(unit, img_num + 1), (1, 1))
            ims.append([frame, text])
        else:
            ims.append([frame])

    ani = animation.ArtistAnimation(fig, ims, interval=350, blit=True,
                                    repeat_delay=350)
    if filename:
        ani.save(filename, writer=writer, fps=framerate, dpi=resolution)

    plt.colorbar()
    plt.show()
```

# EMULSION

Epidemiological Multi-Level Simulation Framework

### Navigation

- 1. Installation
- 2. Getting started with EMULSION
- 3. Modelling principles
- 4. Modelling language (basics)
- 5. Modelling language (advanced)
- 6. Feature examples
- 7. Information
- 8. License
- 9. High-level functions for model designers
- 10. emulsion package

### Related Topics

- Documentation overview
  - Module code

### Quick search

©2016, INRA and Univ. Lille.
|
Powered by Sphinx 1.8.5
& Alabaster 0.7.10
