# Supplementary material for: EMULSION: Transparent and flexible multiscale stochastic models in human, animal and plant epidemiology
Source: PLoS Comput Biol. 2019 Sep 13;15(9):e1007342. doi: 10.1371/journal.pcbi.1007342 (PMC6760811; doi:10.1371/journal.pcbi.1007342)
Supplement: S2 File — This zip file is a clone of EMULSION public Git repository. To install from this file rather than from PyPI, go to EMULSION documentation page: https://sourcesup.renater.fr/www/emulsion-public/pages/Install.html and follow Git-based installation instructions. (ZIP) [file pcbi.1007342.s010.zip › S2_file/doc/_modules/emulsion/tools/misc.html]

emulsion.tools.misc — EMULSION (Epidemiological Multi-Level Simulation framework)

# Source code for emulsion.tools.misc

```
""".. module:: emulsion.tools.misc

Collection of various useful functions used in EMULSION framework,
especially regarding introspection.

"""

# [HEADER]


from   functools                 import partial
from   importlib                 import import_module
from   typing                    import List, Iterable, Tuple, Union, Any

import yaml

import numpy                     as np

from   sortedcontainers          import SortedSet


AGENTS = 1
"""Constant value for the index where list of agents are stored in
'populations' tuples.

"""

POPULATION = 1
"""Constant value for the index where population amounts are stored in
'populations' tuples.

"""

## TODO split module according to functions usage

[docs]def load_class(module=None, class_name: str = None, options: dict = {}):
    """Dynamically load the class with the specified *class_name* from the
    given *module*.

    Parameters
    ----------
    module:
        a Python module, where the class is expected to be located.
    class_name: str
        the name of the class to load
    options:
        some options

    Returns
    -------
    tuple:
        a tuple composed of the class (type) and the options.

    Todo
    ----
    - clarify the role of *options*
    """
    mod = import_module(module)
    return getattr(mod, class_name), options


[docs]def load_module(module_name: str):
    """Dynamically load the module with the specified *module_name* and
    return it.

    Parameters
    ----------
    module_name: str
        the name of a valid Python module (accessible in the
        ``PYTHONPATH`` environment variable).

    Returns
    -------
    ref:
        A reference to the Python module.

    """
    return import_module(module_name)


[docs]def rates_to_probabilities(total_rate: float, rate_values: List[float],
                           delta_t: float = 1) -> List[float]:
    """Transform the specified list of *rate_values*, interpreted as
    outgoing rates, into probabilities, according to the specified
    time step (*delta_t*) and normalized by *total_rate*.

    For exit rates :math:`\\rho_i` (one of the *rate_values*), the
    probability to stay in current state is given by:

    .. math::
        p_0 = e^{-\\delta t.\\sum_i \\rho_i}

    Thus, each rate :math:`\\rho_i` corresponds to a probability

    .. math::
        p_i = \\frac{\\rho_i}{\\sum_i \\rho_i} (1 - p_0)

    Parameters
    ----------
    total_rate: float
        the total exit rate, used for normalization purposes. If
        *rate_values* represent all possible exit rates, *total_rate*
        is their sum.
    rate_values: list
        the list of rates to transform
    delta_t: float
        the value of the time step (expressed in time units)

    Returns
    -------
    list:
        the list of probabilities corresponding to the *rate_values*.

    See Also
    --------
        `emulsion.tools.misc.probabilities_to_rates`_

    """
    # compute the exit probability
    base_proba = 1 - np.exp(- total_rate * delta_t)
    # normalize values proportionnally to the rate
    proba_values = [base_proba * rate / total_rate
                    for rate in rate_values]
    # add the probability to stay in the current state
    proba_values.append(1 - base_proba)
    return proba_values

# This function is useful to modellers for the following purposes:
rates_to_probabilities.__USER_FUNCTION__ = ['Rates / probabilities']

[docs]def aggregate_probability(probability: float, delta_t: float) -> float:
    """Transform the specified *probability* value, intended to represent
    a probability for events tested each time unit, into the
    probability for the specified time step (*delta_t*).

    Parameters
    ----------
    probability: float
        the probability value of an event (during 1 time unit)
    delta_t: float
        the value of the time step (expressed in time units)

    Returns
    -------
    float:
        the probability of the event during *delta_t* time units.
    """
    return 1 - (1 - probability)**delta_t

# This function is useful to modellers for the following purposes:
aggregate_probability.__USER_FUNCTION__ = ['Rates / probabilities']

[docs]def aggregate_probabilities(probability_values: Iterable[float],
                            delta_t: float) -> Iterable[float]:
    """From the specified *probability_values*, intended to represent
    probabilities of events duting one time unit, compute the
    probabilities for the specified time step (*delta_t*).

    Parameters
    ----------
    probability_values: list
        a list of probability values for several events (during 1 time unit)
    delta_t: float
        the value of the time step (expressed in time units)

    Returns
    -------
    list:
        the probabilities of the same events during *delta_t* time units.
    """
    return probability_values if delta_t == 1\
        else tuple(aggregate_probability(p, delta_t)
                   for p in probability_values)

# This function is useful to modellers for the following purposes:
aggregate_probabilities.__USER_FUNCTION__ = ['Rates / probabilities']

[docs]def probabilities_to_rates(probability_values: Iterable[float]) -> List[float]:
    """Transform a list of probabilities into a list of rates. The
    last value is expected to represent the probability of staying in
    the current state.

    Parameters
    ----------
    probability_values: list
        a list of probabilities, the last one representing the
        probability to stay in the current state

    Returns
    -------
    list:
        a list of rates corresponding to those probabilities.

    See Also
    --------
        `emulsion.tools.misc.rates_to_probabilities`_

    """
    if probability_values[-1] == 1:
        return [0] * (len(probability_values) - 1)
    sum_of_rates = - np.log(probability_values[-1])
    proba_change = 1 - probability_values[-1]
    values = [v * sum_of_rates / proba_change for v in probability_values]
    return values[:-1]

# This function is useful to modellers for the following purposes:
probabilities_to_rates.__USER_FUNCTION__ = ['Rates / probabilities']


# internal usage (TODO: make private to relevant module)

[docs]def rewrite_keys(name, position, change_list):
    prefix = name[:position]
    suffix = name[position+1:]
    return [(prefix + (key,) + suffix, value)
            for key, value in change_list]


[docs]def count_population(agents_or_pop: Tuple) -> Union[int, float]:
    """Return the amount of atoms represented in *agents_or_pop*.

    Parameters
    ----------
    agents_or_pop: tuple
      either ('population', qty) or ('agents', list of agents)

    Returns
    -------
    int or float:
        the amount corresponding to the population: generally, an int
        value, but deterministic models produce float values.

    """
    return agents_or_pop[POPULATION]\
      if 'population' in agents_or_pop\
      else len(agents_or_pop[AGENTS])


[docs]def select_random(origin: Iterable, quantity: int,
                  exclude: SortedSet = SortedSet()) -> List:
    """Return a random selection of *quantity* agents from the *origin*
    group, avoiding those explicitly in the *exclude* set. If the
    *origin* population proves too small, all available agents are
    taken, irrespective to the *quantity*.

    Parameters
    ----------
    origin: iterable
        the population where agents must be selected
    quantity: int
        the number of agents to select in the population
    exclude: set
        agents which are not available for the selection

    Returns
    -------
    list:
        a list of randomly selected agents according to the above constraints.
    """
    content = [unit for unit in origin if unit not in exclude]
    size = len(content)
    np.random.shuffle(content)
    return content[:min(quantity, size)]

# This function is useful to modellers for the following purposes:
select_random.__USER_FUNCTION__ = ['Selecting agents']

[docs]def read_from_file(filename: str):
    """Read the specified YAML *filename* and return the corresponding
    Python document.

    Parameters
    ----------
    filename: str
        the name of the YAML file to load

    Returns
    -------
    object:
        a Python object built by parsing of the YAML file, i.e. either
        a dict, list, or even str/int/etc... (Most YAML document will
        produce dictionaries.)

    """
    with open(filename, 'r') as fil:
        description = yaml.safe_load(fil)
    return description


[docs]def retrieve_value(value_or_function, agent):
    """Return a value either directly given by parameter
    *value_or_function* if it is a true value, or computed from this
    parameter seen as a function, with the specified *agent* as argument.

    Parameters
    ----------
    value_or_function:
        either a callable (function that applies to an agent to
        retrieve an individual value), or the value itself
    agent:
        the agent to use as parameter of the callable if necessary

    Returns
    -------
    value:
        the expected value (agent-based or not).

    """

    return value_or_function(agent)\
        if callable(value_or_function)\
        else value_or_function


[docs]def moving_average(values, window_size, mode='same'):
    """Compute a moving average of the specified *values* with respect to
    the *window_size* on which the average is calculated. The return
    moving average has the same size as the original values. To avoid
    boundary effects, use ``mode='valid'``, which produce a result of
    size ``len(values) - window_size + 1``.

    Parameters
    ----------
    values: array-like
        contains the values for moving average computation
    window_size: int
        width of the moving average window
    mode: str
        a parameter for `numpy.convolve`

    Returns
    -------
    nd_array:
        a numpy ``nd_array`` containing the values of the moving average.

    """
    window = np.ones(int(window_size)) / float(window_size)
    return np.convolve(values, window, mode)

# This function is useful to modellers for the following purposes:
moving_average.__USER_FUNCTION__ = ['Computations']

[docs]def add_new_property(agent, property_name, getter_function):
    """Add a new property to an agent.

    Actually, the property is added to the class of the agent, as
    Python properties are descriptors. Yet, the dynamic attribution of
    properties must me done through instances rather than classes,
    since agents must add the name of the property to their
    ``_mbr_cache`` attribute.

    Parameters
    ----------
    agent: AbstractAgent
        the agent to which the property must be added
    property_name: str
        the name of the property
    getter_function: lambda
        the function upon which the property is built

    """
    setattr(agent.__class__, property_name, property(getter_function))
    agent._mbr_cache.add(property_name)

# print('Agent {} adding property {} to class {}'.format(agent, property_name, agent.__class__))

[docs]def create_population_getter(process_name: str,
                             group_name: Union[str, Tuple]):
    """Build a getter for property of the form ``total_X_Y`` where ``X``
    and ``Y`` are states of two different states machines used in a
    grouping.

    The functions can handle groupings with an arbitrary number of states.

    Parameters
    ----------
    process_name : str
        the name of the process associated with the grouping for which
        the population getter is created
    group_name : str or tuple
        either a string representing the state name (if only one), or
        a tuple of several state names

    Returns
    -------
    callable :
        A function which can be applied to an `AbstractProcessManager`
        agent which returns the population size for the specified
        group.

    """
    if not isinstance(group_name, tuple):
        group_name = (group_name,)
    # print('adding automatic property for', process_name, group_name)
    return lambda self: self.get_group_population(process_name, group_name)


[docs]def create_counter_getter(machine_name, state_name):
    return lambda self: self.counters[machine_name][state_name]


[docs]def create_state_tester(state_name):
    return lambda self: self.is_in_state(state_name)


[docs]def create_duration_getter(machine_name):
    # print('adding automatic property for', process_name, group_name)
    return lambda self: self.duration_in_current_state(machine_name)


[docs]def add_all_test_properties(agent):
    for machine in agent.model.state_machines.values():
        add_new_property(agent, 'duration_in_{}'.format(machine.machine_name),
                         create_duration_getter(machine.machine_name))
        for state in machine.states:
            add_new_property(agent, 'is_{}'.format(state.name),
                             create_state_tester(state.name))


[docs]def find_operator(operator: str):
    """Return an aggregation function named *operator*.

    Search starts with emulsion functions (module
    `emulsion.tools.functions`), which includes Python built-ins, then
    in `numpy`.

    A special shortcut is provided for percentiles: ``percentileXX``
    is interpreted as the partial function
    ``numpy.percentile(q=int(XX))``.

    Parameters
    ----------
    operator: str
        the name of the aggregation operator

    Returns
    -------
    lambda:
        a function that takes a list (or array-like) as input and
        returns the application of the *operator* to the values.

    """
    op = None
    current_module = import_module('emulsion.tools.functions')
    # search in emulsion functions module first
    if hasattr(current_module, operator):
        op = getattr(current_module, operator)
    elif operator.startswith('percentile'):
        # special case of 'percentileXX' where XX is a number (0 to
        # 100) and automatically extracted
        op = partial(np.percentile, q=int(operator[10:]))
    else:
        # otherwise search in numpy
        op = getattr(np, operator)
    return op


[docs]def create_aggregator(sourcevar: str, operator: str):
    """Create an aggregator function to be used as property getter. This
    aggregator function has to collect all values of *sourcevar* for
    agents contained in a given host, and reduce them to one avalue
    using the specified *operator*.

    Parameters
    ----------
    sourcevar: str
        the name of the variable to collect in the sublevel
    operator: str
        the name of the operator to apply to the collected values

    Returns
    -------
    lambda:
        A function which can be applied to a `MultiProcessManager`
        agent (i.e. with explicit sublevel agents) which returns the
        aggregated values for the whole population.

    """
    op = find_operator(operator)
    return lambda self: op([sublevel.get_information(sourcevar)
                            for sublevel in self['MASTER']])


[docs]def create_group_aggregator(sourcevar: str, operator: str, process_name: str,
                            group_name: Union[str, Tuple]):
    """Build a getter for property of the form ``newvar_X_Y`` where ``X``
    and ``Y`` are states of two different states machines used in a
    grouping, ``newvar`` is an aggregate variable based on collecting
    all values of *sourcevar* for the specific *group_name* and
    aggregating the values using *operator*. The functions can handle
    groupings with an arbitrary number of states.

    Parameters
    ----------
    sourcevar: str
        the name of the variable to collect in the sublevel
    operator: str
        the name of the operator to apply to the collected values
    process_name: str
        the name of the process associated with the grouping for which
        the population getter is created
    group_name: str or tuple
        either a string representing the state name (if only one), or
        a tuple of several state names

    Returns
    -------
    lambda:
        A function which can be applied to an `MultiProcessManager`
        agent (i.e. with explicit sublevel agents) which returns the
        aggregated value for the specified group.

    """
    op = find_operator(operator)
    if not isinstance(group_name, tuple):
        group_name = (group_name,)
    # print('adding automatic property for', process_name, group_name)
    def aggregate_group(self):
        try:
            return op([sublevel.get_information(sourcevar)
                       for sublevel in self.get_group_atoms(process_name, group_name)])
        except ValueError:
            return np.nan
    return aggregate_group


[docs]def create_atoms_aggregator(sourcevar: str, operator: str,
                            machine_name: str, state_name: str):
    """Build a getter for property of the form ``newvar_X`` where ``X`` if
    the value of *state_name* (a state of *state_machine*), ``newvar``
    is an aggregate variable based on collecting all values of
    *sourcevar* for the specific *group_name* and aggregating the
    values using *operator*. This function is intended to work on
    `IBMProcessManager` agents, which do not benefit from groupings.

    Parameters
    ----------
    sourcevar: str
        the name of the variable to collect in the sublevel
    operator: str
        the name of the operator to apply to the collected values
    machine_name: str
        the name of the state machine for which the getter is created
    state_name: str
        the state name

    Returns
    -------
    lambda:
        A function which can be applied to an `IBMProcessManager`
        agent (i.e. with explicit but ungrouped sublevel agents) which
        returns the aggregated value for the specified group.

    """
    op = find_operator(operator)
    def aggregate_atoms(self):
        try:
            return op([sublevel.get_information(sourcevar)
                            for sublevel in self.select_atoms(machine_name, state=state_name)])
        except ValueError:
            return np.nan
    return aggregate_atoms


[docs]def serial(start=0, end=None, model=None):
    """A very simple serial number generator."""
    value = start
    while True:
        yield value
        value += 1
        if end is not None:
            if value >= model.get_value(end):
                value = start


[docs]def create_new_serial(end=None, model=None):
    """Create the serial number generator associated to the specified
    variable.

    """
    generator = serial(start=0, end=end, model=model)
    return lambda self: next(generator)


[docs]def create_weighted_random(machine_name, weights, model=None):
    """Create a random choice function which returns a random state from
    the given state machine (among non-autoremove states), according
    to the weights. Weights are interpreted either directly as
    probabilities (if the number of weights is stricly one below the
    number of available states, the last state getting the complement
    to 1), or as true weights which are then normalized to be used as
    probabilities.

    Parameters:
    -----------
    machine_name: str
        the name of the state machine where the states must be chosen
        among the *N* non-autoremove states.
    weights: list
        a list of *N* or *N-1* model expressions assumed to produce positive numbers
    model: EmulsionModel
        the model where this function is defined

    Returns
    -------
    lambda: a function that returns a random state according to the
        values of the weights list, interpreted either as
        probabilities (if size *N-1*) or as weights (if size *N*)
        which are then normalized to provide probabilities

    """
    machine = model.state_machines[machine_name]
    states = [state for state in machine.states if not state.autoremove]
    assert(len(states) - len(weights) <= 1)
    def weighted_random(agent):
        values = [agent.get_model_value(weight) for weight in weights]
        total = sum(values)
        if len(values) < len(states):
            assert(0 <= total <= 1)
            probas = values + [1 - total]
        else:
            probas = [v / total for v in values]
        return np.random.choice(states, p=probas)
    return weighted_random
```

# EMULSION

Epidemiological Multi-Level Simulation Framework

### Navigation

- 1. Installation
- 2. Getting started with EMULSION
- 3. Modelling principles
- 4. Modelling language (basics)
- 5. Modelling language (advanced)
- 6. Feature examples
- 7. Information
- 8. License
- 9. High-level functions for model designers
- 10. emulsion package

### Related Topics

- Documentation overview
  - Module code

### Quick search

©2016, INRA and Univ. Lille.
|
Powered by Sphinx 1.8.5
& Alabaster 0.7.10
