# Supplementary material for: EMULSION: Transparent and flexible multiscale stochastic models in human, animal and plant epidemiology
Source: PLoS Comput Biol. 2019 Sep 13;15(9):e1007342. doi: 10.1371/journal.pcbi.1007342 (PMC6760811; doi:10.1371/journal.pcbi.1007342)
Supplement: S2 File — This zip file is a clone of EMULSION public Git repository. To install from this file rather than from PyPI, go to EMULSION documentation page: https://sourcesup.renater.fr/www/emulsion-public/pages/Install.html and follow Git-based installation instructions. (ZIP) [file pcbi.1007342.s010.zip › S2_file/doc/_modules/emulsion/tools/plot.html]

emulsion.tools.plot — EMULSION (Epidemiological Multi-Level Simulation framework)

# Source code for emulsion.tools.plot

```
"""A Python implementation of the EMuLSion framework (Epidemiologic
MUlti-Level SImulatiONs).

Plotting tools... to be improved!
"""

# [HEADER]

from   pathlib                      import Path

import sys
import pandas                       as pd
import numpy                        as np

# from   scipy.misc                   import imread
from   PIL                          import Image
from   xml.etree                    import ElementTree
from   bokeh.plotting               import figure, output_file, show
from   bokeh.models                 import Legend, ColumnDataSource, Range1d
from   bokeh.models.tools           import HoverTool, CrosshairTool
from   bokeh.models.glyphs          import ImageURL
from   bokeh.models.widgets         import Div
from   bokeh.layouts                import gridplot, column, widgetbox
from   bokeh.palettes               import all_palettes

[docs]def build_state_plot(counts, cols, machine, model, y='quantity', group='state',
                     ylab='Number of individuals'):
    """Return a bokeh figure based on the representation of the states in
    the *counts* dataframe, with the associated colors.

    Args:
        counts: a Pandas dataframe containing the values to plot
        cols (dict): dictionary which maps state/variable names to colors
        machine: the state machine to which the states are related
        model: the Emulsion model related to this plot
        y (str): the name of the field containing y values in the dataframe
        group (str): either 'state' (default) or 'variables', name of
          the field containing each legend items.

    Returns:
        A bokeh gridplot (reduced to one figure a the herd level, one
        figure per herd at metapopulation level).

    """
    WIDTH, HEIGHT = 800, 600
    NB_SIMU = 1 + counts.simu_id.max()
    alpha = max(1 /np.sqrt(NB_SIMU), 0.05)
    all_plots = []
    ncols = 1
    leg_pos, leg_orient = 'right', 'vertical'
    metapop = 'population_id' in counts.columns
    if metapop:
        max_pop_id = 1 + max(counts.population_id)
        ncols = 2
        WIDTH = 1200
    else:
        max_pop_id = 1
    for pop_id in range(max_pop_id):
        if metapop:
            df = counts[counts.population_id == pop_id]
        else:
            df = counts
        time_unit = model.time_unit
        origin = model.origin_date
        dur = model.step_duration
        df[time_unit] = pd.Series(model.delta_t * df['step'], index=df.index)
        # df['date'] = pd.Series(pd.to_datetime(origin + dur * df['step']), index=df.index)
        plot = figure(plot_width=WIDTH // ncols, plot_height=HEIGHT // ncols)
        legend_items = []
        for state in cols:
            state_items = []
            subdf = df[df[group] == state]
            if NB_SIMU <= 50:   # don't draw points if too many !
                ci = plot.circle(x=time_unit, y='quantity', source=ColumnDataSource(subdf),
                                 size=2, color=cols[state], alpha=alpha)
                state_items.append(ci)
            for simu_id in range(1+max(df.simu_id)):
                src = ColumnDataSource(subdf[subdf.simu_id == simu_id])
                pl = plot.line(x=time_unit, y='quantity', source=src, color=cols[state], alpha=alpha, line_width=2)
                state_items.append(pl)
            legend_items.append((state, state_items))
        plot.title.text = 'Evolution of %s (%s)' % (machine.replace('_', ' '), model.model_name)
        if metapop:
            plot.title.text += ' - population ID: {}'.format(pop_id)
        plot.xaxis.axis_label = 'Time (%s)' % (time_unit,)
        plot.yaxis.axis_label = ylab
        ylim = max(1, df['quantity'].max()*1.1)
        plot.y_range = Range1d(0, ylim)
        hover = HoverTool()
        hover.tooltips=[
            (group.capitalize(), '@%s' % (group,)),
            ('Quantity', '@quantity'),
            (time_unit.capitalize(), '@%s'%(time_unit,)),
            ('Step', '@step'),
            ('Simulation ID', '@simu_id'),
            # ('Population ID', pop_id)
        ]
        plot.add_tools(hover)
        plot.add_tools(CrosshairTool())
        legend = Legend(items=legend_items, location=(0, 0))
        legend.click_policy = 'hide'
        legend.orientation = leg_orient
        plot.add_layout(legend, leg_pos)
        all_plots.append(plot)
    return gridplot(all_plots, ncols=ncols, toolbar_location="above")


[docs]def build_machine_plot(machine_name, model, params):
    """Return a bokeh figure based on the representation of the state
    machine, with the associated colors.

    Args:
        machine: the state machine to which the states are related

    Returns:
        A bokeh gridplot.

    """
    # WIDTH, HEIGHT = 800, 600
    machine_url = model.model_name + '_' + machine_name + '_machine.' + params.img_format
    machine_path = Path(params.figure_dir, machine_url)
    try:
        # img = imread(machine_path)
        # height, width = img.shape[0:2]
        if params.img_format == 'svg':
            img = ElementTree.parse(machine_path)
            root = img.getroot()
            # retrieve dimensions (in pts)
            width = int(''.join(i for i in root.attrib['width'] if i.isdigit()))
            height = int(''.join(i for i in root.attrib['height'] if i.isdigit())) + 50
        else:
            img = Image.open(machine_path)
            width, height = img.size
    except:
        print("WARNING: Could not retrieve image dimensions")
        width, height = 800, 400

    div = Div(text='''<h1>State machine for {}</h1>
    <P><IMG SRC="{}"></P>
    <H2>Simulation outputs</H2>
    '''.format(machine_name, machine_url),
              width=width, height=height+100)

    return gridplot([widgetbox(div)], ncols=1)


[docs]def plot_outputs(params):
    """Read outputs from previous runs and plot the corresponding
    figures. In the *params* dictionary, `output_dir` is expected to
    contain a `counts.csv` file; `figure_dir` is where the plot is
    saved.

    """
    countpath = Path(params.output_dir, 'counts.csv')
    if not countpath.exists():
        print('ERROR, output file not found: %s' % (countpath, ))
        sys.exit(-1)
    model = params.model
    df = pd.read_csv(countpath)
    vars = ['simu_id', 'step', 'level', 'agent_id']
    if 'population_id' in df.columns:
        vars.append('population_id')
    vals = []
    all_figs = []
    figpath = Path(params.figure_dir, model.model_name + '.html')
    output_file(figpath, title='Emulsion Plot: %s' % (model.model_name,))
    for sm_name, state_machine in model.state_machines.items():
        if params.view_machines:
            all_figs.append(build_machine_plot(sm_name, model, params))
        col_dict = state_machine.state_colors
        # col_dict.update(population="black")
        states, cols = zip(*sorted(col_dict.items()))
        vals += [state.name for state in state_machine.states]
        df2 = pd.melt(df, id_vars=vars, value_vars=states,
                      var_name='state', value_name='quantity')
        fig = build_state_plot(df2, col_dict, sm_name, model)
        all_figs.append(fig)
        # figpath = Path(params.figure_dir,
        #                model.model_name + '_' + sm_name + '.png')
        # plot = build_state_plot(df2, cols, sm_name, model.model_name)
        # plot.save(str(figpath))
        # print('Saved figure %s' % (figpath, ))
    extras = [variable for variable in df.columns if variable not in vars + vals]
    if extras:
        df2 = pd.melt(df, id_vars=vars, value_vars=extras, value_name='quantity')
        idx = [(256 // len(extras))*i for i in range(len(extras))]
        cols=dict(zip(extras, [all_palettes['Plasma'][256][i] for i in idx]))
        fig = build_state_plot(df2, cols, 'Additional variables', model,
                               y='value', group='variable', ylab='Value')
        # plot = build_state_plot(df2, [], 'Additional variables',
        #                         model.model_name, y='value', group='variable')
        # plot.save(str(figpath))
        # print('Saved figure %s' % (figpath, ))
        all_figs.append(fig)
    # print(all_figs)
    print('Outputs plot in file: %s' % (figpath,))
    show(column(all_figs))
```

# EMULSION

Epidemiological Multi-Level Simulation Framework

### Navigation

- 1. Installation
- 2. Getting started with EMULSION
- 3. Modelling principles
- 4. Modelling language (basics)
- 5. Modelling language (advanced)
- 6. Feature examples
- 7. Information
- 8. License
- 9. High-level functions for model designers
- 10. emulsion package

### Related Topics

- Documentation overview
  - Module code

### Quick search

©2016, INRA and Univ. Lille.
|
Powered by Sphinx 1.8.5
& Alabaster 0.7.10
