# Supplementary material for: EMULSION: Transparent and flexible multiscale stochastic models in human, animal and plant epidemiology
Source: PLoS Comput Biol. 2019 Sep 13;15(9):e1007342. doi: 10.1371/journal.pcbi.1007342 (PMC6760811; doi:10.1371/journal.pcbi.1007342)
Supplement: S2 File — This zip file is a clone of EMULSION public Git repository. To install from this file rather than from PyPI, go to EMULSION documentation page: https://sourcesup.renater.fr/www/emulsion-public/pages/Install.html and follow Git-based installation instructions. (ZIP) [file pcbi.1007342.s010.zip › S2_file/doc/_modules/emulsion/tools/state.html]

emulsion.tools.state — EMULSION (Epidemiological Multi-Level Simulation framework)

# Source code for emulsion.tools.state

```
""".. module:: emulsion.tools.state

This module defines:

- a class ``StateVarDict`` which is a special auto-referential
  dictionary to handle state variables
- a class ``EmulsionEnum`` which is a special kind of enumeration
  intended to handle states from state machines

"""

# [HEADER]


from   enum                       import Enum
from   functools                  import total_ordering

[docs]class StateVarDict(dict):
    """A special dictionary aimed at handling the state variables of
    agents in EMULSION models. In addition to the classical dict
    key-based access, it provides an attribute-like access syntax.

    This class is used in EMULSION to store agent properties. Such
    properties include those defined automatically and used by the
    EMULSION engine, such as the values of the states for each state
    machine, the current time step, or "hidden" values such as the
    time spent in the current state for each state machine. They are
    also used to include user-defined attributes (e.g. age,
    weight...).

    When searching for a model ``statevar``, the engine tries first to
    find a classical instance variable (which can be mimicked by a
    Python ``@property``-decored function), then looks inside the
    agent's ``statevar`` attribute; finally, the search continues in
    the agent's host (if any).

    Example
    -------

    .. code-block:: python

        s = StateVarDict(age=10, sick=True)
        s['age'] += 1
        s.sick = False
        s.new_property = 'Wow !'

    """

[docs]    def __init__(self, *args, **kwargs):
        super().__init__(*args, **kwargs)
        # make self its own attributes dictionary (nice, hey ?)
        self.__dict__ = self


[docs]@total_ordering
class EmulsionEnum(Enum):
    """This class represents enumerations for states of state machines in
    EMULSION. They are endowed with some special features:

    #. They provide total ordering between items (based on ``__lt__``
       and ``__eq__`` methods).
    #. A comparison with ``None`` is provided (any state is always
       greater than ``None``).
    #. Other features will be developed soon.

    """

    def __lt__(self, other):
        """Return a less-than comparison for enumeration items. An enum item
        is always greater than ``None``and than any other item of
        another enumeration. Then, the ``value`` attribute of the item
        is used.

        Parameters
        ----------
        other: EmulsionEnum
            Another state from the same enumeration (or ``None``)

        Returns
        -------
        boolean:
            True if the current state is less than *other*
            (i.e. defined before *other* in the list of states), False
            otherwise (including the case where *other* is ``None``).

        """
        return False if other is None or other.__class__ != self.__class__\
          else self.value < other.value

    def __eq__(self, other):
        """Return a equality comparison for enumeration items. An enum item is
        always greater than ``None``and than any other item of another
        enumeration. Then, the ``value`` attribute of the item is
        used.

        Parameters
        ----------
        other: EmulsionEnum
            Another state from the same enumeration (or ``None``)

        Returns
        -------
        boolean:
            True if the current state is equal to *other* (having the
            same value), False otherwise (including the case where
            *other* is ``None``).

        """
        return False if other is None or other.__class__ != self.__class__\
          else self.value == other.value

    def __int__(self):
        """Return the *int* value mapped to this item."""
        return self.value

    def __repr__(self):
        """Return a string representation of the instances of the enumeration,
        hiding associated numerical value.

        """
        return f'<{self.__class__.__name__}.{self.name}>'

    def __hash__(self):
        """Use the value as hashcode."""
        return self.value
```

# EMULSION

Epidemiological Multi-Level Simulation Framework

### Navigation

- 1. Installation
- 2. Getting started with EMULSION
- 3. Modelling principles
- 4. Modelling language (basics)
- 5. Modelling language (advanced)
- 6. Feature examples
- 7. Information
- 8. License
- 9. High-level functions for model designers
- 10. emulsion package

### Related Topics

- Documentation overview
  - Module code

### Quick search

©2016, INRA and Univ. Lille.
|
Powered by Sphinx 1.8.5
& Alabaster 0.7.10
