# Supplementary material for: EMULSION: Transparent and flexible multiscale stochastic models in human, animal and plant epidemiology
Source: PLoS Comput Biol. 2019 Sep 13;15(9):e1007342. doi: 10.1371/journal.pcbi.1007342 (PMC6760811; doi:10.1371/journal.pcbi.1007342)
Supplement: S2 File — This zip file is a clone of EMULSION public Git repository. To install from this file rather than from PyPI, go to EMULSION documentation page: https://sourcesup.renater.fr/www/emulsion-public/pages/Install.html and follow Git-based installation instructions. (ZIP) [file pcbi.1007342.s010.zip › S2_file/doc/_modules/emulsion/tools/timing.html]

emulsion.tools.timing — EMULSION (Epidemiological Multi-Level Simulation framework)

# Source code for emulsion.tools.timing

```
"""A Python implementation of the EMuLSion framework (Epidemiologic
MUlti-Level SImulatiONs).

Tools for performance assessment.
"""

# [HEADER]


import time
from   functools                  import wraps

[docs]def timethis(times=None):
    """A decorator function for printing execution time."""
    def timefunc(func):
        """The actual decorator"""
        @wraps(func)
        def wrapper(*args, **kwargs):
            """Decorate the specified function in order to measure its
            execution time. Execution times are stored in the TIMES
            dictionary.

            """
            start = time.perf_counter()
            result = func(*args, **kwargs)
            end = time.perf_counter()
            if times is None:
                print('{}.{} : {}'.format(func.__module__,
                                          func.__name__,
                                          end - start))
            else:
                if func.__name__ not in times:
                    times[func.__name__] = [end-start]
                else:
                    times[func.__name__].append(end-start)
            return result
        return wrapper
    return timefunc
```

# EMULSION

Epidemiological Multi-Level Simulation Framework

### Navigation

- 1. Installation
- 2. Getting started with EMULSION
- 3. Modelling principles
- 4. Modelling language (basics)
- 5. Modelling language (advanced)
- 6. Feature examples
- 7. Information
- 8. License
- 9. High-level functions for model designers
- 10. emulsion package

### Related Topics

- Documentation overview
  - Module code

### Quick search

©2016, INRA and Univ. Lille.
|
Powered by Sphinx 1.8.5
& Alabaster 0.7.10
