# Supplementary material for: EMULSION: Transparent and flexible multiscale stochastic models in human, animal and plant epidemiology
Source: PLoS Comput Biol. 2019 Sep 13;15(9):e1007342. doi: 10.1371/journal.pcbi.1007342 (PMC6760811; doi:10.1371/journal.pcbi.1007342)
Supplement: S2 File — This zip file is a clone of EMULSION public Git repository. To install from this file rather than from PyPI, go to EMULSION documentation page: https://sourcesup.renater.fr/www/emulsion-public/pages/Install.html and follow Git-based installation instructions. (ZIP) [file pcbi.1007342.s010.zip › S2_file/doc/_modules/emulsion/agent/meta.html]

emulsion.agent.meta — EMULSION (Epidemiological Multi-Level Simulation framework)

# Source code for emulsion.agent.meta

```
"""A Python implementation of the EMuLSion framework (Epidemiologic
MUlti-Level SImulatiONs).

Classes and functions for abstract agent management.

Part of this code was adapted from the PADAWAN framework (S. Picault,
Univ. Lille).
"""

# [HEADER]


import abc

from   sortedcontainers           import SortedSet


#  __  __      _                                 _
# |  \/  |    | |          /\                   | |
# | \  / | ___| |_ __ _   /  \   __ _  ___ _ __ | |_
# | |\/| |/ _ \ __/ _` | / /\ \ / _` |/ _ \ '_ \| __|
# | |  | |  __/ || (_| |/ ____ \ (_| |  __/ | | | |_
# |_|  |_|\___|\__\__,_/_/    \_\__, |\___|_| |_|\__|
#                                __/ |
#                               |___/

################################################################
# Metaclass for all agents
################################################################

[docs]class MetaAgent(abc.ABCMeta):
    """The Metaclass definition for all agents. When created, agents
    are stored in a class-specific dictionaries of agents. They are
    given an ID value (unique value within each class) and can be
    assigned to several agents families (by default, each agent is
    assigned to its own class).

    """
    @classmethod
    def __prepare__(mcs, name, bases, **kwds):
        families = SortedSet()
        families.add(name)
        attrs = {'agcount': 0,               # number of instances created
                 'agdict': {},               # dict of instances (ID -> agent)
                 'families': families}        # families where the class belongs
        # state NB: no need to keep information on passivity since it
        # depends on the families of the agent
        return attrs

    def __new__(mcs, name, bases, attrs, **_):
        attrs = dict(attrs)
        result = super(MetaAgent, mcs).__new__(mcs, name, bases, dict(attrs))
        result.members = tuple(attrs)
        return result
```

# EMULSION

Epidemiological Multi-Level Simulation Framework

### Navigation

- 1. Installation
- 2. Getting started with EMULSION
- 3. Modelling principles
- 4. Modelling language (basics)
- 5. Modelling language (advanced)
- 6. Feature examples
- 7. Information
- 8. License
- 9. High-level functions for model designers
- 10. emulsion package

### Related Topics

- Documentation overview
  - Module code

### Quick search

©2016, INRA and Univ. Lille.
|
Powered by Sphinx 1.8.5
& Alabaster 0.7.10
