# Supplementary material for: EMULSION: Transparent and flexible multiscale stochastic models in human, animal and plant epidemiology
Source: PLoS Comput Biol. 2019 Sep 13;15(9):e1007342. doi: 10.1371/journal.pcbi.1007342 (PMC6760811; doi:10.1371/journal.pcbi.1007342)
Supplement: S2 File — This zip file is a clone of EMULSION public Git repository. To install from this file rather than from PyPI, go to EMULSION documentation page: https://sourcesup.renater.fr/www/emulsion-public/pages/Install.html and follow Git-based installation instructions. (ZIP) [file pcbi.1007342.s010.zip › S2_file/doc/_modules/emulsion/agent/core/groups.html]

emulsion.agent.core.groups — EMULSION (Epidemiological Multi-Level Simulation framework)

# Source code for emulsion.agent.core.groups

```
"""
.. module:: emulsion.agent.core.groups

.. moduleauthor:: Sébastien Picault <sebastien.picault@inra.fr>

Part of this code was adapted from the PADAWAN framework (S. Picault,
Univ. Lille).
"""

# [HEADER]

import abc

from   emulsion.agent.core.emulsion_agent  import EmulsionAgent

#   _____                                               _
#  / ____|                        /\                   | |
# | |  __ _ __ ___  _   _ _ __   /  \   __ _  ___ _ __ | |_
# | | |_ | '__/ _ \| | | | '_ \ / /\ \ / _` |/ _ \ '_ \| __|
# | |__| | | | (_) | |_| | |_) / ____ \ (_| |  __/ | | | |_
#  \_____|_|  \___/ \__,_| .__/_/    \_\__, |\___|_| |_|\__|
#                        | |            __/ |
#                        |_|           |___/

[docs]class GroupAgent(EmulsionAgent):
    """A GroupAgent is aimed at representing a group of agents. The
    underlying level may be either explicitly represented (using a
    ViewCompartment) or aggregated (using an Compartment). Each
    compartment is associated with keys, i.e. a tuple of state
    variables (possibly empty) which play a crucial role in this
    compartment.

    """

[docs]    def __init__(self, keys=(), **others):
        super().__init__(**others)
        self.keys = keys
        self.autoremove = False


[docs]    @abc.abstractmethod
    def add(self, population):
        """Add the specified population to the current compartment."""
        pass


[docs]    @abc.abstractmethod
    def remove(self, population):
        """Remove the specified population from the current
        compartment.

        """
        pass

@abc.abstractmethod
    def _base_move(self, other_unit, **others):
        pass

    def _before_move(self, state_machine, old_state, new_state, **others):
        # execute actions when exiting current state (if any)
        self.do_state_actions('on_exit',
                              state_machine,
                              old_state,
                              **others)
        # execute actions when crossing edge (if any)
        self.do_edge_actions(**others)
        # update states of atom units
        if 'agents' in others:
            for unit in others['agents']:
                unit.change_state(state_machine.machine_name,
                                  state_machine.states[new_state])
                # unit.statevars[state_machine.machine_name] =\
                #   state_machine.states[new_state]

    def _after_move(self, state_machine, new_state, **others):
        # execute actions when entering new state (if any)
        self.do_state_actions('on_enter',
                              state_machine,
                              new_state,
                              **others)

[docs]    def move_to(self, other_unit, state_machine=None, **others):
        """Move the specified population from the current population
        of the compartment to the other unit. If a state machine is
        provided, executes the corresponding actions when
        entering/exiting nodes and crossing edges if needed.

        """
        if state_machine:
            #old_state = self.get_information(state_machine.machine_name).name
            old_state = self.statevars[state_machine.machine_name]
            new_state = other_unit.statevars[state_machine.machine_name].name
            if old_state is not None:
                self._before_move(state_machine, old_state.name,
                                  new_state, **others)
        # move population from current compartment to other unit
        self._base_move(other_unit, **others)
        if state_machine:
            other_unit._after_move(state_machine, new_state, **others)

#                                           _   _
#     /\                                   | | (_)
#    /  \   __ _  __ _ _ __ ___  __ _  __ _| |_ _  ___  _ __
#   / /\ \ / _` |/ _` | '__/ _ \/ _` |/ _` | __| |/ _ \| '_ \
#  / ____ \ (_| | (_| | | |  __/ (_| | (_| | |_| | (_) | | | |
# /_/    \_\__, |\__, |_|  \___|\__, |\__,_|\__|_|\___/|_| |_|
#           __/ | __/ |          __/ |
#          |___/ |___/          |___/

[docs]class Aggregation(GroupAgent):
    """An Aggregation is aimed at grouping agents from the underlying
    level. Thus, aggregate information such as the total number of
    agents in the compartment (population) is calculated from the
    actual content of the compartment. The evolution of a
    ViewCompartment, by default, consists in making the units
    contained in the compartment evolve themselves, unless
    `recursive=False` is specified during instantiation.

    """

[docs]    def __init__(self, recursive=True, **others):
        super().__init__(**others)
        self._content = None
        self.recursive = recursive

@property
    def population(self):
        """Return the total population of the compartment. It is
        calculated either using a true 'population' statevar if any,
        or as the sum of the population of each unit contained in the
        compartment.

        TAG: USER
        """
        ## DEBUG
        # print('\t\tstatevars.population:', self.statevars.population if 'population' in self.statevars else 'None', [f'{unit.agid}->{unit._host}' for unit in self])
        return self.statevars.population if 'population' in self.statevars\
            else sum([unit.get_information('population')
                      for unit in self])

[docs]    def add(self, population):
        """Add the specified population to the current compartment."""
        for unit in population:
            unit.add_host(self)


[docs]    def remove(self, population):
        """Remove the specified population from the current
        compartment.

        """
#        print([u.agid for u in population])
        for unit in population:
            unit.remove_host(self, keys=self.keys)

def _base_move(self, other_unit, agents=[], **others):
        self.remove(agents)
        other_unit.add(agents)


    @abc.abstractmethod
    def __iter__(self):
        pass

    # TODO: shuffle content first

[docs]    def evolve(self, machine=None):
        """Ask each unit in the current compartment to make its content evolve
        according to its own capabilities. A specific state machine
        can be specified if needed.

        """
        super().evolve(machine=machine)
        if self.recursive:
            for unit in self:
                unit.evolve(machine=machine)
```

# EMULSION

Epidemiological Multi-Level Simulation Framework

### Navigation

- 1. Installation
- 2. Getting started with EMULSION
- 3. Modelling principles
- 4. Modelling language (basics)
- 5. Modelling language (advanced)
- 6. Feature examples
- 7. Information
- 8. License
- 9. High-level functions for model designers
- 10. emulsion package

### Related Topics

- Documentation overview
  - Module code

### Quick search

©2016, INRA and Univ. Lille.
|
Powered by Sphinx 1.8.5
& Alabaster 0.7.10
