# Supplementary material for: EMULSION: Transparent and flexible multiscale stochastic models in human, animal and plant epidemiology
Source: PLoS Comput Biol. 2019 Sep 13;15(9):e1007342. doi: 10.1371/journal.pcbi.1007342 (PMC6760811; doi:10.1371/journal.pcbi.1007342)
Supplement: S2 File — This zip file is a clone of EMULSION public Git repository. To install from this file rather than from PyPI, go to EMULSION documentation page: https://sourcesup.renater.fr/www/emulsion-public/pages/Install.html and follow Git-based installation instructions. (ZIP) [file pcbi.1007342.s010.zip › S2_file/doc/_modules/emulsion/agent/core/emulsion_agent.html]

emulsion.agent.core.emulsion\_agent — EMULSION (Epidemiological Multi-Level Simulation framework)

# Source code for emulsion.agent.core.emulsion\_agent

```
"""
.. module:: emulsion.agent.core.emulsion_agent

.. moduleauthor:: Sébastien Picault <sebastien.picault@inra.fr>

Part of this code was adapted from the PADAWAN framework (S. Picault,
Univ. Lille).
"""

# [HEADER]

import abc

import numpy                      as np


from   emulsion.tools.graph       import EdgeTypes
from   emulsion.model             import EDGE_KEYWORDS
from   emulsion.tools.misc        import rates_to_probabilities,\
    aggregate_probabilities, probabilities_to_rates, count_population, AGENTS

from   emulsion.agent.core.abstract_agent  import AbstractAgent


#  ______                 _     _                                      _
# |  ____|               | |   (_)               /\                   | |
# | |__   _ __ ___  _   _| |___ _  ___  _ __    /  \   __ _  ___ _ __ | |_
# |  __| | '_ ` _ \| | | | / __| |/ _ \| '_ \  / /\ \ / _` |/ _ \ '_ \| __|
# | |____| | | | | | |_| | \__ \ | (_) | | | |/ ____ \ (_| |  __/ | | | |_
# |______|_| |_| |_|\__,_|_|___/_|\___/|_| |_/_/    \_\__, |\___|_| |_|\__|
#                                                      __/ |
#                                                     |___/

[docs]class EmulsionAgent(AbstractAgent):
    """The EmulsionAgent is the base class for multi-level
    epidemiologic agents. An EmulsionAgent can represent any entity
    involved in the epidemiologic model (individual, compartment,
    herd, [meta]population, etc.).

    Each agent contains an exchange box (in & out) which is composed
    of a list of messages. A message is generally a dictionary of
    Source/Destination (In/Out) and content of exchange with
    Source/Destination agent.

    """

[docs]    def __init__(self, model=None, level=None, name=None, host=None,
                 simulation=None, **others):
        """Initialize the unit with a health state and a name."""
        super().__init__(**others)
        # self.statevars.health_state = health_state
        self.model = model
        self.level = level
        self._name = name
        self._host = host
        self.simulation = simulation
        if 'step' not in self.statevars:
            self.statevars.step = 0
        self.statevars.creation_step = self.statevars.step
        # exchange inbox/outbox
        self._inbox = []
        self._outbox = []


[docs]    def upper_level(self, init=True):
        """Return the 'upper level' for this agent, i.e. the first host with
        a not-None level attribute.

        TAG: USER
        """
        if self.level is not None and not init:
            return self
        return self.get_host().upper_level(init=False)


[docs]    def top_level(self):
        """Return the highest level used in the simulation"""
        if self.get_host() is None:
            return self
        return self.upper_level().top_level()


[docs]    def log_path(self):
        """Return the path to the log file for the current simulation"""
        return self.top_level().simulation.log_path()

@property
    def name(self):
        """Return the name of the unit. If no name was provided during
        instantiation,  the class name is returned.

        TAG: USER
        """
        return self._name if self._name is not None\
            else super().__repr__() # self.__class__.__name__

    def __repr__(self):
        return self.name

[docs]    def evolve(self, machine=None):
        """This method is aimed at defining what has systematically to
        be done in the unit at each time step (e.g. age change...). It
        has to be overriden if needed in subclasses.

        """
        self.statevars.step += 1


[docs]    def get_host(self, key=None):
        """Return the host of the current unit.

        TAG: USER
        """
        return self._host


[docs]    @abc.abstractmethod
    def get_content(self):
        """Return either the population (number) of the current unit,
        or the list of agents contained in the current unit. The
        output is a tuple with either ('population', qty) or ('agents', list).

        """
        pass


[docs]    def do_state_actions(self, event, state_machine, state_name,
                         population=None, agents=None, **_):
        """Perform the actions associated to the current state. If the
        unit is a ViewAgent, actions are actually performed by each
        unit of the specified agents list, in turn. Otherwise, the
        actions are performed according to the population, which is
        expected to be a number.

        """
        # if actions are actually associated to the current state of
        # the state machine...
           # ... and to the 'event' (enter/exit/stay)
        if state_name in state_machine.state_actions\
           and event in state_machine.state_actions[state_name]:
            # print(f'Doing state actions {event} for {state_machine}\n\tfor {state_name}')
            # retrieve the list of actions
            l_actions = state_machine.state_actions[state_name][event]
            # ask the current unit to perform the actions with the
            # specified population
            # print(f'\t{l_actions}')
            for action in l_actions:
                action.execute_action(self, population=population, agents=agents)


[docs]    def do_edge_actions(self, actions=None, population=None, agents=None):
        """Perform the actions associated to a transition between
        states. If the unit is a ViewCompartment, actions are actually
        performed by each unit of the specified agents list, in
        turn. Otherwise, the actions are performed according to the
        population, which is expected to be a number.

        """
        # # if actions are actually associated to the current edge...
        #    # ... and to the 'event' (cross)
        # if from_ in state_machine.edge_actions\
        #    and to_ in state_machine.edge_actions[from_]\
        #    and event in state_machine.edge_actions[from_][to_]:
        #     # retrieve the list of actions
        #     l_actions = state_machine.edge_actions[from_][to_][event]
        #     # ask the current unit to perform the actions with the
        #     # specified population
        for action in actions:
#            print(action)
            action.execute_action(self, population=population, agents=agents)


[docs]    def next_states_from(self, initial_state, state_machine):
        """Return a list of tuples composed of:

        - each of the possible states reachable from the specified
        initial state (some depending on a condition)

        - the transition rate, probability or amount to each state in
        the specified state_machine (possible clauses: 'rate',
        'proba', 'amount', 'amount-all-but' with the actual value)

        - a tuple indicating who can cross the edge, depending on
        conditions (either ('population', qty) or ('agents', list))

        - the list of actions to perform when crossing the edge (if
        any).

        The conditions, rates and probabilities may depend on the state
        variables or properties of the current unit.

        """
        result = []
        # remove unfulfilled 'when' clauses if any
        for (state, props) in state_machine.graph.edges_from(initial_state,
                                                             type_id=EdgeTypes.TRANSITION):
            if 'when' in props:
                when = state_machine.get_value(props['when'])
                fulfilled = when(self) if callable(when) else when
                if not fulfilled:
                    continue
            cond_result = self.get_content()
            # if any condition, evaluate it
            if 'cond' in props:
                cond = state_machine.get_value(props['cond'])
                # compute the content tuple (either ('population', qty)
                # or ('agents', lsit)) which fulfils the condition
                cond_result = self.evaluate_condition(cond)
            # only edges with condition fulfilled are taken into account
            if cond_result:
                # print(cond_result)
                flux = None
                for keyword in EDGE_KEYWORDS:
                    if keyword in props:
                        flux = keyword
                        break
                value = state_machine.get_value(props[flux])
                actions = props['actions'] if 'actions' in props else []
                if callable(value):
                    value = value(self)
                if value > 0 or flux == 'amount-all-but':
                    result.append((state, flux, value, cond_result, actions))
        return result


[docs]    def production_from(self, initial_state, state_machine):
        """Return a list of tuples composed of:

        - each of the possible states that can be produced from the specified
        initial state (some depending on a condition)

        - the transition rate, probability or amount to each state in
        the specified state_machine (possible clauses: 'rate',
        'proba', 'amount', 'amount-all-but' with the actual value)

        - a tuple indicating who can produce cross the edge, depending on
        conditions (either ('population', qty) or ('agents', list))

        - the prototypes of agents produced through the edge (if
        any).

        The conditions, rates and probabilities may depend on the state
        variables or properties of the current unit.

        """
        result = []
        # remove unfulfilled 'when' clauses if any
        for (state, props) in state_machine.graph.edges_from(initial_state,
                                                             type_id=EdgeTypes.PRODUCTION):
            if 'when' in props:
                when = state_machine.get_value(props['when'])
                fulfilled = when(self) if callable(when) else when
                if not fulfilled:
                    continue
            cond_result = self.get_content()
            # if any condition, evaluate it
            if 'cond' in props:
                cond = state_machine.get_value(props['cond'])
                # compute the content tuple (either ('population', qty)
                # or ('agents', lsit)) which fulfils the condition
                cond_result = self.evaluate_condition(cond)
            # only edges with condition fulfilled are taken into account
            if cond_result:
                # print(cond_result)
                flux = None
                for keyword in EDGE_KEYWORDS:
                    if keyword in props:
                        flux = keyword
                        break
                value = state_machine.get_value(props[flux])
                protos = props['prototype'] if 'prototype' in props else None
                if callable(value):
                    value = value(self)
                if value > 0 or flux == 'amount-all-but':
                    result.append((state, flux, value, cond_result, protos))
        return result

def _compute_production(self, value, flux, pop_size, stochastic):
        """Compute the values of production edges.

        """
        # ALL PRODUCTIONS COME FROM THE SAME POPULATION
        # (reference_pop) The `values` can represent : 1) amounts - in
        # that case no transformation occurs, 2) probabilities - in
        # that case the values must be converted to rates if the
        # target compartment is deterministic, otherwise the step
        # duration must be taken into account; 3) rates - computed
        # using Poisson distribution if the target compartment is
        # stochastic
        if flux == 'amount-all-but':
            amount = max(0, pop_size - value)
        elif flux == 'amount':
            amount = value
        elif flux == 'proba':
            # if stochastic uses binomial
            if stochastic:
                # aggregate probability wrt the time step duration
                value = aggregate_probabilities([value], self.model.delta_t)[0]
                amount = np.random.binomial(pop_size, value)
            else:
                # otherwise treat proba as proportion
                amount = pop_size * value
        elif flux == 'rate':
            # if stochastic: use Poisson distribution
            if stochastic:
                amount = np.random.poisson(value * pop_size * self.model.delta_t)
                # amount = np.random.binomial(pop_size, 1-np.exp(-value * self.model.delta_t))
            else:
                # consider rate as a speed
                # S = S0 exp(rate * dt) => S += S * (exp(rate*dt) - 1)
                # amount = value * pop_size * self.model.delta_t
                amount = pop_size * (np.exp(value * self.model.delta_t) - 1)
        else:                   # should not happen !
            amount = None
        return amount


    def _compute_values_for_unique_population(self,
                                              values,
                                              flux,
                                              reference_pop,
                                              stochastic):
        """Restructure the values according to the situation, for
        edges concerning the same population.

        """
        # ALL TRANSITIONS AFFECT THE SAME POPULATION (reference_pop)
        # The `values` can represent : 1) amounts - in that case no
        # transformation occurs, 2) probabilities - in that case the
        # values must be converted to rates if the target compartment
        # is deterministic, otherwise the step duration must be taken
        # into account; 3) rates - in that case the values must be
        # converted to probabilities if the target compartment is
        # stochastic
        # print('IDENTICAL')
        available_flux = set(flux)
        # try:
        assert len(available_flux) == 1 or available_flux == set(['amount', 'amount-all-but'])
        # except:
        #     print(available_flux)

        method = None
        if 'amount' in available_flux or 'amount-all-but' in available_flux:
            # handle values as amounts
            method = 'amount'
            total_ref_pop = count_population(reference_pop)
            # check that values are between 0 and the population size,
            # if needed invert 'amount-all-but' values
            values = tuple([max(0, min(total_ref_pop-v, total_ref_pop))\
                              if f == 'amount-all-but'\
                              else max(0, min(v, total_ref_pop))
                            for (f, v) in zip(flux, values)])
            # when finished the length of values is the number of
            # outgoing edges
            # print('AMOUNT', values)
        elif 'proba' in available_flux:
            if not stochastic:
                # then if the target compartment is deterministic,
                # probabilities must be converted into rates
                # print('PROBA -> RATES', values)
                values = probabilities_to_rates(values + (1 - sum(values),))
                # when finished the length of values is the number of
                # outgoing edges
                # print(values)
            else:
                # aggregate probabilities wrt the time step duration
                values = aggregate_probabilities(values, self.model.delta_t)
                values = values + (1 - sum(values),)
                # when finished the length of values is the number of
                # outgoing edges + 1
                # print('PROBA', values)
        elif not stochastic:
            # print('RATES', values)
            pass
        else:
            # otherwise values are transformed from rates to
            # probabilities
            values = rates_to_probabilities(sum(values),
                                            values,
                                            delta_t=self.model.delta_t)
            # when finished the length of values is the number of
            # outgoing edges + 1
            # print("RATES -> PROBAS", values)
        return values, method

    def _compute_values_for_multiple_populations(self,
                                                 values,
                                                 flux,
                                                 populations,
                                                 stochastic):
        """Restructure the values according to the situation, for
        edges concerning distinct populations.

        """
        # IN THAT CASE, EACH POSSIBLE TRANSITION IS RELATED TO A
        # SPECIFIC SUBGROUP OF THE COMPART.
        ### IMPORTANT: we assume that all conditions are disjoint,
        ### i.e. there is no intersection between the populations. IF
        ### NOT THE CASE, this means that the model is not really
        ### consistent, and the calculation of probabilities should be
        ### done on each individual... thus why use a StructuredView
        ### instead of a set of EvolvingAtom ???
        # print('MULTIPLE')
        pop_sets = [SortedSet(pop[AGENTS]) for pop in populations]
        # pop_sets must be disjoint
        assert(not any([pop_sets[i] & pop_sets[j]
                        for i in range(len(pop_sets)-1)
                        for j in range(i+1, len(pop_sets))]))
        # binomial values must be computed for each transition to
        # determine how many units among the candidates will cross the
        # transition, thus we need probabilities. If all values are
        # given as rates, they must be transformed
        method = None
        available_flux = set(flux)
        # try:
        assert len(available_flux) == 1 or available_flux == set(['amount', 'amount-all-but'])
        # except:
        #     print(available_flux)

        if available_flux == {'rate'} and stochastic:
            # values are transformed from rates to probabilities
            values = rates_to_probabilities(sum(values),
                                            values,
                                            delta_t=self.model.delta_t)
            # print('RATES -> PROBAS', values)
        elif 'amount' in available_flux or 'amount-all-but' in available_flux:
            method = 'amount'
            pops = [len(p) for p in pop_sets]
            values = tuple([max(0, min(pop-v, pop))\
                              if f == 'amount-all-but'\
                              else max(0, min(v, pop))
                            for (f, v, pop) in zip(flux, values, pops)])
            values = values #+ (1 - sum(values),)
            # print('AMOUNTS:', values)
        elif 'proba' in available_flux:
            if stochastic:
                # values must be aggregated wrt the times step duration
                values = aggregate_probabilities(values,
                                                 delta_t=self.model.delta_t)
                values = values + (1 - sum(values),)
                # print('PROBAS:', values)
            else:
                # values must be transformed from probabilities to rates
                # print('PROBABILITIES -> RATES', values)
                values = probabilities_to_rates(values + (1 - sum(values),))
                # print(values)
        else:
            print('ERROR - DETERMINISTIC COMPARTMENT MODEL MUST NOT'
                  ' TRY TO HANDLE SEPARATE SUBPOPULATIONS')
        return values, method

[docs]    def evaluate_condition(self, condition):
        """Return the content (tuple either ('population', qty) or
        ('agents', list)) if the condition is fulfilled, () otherwise.

        """
        if callable(condition):
            condition = condition(self)
        return self.get_content() if condition else ()


[docs]    def evaluate_event(self, name):
        """Evaluate if the specified event name is fulfilled, using a
        calendar. The calendar is determined dynamically according to
        the name of the event.

        TAG: USER
        """
        # print(self, 'evaluating event', name, "at", self.statevars.step)
        return self.model.get_calendar_for_event(name)[name](self.statevars.step)


[docs]    def is_in_state(self, state_name):
        """Evaluate if the agent is in the specified state. The state name is
        expected to be a valid state of one of the state machines. If
        so, the name of the state machine is expected to be one of the
        statevars of the agent.

        """
        # retrieve name of the state machine associated with the state_name
        varname = self.get_model_value(state_name).state_machine.machine_name
        result = self.statevars[varname].name == state_name\
                 if varname in self.statevars else False
        # print(self, 'testing var:', varname, ' = ', result)
        return result

# def time_before_event(self, event_name):
    #     """Return the time to wait from the current time to the specified
    #     *event_name*.
    #     """
    #     pass self.model.get_cal

[docs]    def get_outbox(self):
        """Return the content of the outbox.

        TAG: USER
        """
        return self._outbox


[docs]    def add_outbox(self, message):
        """Appendd a message to the outbox.

        TAG: USER
        """
        return self._outbox.append(message)


[docs]    def reset_outbox(self):
        """Reset the content of the outbox.

        TAG: USER
        """
        self._outbox = []


[docs]    def add_inbox(self, messages=[]):
        """Add the specified list of messages in the inbox.

        TAG: USER"""
        self._inbox += messages


[docs]    def clean_inbox(self):
        """Remove non sticky messages from the inbox.

        TAG: USER
        """
        self._inbox = [message for message in self._inbox if message.get('sticky')]


[docs]    def checkout_inbox(self):
        """Pick up agent's inbox content.

        TAG: USER
        """
        for message in self._inbox:
            for name, value in message.items():
                self.set_information(name, value)
```

# EMULSION

Epidemiological Multi-Level Simulation Framework

### Navigation

- 1. Installation
- 2. Getting started with EMULSION
- 3. Modelling principles
- 4. Modelling language (basics)
- 5. Modelling language (advanced)
- 6. Feature examples
- 7. Information
- 8. License
- 9. High-level functions for model designers
- 10. emulsion package

### Related Topics

- Documentation overview
  - Module code

### Quick search

©2016, INRA and Univ. Lille.
|
Powered by Sphinx 1.8.5
& Alabaster 0.7.10
