# Supplementary material for: EMULSION: Transparent and flexible multiscale stochastic models in human, animal and plant epidemiology
Source: PLoS Comput Biol. 2019 Sep 13;15(9):e1007342. doi: 10.1371/journal.pcbi.1007342 (PMC6760811; doi:10.1371/journal.pcbi.1007342)
Supplement: S2 File — This zip file is a clone of EMULSION public Git repository. To install from this file rather than from PyPI, go to EMULSION documentation page: https://sourcesup.renater.fr/www/emulsion-public/pages/Install.html and follow Git-based installation instructions. (ZIP) [file pcbi.1007342.s010.zip › S2_file/doc/_modules/emulsion/agent/core/abstract_agent.html]

emulsion.agent.core.abstract\_agent — EMULSION (Epidemiological Multi-Level Simulation framework)

# Source code for emulsion.agent.core.abstract\_agent

```
"""
.. module:: emulsion.agent.core.asbtract_agent

.. moduleauthor:: Sébastien Picault <sebastien.picault@inra.fr>

Part of this code was adapted from the PADAWAN framework (S. Picault,
Univ. Lille).
"""

# [HEADER]

from   functools                  import total_ordering

import numpy                      as np


from   emulsion.tools.state       import StateVarDict
from   emulsion.agent.meta        import MetaAgent
from   emulsion.agent.exceptions  import StateVarNotFoundException
from   emulsion.tools.misc        import retrieve_value


#           _         _                  _                            _
#     /\   | |       | |                | |     /\                   | |
#    /  \  | |__  ___| |_ _ __ __ _  ___| |_   /  \   __ _  ___ _ __ | |_
#   / /\ \ | '_ \/ __| __| '__/ _` |/ __| __| / /\ \ / _` |/ _ \ '_ \| __|
#  / ____ \| |_) \__ \ |_| | | (_| | (__| |_ / ____ \ (_| |  __/ | | | |_
# /_/    \_\_.__/|___/\__|_|  \__,_|\___|\__/_/    \_\__, |\___|_| |_|\__|
#                                                     __/ |
#                                                    |___/

################################################################
# superclass of all agents

[docs]@total_ordering
class AbstractAgent(object, metaclass=MetaAgent):
    """The Superclass for any multi-level agent. Due to the MetaAgent
    metaclass, all agents of the same class can be accessed through
    their ID, using the agdict class attribute. Agents are endowed
    with an automatic ID and possibly with a label. Agents also belong
    to families which can be chosen arbitrarily. By default, each
    agent belongs to the family named afer its own class.

    An agent is situated in one or more environments. Besides, agents
    can encapsulate environments where other agents can be
    situated. Agents are also endowed with State Variables, which can
    represent either properties of their own, or properties perceived
    from their inner environment or from the environments where they
    are situated.

    """

[docs]    @classmethod
    def from_dict(cls, dct):
        """Instantiate an agent using the specified dictionary.

        TAG: USER
        """
#        print('Instantiation of', cls.__name__, 'from dict:', dct)
        return cls(**dct)

def _register_instance(self, key=None):
        """Register the instance in the instance dictionary of the
        class. If no key is specified, the agent ID is used.

        """
        if key is not None:
            self._agkey = key
        self.__class__.agdict[self._agkey] = self

[docs]    def __init__(self, envt=None, content=None, **others):
        """Instantiate an agent. The instance is automatically added
        to the agentset of its own class. An arbitrary label can be
        specified to give the agent a label (otherwise a label is
        automatically computed using the class name and the agent
        ID).

        """
        super().__init__()
        self.__class__.agcount = self.__class__.agcount + 1
        # agent id
        self.agid = self.__class__.agcount
        # key used to register agents (agent ID by default)
        self._agkey = self.agid
        # environments in which the agent is situated
        self._envt = envt
        # environment encapsulated by the agent
        self._content = content
        # simulation where the agent belongs
        self.simulation = None
        # model used to define simulation behaviors
        self.model = None
        # "true" state variables of the agent
        self.statevars = StateVarDict({
            key: (value(self) if callable(value) else value)
            for key, value in others.items()
        })
        # refresh cache of class members
        self._reset_mbr_cache()

def _reset_mbr_cache(self):
        # Build a cache of regular class members. This cache is used
        # to make the 'get_information' method as efficient as
        # possible, thus this operations should be performed at the
        # end of instance creation (after other attributes have been
        # initialized)
        self._mbr_cache = set(name for name in dir(self)
                              if not name.startswith('_'))

    def __hash__(self):
        """Return a hashcode for the agent. The hashcode is actually
        the hashcode of the internal id of the object.

        """
        return hash(id(self))

    def __len__(self):
        """Return the 'size' of the agent. The size of the agent is based on
        the `_content` attribute : len() if __len__ exists, 0
        otherwise.

        """
        if self._content is None:
            return 0
        if hasattr(self._content, '__len__'):
            return len(self._content)
        return 0


    def __str__(self):
        return '{} #{}'.format(self.__class__.__name__, self.agid)

    __repr__ = __str__

    def __eq__(self, other):
        """Two agents are considered equal if they belong to the same
        class and have the same agent ID. (This is consistent with the
        fact that all instances are stored by each class using the
        agent ID as key.)

        """
        return self.__class__ == other.__class__\
            and self.agid == other.agid

    def __lt__(self, other):
        """Return an order for sorting agents. In this simple method
        the order is defined by sorting classes, then agents IDs.

        """
        return self.agid < other.agid if self.__class__ == other.__class__\
            else self.__class__.__name__ < other.__class__.__name__

[docs]    def get_model_value(self, name):
        """Return the value corresponding to the specified name in the model
        of the agent. If the name refers to a function, apply this
        function to the agent.

        TAG: USER
        """
        return retrieve_value(self.model.get_value(name), self)

@property
    def delta_t(self):
        """A shortcut to `self.model.delta_t`."""
        return self.model.delta_t

    @property
    def time(self):
        """The time elapsed since the beginning of simulation (in time
        units).

        """
        return self.model.delta_t * self.statevars.step

[docs]    def die(self):
        """Operation performed when the agent is removed from the
        simulation. Recursively destroy agents contained in the
        current agent if any, and remove the current agent from the
        agdict attribute of its class.

        """
        if self._content:
            for agent in self._content:
                agent.die()
        if self._agkey in self.__class__.agdict:
            del self.__class__.agdict[self._agkey]


[docs]    def get_information(self, name):
        """Return the value corresponding to the specified name in the
        agent. This value can be stored either as an attribute or a
        property-like descriptor (and thus accessed through an
        attribute-like syntax), or as a State Variable using a
        StateVarDict attribute named ``statevars``.

        Example:
        class Cow(AtomAgent):
            ...
            @property
            def age(self):
                return self._age

        c = Cow()
        c.get_information('age')
        # -> access through property 'age'
        c.get_information('health_state')
        # -> access through statevar 'health_state' (present in any Unit),
        # unless a 'health_state' attribute or property is explicitly
        # redefined to override the state variable


        TAG: USER
        """
        # return getattr(self, name) if hasattr(self, name)\
        #     else getattr(self.statevars, name)
        # REWRITTEN for efficiency improvement
        try:
            return getattr(self, name)\
                if name in self._mbr_cache\
                   else getattr(self.statevars, name)
        # do NOT use self.statevars[name] to ensure recursive lookup

        except AttributeError:
            if self._host is not None:
                # print('%s trying to find %s in %s' %(self, name, self.get_host()))
                return self.get_host().get_information(name)
                # print('Found: %s' % (result,))
            elif name in self.model._values:
                return self.get_model_value(name)
            else:
                # print(str(self), self._mbr_cache)
                raise StateVarNotFoundException(name, self)


[docs]    def set_information(self, name, value):
        """Set the specified value for the statevar/attribute.

        TAG: USER
        """
        # if hasattr(self, name):
        #     setattr(self, name, value)
        # else:
        #     setattr(self.statevars, name, value)
        # REWRITTEN for efficiency improvement
        if name in self._mbr_cache:
            setattr(self, name, value)
            # print(self, 'changed', name, 'to', value, 'through property')
        elif name in self.statevars:
            setattr(self.statevars, name, value)
            # do NOT use self.statevars[name]=value to ensure recursive lookup
            # print(self, 'changed', name, 'to', value, 'through statevar')
        elif self._host is not None:
            self.get_host().set_information(name, value)
        else:
            print(str(self), self._mbr_cache)
            raise StateVarNotFoundException(name, self)


[docs]    def init_time_entered(self, machine_name, advance=0, nb_timesteps=0):
        """Initialize the time step value when this agent is entering a new
        state of the specified state machine. A (positive) `advance`
        value can be provided to mimick an earlier entering in the
        state. By default, a `_time_to_exit` value is set according to
        the value of `nb_timesteps`.

        """
        key = '_time_entered_{}'.format(machine_name)
        key_TTE = '_time_to_exit_{}'.format(machine_name)
        self.statevars[key] = self.statevars.step - advance
        self.statevars[key_TTE] = self.statevars[key] + nb_timesteps

# print(value)

[docs]    def change_state(self, machine_name, new_state, do_actions=False):
        """Change the state of this agent for the specified state machine to
        `new_state`. The `_time_entered_MACHINE` value for this state machine
        is initialized. If do_actions is True, perform the actions to
        do on_exit from the previous state (if any) and those to do
        on_enter in the new state (if any).

        TAG: USER?
        """
        # retrieve the state machine from its name
        state_machine = self.model.state_machines[machine_name]
        # if asked to do actions, first execute the 'on_exit' actions
        # of current state
        if do_actions:
            current_state = self.statevars[machine_name]\
                            if machine_name in self.statevars else None
            if current_state is not None:
                self.do_state_actions('on_exit', state_machine, current_state.name)
        # change the state in the statevar
        self.statevars[machine_name] = new_state
        # initialize the _time_entered_STATEMACHINE variable
        self.init_time_entered(machine_name)
        # if asked to do actions, execute the 'on_enter' actions of
        # the new state
        if do_actions:
            # do on_enter actions associated to the new value
            # print(f'doing on_enter for {state_machine} in {new_state}')
            self.do_state_actions('on_enter', state_machine, new_state.name, agents=[self])

# DEPRECATED in >0.9.5
    # def init_time_to_live(self, machine_name, value):
    #     """Initialize the time this agent is expected to stay in the
    #     current state of the specified state machine. If an offset is
    #     defined for this state machine, it is added to the specified
    #     value, then reset to 0.

    #     """
    #     key = '_time_spent_{}'.format(machine_name)
    #     keymax = '_time_to_live_{}'.format(machine_name)
    #     keyoff = '_time_offset_{}'.format(machine_name)
    #     if keyoff not in self.statevars:
    #         self.statevars[keyoff] = 0
    #     self.statevars[key] = 0
    #     # compute the time to live w.r.t. the time step duration
    #     # (deltat_t)
    #     self.statevars[keymax] = int(np.round((value + self.statevars[keyoff])\
    #                                           / self.model.delta_t))
    #     self.statevars[keyoff] = 0
    #     # print(value)

[docs]    def update_time_to_exit(self, machine_name, duration):
        """Update the *duration* this agent is expected to stay in the current
        state of the specified state machine (*machine_name*).

        The time step value after which the agent is allowed to leave
        the current state is stored in a statevar called
        ``_time_to_exit_machine_name``, is computed from the
        current time (``step``) plus the *duration*.

        Args:
            machine_name (str): name of the state machine for which
              the duration in the current state must be updated
            duration (datetime.timedelta): the new duration for the
              agent to stay in the current state, calculated from the
              current time in the simulation
        """
        if (type(duration) == str): # DEBUG: should not happen !!!
            print(duration)
        key = '_time_to_exit_{}'.format(machine_name)
        # compute the time to live w.r.t. the time step duration
        # (deltat_t)
        nb_timesteps = int(np.round(duration / self.model.delta_t))
        self.statevars[key] = self.statevars.step + nb_timesteps

# print(value)


    # DEPRECATED in >0.9.5
    # def increase_time_spent(self, machine_name):
    #     """Increase the time this agent has spent in the current state of the
    #     specified state machine.

    #     """
    #     self.statevars['_time_spent_{}'.format(machine_name)] += 1

    # DEPRECATED in >0.9.5
    # def set_time_to_live_offset(self, machine_name, value):
    #     """Specify an additional value to the time this agent is
    #     expected to stay in the next state of the specified state
    #     machine.

    #     """
    #     self.statevars['_time_offset_{}'.format(machine_name)] = value

[docs]    def duration_in_current_state(self, machine_name: str) -> float:
        """Return the duration (in time units) this agent has spent in the
        current state of the specified state machine (*machine_name*).

        Args:
            machine_name: the name of the state machine for which
              the duration in the current state must be computed

        Returns:
          A duration (expressed in time units) corresponding to the
          duration spent in the current state for this state machine.
        """
        return (self.statevars.step -\
                self.statevars['_time_entered_{}'.format(machine_name)]) *\
            self.model.delta_t


[docs]    def apply_prototype(self, name=None, prototype=None, execute_actions=False):
        """Apply the prototype with the specified name to this agent. A
        prototype is basically a dictionary of statevars associated to
        values. Thus this method changes specific statevars to new
        values as required in the prototype. Other statevars are kept
        unchanged.  If a statevar was no amongst those of this agent,
        it is added. When statevars hold the state of a state machine,
        the state is changed with correct initialization of
        corresponding attributes (mainly _time_entered) ; if
        execute_actions is True, on_enter/on_exit actions are
        performed.

        TAG: USER
        """
        # give priority to named prototypes if provided, else
        if name is not None:
            prototype = self.model.get_prototype(self.level, name)
        if prototype is None:
            print("WARNING: agent {} was asked to apply inexistent prototype".format(self))
        # apply changes in states first, then other variables
        for var, value in prototype.items():
            if var in self.model.state_machines:
                val = value(self) if callable(value) else value
                self.change_state(var, val, do_actions=execute_actions)
        for var, value in prototype.items():
            if var not in self.model.state_machines:
                val = value(self) if callable(value) else value
                self.statevars[var] = val

        ### ADDED TO ENSURE CONSISTENCY WHEN APPLYING PROTOTYPES.
        ### TODO: revise prototype application to enhance efficiency
        if self._host and 'MASTER' in self._host:
            self._host['MASTER'].get_host().make_all_consistent()


[docs]    def apply_initial_prototype(self, name=None, prototype=None, execute_actions=False):
        """Apply prototype to a newly created agent. This function is called
        by `new_atom`in `MultiProcessManager` and redefined to do nothing in
        this class, in order to ensure that the prototype of a level is
        applied before creating entities for the sublevels.
        """
        if name is not None:
            # print('applying prototype', name, 'on', self)
            self.apply_prototype(name=name, execute_actions=execute_actions)
        elif prototype is not None:
            # print('applying custom prototype', prototype, 'on', self)
            self.apply_prototype(prototype=prototype,
                                 execute_actions=execute_actions)

# Methods useful to model designers
AbstractAgent.duration_in_current_state.__USER_METHOD__ = ['Durations']
AbstractAgent.update_time_to_exit.__USER_METHOD__ = ['Durations']
AbstractAgent.apply_prototype.__USER_METHOD__ =\
  ['Agent State and Variable Changes']
```

# EMULSION

Epidemiological Multi-Level Simulation Framework

### Navigation

- 1. Installation
- 2. Getting started with EMULSION
- 3. Modelling principles
- 4. Modelling language (basics)
- 5. Modelling language (advanced)
- 6. Feature examples
- 7. Information
- 8. License
- 9. High-level functions for model designers
- 10. emulsion package

### Related Topics

- Documentation overview
  - Module code

### Quick search

©2016, INRA and Univ. Lille.
|
Powered by Sphinx 1.8.5
& Alabaster 0.7.10
