# Supplementary material for: EMULSION: Transparent and flexible multiscale stochastic models in human, animal and plant epidemiology
Source: PLoS Comput Biol. 2019 Sep 13;15(9):e1007342. doi: 10.1371/journal.pcbi.1007342 (PMC6760811; doi:10.1371/journal.pcbi.1007342)
Supplement: S2 File — This zip file is a clone of EMULSION public Git repository. To install from this file rather than from PyPI, go to EMULSION documentation page: https://sourcesup.renater.fr/www/emulsion-public/pages/Install.html and follow Git-based installation instructions. (ZIP) [file pcbi.1007342.s010.zip › S2_file/doc/_modules/emulsion/agent/process.html]

emulsion.agent.process — EMULSION (Epidemiological Multi-Level Simulation framework)

# Source code for emulsion.agent.process

```
"""A Python implementation of the EMuLSion framework (Epidemiologic
MUlti-Level SImulatiONs).

Classes and functions for process management in MultiProcessCompartments.
"""

# [HEADER]


from   abc                       import abstractmethod
import numpy                     as np

from   emulsion.tools.misc       import rates_to_probabilities, aggregate_probabilities, count_population

[docs]class AbstractProcess(object):
    """An AbstractProcess is aimed at controlling a specific activity
    in a compartment, and is identified by its name.

    """

[docs]    def __init__(self, name):
        self.name = name

def __repr__(self):
        return 'Process "{}"'.format(self.name)

    __str__ = __repr__

[docs]    @abstractmethod
    def evolve(self):
        """Define the actions that the process must perform."""
        pass


[docs]class MethodProcess(AbstractProcess):
    """A MethodProcess is aimed at running a specific method (and
    possibly any function or even any callable object).

    """

[docs]    def __init__(self, name, method, lparams=[], dparams={}):
        super().__init__(name)
        self.method = method
        self.lparams = lparams
        self.dparams = dparams


[docs]    def evolve(self):
        """Define the actions that the process must perform. In a
        MethodProcess, those actions consist in running the method of
        the target compartment.

        """
        self.method(*self.lparams, **self.dparams)


[docs]class StateMachineProcess(AbstractProcess):
    """A StateMachineProcess is aimed at running a specific state machine
    within the agent (not within a grouping).

    """

[docs]    def __init__(self, name, agent, state_machine):
        super().__init__(name)
        self.agent = agent
        self.state_machine = state_machine


[docs]    def evolve(self):
        """Define the actions that the process must perform. In a
        StateMachineProcess, those actions consist in 1) executing the
        transitions of the state machine to change the agent's states
        and 2) computing productions and transmit them to the upper
        level of the agent.

        """
        self.evolve_transitions()
        self.agent.upper_level().new_agents += self.evolve_productions()


[docs]    def evolve_transitions(self):
        # retrieve the name of the statevar/attribute where the
        # current state is stored
        statevar = self.state_machine.machine_name
        # retrieve the value of the current state
        #current_state = self.get_information(statevar).name
        current_state = self.agent.statevars[statevar].name
        # perform actions associated to the current state
        self.agent.do_state_actions('on_stay', self.state_machine,
                                    current_state, population=1)
        # retrieve all possible transitions from this state
        transitions = self.agent.next_states_from(current_state,
                                                  self.state_machine)
        # skip this machine if no available transitions
        if transitions:
            states, flux, values, _, actions = zip(*transitions)
            total_value = sum(values)
            states = states + (current_state,)
            actions = actions + ([], )
            available_flux = set(flux)
            if 'amount' in available_flux or 'amount-all-but' in available_flux:
            # handle amounts
                # compute proper values (bounded by 0/1) and when
                # needed, invert 'amount-all-but' values
                values = [max(min(1-v, 1), 0) if f == 'amount-all-but'\
                            else max(min(v, 1), 0)
                          for (f, v) in zip(flux, values)]
                # recompute total value
                total_value = sum(values)
                # normalize to have probabilities
                if total_value == 0:
                    values = (0,)*len(values) + (1,)
                else:
                    values = tuple(v / total_value
                                   for v in values) + (1- total_value,)
            elif 'proba' in available_flux:
                # handle probabilities
                values = aggregate_probabilities(values,
                                                 self.agent.model.delta_t)
                values += (1 - sum(values),)
            else:
                # transform rates into probabilities
                values = rates_to_probabilities(total_value, values,
                                                delta_t=self.agent.model.delta_t)
            index = np.nonzero(np.random.multinomial(1, values))[0][0]
            next_state = states[index]
            next_action = actions[index]
            # condition on the index, so as to allow reflexive edges !
            if index != len(values)-1:
            # if next_state != current_state:
                self.agent.do_state_actions('on_exit', self.state_machine,
                                            current_state, population=1)
                # self.agent.set_information(statevar,
                #                            self.state_machine.states[next_state])
                self.agent.change_state(statevar,
                                        self.state_machine.states[next_state])
                # self.agent.do_edge_actions('on_cross', state_machine,
                #                      current_state, next_state, population=1)
                if next_action:
                    self.agent.do_edge_actions(actions=next_action, population=1)
                self.agent.do_state_actions('on_enter', self.state_machine,
                                            next_state, population=1)


[docs]    def evolve_productions(self):
        # init empty list for all changes to perform
        future = []
        # retrieve the name of the statevar/attribute where the
        # current state is stored
        statevar = self.state_machine.machine_name
        # retrieve the value of the current state
        current_state = self.agent.statevars[statevar].name
        # retrieve all possible productions from this state
        productions = self.agent.production_from(current_state,
                                                 self.state_machine)
        # skip this machine if no available transitions
        if productions:
            ### HERE WE ASSUME THAT AN AGENT CAN PRODUCE SEVERAL OTHER
            ### AGENTS SIMULTANEOUSLY (OTHERWISE USE CONDITIONS)
            ### REWRITE TRANSITIONS TO HAVE DISJOINT SUB-POPULATIONS
            for target_state, flux, values, ref_pop, proto in productions:
                pop_size = count_population(ref_pop)
                amount = self.agent._compute_production(values, flux, pop_size, self.agent.stochastic)
                if amount > 0:
                    future.append((target_state, amount, proto))
        return future
```

# EMULSION

Epidemiological Multi-Level Simulation Framework

### Navigation

- 1. Installation
- 2. Getting started with EMULSION
- 3. Modelling principles
- 4. Modelling language (basics)
- 5. Modelling language (advanced)
- 6. Feature examples
- 7. Information
- 8. License
- 9. High-level functions for model designers
- 10. emulsion package

### Related Topics

- Documentation overview
  - Module code

### Quick search

©2016, INRA and Univ. Lille.
|
Powered by Sphinx 1.8.5
& Alabaster 0.7.10
