# Supplementary material for: EMULSION: Transparent and flexible multiscale stochastic models in human, animal and plant epidemiology
Source: PLoS Comput Biol. 2019 Sep 13;15(9):e1007342. doi: 10.1371/journal.pcbi.1007342 (PMC6760811; doi:10.1371/journal.pcbi.1007342)
Supplement: S2 File — This zip file is a clone of EMULSION public Git repository. To install from this file rather than from PyPI, go to EMULSION documentation page: https://sourcesup.renater.fr/www/emulsion-public/pages/Install.html and follow Git-based installation instructions. (ZIP) [file pcbi.1007342.s010.zip › S2_file/doc/_modules/emulsion/agent/atoms.html]

emulsion.agent.atoms — EMULSION (Epidemiological Multi-Level Simulation framework)

# Source code for emulsion.agent.atoms

```
"""A Python implementation of the EMuLSion framework (Epidemiologic
MUlti-Level SImulatiONs).

Classes and functions for entities management.
"""

# [HEADER]


from   collections               import OrderedDict

from   emulsion.agent.core       import EmulsionAgent
from   emulsion.agent.process    import StateMachineProcess, MethodProcess

#          _                                           _
#     /\  | |                    /\                   | |
#    /  \ | |_ ___  _ __ ___    /  \   __ _  ___ _ __ | |_
#   / /\ \| __/ _ \| '_ ` _ \  / /\ \ / _` |/ _ \ '_ \| __|
#  / ____ \ || (_) | | | | | |/ ____ \ (_| |  __/ | | | |_
# /_/    \_\__\___/|_| |_| |_/_/    \_\__, |\___|_| |_|\__|
#                                      __/ |
#                                     |___/

[docs]class AtomAgent(EmulsionAgent):
    """The AtomAgent is aimed at representing an 'individual', i.e. the
    smallest organization level to be modeled as an entity in the
    simulation. An AtomAgent may be situated in several hosts, each one
    associated with a specific tuple of state variables.

    """

[docs]    def __init__(self, **others):
        super().__init__(**others)
        self.statevars.population = 1
        self.stochastic = True
        self._host = OrderedDict()
        if 'host' in others:
            self.add_host(others['host'])

def __len__(self):
        return 1

[docs]    def get_content(self):
        """Return the population (1) of the current unit.

        """
        return ('population', 1)


[docs]    def add_host(self, host):
        """Add the specified host to the current AtomAgent, associated
        with the specified key.

        """
        self._host[host.keys] = host
        self.simulation = host.simulation


[docs]    def remove_host(self, host, keys=None):
        """Remove the specified host from the current AtomAgent,
        associated with the specified key.

        """
        if keys is None:
            del self._host[host.keys]
        else:
            if keys in self._host:
                del self._host[keys]


[docs]    def get_host(self, key='MASTER'):
        """Retrieve the host of the current AtomAgent identified by the
        specific key.

        """
        return self._host[key]


[docs]    def clone(self, prototype=None, custom_prototype=None, **others):
        """Make a copy of the current compartment with the specified
        observable/value settings. If a prototype is provided, it is
        applied to the new atom.

        """
        new_atom = self.__class__.from_dict(self.statevars)
        new_atom.model = self.model
        new_atom.level = self.level
        new_atom.statevars.update(**others)
        if prototype is not None:
            new_atom.apply_prototype(name=prototype)
        elif custom_prototype is not None:
            new_atom.apply_prototype(prototype=custom_prototype)
        return new_atom

#  ______          _       _                     _
# |  ____|        | |     (_)               /\  | |
# | |____   _____ | |_   ___ _ __   __ _   /  \ | |_ ___  _ __ ___
# |  __\ \ / / _ \| \ \ / / | '_ \ / _` | / /\ \| __/ _ \| '_ ` _ \
# | |___\ V / (_) | |\ V /| | | | | (_| |/ ____ \ || (_) | | | | | |
# |______\_/ \___/|_| \_/ |_|_| |_|\__, /_/    \_\__\___/|_| |_| |_|
#                                   __/ |
#                                  |___/

[docs]class EvolvingAtom(AtomAgent):
    """An EvolvingAtom is able to change state according to its
    own statemachines.

    """

[docs]    def __init__(self, statemachines=[], **others):
        super().__init__(**others)
        self.processes = []
        self.statemachine_processes = {}
        self.method_processes = {}
        if statemachines:
            self.set_statemachines(statemachines)


[docs]    def set_statemachines(self, statemachines):
        """Define the state machines that this agent is able to execute."""
        self.statemachine_processes = {
            sm.machine_name: StateMachineProcess(sm.machine_name, self, sm)
            for sm in statemachines
        }


[docs]    def init_level_processes(self):
        """Initialize the level of the agent."""
        if self.level in self.model.processes:
            self.processes = self.model.processes[self.level]
            for process in self.processes:
                if process not in self.statemachine_processes:
                    self.add_method_process(process)


[docs]    def add_method_process(self, process_name, method=None):
        """Add a process based on a method name."""
        # print('process:', process_name)
        if method is None:
            method = getattr(self, process_name)
        self.method_processes[process_name] = MethodProcess(process_name, method)


[docs]    def get_machine(self, name):
        """Return the state machine with the specified name."""
        return self.statemachine_processes[name].state_machine


[docs]    def evolve(self, machine=None):
        super().evolve(machine=machine)
        self.evolve_states()


[docs]    def evolve_states(self, machine=None):
        """Change the state of the current unit according to the
        specified state machine name. If no special state machine is
        provided, executes all the machines.

        """
        # retrieve the iterable containing the processes to execute
        if machine is not None:
            self.statemachine_processes[machine].evolve()
        else:
            for process_name in self.processes:
                process = self.statemachine_processes[process_name]\
                            if process_name in self.statemachine_processes\
                            else self.method_processes[process_name]
                process.evolve()
```

# EMULSION

Epidemiological Multi-Level Simulation Framework

### Navigation

- 1. Installation
- 2. Getting started with EMULSION
- 3. Modelling principles
- 4. Modelling language (basics)
- 5. Modelling language (advanced)
- 6. Feature examples
- 7. Information
- 8. License
- 9. High-level functions for model designers
- 10. emulsion package

### Related Topics

- Documentation overview
  - Module code

### Quick search

©2016, INRA and Univ. Lille.
|
Powered by Sphinx 1.8.5
& Alabaster 0.7.10
