# Supplementary material for: EMULSION: Transparent and flexible multiscale stochastic models in human, animal and plant epidemiology
Source: PLoS Comput Biol. 2019 Sep 13;15(9):e1007342. doi: 10.1371/journal.pcbi.1007342 (PMC6760811; doi:10.1371/journal.pcbi.1007342)
Supplement: S2 File — This zip file is a clone of EMULSION public Git repository. To install from this file rather than from PyPI, go to EMULSION documentation page: https://sourcesup.renater.fr/www/emulsion-public/pages/Install.html and follow Git-based installation instructions. (ZIP) [file pcbi.1007342.s010.zip › S2_file/doc/_modules/emulsion/agent/managers/multi_process_manager.html]

emulsion.agent.managers.multi\_process\_manager — EMULSION (Epidemiological Multi-Level Simulation framework)

# Source code for emulsion.agent.managers.multi\_process\_manager

```
"""
.. module:: emulsion.agent.managers.functions

.. moduleauthor:: Sébastien Picault <sebastien.picault@inra.fr>

"""

# [HEADER]

from   collections               import OrderedDict

import numpy                     as np

from   emulsion.agent.exceptions import LevelException
from   emulsion.agent.views      import AdaptiveView
from   emulsion.tools.misc       import load_class, select_random,\
    add_all_test_properties, AGENTS


from   emulsion.agent.managers.group_manager  import  GroupManager
from   emulsion.agent.managers.abstract_process_manager  import  AbstractProcessManager


#  __  __       _ _   _ _____
# |  \/  |     | | | (_)  __ \
# | \  / |_   _| | |_ _| |__) | __ ___   ___ ___  ___ ___
# | |\/| | | | | | __| |  ___/ '__/ _ \ / __/ _ \/ __/ __|
# | |  | | |_| | | |_| | |   | | | (_) | (_|  __/\__ \__ \
# |_|  |_|\__,_|_|\__|_|_|   |_|  \___/ \___\___||___/___/
#  __  __
# |  \/  |
# | \  / | __ _ _ __   __ _  __ _  ___ _ __
# | |\/| |/ _` | '_ \ / _` |/ _` |/ _ \ '__|
# | |  | | (_| | | | | (_| | (_| |  __/ |
# |_|  |_|\__,_|_| |_|\__,_|\__, |\___|_|
#                            __/ |
#                           |___/

[docs]class MultiProcessManager(AbstractProcessManager):
    """A MultiProcessManager is aimed handling several independent
    StructuredViews at the same time, together with a
    SimpleView containing all the atom units. It can
    automatically build compartments for:
    - state machines associated with a specific state variable or attribute
    - specific state variables or attributes with a limited number of
    values, such as booleans or enumerations

    """

[docs]    def __init__(self, model=None, level=None, **others):
        view_class, options = load_class(
            **model.levels[level]['super']['master']
        )
        master = view_class(keys='MASTER', host=self, recursive=True, **options)
        super().__init__(model=model, master=master, level=level, **others)


[docs]    def apply_initial_conditions(self):
        """Initialize level with initial conditions specified in the model.

        As this agent is aimed at managing individuals in the
        sub-levels, only 'prototypes' are taken into account to
        initialize the sub-levels.

        """
        if self.level in self.model.initial_conditions:
            conds = self.model.initial_conditions[self.level]
            for protos, qty, probas in conds['prototypes']:
                proba_values = [self.get_model_value(p) for p in probas]
                total_value = sum(proba_values)
                assert(0 <= total_value <= 1)
                ## if sum of probas < 1 add complement (even if
                ## proba_values same size as protos) to ensure that
                ## e.g. [0.1, 0.1] with amount 100 gives in average
                ## [10, 10] individuals
                if total_value < 1:
                    proba_values += [1 - total_value]
                amount = int(self.get_model_value(qty))
                qty_by_proto = np.random.multinomial(amount, proba_values)
                # truncate qty_by_proto if size > nb of protos actually used
                qty_by_proto = qty_by_proto[:len(protos)]
                to_add = []
                for proto, nb in zip(protos, qty_by_proto):
                    to_add += [ self.new_atom(prototype=proto, execute_actions=True)
                                for _ in range(nb)
                    ]
                self.add_atoms(to_add, init=True)


[docs]    def add_host(self, host):
        """Add the specified host to the current Multiprocessmanager, associated
        with the specified key.

        """
        if self._host is None:
            self._host = OrderedDict()
        self._host[host.keys] = host
        self.simulation = host.simulation


[docs]    def add_new_population(self, process_name, population):
        """Create new atoms with the population information"""
        new_atoms = []
        for target_state, amount, proto in population:
            val = self.get_model_value(target_state)
            var = val.state_machine
            sublevel = self.get_default_sublevel()
            prototype = self.model.get_prototype(sublevel, proto)
            prototype[var.machine_name] = val
            to_add = [ self.new_atom(custom_prototype=prototype, execute_actions=True)
                       for _ in range(int(amount)) ]
            # print(var, '=', val)
            # for atom in to_add:
            #     atom.change_state(var, val)
            new_atoms += to_add
        self.add_atoms(new_atoms)


[docs]    def add_compart_process(self,
                            process_name,
                            key_variables,
                            compart_manager=(GroupManager, {}),
                            machine_name=None,
                            allowed_values=None,
                            compart_class=(AdaptiveView, {})):
        super().add_compart_process(process_name, key_variables,
                                    compart_manager=compart_manager,
                                    machine_name=machine_name,
                                    allowed_values=allowed_values,
                                    compart_class=compart_class)


[docs]    def select_atoms(self, variable=None, state=None, value=None, process=None):
        """Return a list of atoms selected by specific *value* or *state* of a
        *variable*.

        Parameters
        ----------
        variable: str
            the variable to be compared to the *value* - if ``None``,
            all atoms are selected.
        state: str
            a name of the state used for the selection (instead of *value*)
        value: object
            the value to use for selection - if ``None``, the value is
            replaced with the model state specified in parameter
            *state*
        process: str
            name of a process associated to a specific grouping based
            on the *variable* (to accelerate search)

        Returns
        -------
        list:
            the list of matching agents in the sublevel
        """
        if variable is None:
            return list(self['MASTER'])
        if state is not None:
            value = self.model.get_value(state)
        if process is not None:
            if (value,) in self[process]._content:
                return list(self[process][(value,)]._content)
        return [agent for agent in self['MASTER']
                if agent.get_information(variable) == value]


[docs]    def get_group_atoms(self, process_name, group_name):
        """Return all atoms which belong to the the specified group
        name (state names). (*group_name* may be a subset of the grouping key)

        """
        complete_key = set(self.get_model_value(state_name)
                           for state_name in group_name)
        groups = self[process_name]
        value = []
        for key, compart in groups._content.items():
            if complete_key <= set(key):
                value += compart.get_content()[AGENTS]
        return value


[docs]    def get_agent_class_for_sublevel(self, sublevel):
        """Return the agent class in charge of representing the specified
        sublevel.

        """
        if sublevel not in self.model.levels:
            raise LevelException('not found', sublevel)
        if 'contains' not in self.model.levels[self.level]:
            raise LevelException('not linked with %s' % (self.level), sublevel)
        cl, _ = self.model.get_agent_class_for_level(sublevel)
        return cl


[docs]    def get_default_sublevel(self):
        """Return by default the first sublevel contained in this level, if
        any. If this level contains no sublevels, raise a
        LevelException.

        """
        if 'contains' not in self.model.levels[self.level]:
            raise LevelException('not specified for atom creation', '')
        return self.model.levels[self.level]['contains'][0]


[docs]    def new_atom(self, sublevel=None, prototype=None, custom_prototype=None,
                 execute_actions=False, **args):
        """Instantiate a new atom for the specified sublevel, with the
        specified arguments. If the sublevel is not specified, the
        first one from the `contains` list is taken. If the name of a
        prototype is provided, it is applied to the new agent (using
        the `execute_actions` parameter).

        """
        # print('Entering new_atom for', self, 'with prototype', prototype)
        if sublevel is None:
            sublevel = self.get_default_sublevel()
        atom_class = self.get_agent_class_for_sublevel(sublevel)
        args.update(model=self.model, simu_id=self.statevars.simu_id,
                    level=sublevel, step=self.statevars.step)
        if prototype is not None:
            args.update(prototype=prototype, execute_actions=execute_actions)
        elif custom_prototype is not None:
            args.update(custom_prototype=custom_prototype, execute_actions=execute_actions)
        new_atom = atom_class(**args)
        # initialize MASTER host
        new_atom.add_host(self['MASTER'])
        add_all_test_properties(new_atom)
        new_atom.apply_initial_prototype(name=prototype, prototype=custom_prototype,
                                         execute_actions=execute_actions)
        return new_atom


[docs]    def add_atoms(self, atom_set, init=False, level=None):
        """Add the specified set of atoms to the current
        MultiProcessManager. Atoms are especially added
        automatically to each of the compartment managers.  If `init`
        is True, the compartment managers counts the initial value of
        the populations in each compartment.

        """
        self['MASTER'].add(atom_set)
        self.statevars.population = len(self['MASTER']._content)
        # update the model of atoms
        if level is None:
            if 'contains' in self.model.levels[self.level]:
                level = self.model.levels[self.level]['contains'][0]
        for atom in atom_set:
            atom.model = self.model
            atom.level = level
        # check if any initialization action has to be performed
        for machine in self.init_machines:
            agents_to_init = OrderedDict()
            for atom in atom_set:
                state = atom.statevars[machine]
                if state in self.model.init_actions[machine]:
                    if state not in agents_to_init:
                        agents_to_init[state] = []
                    agents_to_init[state].append(atom)
            for state, atoms in agents_to_init.items():
                for action in self.model.init_actions[machine][state]:
                    # print(f'Executing INIT action {action} for state {state.name}')
                    action.execute_action(self['MASTER'], agents=atoms)

        # add atoms to appropriate compartments and make them
        # consistent
        for name, comp in self._content.items():
            if name not in self.no_compart:
                default_key = tuple(None for _ in comp.keys)
                comp[default_key].add(atom_set)
                self.make_consistent(comp)
                if init:
                    comp.update_counts()


[docs]    def make_all_consistent(self):
        """Check all compartments to ensure their consistency"""
        for name, comp in self._content.items():
            if name not in self.no_compart:
                self.make_consistent(comp)
                comp.update_counts()


[docs]    def make_consistent(self, compartment):
        """Make the specified dict compartment check and handle the
        consistency of its own sub-compartments.

        """
        for comp in compartment:
            comp.check_consistency()
        compartment.handle_notifications()


[docs]    def remove(self, agents_or_population):
        assert(agents_or_population[0] == 'agents')
        self.remove_atoms(agents_or_population[1])


[docs]    def remove_atoms(self, atom_set):
        """Remove the specified atoms from the current
        MultiProcessManager. Atoms are removed from each of the
        compartment managers (including the 'MASTER' set).

        """
        for atom in list(atom_set):
            for host in list(atom._host.values()):
                host.remove([atom])
        self.statevars.population = len(self['MASTER']._content)


[docs]    def select_randomly(self, proba=0, amount=None, process=None):
        """Select randomly chosen atoms from this ProcessManager. `proba` can
        be either a probability or a dictionary. In that case, the
        `process` parameter indicates the name of the process grouping
        which drives the probabilities, and the keys must be those of
        the grouping. Selected atoms are removed and returned by the
        method.

        """
        if self.population <= 0:
            return []
        if process is None:
            if amount is None:
                amount = np.random.binomial(len(self['MASTER']), proba)
            selection = select_random(self['MASTER'], amount)
        else:
            selection = []
            if amount is None:
                for key, compart in self[process].items():
                    if key in proba:
                        selection += select_random(
                            compart, np.random.binomial(len(compart),
                                                        proba[key]))
            else:
                return []       #  inconsistent call
        return selection


[docs]    def remove_randomly(self, proba=0, amount=None, process=None):
        """Remove randomly chosen atoms from this ProcessManager. `proba` can
        be either a probability or a dictionary. In that case, the
        `process` parameter indicates the name of the process grouping
        which drives the probabilities, and the keys must be those of
        the grouping. Selected atoms are removed and returned by the
        method.

        """
        to_remove = self.select_randomly(amount=amount, proba=proba,
                                         process=process)
        self.remove_atoms(to_remove)
        # print(to_remove)
        return to_remove
```

# EMULSION

Epidemiological Multi-Level Simulation Framework

### Navigation

- 1. Installation
- 2. Getting started with EMULSION
- 3. Modelling principles
- 4. Modelling language (basics)
- 5. Modelling language (advanced)
- 6. Feature examples
- 7. Information
- 8. License
- 9. High-level functions for model designers
- 10. emulsion package

### Related Topics

- Documentation overview
  - Module code

### Quick search

©2016, INRA and Univ. Lille.
|
Powered by Sphinx 1.8.5
& Alabaster 0.7.10
