# Supplementary material for: EMULSION: Transparent and flexible multiscale stochastic models in human, animal and plant epidemiology
Source: PLoS Comput Biol. 2019 Sep 13;15(9):e1007342. doi: 10.1371/journal.pcbi.1007342 (PMC6760811; doi:10.1371/journal.pcbi.1007342)
Supplement: S2 File — This zip file is a clone of EMULSION public Git repository. To install from this file rather than from PyPI, go to EMULSION documentation page: https://sourcesup.renater.fr/www/emulsion-public/pages/Install.html and follow Git-based installation instructions. (ZIP) [file pcbi.1007342.s010.zip › S2_file/doc/_modules/emulsion/agent/managers/functions.html]

emulsion.agent.managers.functions — EMULSION (Epidemiological Multi-Level Simulation framework)

# Source code for emulsion.agent.managers.functions

```
"""
.. module:: emulsion.agent.managers.functions

.. moduleauthor:: Sébastien Picault <sebastien.picault@inra.fr>

"""

# [HEADER]

from   collections               import OrderedDict


#  ______                _   _
# |  ____|              | | (_)
# | |__ _   _ _ __   ___| |_ _  ___  _ __  ___
# |  __| | | | '_ \ / __| __| |/ _ \| '_ \/ __|
# | |  | |_| | | | | (__| |_| | (_) | | | \__ \
# |_|   \__,_|_| |_|\___|\__|_|\___/|_| |_|___/

def _group_by_populations(transitions):
    """`transitions` is a list of tuples (state, flux, value,
    cond_result, actions) where:
    - state is a possible state reachable from the current state flux
    - is either 'rate' or 'proba' or 'amount' or 'amount-all-but'
    - value is the corresponding rate or probability or amount
    - cond_result is a couple (either ('population', qty) or ('agents', list))
      describing who fulfills the condition to cross the transition
    - actions is the list of actions on cross
    """
    transitions_by_pop = OrderedDict()
    for state, flux, value, population, actions in transitions:
        desc, pop = population
        if desc == 'agents':
            pop = frozenset(pop)    # required to be hashable
        if (desc, pop) not in transitions_by_pop:
            transitions_by_pop[(desc, pop)] = []
        transitions_by_pop[(desc, pop)].append((state, flux, value, actions))
    # rewrite elements as a list
    return list(transitions_by_pop.items())

def _split_populations(list_of_items):
    """Transform a list of transitions into a dictionary based on the
    underlying populations.

    `list_of_items` is composed of pairs
    - the first element is a tuple ('agents', frozenset)
    - the second element is a list of tuples (state, flux, value,
      actions) associated with the population/agents

    Greedy algorithm: the first population of agents is compared with
    all others to check if any intersection exists. If so, both are
    replaced by their difference (if not empty) with the intersection,
    and the intersection is added to the list with a concatenation of
    the features attached to both populations.
    """
    # remove 'agents' keyword to facilitate set-like operations
    population_list = [(pop[1], attributes) for pop, attributes in list_of_items]
    i = 0
    while i < len(population_list) - 1:
        # select the first population in the list
        ref_pop, ref_attrib = population_list[i]
        j = i + 1
        intersects = False
        # check if any intersection with another population
        while j < len(population_list) and not intersects:
            # intersection found
            if population_list[j][0] & ref_pop:
                intersects = True
            else:
                j += 1
        if intersects:
            # retrieve features of the other population
            other_pop, other_attrib = population_list[j]
            # compute intersections and differences
            inter = ref_pop & other_pop
            new_ref =  ref_pop - inter
            new_other = other_pop - inter
            # if empty populations, remove them else update the populations
            if not new_other:
                population_list.pop(j)
            else:
                population_list[j] = (new_other, other_attrib)
            if not new_ref:
                population_list.pop(i)
            else:
                population_list[i] = (new_ref, ref_attrib)
            # add the intersection as a new population with a
            # concatenation of other properties
            population_list.append((inter, ref_attrib + other_attrib))
        else:
            # if no intersection was found, move to next item
            # (otherwise keep the same and iterate again on other
            # items)
            i += 1
    return [(('agents', list(agents)), attributes)
            for agents, attributes in population_list]

[docs]def group_and_split_populations(transitions):
    """Transform a list of transitions into a dictionary based on the
    underlying populations.

    `transitions` is a list of tuples (state, flux, value,
    cond_result, actions) where:
    - state is a possible state reachable from the current state flux
    - is either 'rate' or 'proba' or 'amount' or 'amount-all-but'
    - value is the corresponding rate or probability or amount
    - cond_result is a couple (either ('population', qty) or ('agents', list))
      describing who fulfills the condition to cross the transition
    - actions is the list of actions on cross

    The goal of this function is to restructure all those elements for
    disjoint sub-populations.

    Return a list of tuples:
      either (('population', qty), attributes)
      or     (('agents', list), attributes)
    where attributes is a list of tuples (state, flux, value, actions)

    """
    ### 1st step: gather elements
    # list_of_items is composed of pairs
    # - the first element is a tuple either ('population', qty) or
    #   ('agents', frozenset)
    # - the second element is a list of tuples (state, flux, value,
    #   actions) associated with the population/agents
    list_of_items = _group_by_populations(transitions)
    # if based on population (not on agents) then we're done (assuming
    # that all other values are based on populations)
    if list_of_items[0][0][0] == 'population':
        return list_of_items
    # otherwise we have true agents populations
    ### 2nd step: split sub-populations
    result =  _split_populations(list_of_items)
    #print(result)
    return result
```

# EMULSION

Epidemiological Multi-Level Simulation Framework

### Navigation

- 1. Installation
- 2. Getting started with EMULSION
- 3. Modelling principles
- 4. Modelling language (basics)
- 5. Modelling language (advanced)
- 6. Feature examples
- 7. Information
- 8. License
- 9. High-level functions for model designers
- 10. emulsion package

### Related Topics

- Documentation overview
  - Module code

### Quick search

©2016, INRA and Univ. Lille.
|
Powered by Sphinx 1.8.5
& Alabaster 0.7.10
