# Supplementary material for: EMULSION: Transparent and flexible multiscale stochastic models in human, animal and plant epidemiology
Source: PLoS Comput Biol. 2019 Sep 13;15(9):e1007342. doi: 10.1371/journal.pcbi.1007342 (PMC6760811; doi:10.1371/journal.pcbi.1007342)
Supplement: S2 File — This zip file is a clone of EMULSION public Git repository. To install from this file rather than from PyPI, go to EMULSION documentation page: https://sourcesup.renater.fr/www/emulsion-public/pages/Install.html and follow Git-based installation instructions. (ZIP) [file pcbi.1007342.s010.zip › S2_file/doc/_modules/emulsion/agent/managers/compart_process_manager.html]

emulsion.agent.managers.compart\_process\_manager — EMULSION (Epidemiological Multi-Level Simulation framework)

# Source code for emulsion.agent.managers.compart\_process\_manager

```
"""
.. module:: emulsion.agent.managers.functions

.. moduleauthor:: Sébastien Picault <sebastien.picault@inra.fr>

"""

# [HEADER]

import itertools                 as it
from   collections               import OrderedDict

import numpy                     as np
import pandas                    as pd

from   emulsion.agent.comparts   import Compartment

from   emulsion.agent.managers.group_manager  import  GroupManager
from   emulsion.agent.managers.abstract_process_manager  import  AbstractProcessManager
from   emulsion.agent.exceptions import StateVarNotFoundException

#   _____                                 _   _____
#  / ____|                               | | |  __ \
# | |     ___  _ __ ___  _ __   __ _ _ __| |_| |__) | __ ___   ___ ___  ___ ___
# | |    / _ \| '_ ` _ \| '_ \ / _` | '__| __|  ___/ '__/ _ \ / __/ _ \/ __/ __|
# | |___| (_) | | | | | | |_) | (_| | |  | |_| |   | | | (_) | (_|  __/\__ \__ \
#  \_____\___/|_| |_| |_| .__/ \__,_|_|   \__|_|   |_|  \___/ \___\___||___/___/
#                       | |
#                       |_|
#  __  __
# |  \/  |
# | \  / | __ _ _ __   __ _  __ _  ___ _ __
# | |\/| |/ _` | '_ \ / _` |/ _` |/ _ \ '__|
# | |  | | (_| | | | | (_| | (_| |  __/ |
# |_|  |_|\__,_|_| |_|\__,_|\__, |\___|_|
#                            __/ |
#                           |___/

[docs]class CompartProcessManager(AbstractProcessManager):
    """A CompartProcessManager is aimed handling several independent
    StructuredViews at the same time, for managing true compartments.
    It can automatically allocate compartments for state machines
    associated with a specific state variable or attribute.

    """

[docs]    def add_compart_process(self,
                            process_name,
                            key_variables,
                            compart_manager=(GroupManager, {}),
                            machine_name=None,
                            compart_class=(Compartment, {})):
        super().add_compart_process(process_name, key_variables,
                                    compart_manager=compart_manager,
                                    machine_name=machine_name,
                                    compart_class=compart_class)


[docs]    def add_host(self, host):
        """Add the specified host to the current Multiprocessmanager, associated
        with the specified key.

        """
        if self._host is None:
            self._host = OrderedDict()
        self._host[host.keys] = host
        self.simulation = host.simulation


[docs]    def apply_initial_conditions(self):
        """Initialize level with initial conditions specified in the model.

        As this agent is aimed at managing aggregated populations in
        the sub-levels, only 'populations' are taken into account to
        initialize the sub-levels.

        """
        # print(self.keys)
        if self.level in self.model.initial_conditions:
            conds = self.model.initial_conditions[self.level]
            total = self.get_model_value(conds['total']) if 'total' in conds else 0
            population_to_add = {}
            # print(conds)
            for proc, compart in self._content.items():
                if proc in self.no_compart:
                    continue
                ## assuming here that in compartment-based models all
                ## keys are based on enumerations

                ## build the combinations of all possible compartment
                ## keys (excluding autoremove states)
                combinations = list(it.product(*[[state
                                                  for state in self.model.state_machines[machine_name].states
                                                  if not state.autoremove]
                                                 for machine_name in compart.keys]))
                # population_to_add[proc] = { key: 0 for key in combinations }
                population_to_add[proc] = {}
                subtotal = 0
                # compute initial values for each group
                for state_names, qty in conds['populations']:
                    key = tuple(self.get_model_value(state_name) for state_name in state_names)
                    # if key in population_to_add[proc]:
                    if key in combinations:
                        amount = self.get_model_value(qty)
                        population_to_add[proc][key] = amount
                        subtotal += amount

                total = max(total, subtotal)
            self.complement_population(population_to_add, total=total)
            # print(population_to_add)
            self.add_population(population_to_add, init=True)
            for proc, compart in self._content.items():
                if proc not in self.no_compart:
                    compart.update_counts()


[docs]    def add_new_population(self, process_name, population):
        to_add = {}
        for target_state, amount, _ in population:
            key = (self.get_model_value(target_state),)
            if key not in to_add and amount > 0:
                to_add[key] = amount
            else:
                to_add[key] += amount
        self.add_population({process_name: to_add})


[docs]    def complement_population(self, population_to_change, total=0, remove=False):
        """Modifiy the population spec in a consistent way. Check is all
        populations in each process sum to the same total. If not, distribute
        the difference between total and sum at random between all available
        groups (FAIL if no available groups).

        """
        ## complement keys
        for proc, compart in self._content.items():
            if proc in self.no_compart:
                continue
            ## assuming here that in compartment-based models all
            ## keys are based on enumerations

            ## build the combinations of all possible compartment
            ## keys (excluding autoremove states)
            if remove:
                combinations = list(
                    it.product(*[[state
                                  for state in self.model.state_machines[machine_name].states
                                  if not state.autoremove]
                                 for machine_name in compart.keys]))
            else:
                combinations = list(
                    it.product(*[self.model.state_machines[machine_name].states.available
                                 for machine_name in compart.keys]))
            if proc not in population_to_change:
                population_to_change[proc] = {}
            population_to_change[proc].update({ key: 0
                                                for key in combinations
                                                if key not in population_to_change[proc]})
        ## compute total per process
        total_values = {}
        for proc in self._content:
            if proc in self.no_compart:
                continue
            total_values[proc] = sum(population_to_change[proc].values())\
                                 if proc in population_to_change else 0
        total = max(total, *total_values.values())

        # any process with less than total ?
        if any(value < total for value in total_values.values()):
            for proc, compart in self._content.items():
                if proc in self.no_compart:
                    continue
                subtotal = sum(population_to_change[proc].values())
                if subtotal < total:
                    difference = total - subtotal
                    available = [key for key in population_to_change[proc].keys()
                                 if population_to_change[proc][key] == 0]
                    if remove:
                        available = [key for key in available
                                     if key in compart._content and\
                                     compart[key].statevars.population > 0]
                    if not available:
                        print("WARNING !!! initial populations for process {} is"
                              " less than total population {} and there is no"
                              " empty compartment to redistribute the"
                              " difference".format(proc, subtotal))
                    else:
                        nb = len(available)
                        if remove:
                            pops = [compart[key].statevars.population for key in available]
                            probas = [pop / sum(pops) for pop in pops]
                        else:
                            probas = [1 / nb] * nb

                        amounts = np.random.multinomial(difference, probas)\
                                  if self.stochastic\
                                  else [difference * p for p in probas]

                        for key, amount in zip(available, amounts):
                            population_to_change[proc][key] = amount


[docs]    def add_population(self, population_spec, init=False):
        """Add the specified population specification to the current
        CompartProcessManager. `population_spec` is a dictionary with
        process names as keys, each one associated with a dictionary
        (tuple of statevars) -> population. If `init` is True, the compartment
        managers counts the initial value of the populations in each
        compartment.

        """
        self.complement_population(population_spec)
        # add populations to appropriate compartments for each process
        added = {}
        for process, spec in population_spec.items():
            # print(process, spec)
            if process not in self.no_compart:
                # retrieve the group manager associated to the process
                manager = self[process]
                # retrieve the machine name (always here in compartment models)
                machine_name = manager.state_machine.machine_name
                # locate the index of the statevar holding the state
                # of the state machine
                index = manager.keys.index(machine_name)\
                        if machine_name in manager.keys else None
                default_key = tuple(None for _ in manager.keys)
                added[process] = 0
                for key, qty in spec.items():
                    if key not in manager._content:
                        new_comp = manager[default_key].clone(population=qty)
                        if index is not None:
                            new_comp.statevars[machine_name] = key[index]
                        new_comp.keys = key
                        manager._content[key] = new_comp
                    else:
                        manager[key].add(qty)
                    added[process] += qty
        if init:
            manager.update_counts()
        # print(list(added.values()))
        nb = set(added.values())
        assert(len(nb) <= 1)
        if nb:
            self.statevars.population += nb.pop()


[docs]    def remove(self, agents_or_population):
        assert(agents_or_population[0] == 'population')
        self.remove_population(agents_or_population[1])


[docs]    def remove_population(self, population_spec):
        """Remove the specified population spec from the current
        CompartProcessManager.

        """
        self.complement_population(population_spec, remove=True)
        # print('>'*10)
        # print(population_spec)
        # print('<'*10)
        removed = {}
        for process, spec in population_spec.items():
            if process not in self.no_compart:
                # print(process, spec)
                manager = self[process]
                removed[process] = 0
                for key, qty in spec.items():
                    if key in manager._content:
                        manager[key].remove(qty)
                        removed[process] += qty
        nb = set(removed.values())
        assert(len(nb) <= 1)
        if nb:
            self.statevars.population -= nb.pop()


[docs]    def remove_randomly(self, proba=0, amount=None, process=None):
        """Remove random amounts of populations from this ProcessManager. If
        `amount` is not None, a multinomial sampling is performed for
        each process. Otherwise: `proba` can be either a probability
        or a dictionary. In that case, the `process` parameter
        indicates the name of the process grouping which drives the
        probabilities, and the keys must be those of the
        grouping. Selected quantities are removed and returned by the
        method.

        """
        to_remove = {}
        total = None
        if amount is not None:
            for name, proc in self._content.items():
                if name not in self.no_compart:
                    keys, probs = zip(*[(key, comp.population)
                                        for key, comp in proc._content.items()])
                    s = sum(probs)
                    probas = [p / s for p in probs]
                    amounts = np.random.multinomial(amount, probas)
                    to_remove[name] = dict(zip(keys, amounts))
                    # print(to_remove)
            self.remove_population(to_remove)
            return to_remove

        if process is not None:
            to_remove[process] = {}
            total = 0
            # print(self._content, process, name, self.no_compart)
            for key, comp in self[process].items():
                pop = comp.statevars.population
                if key in proba:
                    n = np.random.binomial(pop, proba[key])
                    to_remove[process][key] = n
                    total += n
        for name, proc in self._content.items():
            if name in self.no_compart or name == process:
                continue
            if total is None:
                to_remove[name] = {}
                total = 0
                for key, comp in proc._content.items():
                    pop = comp.statevars.population
                    n = np.random.binomial(pop, proba)
                    to_remove[name][key] = n
                    total += n
                continue
            keys, pops = zip(*[(key, comp.statevars.population)
                               for key, comp in self[name].items()])
            total_pop = sum(pops)
            probas = [ n / total_pop for n in pops]
            qties = np.random.multinomial(total, probas)
            to_remove[name] = dict(zip(keys, qties))
        # print(to_remove)
        self.remove_population(to_remove)
        return to_remove

@property
    def counts(self):
        """Return a pandas DataFrame containing counts of each process if existing.
        TODO: column steps need to be with one of process

        """
        res = {}
        for comp in self:
            try:
                res.update(comp.counts)
            except AttributeError:
                pass
            except Exception as exc:
                raise exc
        if not self.keep_history:
            res.update({
                'level': self.level,
                'agent_id': self.agid,
                # 'population': self.population}
            })
            if self.level in self.model.outputs and\
               'extra_vars' in self.model.outputs[self.level]:
                for name in self.model.outputs[self.level]['extra_vars']:
                    if name in self.model.parameters:
                        res[name] = self.get_model_value(name)
                    else:
                        try:
                            value = self.get_information(name)
                        except StateVarNotFoundException:
                            value = np.nan
                        res[name] = value
        return pd.DataFrame(res, index=[0])
```

# EMULSION

Epidemiological Multi-Level Simulation Framework

### Navigation

- 1. Installation
- 2. Getting started with EMULSION
- 3. Modelling principles
- 4. Modelling language (basics)
- 5. Modelling language (advanced)
- 6. Feature examples
- 7. Information
- 8. License
- 9. High-level functions for model designers
- 10. emulsion package

### Related Topics

- Documentation overview
  - Module code

### Quick search

©2016, INRA and Univ. Lille.
|
Powered by Sphinx 1.8.5
& Alabaster 0.7.10
