# Supplementary material for: EMULSION: Transparent and flexible multiscale stochastic models in human, animal and plant epidemiology
Source: PLoS Comput Biol. 2019 Sep 13;15(9):e1007342. doi: 10.1371/journal.pcbi.1007342 (PMC6760811; doi:10.1371/journal.pcbi.1007342)
Supplement: S2 File — This zip file is a clone of EMULSION public Git repository. To install from this file rather than from PyPI, go to EMULSION documentation page: https://sourcesup.renater.fr/www/emulsion-public/pages/Install.html and follow Git-based installation instructions. (ZIP) [file pcbi.1007342.s010.zip › S2_file/doc/_modules/emulsion/agent/managers/ibm_process_manager.html]

emulsion.agent.managers.ibm\_process\_manager — EMULSION (Epidemiological Multi-Level Simulation framework)

# Source code for emulsion.agent.managers.ibm\_process\_manager

```
"""
.. module:: emulsion.agent.managers.functions

.. moduleauthor:: Sébastien Picault <sebastien.picault@inra.fr>

"""

# [HEADER]

from   collections               import Counter

import numpy                     as np
import pandas                    as pd

from   emulsion.tools.misc       import select_random,\
    create_counter_getter, create_atoms_aggregator


from   emulsion.agent.managers.multi_process_manager  import  MultiProcessManager


#  _____ ____  __  __ _____
# |_   _|  _ \|  \/  |  __ \
#   | | | |_) | \  / | |__) | __ ___   ___ ___  ___ ___
#   | | |  _ <| |\/| |  ___/ '__/ _ \ / __/ _ \/ __/ __|
#  _| |_| |_) | |  | | |   | | | (_) | (_|  __/\__ \__ \
# |_____|____/|_|  |_|_|   |_|  \___/ \___\___||___/___/
#  __  __
# |  \/  |
# | \  / | __ _ _ __   __ _  __ _  ___ _ __
# | |\/| |/ _` | '_ \ / _` |/ _` |/ _ \ '__|
# | |  | | (_| | | | | (_| | (_| |  __/ |
# |_|  |_|\__,_|_| |_|\__,_|\__, |\___|_|
#                            __/ |
#                           |___/

[docs]class IBMProcessManager(MultiProcessManager):
    """An IBMProcessManager is a MultiProcessManager dedicated to the
    management of Individual-Based Models. This class is endowed with
    a `counters` attribute which is a dictionary {process -> counter of
    states in relation with the process}.

    """

[docs]    def __init__(self, **others):
        super().__init__(**others)
        self.statemachines = self.find_sublevel_statemachines()
        self.counters = {}
        self.autoremove_states = []
        self.new_agents = []
        self.counters = {}
        for machine in self.statemachines:
            self.counters[machine.machine_name] = Counter(
                atom.statevars[machine.machine_name].name
                for atom in self['MASTER']
            )
            self.autoremove_states += [(state.state_machine.machine_name, state)
                                       for state in machine.states
                                       if machine.get_property(state.name, 'autoremove')]
            for state in machine.states:
                if not state.autoremove:
                    self.create_count_properties_for_state(machine.machine_name,
                                                           state.name,
                                                           create_counter_getter,
                                                           create_atoms_aggregator)
        # print(self.autoremove_states)

        for atom in self['MASTER']:
            atom.set_statemachines(self.statemachines)
            atom.init_level_processes()


[docs]    def add_atoms(self, atom_set, init=False, **others):
        super().add_atoms(atom_set, init=init, **others)
        if not init:
            for atom in self['MASTER']:
                atom.set_statemachines(self.statemachines)
                atom.init_level_processes()


[docs]    def get_sublevels(self):
        """Return the list of sublevels contained in this level."""
        return self.model.levels[self.level]['contains']\
            if 'contains' in self.model.levels[self.level] else []


[docs]    def find_sublevel_statemachines(self):
        """Retrieve state machines used as processes by agents from the
        sub-level.

        """
        sublevels = self.get_sublevels()
        if not sublevels:
            return []
        return set([self.model.state_machines[process]
                    for sublevel in sublevels
                    for process in self.model.processes[sublevel]
                    if process in self.model.state_machines])


[docs]    def evolve(self, **others):
        """Make the agent evolve and update counts based on sub-level
        agents.

        """
        # prepair creation of new agents
        self.new_agents.clear()
        super().evolve(**others)
        ## handle autoremove states
        to_remove = []
        for machine_name, state in self.autoremove_states:
            to_remove += self.select_atoms(machine_name, value=state)
        self.remove_atoms(set(to_remove))
        self.add_new_population(None, self.new_agents)
        self.update_counts()


[docs]    def update_counts(self):
        """Update counters based on invdividual status."""
        for name, counter in self.counters.items():
            counter.clear()
            counter.update([atom.statevars[name].name
                            for atom in self['MASTER']])

# print(self.counters)
    @property
    def counts(self):
        """Return a pandas DataFrame containing counts of each process if
        existing.

        """
        res = {state.name: self.counters[state_machine.machine_name][state.name]
               for state_machine in self.statemachines
               for state in state_machine.states}
        res.update({'step': self.statevars.step,
                    'level': self.level,
                    'agent_id': self.agid,
                    # 'population': self.population}
        })
        if self.level in self.model.outputs and\
           'extra_vars' in self.model.outputs[self.level]:
            res.update({name: self.get_model_value(name)\
                        if name in self.model.parameters\
                        else self.get_information(name)
                        for name in self.model.outputs[self.level]['extra_vars']})
        return pd.DataFrame(res, index=[0])

[docs]    def remove_randomly(self, proba=0, statevar=None):
        """Remove randomly chosen atoms from this ProcessManager. `proba` can
        be either a probability or a dictionary. In that case, the
        `statevar` parameter indicates the name of the state variable
        which drives the probabilities, and the keys must be valid
        values for this state variable. Selected atoms are removed and
        returned by the method.

        """
        if statevar is None:
            to_remove = select_random(self['MASTER'],
                                      np.random.binomial(len(self['MASTER']),
                                                         proba))
        else:
            to_remove = []
            for atom in self['MASTER']:
                val = atom.get_information(statevar)
                if val in proba:
                    if np.random.binomial(1, proba[val]):
                        to_remove.append(atom)
        self.remove_atoms(to_remove)
        # print(to_remove)
        return to_remove
```

# EMULSION

Epidemiological Multi-Level Simulation Framework

### Navigation

- 1. Installation
- 2. Getting started with EMULSION
- 3. Modelling principles
- 4. Modelling language (basics)
- 5. Modelling language (advanced)
- 6. Feature examples
- 7. Information
- 8. License
- 9. High-level functions for model designers
- 10. emulsion package

### Related Topics

- Documentation overview
  - Module code

### Quick search

©2016, INRA and Univ. Lille.
|
Powered by Sphinx 1.8.5
& Alabaster 0.7.10
