# Supplementary material for: EMULSION: Transparent and flexible multiscale stochastic models in human, animal and plant epidemiology
Source: PLoS Comput Biol. 2019 Sep 13;15(9):e1007342. doi: 10.1371/journal.pcbi.1007342 (PMC6760811; doi:10.1371/journal.pcbi.1007342)
Supplement: S2 File — This zip file is a clone of EMULSION public Git repository. To install from this file rather than from PyPI, go to EMULSION documentation page: https://sourcesup.renater.fr/www/emulsion-public/pages/Install.html and follow Git-based installation instructions. (ZIP) [file pcbi.1007342.s010.zip › S2_file/doc/_modules/emulsion/agent/managers/abstract_process_manager.html]

emulsion.agent.managers.abstract\_process\_manager — EMULSION (Epidemiological Multi-Level Simulation framework)

# Source code for emulsion.agent.managers.abstract\_process\_manager

```
"""
.. module:: emulsion.agent.managers.functions

.. moduleauthor:: Sébastien Picault <sebastien.picault@inra.fr>

"""

# [HEADER]

import abc
import itertools                 as it

from   sortedcontainers          import SortedSet

import numpy                     as np
import pandas                    as pd

from   emulsion.agent.views      import StructuredView, AdaptiveView
from   emulsion.agent.process    import MethodProcess, StateMachineProcess
from   emulsion.agent.exceptions import StateVarNotFoundException
from   emulsion.tools.misc       import load_class, add_new_property,\
    create_population_getter, add_all_test_properties, create_aggregator,\
    create_group_aggregator


from   emulsion.agent.managers.group_manager  import  GroupManager


#           _         _                  _
#     /\   | |       | |                | |
#    /  \  | |__  ___| |_ _ __ __ _  ___| |_
#   / /\ \ | '_ \/ __| __| '__/ _` |/ __| __|
#  / ____ \| |_) \__ \ |_| | | (_| | (__| |_
# /_/    \_\_.__/|___/\__|_|  \__,_|\___|\__|
#  _____                             __  __
# |  __ \                           |  \/  |
# | |__) | __ ___   ___ ___  ___ ___| \  / | __ _ _ __   __ _  __ _  ___ _ __
# |  ___/ '__/ _ \ / __/ _ \/ __/ __| |\/| |/ _` | '_ \ / _` |/ _` |/ _ \ '__|
# | |   | | | (_) | (_|  __/\__ \__ \ |  | | (_| | | | | (_| | (_| |  __/ |
# |_|   |_|  \___/ \___\___||___/___/_|  |_|\__,_|_| |_|\__,_|\__, |\___|_|
#                                                              __/ |
#                                                             |___/

[docs]class AbstractProcessManager(StructuredView):
    """An AbstractProcessManager is aimed handling several independent
    StructuredViews at the same time, to represent several
    concerns. It can automatically build compartments for state
    machines associated with a specific state variable or attribute.

    """

[docs]    def __init__(self, model=None, master=None, level=None, stochastic=True,
                 keep_history=False, prototype=None, custom_prototype=None,
                 execute_actions=False, **others):
        super().__init__(recursive=False, **others)
        self.statevars.population = 0
        self.stochastic = stochastic
        self.model = model
        self.level = level
        self.keep_history = keep_history
        if master is not None:
            self._content['MASTER'] = master
            self.no_compart = SortedSet(['MASTER'])
        else:
            self.no_compart = SortedSet()
        # dynamically add property 'total_level' which returns the
        # total population in this level, and aggegate variables if any
        if self.level is not None:
            add_new_property(self, 'total_{}'.format(self.level),
                             lambda self: self.get_information('population'))
            if self.level in self.model.aggregate_vars:
                for (varname, sourcevar, operator) in self.model.aggregate_vars[self.level]:
                    add_new_property(self, varname, create_aggregator(sourcevar, operator))
        # machine names that potentially require state initialization
        self.init_machines = SortedSet()
        self.init_processes()
        ## apply prototype to self before creating sublevels
        ## done with super() method to control when it happens
        super().apply_initial_prototype(name=prototype, prototype=custom_prototype, execute_actions=execute_actions)
        self.apply_initial_conditions()
        self.initialize_level()


[docs]    def load_state_from_file(self, simu_id, filename):
        #TODO
        pass


[docs]    def save_state_to_file(self, simu_id, filename):
        #TODO
        pass


[docs]    def apply_initial_prototype(self, name=None, prototype=None, execute_actions=False):
        """This method inherited from `AbstractAgent` and called by `new_atom`
        is intentionnaly doing nothing in the current class to ensure
        that initial prototype of a `MultiProcessManager` is applied
        before creating the sublevels.

        """
        pass


[docs]    @abc.abstractmethod
    def apply_initial_conditions(self):
        """Apply initial conditions (if any) as defined in the model."""
        pass


[docs]    def initialize_level(self, **others):
        """User-defined operations when creating an instance of this
        level. These operations are performed *after* the application of
        initial conditions possibly defined in the corresponding model
        section."""
        pass


[docs]    def finalize_level(self, simulation=None, **others):
        """User-defined operations at the end of simulation for an instance of
        this level.

        """
        pass

# @abc.abstractmethod

[docs]    def add_new_population(self, process_name, population):
        pass


[docs]    def init_processes(self):
        """Init the processes that the ProcessManager will undergo during each
        time step, in order. Processes may be either 'method'
        processes (based on the execution of the specified method
        name), or 'group-based' processes (defined by the evolution of
        a grouping (aggregation or compartment), possibly endowed with
        a state machine), or even a 'state-machine-driven' process,
        based on the direct execution of a state machine within the
        ProcessManager.

        """
        if self.level in self.model.processes:
            for process in self.model.processes[self.level]:
                if self.level not in self.model.compartments or\
                   process not in self.model.compartments[self.level]:
                    if process in self.model.state_machines:
                        self.add_statemachine_process(process)
                    else:
                        self.add_method_process(process)
                else:
                    compart_properties = dict(
                        self.model.compartments[self.level][process])
                    for keyword in ['compart_manager', 'compart_class']:
                        if keyword in compart_properties:
                            class_desc = compart_properties[keyword]
                            # print(keyword, class_desc)
                            compart_properties[keyword] = load_class(**class_desc)
                    self.add_compart_process(process, **compart_properties)


[docs]    def evolve(self, **others):
        """Make the ProcessManager evolve, i.e. all the registered processes
        in order, starting with the evolution of the sublevels, and
        followed by the evolution inherited from superclasses.

        """
#        self['MASTER'].evolve()
        for process in self:
            process.evolve()
        for name, process in self._content.items():
            if name not in self.no_compart:
                self.add_new_population(name, process.new_population)
                process.update_counts()
        super().evolve(**others)


[docs]    def add_method_process(self, process_name, method=None):
        """Add a process based on a method name."""
        # print('process:', process_name)
        if method is None:
            method = getattr(self, process_name)
        self._content[process_name] = MethodProcess(process_name, method)
        self.no_compart.add(process_name)


[docs]    def add_statemachine_process(self, process_name):
        """Add a process based on the direct execution of a state machine."""
        self._content[process_name] = StateMachineProcess(
            process_name, self, self.model.state_machines[process_name]
        )
        self.no_compart.add(process_name)


[docs]    def add_compart_process(self,
                            process_name,
                            key_variables,
                            compart_manager=(GroupManager, {}),
                            machine_name=None,
                            allowed_values=None,
                            compart_class=(AdaptiveView, {})):
        """Add a process aimed at managing a 'Compartment Manager', i.e. an
        object aimed at managing a collection of compartments. This
        compartment manager is automatically initialized from the
        `compart_manager` class (which should be a subclass of
        StructuredView or GroupManager). The compartment manager may
        be associated with a specific state machine, and MUST BE
        identified by a tuple of state variables names. Additionally,
        since a first compartment is also instantiated, a specific
        class to do so can be also specified.

        """
        args = {'keys': tuple(key_variables), 'host': self,
                'keep_history': self.keep_history}
        compart_manager_cl, manager_options = compart_manager
        compart_class_cl, compart_options = compart_class
        if machine_name:
            machine = self.model.state_machines[machine_name]
            args['state_machine'] = machine
            # print(machine_name)
            if machine_name in self.model.init_actions:
                self.init_machines.add(machine_name)
            # dynamically add properties for accessing counts of each state
            for state in machine.states:
                if not state.autoremove:
                    self.create_count_properties_for_state(process_name,
                                                           state.name,
                                                           create_population_getter,
                                                           create_group_aggregator)
        if allowed_values:
            args['allowed_values'] = allowed_values
        args.update(manager_options)
        dict_comp = compart_manager_cl(**args)
        # update the model of the compartment manager
        dict_comp.model = self.model
        dict_comp.process_name = process_name
        init_key = tuple(None for _ in key_variables)
        dict_comp._content[init_key] = compart_class_cl(
            recursive=False,
            stochastic=self.stochastic,
            observables=key_variables,
            keys=init_key,
            values=init_key,
            host=dict_comp, **compart_options)
        # update the model of views
        dict_comp._content[init_key].model = self.model
        self._content[process_name] = dict_comp
        # dynamically add properties for accessing sub-groups when
        # groupings are based on several states from statemachines
        if len(key_variables) > 1 and\
           all(key in self.model.state_machines for key in key_variables):
            self.create_properties_for_groups(process_name, key_variables)
        # dynamically add properties for testing states in compart_class instances
        add_all_test_properties(dict_comp._content[init_key])


[docs]    def create_count_properties_for_state(self, process_or_machine_name, state_name,
                                          count_function, aggregation_function):
        """Dynamically add properties of the form ``total_S`` where S can be
        any state of the state machine. Counts are expected to be
        computed by the grouping associated with the specified
        process. The access to counts is defined by
        *count_function*. If aggregated variables such as ``aggvar``
        are defined, corresponding properties of the form
        ``aggvar_S``are also defined with the specified
        *aggregation_function*.

        """
        add_new_property(self, 'total_{}'.format(state_name),
                         count_function(process_or_machine_name, state_name))
        if self.level in self.model.aggregate_vars:
            for (varname, sourcevar, operator) in self.model.aggregate_vars[self.level]:
                add_new_property(self, '{}_{}'.format(varname, state_name),
                                 aggregation_function(sourcevar, operator,
                                                      process_or_machine_name, state_name))


[docs]    def create_properties_for_groups(self, process_name, key_variables):
        """Dynamically add properties of the form `total_S_T` where S, T are a
        valid key in grouping associated with the specified
        process_name.

        """
        # print(key_variables)
        combinations = list(it.product(*[[state.name
                                          for state in self.model.state_machines[machine_name].states
                                          if not state.autoremove]
                                         for machine_name in key_variables]))
        for group in combinations:
            add_new_property(self, 'total_{}'.format('_'.join(group)),
                             create_population_getter(process_name, group))
            if self.level in self.model.aggregate_vars:
                for (varname, sourcevar, operator) in self.model.aggregate_vars[self.level]:
                    add_new_property(self, '{}_{}'.format(varname, '_'.join(group)),
                                     create_group_aggregator(sourcevar, operator,
                                                             process_name, group))


[docs]    def get_group_population(self, process_name, group_name):
        """Return the size of the subgroup associated with the specified group
        name (state names).

        """
        complete_key = set(self.get_model_value(state_name)
                           for state_name in group_name)
        groups = self[process_name]
        value = 0
        for key, compart in groups._content.items():
            if complete_key <= set(key):
                value += compart.population
        return value

@property
    def counts(self):
        """Return a pandas DataFrame containing counts of each process if existing.
        TODO: column steps need to be with one of process

        """
        res = {}
        for comp in self:
            try:
                # TODO: BUG with StructuredViewWithCounts, step=0 always !
                if comp.__class__.__name__ != 'StructuredViewWithCounts':
                    comp.update_counts()
                    res.update(comp.counts)
            except AttributeError:
                pass
            except Exception as exc:
                raise exc
        if not self.keep_history:
            res.update({
                'level': self.level,
                'agent_id': self.agid,
                # 'population': self.population
            })
            if self.level in self.model.outputs and\
               'extra_vars' in self.model.outputs[self.level]:
                for name in self.model.outputs[self.level]['extra_vars']:
                    if name in self.model.parameters:
                        res[name] = self.get_model_value(name)
                    else:
                        try:
                            value = self.get_information(name)
                        except StateVarNotFoundException:
                            value = np.nan
                        res[name] = value
        df = pd.DataFrame(res, index=[0])
        ## DEBUG
        # print(df)
        return df

[docs]    @abc.abstractmethod
    def remove_randomly(self, proba=0):
        """Remove randomly chosen atoms or population from this
        ProcessManager.

        """
        pass


[docs]    @abc.abstractmethod
    def remove(self, agents_or_population):
        pass

@property
    def population(self):
        return self.statevars.population
```

# EMULSION

Epidemiological Multi-Level Simulation Framework

### Navigation

- 1. Installation
- 2. Getting started with EMULSION
- 3. Modelling principles
- 4. Modelling language (basics)
- 5. Modelling language (advanced)
- 6. Feature examples
- 7. Information
- 8. License
- 9. High-level functions for model designers
- 10. emulsion package

### Related Topics

- Documentation overview
  - Module code

### Quick search

©2016, INRA and Univ. Lille.
|
Powered by Sphinx 1.8.5
& Alabaster 0.7.10
