# Supplementary material for: EMULSION: Transparent and flexible multiscale stochastic models in human, animal and plant epidemiology
Source: PLoS Comput Biol. 2019 Sep 13;15(9):e1007342. doi: 10.1371/journal.pcbi.1007342 (PMC6760811; doi:10.1371/journal.pcbi.1007342)
Supplement: S2 File — This zip file is a clone of EMULSION public Git repository. To install from this file rather than from PyPI, go to EMULSION documentation page: https://sourcesup.renater.fr/www/emulsion-public/pages/Install.html and follow Git-based installation instructions. (ZIP) [file pcbi.1007342.s010.zip › S2_file/doc/_modules/emulsion/agent/managers/group_manager.html]

emulsion.agent.managers.group\_manager — EMULSION (Epidemiological Multi-Level Simulation framework)

# Source code for emulsion.agent.managers.group\_manager

```
"""
.. module:: emulsion.agent.managers.functions

.. moduleauthor:: Sébastien Picault <sebastien.picault@inra.fr>

"""

# [HEADER]

from   collections               import OrderedDict

from   sortedcontainers          import SortedDict

from   emulsion.agent.views      import StructuredView
from   emulsion.tools.misc       import count_population, rewrite_keys

from   emulsion.agent.managers.functions import group_and_split_populations

#   _____                       __  __
#  / ____|                     |  \/  |
# | |  __ _ __ ___  _   _ _ __ | \  / | __ _ _ __   __ _  __ _  ___ _ __
# | | |_ | '__/ _ \| | | | '_ \| |\/| |/ _` | '_ \ / _` |/ _` |/ _ \ '__|
# | |__| | | | (_) | |_| | |_) | |  | | (_| | | | | (_| | (_| |  __/ |
#  \_____|_|  \___/ \__,_| .__/|_|  |_|\__,_|_| |_|\__,_|\__, |\___|_|
#                        | |                              __/ |
#                        |_|                             |___/

[docs]class GroupManager(StructuredView):
    """An GroupManager is able to make its content
    evolve according to a specific state machine, the state of each
    subcompartment being stored in a specific state variable or
    attribute.

    """

[docs]    def __init__(self, state_machine=None, **others):
        """Create an GroupManager based on the
        specified state machine. The state of each subcompartment can
        be retrieved in the specified statevar name ('true' statevar
        or attribute)

        """
        ### WARNING: strange bug found sometimes when content={} not
        ### explicitly specified, another content (from another
        ### instance ???) may be used instead !!!!
        super().__init__(**others)
        self._content = SortedDict()
        self.state_machine = state_machine
        self.process_name = None
        self.init_counts()


[docs]    def init_counts(self, index=0):
        """Initialize the counts."""
        ## DEBUG
        # print('STEP', self.statevars.step, '\nInit counts in GM', self)
        self.counts = {}
        if self.state_machine is not None:
            self.counts = {state.name: [] if self.keep_history else 0
                           for state in self.state_machine.states}
            self.counts['step'] = [] if self.keep_history else 0
        else:
            super().init_counts()

## DEBUG
        # print(self.counts)

[docs]    def update_counts(self, index=0):
        """Update the number of atoms for each state of the state
        machine (TODO: for each value of the key[index] enum).

        """
        if self.state_machine is not None:
            total = {state.name: 0 for state in self.state_machine.states}
            ## DEBUG
            # print('\t', self.statevars.step, sep='')
            for (key, unit) in self._content.items():
                if key[index] is not None:
                    total[key[index].name] += unit.get_information('population')
                    # total[key[index].name] += unit.population
                    ## DEBUG
                    # print(key, unit.population, sep=' + ')
            # print()
            if self.keep_history:
                self.counts['step'].append(self.statevars.step)
                for state in self.state_machine.states:
                    self.counts[state.name].append(total[state.name])
            else:
                self.counts['step'] = self.statevars.step
                self.counts.update(total)
        else:
            super().update_counts()

## DEBUG
        # print('STEP', self.statevars.step, '\nUPDATE', self.counts)

[docs]    def apply_changes(self, transitions, productions):
        """Apply modifications to the compartments contained in the current
        StructuredView, according to `transitions` and
        `productions`. Dictionary `transitions` is keyed by a tuple of
        variables and associated with a list of dictionaries, either
        {'population': qty, 'actions': list} or {'agents': list,
        'actions': list}. List `productions` contains tuples (target,
        {'population': qty}, None) or (target, {'agents': list},
        prototype).

        """
        for source, evolutions in transitions.items():
            for target, population_or_agents in evolutions:
                target_comp = self.get_or_build(target, source=self[source])
                self._content[source].move_to(
                    target_comp,
                    state_machine=self.state_machine,
                    **population_or_agents)
        self.new_population = productions


[docs]    def evolve(self, machine=None):
        super().evolve(machine=machine)
        ## DEBUG
        # print(self.statevars.step, self.counts)
        self.evolve_states()
        ## DEBUG
        # print(self.statevars.step, self.counts)
        for key, comp in self._content.items():
            if comp.autoremove:
                agents_or_population = comp.get_content()
                if agents_or_population[0] == 'population':
                    agents_or_population = (agents_or_population[0],
                                            {self.process_name: {
                                                key: agents_or_population[1]
                                            }})
                self._host.remove(agents_or_population)
        self.update_counts()

## DEBUG
        # print(self.statevars.step, self.counts)

[docs]    def evolve_states(self, machine=None):
        """Ask each compartment to make its content evolve according
        to its current state and the specified state_machine.

        """
        self.new_population = None
        transitions = self._evolve_transitions(machine=machine)
        productions = self._evolve_productions(machine=machine)
        self.apply_changes(transitions, productions)

def _evolve_transitions(self, machine=None):
        # init empty dictionary for all changes to perform
        future = OrderedDict()
        # iterate over all compartments
        for name, compart in self._content.items():
            future[name] = []
            # compute the current population of each source compartment
            current_pop = compart.get_information('population')
            # no action if current pop <= 0
            if current_pop <= 0:
                continue
            # compute all possible transitions from the current state
            current_state = compart.get_information(
                self.state_machine.machine_name)
            # execute actions on stay for current state
            compart.do_state_actions('on_stay', self.state_machine,
                                     current_state.name,
                                     **dict([compart.get_content()]))
            # get the possible transitions from the current state
            # i.e. a list of tuples (state, flux, value, cond_result,
            # actions) where:
            # - state is a possible state reachable from the current state
            # - flux is either 'rate' or 'proba' or 'amount' or 'amount-all-but'
            # - value is the corresponding rate or probability or amount
            # - cond_result is a tuple (either ('population', qty) or
            # ('agents', list)) describing who fulfills the condition to cross
            # the transition
            # - actions is the list of actions on cross
            transitions = compart.next_states_from(current_state.name,
                                                   self.state_machine)
            # print('TRANSITIONS = ', name, '->', transitions)
            # nothing to do if no transitions
            if not transitions:
                continue

            ### REWRITE TRANSITIONS TO HAVE DISJOINT SUB-POPULATIONS
            transitions_by_pop = group_and_split_populations(transitions)
            # print(transitions_by_pop)
            for ref_pop, properties in transitions_by_pop:
                # retrieve the list of states, the list of flux, the
                # list of values, the list of populations affected by
                # each possible transition
                states, flux, values, actions = zip(*properties)
                # print(name, '->\n\t', states, values, [ag._agid
                #                                        for u in populations
                #                                        for ag in u['agents']])
                # add the current state to the possible destination states...
                states = states + (current_state.name,)
                # ... with no action
                actions = actions + ([], )
                #
                values, method = self._compute_values_for_unique_population(
                    values, flux, ref_pop, compart.stochastic)
                change_list = compart.next_states(states,
                                                  values,
                                                  [ref_pop],
                                                  actions, method=method)
                future[name] += rewrite_keys(name, name.index(current_state),
                                              change_list)
        # print('FUTURE:', future)
        return future


    def _evolve_productions(self, machine=None):
        # init empty list for all changes to perform
        future = []
        # iterate over all compartments
        for name, compart in self._content.items():
            # compute the current population of each source compartment
            current_pop = max(compart.get_information('population'), 0)
            # no action if "fake" compartment
            if set(name) == {None}:
                continue
            # if current pop == 0, productions are still possible (e.g. amounts)
            # compute all possible transitions from the current state
            current_state = compart.get_information(self.state_machine.machine_name)
            # get the possible productions from the current state
            # i.e. a list of tuples (state, flux, value, cond_result,
            # prototype) where:
            # - state is a possible state producible from the current state
            # - flux is either 'rate' or 'proba' or 'amount' or 'amount-all-but'
            # - value is the corresponding rate or probability or amount
            # - cond_result is a tuple (either ('population', qty) or
            # ('agents', list)) describing who fulfills the condition to cross
            # the transition
            # - prototype is the prototype for creating new agents
            productions = compart.production_from(current_state.name, self.state_machine)
            # print('PRODUCTIONS = ', productions)
            # nothing to do if no transitions
            if not productions:
                continue
            ### HERE WE ASSUME THAT AN AGENT CAN PRODUCE SEVERAL OTHER
            ### AGENTS SIMULTANEOUSLY (OTHERWISE USE CONDITIONS)
            ### REWRITE TRANSITIONS TO HAVE DISJOINT SUB-POPULATIONS
            for target_state, flux, values, ref_pop, proto in productions:
                pop_size = count_population(ref_pop)
                amount = self._compute_production(values, flux, pop_size, compart.stochastic)
                if amount > 0:
                    future.append((target_state, amount, proto))
        # print('FUTURE:', future)
        return future
```

# EMULSION

Epidemiological Multi-Level Simulation Framework

### Navigation

- 1. Installation
- 2. Getting started with EMULSION
- 3. Modelling principles
- 4. Modelling language (basics)
- 5. Modelling language (advanced)
- 6. Feature examples
- 7. Information
- 8. License
- 9. High-level functions for model designers
- 10. emulsion package

### Related Topics

- Documentation overview
  - Module code

### Quick search

©2016, INRA and Univ. Lille.
|
Powered by Sphinx 1.8.5
& Alabaster 0.7.10
