# Supplementary material for: EMULSION: Transparent and flexible multiscale stochastic models in human, animal and plant epidemiology
Source: PLoS Comput Biol. 2019 Sep 13;15(9):e1007342. doi: 10.1371/journal.pcbi.1007342 (PMC6760811; doi:10.1371/journal.pcbi.1007342)
Supplement: S2 File — This zip file is a clone of EMULSION public Git repository. To install from this file rather than from PyPI, go to EMULSION documentation page: https://sourcesup.renater.fr/www/emulsion-public/pages/Install.html and follow Git-based installation instructions. (ZIP) [file pcbi.1007342.s010.zip › S2_file/doc/_modules/emulsion/agent/managers/metapop_process_manager.html]

emulsion.agent.managers.metapop\_process\_manager — EMULSION (Epidemiological Multi-Level Simulation framework)

# Source code for emulsion.agent.managers.metapop\_process\_manager

```
"""
.. module:: emulsion.agent.managers.functions

.. moduleauthor:: Sébastien Picault <sebastien.picault@inra.fr>

"""

# [HEADER]

from   collections               import OrderedDict

import pandas                    as pd


from   emulsion.agent.managers.multi_process_manager  import  MultiProcessManager

#  __  __      _
# |  \/  |    | |
# | \  / | ___| |_ __ _ _ __   ___  _ __
# | |\/| |/ _ \ __/ _` | '_ \ / _ \| '_ \
# | |  | |  __/ || (_| | |_) | (_) | |_) |
# |_|  |_|\___|\__\__,_| .__/ \___/| .__/
#                      | |         | |
#                      |_|         |_|

[docs]class MetapopProcessManager(MultiProcessManager):
    """This class is in charge of handling multiple populations."""

[docs]    def get_populations(self):
        return OrderedDict(self['MASTER']._content)

@property
    def counts(self):
        """Return a pandas DataFrame containing counts of each process if
        existing.

        """
        result = None
        for population in self['MASTER']:
            res = {}
            for comp in population:
                try:
                    res.update(comp.counts)
                except AttributeError:
                    pass
                except Exception as exc:
                    raise exc
            if not self.keep_history:
                res.update({
                    'level': population.level,
                    'agent_id': population.agid,
                    # 'population': population.population,
                    'population_id': population.statevars.population_id})
                if population.level in population.model.outputs and\
                   'extra_vars' in population.model.outputs[population.level]:
                    res.update({name: population.get_model_value(name)\
                                if name in population.model.parameters\
                                else population.get_information(name)
                                for name in population.model.outputs[population.level]['extra_vars']})
            result = pd.DataFrame(res, index=[0]) if result is None\
                        else result.append(pd.DataFrame(res, index=[0]))
        return result
```

# EMULSION

Epidemiological Multi-Level Simulation Framework

### Navigation

- 1. Installation
- 2. Getting started with EMULSION
- 3. Modelling principles
- 4. Modelling language (basics)
- 5. Modelling language (advanced)
- 6. Feature examples
- 7. Information
- 8. License
- 9. High-level functions for model designers
- 10. emulsion package

### Related Topics

- Documentation overview
  - Module code

### Quick search

©2016, INRA and Univ. Lille.
|
Powered by Sphinx 1.8.5
& Alabaster 0.7.10
