# Supplementary material for: EMULSION: Transparent and flexible multiscale stochastic models in human, animal and plant epidemiology
Source: PLoS Comput Biol. 2019 Sep 13;15(9):e1007342. doi: 10.1371/journal.pcbi.1007342 (PMC6760811; doi:10.1371/journal.pcbi.1007342)
Supplement: S2 File — This zip file is a clone of EMULSION public Git repository. To install from this file rather than from PyPI, go to EMULSION documentation page: https://sourcesup.renater.fr/www/emulsion-public/pages/Install.html and follow Git-based installation instructions. (ZIP) [file pcbi.1007342.s010.zip › S2_file/doc/_modules/emulsion/agent/action.html]

emulsion.agent.action — EMULSION (Epidemiological Multi-Level Simulation framework)

# Source code for emulsion.agent.action

```
"""A Python implementation of the EMuLSion framework (Epidemiologic
MUlti-Level SImulatiONs).

Classes and functions for actions.
"""

# [HEADER]

from   abc                 import abstractmethod
import numpy               as     np

from   emulsion.tools.misc import retrieve_value, rates_to_probabilities


#  ______                    _   _
# |  ____|                  | | (_)
# | |__  __  _____ ___ _ __ | |_ _  ___  _ __  ___
# |  __| \ \/ / __/ _ \ '_ \| __| |/ _ \| '_ \/ __|
# | |____ >  < (_|  __/ |_) | |_| | (_) | | | \__ \
# |______/_/\_\___\___| .__/ \__|_|\___/|_| |_|___/
#                     | |
#                     |_|

[docs]class InvalidActionException(Exception):
    """Exception raised when a semantic error occurs in action definition.

    """

[docs]    def __init__(self, message):
        super().__init__()
        self.message = message

def __str__(self):
        return self.message

#   _____ _
#  / ____| |
# | |    | | __ _ ___ ___  ___  ___
# | |    | |/ _` / __/ __|/ _ \/ __|
# | |____| | (_| \__ \__ \  __/\__ \
#  \_____|_|\__,_|___/___/\___||___/

[docs]class AbstractAction(object):
    """AbstractActions are aimed at describing actions triggered by a
    state machine.

    """

[docs]    def __init__(self, state_machine=None, **_):
        self.state_machine = state_machine


[docs]    @abstractmethod
    def execute_action(self, unit, **others):
        """Execute the action on the specified unit."""
#        print(self, 'executed by', unit)
        pass


[docs]    @classmethod
    def build_action(cls, action_name, **others):
        """Return an instance of the appropriate Action subclass,
        depending on its name. The appropriate parameters for this
        action should be passed as a dictionary.

        """
        return ACTION_DICT[action_name](**others)

def __str__(self):
        return self.__class__.__name__


[docs]class ValueAction(AbstractAction):
    """ValueActions represent modifications of state variables or
    attributes.

    """

[docs]    def __init__(self, statevar_name=None, parameter=None, delta_t=1, **others):
        """Create a ValueAction aimed at modifying the specified
        statevar according to the parameter.

        """
        super().__init__(**others)
        self.statevar_name = statevar_name
        self.parameter = parameter
        self.delta_t = delta_t


[docs]class RecordChangeAction(ValueAction):
    """RecordChangeAction allows to record how many agents performed an
    action set. The corresponding value is added to a variable assumed
    to be defined at the upper level.

    """

[docs]    def __init__(self, parameter=None, model=None, **others):
        """Create a RecordChangeAction aimed at modifying the specified
        parameter, (assumed to be a statevar defined at the upper
        level).

        """
        super().__init__(**others)
        self.statevar_name = parameter

# print(self)

[docs]    def execute_action(self, unit, population=None, agents=None):
        """Execute the action on the specified unit, with the
        specified population size.

        """
        if population is None:
            population = len(agents)
        target_agent = unit.upper_level()
        value = target_agent.statevars[self.statevar_name]\
                if self.statevar_name in target_agent.statevars else 0
        target_agent.statevars[self.statevar_name] = value + population

def __str__(self):
        return super().__str__() + ' on {}'.format(self.statevar_name)
    __repr__ = __str__


[docs]class SetVarAction(ValueAction):
    """SetVarAction allows to set the variable of the agent.

    """

[docs]    def __init__(self, statevar_name=None, parameter=None, model=None, **others):
        """Create a SetVarAction aimed at modifying the specified statevar
        according to the parameter.

        """
        super().__init__(**others)
        if statevar_name in model.state_machines:
            raise InvalidActionException("Action set_var must not change values of state machines. Use action become instead.\n\tset_var: {} value: {}".format(statevar_name, parameter))
        self.statevar_name = statevar_name
        self.parameter = parameter

# print(self)

[docs]    def execute_action(self, unit, agents=None, **others):
        """Execute the action in the specified unit. If the `agents` parameter
        is specified (as a list), each agent of this list will execute
        the action. If changes of state variables in relation to a
        state machine occur, the corresponding actions (if any) are
        executed: on_exit from the current state, and on_enter for the
        new state.

        """
        if agents is None:
            agents = [unit]
        for agent in agents:
            # if the value which has to be affected to the variable is
            # already a statevar, retrieve its value from the agent
            if self.parameter in agent.statevars:
                value = agent.statevars[self.parameter]
            # otherwise get its value through the model
            else:
                value = agent.get_model_value(self.parameter)
            # agent.set_information(self.statevar_name, value)
            agent.statevars[self.statevar_name] = value

def __str__(self):
        return super().__str__() + ' {} <- {}'.format(self.statevar_name,
                                                      self.parameter)
    __repr__ = __str__


[docs]class RateAdditiveAction(ValueAction):
    """A RateChangeAction is aimed at increasing or decreasing a
    specific state variable or attribute, according to a specific rate
    (i.e. the actual increase or decrease is the product of the
    `parameter` attribute and a population size).

    """

[docs]    def __init__(self, sign=1, **others):
        super().__init__(**others)
        self.sign = sign


[docs]    def execute_action(self, unit, population=None, agents=None):
        """Execute the action on the specified unit, with the
        specified population size.

        """
        super().execute_action(unit)
        if population is None:
            population = len(agents)
        rate_value = self.state_machine.get_value(self.parameter)
        rate = retrieve_value(rate_value, unit)
        current_val = unit.get_information(self.statevar_name)
        new_val = current_val + self.sign*rate*population*self.delta_t
        # print('Executing', self.__class__.__name__, 'for', unit,
        #       self.statevar_name, current_val, '->', new_val,
        #       self.sign, rate, population)
        unit.set_information(self.statevar_name, new_val)

def __str__(self):
        return super().__str__() + ' ({}, {})'.format(self.statevar_name,
                                                      self.parameter)
    __repr__ = __str__


[docs]class RateDecreaseAction(RateAdditiveAction):
    """A RateDecreaseAction is aimed at decreasing a specific state
    variable or attribute, according to a specific rate (i.e. the
    actual decrease is the product of the `parameter` attribute and a
    population size).

    """

[docs]    def __init__(self, **others):
        super().__init__(sign=-1, **others)


[docs]class RateIncreaseAction(RateAdditiveAction):
    """A RateIncreaseAction is aimed at increasing a specific state
    variable or attribute, according to a specific rate (i.e. the
    actual increase is the product of the `parameter` attribute and a
    population size).

    """

[docs]    def __init__(self, **others):
        super().__init__(sign=1, **others)


[docs]class StochAdditiveAction(ValueAction):
    """A StochAdditiveAction is aimed at increasing or decreasing a
    specific state variable or attribute, according to a specific
    rate, using a *binomial sampling*.

    """

[docs]    def __init__(self, sign=1, **others):
        super().__init__(**others)
        self.sign = sign


[docs]    def execute_action(self, unit, population=None, agents=None):
        """Execute the action on the specified unit, with the
        specified population size.

        """
        super().execute_action(unit)
        if population is None:
            population = len(agents)
        rate_value = self.state_machine.get_value(self.parameter)
        rate = retrieve_value(rate_value, unit)
        # convert rate into a probability
        proba = rates_to_probabilities(rate, [rate], delta_t=self.delta_t)[0]
        current_val = unit.get_information(self.statevar_name)
        new_val = current_val + self.sign*np.random.binomial(population, proba)
        # print('Executing', self.__class__.__name__, 'for', unit,
        #       self.statevar_name, current_val, '->', new_val,
        #       self.sign, rate, population)
        unit.set_information(self.statevar_name, new_val)

def __str__(self):
        return super().__str__() + ' ({}, {})'.format(self.statevar_name,
                                                      self.parameter)
    __repr__ = __str__


[docs]class StochDecreaseAction(StochAdditiveAction):
    """A StochDecreaseAction is aimed at decreasing a specific state
    variable or attribute, according to a specific rate, using a
    *binomial sampling*.

    """

[docs]    def __init__(self, **others):
        super().__init__(sign=-1, **others)


[docs]class StochIncreaseAction(StochAdditiveAction):
    """A StochIncreaseAction is aimed at increasing a specific state
    variable or attribute, according to a specific rate, using a
    *binomial sampling*.

    """

[docs]    def __init__(self, **others):
        super().__init__(sign=1, **others)


[docs]class StringAction(AbstractAction):
    """A StringAction is based on the specification of a string
    parameter.

    """

[docs]    def __init__(self, parameter=None, l_params=[], d_params={}, **others):
        super().__init__(**others)
        self.parameter = parameter
        self.l_params = l_params
        self.d_params = d_params

def __str__(self):
        return super().__str__() + ' ({!s}, {}, {})'.format(self.parameter,
                                                            self.l_params,
                                                            self.d_params)
    __repr__ = __str__


[docs]class BecomeAction(AbstractAction):
    """A BecomeAction is aimed at making an agent change its state
    according to one ore more specified prototypes.

    """

[docs]    def __init__(self, prototypes=[], probas=None, model=None, **others):
        super().__init__(**others)
        self.prototype_names = prototypes if isinstance(prototypes, list)\
                               else [prototypes]
        if probas is None:
            probas = [1/len(self.prototype_names)] * len(self.prototype_names)
        else:
            probas = probas if isinstance(probas, list) else [probas]
        assert(0 <= len(self.prototype_names) - len(probas) <= 1)
        self.probas = [model.add_expression(pr) for pr in probas]

def __str__(self):
        return super().__str__() + ' ({!s}, {})'.format(self.prototype_names, self.probas)
    __repr__ = __str__

[docs]    def execute_action(self, unit, agents=None, **others):
        """Execute the action in the specified unit. If the `agents` parameter
        is specified (as a list), each agent of this list will execute
        the action. If changes of state variables in relation to a
        state machine occur, the corresponding actions (if any) are
        executed: on_exit from the current state, and on_enter for the
        new state.

        """
        if agents is None:
            agents = [unit]
        for agent in agents:
            protos = list(self.prototype_names)
            # compute actual values for probabilities
            proba_values = [agent.get_model_value(prob) for prob in self.probas]
            total = sum(proba_values)
            assert(0 <= total <= 1)
            if total < 1:
                # add complement
                proba_values.append(1 - total)
                # if N-1 probabilities were given for N prototypes, no
                # problem: the last prototype is getting the 1-total
                # value. Otherwise, this means that there is a
                # possibility that no individuals are produced => None
                if len(proba_values) > len(protos):
                    protos.append(None)
            prototype = np.random.choice(protos, p=proba_values)
            if prototype is not None:
                agent.apply_prototype(name=prototype, execute_actions=True)


[docs]class CloneAction(AbstractAction):
    """A CloneAction produces several copies of the agent with a given
    prototype.

    """

[docs]    def __init__(self, prototypes=[], amount=None, probas=None, model=None, **others):
        super().__init__(**others)
        self.prototype_names = prototypes if isinstance(prototypes, list)\
                               else [prototypes]
        amount= amount if amount is not None else 1
        if probas is None:
            probas = [1/len(self.prototype_names)] * len(self.prototype_names)
        else:
            probas = probas if isinstance(probas, list) else [probas]
        assert(0 <= len(self.prototype_names) - len(probas) <= 1)
        self.amount = model.add_expression(amount)
        self.probas = [model.add_expression(pr) for pr in probas]

def __str__(self):
        return super().__str__() + ' ({!s}, {}, {})'.format(self.prototype_names,
                                                            self.amount, self.probas)
    __repr__ = __str__

[docs]    def execute_action(self, unit, agents=None, **others):
        """Execute the action in the specified unit. If the `agents` parameter
        is specified (as a list), each agent of this list will execute
        the action. If changes of state variables in relation to a
        state machine occur, the corresponding actions (if any) are
        executed: on_exit from the current state, and on_enter for the
        new state.

        """
        if agents is None:
            agents = [unit]
        for agent in agents:
            protos = list(self.prototype_names)
            # compute actual values for probabilities
            proba_values = [agent.get_model_value(prob) for prob in self.probas]
            total = sum(proba_values)
            assert(0 <= total <= 1)
            if total < 1:
                # add complement
                proba_values.append(1 - total)
                # if N-1 probabilities were given for N prototypes, no
                # problem: the last prototype is getting the 1-total
                # value. Otherwise, this means that there is a
                # possibility that no individuals are produced => None
                if len(proba_values) > len(protos):
                    protos.append(None)
            quantities = np.random.multinomial(int(agent.get_model_value(self.amount)),
                                               proba_values)
            for prototype, quantity in zip(protos, quantities):
                if prototype is not None:
                    newborns = [agent.clone(prototype = prototype)
                                for _ in range(quantity)]
                    agent.upper_level().add_atoms(newborns)


[docs]class MessageAction(StringAction):
    """A MessageAction is aimed at making an agent print a given
    string. It requires a string message. This string can contain one
    reference to a variable or method of the agent, using Python's
    formatting syntax.

    For instance, 'My state is {.statevars.health_state}' will print
    the current health state of the agent.

    Output is formatted in four comma-separated fields: the simulation
    ID, the time step when the message was produced, the agent
    speaking, and the message itself.

    """

[docs]    def execute_action(self, unit, agents=None, **others):
        """Execute the action in the specified unit. If the `agents` parameter
        is specified (as a list), each agent of this list will execute
        the action.

        """
        if agents is None:
            agents = [unit]
        for agent in agents:
            message = self.parameter.format(agent)
            print("${},@{},{},{}".format(agent.get_information('simu_id'), agent.statevars.step, agent, message))


[docs]class LogVarsAction(StringAction):
    """A LogVarsAction is aimed at making an agent print a list of
    variables into a file. Output is formatted in several
    comma-separated fields: the simulation ID, the time step when the
    message was produced, the class and ID of agent speaking, and the
    values of variables (in the order they were defined in the
    action).

    """

[docs]    def execute_action(self, unit, agents=None, **others):
        """Execute the action in the specified unit. If the `agents` parameter
        is specified (as a list), each agent of this list will execute
        the action.

        """
        if agents is None:
            agents = [unit]
        for agent in agents:
            message = '{},{},{},{}'.format(agent.get_information('simu_id'), agent.statevars.step, agent.__class__.__name__, agent.agid)
            variables = ','.join(['{}={}'.format(varname, agent.get_information(varname))
                                  for varname in self.l_params])
            with open(agent.log_path(), 'a') as logfile:
                logfile.write('{},{}\n'.format(message, variables))


[docs]class MethodAction(AbstractAction):
    """A MethodAction is aimed at making an agent perform an action on
    a specific population. It requires a method name, and optionnally
    a list and a dictionary of parameters.

    """

[docs]    def __init__(self, method=None, l_params=[], d_params={}, **others):
        super().__init__(**others)
        self.method = method
        self.l_params = l_params
        self.d_params = d_params

def __str__(self):
        return super().__str__() + ' ({!s}, {}, {})'.format(self.method,
                                                            self.l_params,
                                                            self.d_params)
    __repr__ = __str__

[docs]    def execute_action(self, unit, agents=None, **others):
        """Execute the action using the specified unit. If the
        `agents` parameter is a list of units, each unit of this list
        will execute the action.

        """
        if agents is None:
            agents = [unit]
        for agent in agents:
            action = getattr(agent, self.method)
            l_params = [retrieve_value(self.state_machine.get_value(expr), agent)
                        for expr in self.l_params]
            ### introduced to pass internal information such as population
            d_params = others
            d_params.update({key: retrieve_value(self.state_machine.get_value(expr), agent)
                             for key, expr in self.d_params.items()})
            action(*l_params, **d_params)


[docs]class FunctionAction(MethodAction):
    """A FunctionAction is aimed at making an agent perform an action
    on a specific population. It requires a function, and optionnally
    a list and a dictionary of parameters. A FunctionAction runs
    faster than a MethodAction since it does not require to retrieve
    the method in each agent.

    """

[docs]    def __init__(self, function=None, **others):
        super().__init__(**others)
        self.function = function
        self.method = function.__name__


[docs]    def execute_action(self, unit, agents=None, **others):
        """Execute the action using the specified unit. If the
        `agents` parameter is a list of units, each unit of this list
        will execute the action.

        """
        if agents is None:
            agents = [unit]
        for agent in agents:
            l_params = [retrieve_value(self.state_machine.get_value(expr),
                                       agent)
                        for expr in self.l_params]
            ### introduced to pass internal information such as population
            d_params = others
            d_params.update({key:\
                             retrieve_value(self.state_machine.get_value(expr),
                                            agent)
                             for key, expr in self.d_params.items()})
            self.function(agent, *l_params, **d_params)

ACTION_DICT = {
    'increase': RateIncreaseAction,
    'decrease': RateDecreaseAction,
    'increase_stoch': StochIncreaseAction,
    'decrease_stoch': StochDecreaseAction,
    'message': MessageAction,
    'log_vars': LogVarsAction,
    'become': BecomeAction,
    'clone': CloneAction,
    'produce_offspring': CloneAction,
    'action': MethodAction,
    'duration': FunctionAction,
    'set_var': SetVarAction,
    'record_change': RecordChangeAction,
}
```

# EMULSION

Epidemiological Multi-Level Simulation Framework

### Navigation

- 1. Installation
- 2. Getting started with EMULSION
- 3. Modelling principles
- 4. Modelling language (basics)
- 5. Modelling language (advanced)
- 6. Feature examples
- 7. Information
- 8. License
- 9. High-level functions for model designers
- 10. emulsion package

### Related Topics

- Documentation overview
  - Module code

### Quick search

©2016, INRA and Univ. Lille.
|
Powered by Sphinx 1.8.5
& Alabaster 0.7.10
