# Supplementary material for: EMULSION: Transparent and flexible multiscale stochastic models in human, animal and plant epidemiology
Source: PLoS Comput Biol. 2019 Sep 13;15(9):e1007342. doi: 10.1371/journal.pcbi.1007342 (PMC6760811; doi:10.1371/journal.pcbi.1007342)
Supplement: S2 File — This zip file is a clone of EMULSION public Git repository. To install from this file rather than from PyPI, go to EMULSION documentation page: https://sourcesup.renater.fr/www/emulsion-public/pages/Install.html and follow Git-based installation instructions. (ZIP) [file pcbi.1007342.s010.zip › S2_file/doc/_modules/emulsion/agent/comparts.html]

emulsion.agent.comparts — EMULSION (Epidemiological Multi-Level Simulation framework)

# Source code for emulsion.agent.comparts

```
"""A Python implementation of the EMuLSion framework (Epidemiologic
MUlti-Level SImulatiONs).

Classes and functions for entities management.
"""

# [HEADER]


import numpy                     as np

from   emulsion.agent.core       import GroupAgent
from   emulsion.agent.exceptions import InvalidCompartmentOperation
from   emulsion.tools.misc       import POPULATION

[docs]class Compartment(GroupAgent):
    """An Compartment is a compartment which does not
    represent the underlying level but with aggregate information such
    as the total population ('individuals' are not represented).

    """

[docs]    def __init__(self, population=0, stochastic=True, **others):
        """Create an Compartment with an initial population."""
        super().__init__(**others)
        self.statevars.population = population
        self.stochastic = stochastic

def __len__(self):
        return self.statevars.population

[docs]    def get_content(self):
        """Return the population of the current unit.

        """
        return ('population', self.statevars.population)


[docs]    def add(self, population):
        """Add the specified population to the current population of
        the compartment.

        """
        self.statevars.population += population


[docs]    def remove(self, population):
        """Remove the specified population from the current population
        of the compartment (the population is kept positive).

        """
        self.statevars.population = max(0, self.statevars.population - population)

def _base_move(self, other_unit, population=0, **others):
        self.remove(population)
        other_unit.add(population)

[docs]    def move_to(self, other_unit, population, state_machine=None, **others):
        """Move the specified population from the current population
        of the compartment (the population is kept positive) to the
        other unit. If a state machine is provided, executes the
        corresponding actions when entering/exiting nodes and crossing
        edges if needed.

        """
        quantity = min(population, self.statevars.population)
        super().move_to(other_unit, population=quantity, state_machine=state_machine, **others)

@property
    def population(self):
        return self.statevars.population

[docs]    def clone(self, **others):
        """Make a copy of the current compartment with the specified
        observable/value settings. The new content is empty.

        """
        new_comp = self.__class__.from_dict(self.statevars)
        new_comp.statevars.population = 0
        new_comp.model = self.model
        new_comp.stochastic = self.stochastic
        new_comp._host = self._host
        new_comp.statevars.update(**others)
        return new_comp


[docs]    def next_states(self, states, values, populations, actions, method=None):
        """Compute the population moving from the current compartment to each
        of the destination states, handling the values according the
        the specified method. Values can be handled either as absolute
        amounts ('amount' method), as proportions ('rate', in a
        deterministic approach) or as probabilities ('proba', in a
        stochastic approach). Actions are to be performed when
        changing state. The actual population affected by the
        transitions is stored in the first element of the
        `populations` parameter, as a dictionary: {'population':
        number, 'actions': actions}. Several edges can lead to the
        same state.

        Return a list of tuples:
          (state, {'population': qty, 'actions:' list of actions})
        """
        current_pop = populations[0][POPULATION]
        if method == 'amount':
            # length of values is expected to be the number of output edges
            # retrieve the amount of population exiting
            total_value = sum(values)
            if total_value > current_pop:
                # restart with proportions instead
                return self.next_states(states,
                                        tuple(v / total_value for v in values) + (0,),
                                        populations, actions, method=None)
            evolution = values
        else:
            if self.stochastic:
                # length of values is expected to be the number of
                # output edges + 1 (last value = 1 - sum(values[:-1])
                evolution = np.random.multinomial(current_pop, values)
            else:
                # length of values is expected to be the number of
                # output edges
                evolution = [(np.exp(rate*self.model.delta_t) - 1) * current_pop
                             for rate in values]
        result =  [(self._host.state_machine.states[state],
                    {'population': qty, 'actions': act})
                   for state, qty, act in zip(states[:-1], evolution, actions[:-1])
                   if qty > 0]
        return result
```

# EMULSION

Epidemiological Multi-Level Simulation Framework

### Navigation

- 1. Installation
- 2. Getting started with EMULSION
- 3. Modelling principles
- 4. Modelling language (basics)
- 5. Modelling language (advanced)
- 6. Feature examples
- 7. Information
- 8. License
- 9. High-level functions for model designers
- 10. emulsion package

### Related Topics

- Documentation overview
  - Module code

### Quick search

©2016, INRA and Univ. Lille.
|
Powered by Sphinx 1.8.5
& Alabaster 0.7.10
