# Supplementary material for: EMULSION: Transparent and flexible multiscale stochastic models in human, animal and plant epidemiology
Source: PLoS Comput Biol. 2019 Sep 13;15(9):e1007342. doi: 10.1371/journal.pcbi.1007342 (PMC6760811; doi:10.1371/journal.pcbi.1007342)
Supplement: S2 File — This zip file is a clone of EMULSION public Git repository. To install from this file rather than from PyPI, go to EMULSION documentation page: https://sourcesup.renater.fr/www/emulsion-public/pages/Install.html and follow Git-based installation instructions. (ZIP) [file pcbi.1007342.s010.zip › S2_file/doc/_modules/emulsion/agent/views.html]

emulsion.agent.views — EMULSION (Epidemiological Multi-Level Simulation framework)

# Source code for emulsion.agent.views

```
"""A Python implementation of the EMuLSion framework (Epidemiologic
MUlti-Level SImulatiONs).

Classes and functions for entities management.
"""

# [HEADER]


from   collections               import OrderedDict
import numpy                     as np

from   sortedcontainers          import SortedSet

from   emulsion.agent.core       import Aggregation
from   emulsion.tools.misc       import select_random, AGENTS

#   _____ _                 _   __      ___
#  / ____(_)               | |  \ \    / (_)
# | (___  _ _ __ ___  _ __ | | __\ \  / / _  _____      __
#  \___ \| | '_ ` _ \| '_ \| |/ _ \ \/ / | |/ _ \ \ /\ / /
#  ____) | | | | | | | |_) | |  __/\  /  | |  __/\ V  V /
# |_____/|_|_| |_| |_| .__/|_|\___| \/   |_|\___| \_/\_/
#                    | |
#                    |_|

[docs]class SimpleView(Aggregation):
    """A SimpleView uses a set to store the underlying
    units. It is rather aimed at storing AtomAgents. All units are
    considered in the same state, thus conditions in the state
    machines are evaluated for the whole compartment, not for
    individuals.

    """

[docs]    def __init__(self, **others):
        super().__init__(**others)
        self._content = SortedSet()
        self.stochastic = True

def __iter__(self):
        return self._content.__iter__()

[docs]    def get_content(self):
        """Return the units contained in the current unit.

        """
        return ('agents', list(self._content))


[docs]    def add(self, population):
        """Add the specified population to the current compartment."""
        super().add(population)
        self._content |= SortedSet(population)


[docs]    def remove(self, population):
        """Remove the specified population from the current
        compartment.

        """
        super().remove(population)
        self._content -= SortedSet(population)


[docs]    def next_states(self, states, values, populations, actions, method=None):
        """Compute stochastically the population moving from the current
        compartment to each of the destination states, according to
        the values interpreted as probabilities. Actions are to be
        performed when changing state. The population affected by the
        transitions is stored in the first element of the
        `populations` parameter, as a tuple: ('agents',
        list_of_agents). Several edges can lead to the same state.

        Return a list of tuples:
          (state, {'agent': list of agents, 'actions': list of actions})
        """
        # retrieve the list of agents from the tuple at first position
        origin = populations[0][AGENTS]
        if method == 'amount':
            # length of values is expected to be the number of output edges
            total_value = sum(values)
            if total_value > len(origin):
                # restart with proportions instead
                return self.next_states(states,
                                        tuple(v / total_value for v in values) + (0,),
                                        populations, actions, method=None)
            evolution = [int(v) for v in values]
            # print(states, evolution)
        else:
            # values is expected to be the number of output edges + 1
            evolution = np.random.multinomial(len(origin), values)[:-1]

        ### now select agents corresponding to the amounts
        # init change list
        changes = []
        # track agents already selected
        modified = SortedSet()
        for state, qty, act in zip(states[:-1], evolution, actions[:-1]):
            if qty > 0:
                # pick up agents randomly (not already chosen) in the
                # origin group
                moving = select_random(origin, qty, exclude=modified)
                modified |= SortedSet(moving)
                # append this subgroup to the change list
                changes.append((self._host.state_machine.states[state],
                              {'agents': moving, 'actions': act}))
        return changes

#              _             _   _        __      ___
#     /\      | |           | | (_)       \ \    / (_)
#    /  \   __| | __ _ _ __ | |_ ___   ____\ \  / / _  _____      __
#   / /\ \ / _` |/ _` | '_ \| __| \ \ / / _ \ \/ / | |/ _ \ \ /\ / /
#  / ____ \ (_| | (_| | |_) | |_| |\ V /  __/\  /  | |  __/\ V  V /
# /_/    \_\__,_|\__,_| .__/ \__|_| \_/ \___| \/   |_|\___| \_/\_/
#                     | |
#                     |_|

[docs]class AdaptiveView(SimpleView):
    """An AdaptiveView is able to evaluate conditions on individuals
    (AtomAgents) for state transitions, and to detect changes in its
    content and automatically report it to the upper level. Each
    compartment is associated with a particular value of a specific
    state variable or attribute of the units in its content. At the
    end of the evolve step, the compartment checks the values of the
    units in its content and reports units where changes occured.

    """

[docs]    def __init__(self, observables=(None,), values=(None,), **others):
        """Specify the state variables or attributes associated with
        this compartment (``observables``) and the expected values.

        """
        super().__init__(**others)
        self.statevars.observables = observables
        for (observable, value) in zip(observables, values):
            self.statevars[observable] = value


[docs]    def evaluate_condition(self, condition):
        """Return the population of the compartment if the condition
        is fulfilled, 0 otherwise.

        """
        if not callable(condition):
            return self.get_content() if condition else ()
        result = list(filter(condition, self._content))
        return ('agents', result) if result else ()


[docs]    def next_states(self, states, values, populations, actions, method=None):
        """Compute stochastically the population moving from each population
        (tuples ('agents': list_of_agents)) in the `populations`
        parameter, to each of the possible destination states,
        according to each of the values interpreted as
        probabilities. Actions are to be performed when changing
        state. Several edges can lead to the same state.

        Return a list of tuples:
          (state, {'agent': list of agents, 'actions': list of actions})
        """
        if len(populations) == 1:
            if method == 'amount':
                # length of values is expected to be the number of output edges
                total_value = sum(values)
                if total_value > len(populations[0][AGENTS]):
                    # restart with proportions instead
                    return self.next_states(states,
                                            tuple(v / total_value
                                                  for v in values) + (0,),
                                            populations, actions, method=None)
                values = values + (total_value,)
            populations *= len(states)-1
        # init change list
        changes = []
        # track agents already selected
        modified = SortedSet()
        for state, value, population, act\
          in zip(states[:-1], values[:-1], populations, actions[:-1]):
            if method == 'amount':
                evolution = int(value)
            else:
                evolution = np.random.binomial(len(population[AGENTS]), value)
            if evolution > 0:
                ### now select agents corresponding to the amounts
                # pick up agents randomly (not already chosen) in the
                # origin group
                moving = select_random(population[AGENTS],
                                       evolution, exclude=modified)
                modified |= SortedSet(moving)
                # append this subgroup to the change list
                changes.append((self._host.state_machine.states[state],
                              {'agents': moving, 'actions': act}))
        return changes


[docs]    def evolve(self, machine=None):
        """After the ordinary ``evolve`` step, check units which have
        changed their value(s) of the compartment-specific
        observable(s). The corresponding units are reported to the
        upper level.

        """
        super().evolve(machine=machine)
        self.check_consistency()


[docs]    def check_consistency(self):
        """Check the value of the observables of the current
        compartment. Units which do not have the expected values are
        notified to the upper level.

        """
        # intruders = [unit for unit in self
        #              if any(unit.get_information(observable)\
        #                       != self.statevars[observable]
        #                     for observable in self.statevars.observables)]
        intruders = SortedSet()
        for unit in self:
            for observable in self.statevars.observables:
                if unit.get_information(observable) != self.statevars[observable]:
                    intruders.add(unit)
                    break
        if intruders:
            self._host.notify_changed_units(self, intruders)


[docs]    def clone(self, **others):
        """Make a copy of the current compartment with the specified
        observable/value settings. The new content is empty.

        """
        # new_comp = copy.copy(self)
        # new_comp._content = SortedSet()
        # new_comp.statevars = StateVarDict(self.statevars)
        new_comp = self.__class__.from_dict(self.statevars)
        new_comp.model = self.model
        new_comp._content = SortedSet()
        new_comp._host = self._host
        new_comp.statevars.update(**others)
        # new_comp.statevars.step = self.statevars.step
        new_comp.recursive = self.recursive
#        new_comp.set_information(observable, value)
        return new_comp

#   _____ _                   _                      ___      ___
#  / ____| |                 | |                    | \ \    / (_)
# | (___ | |_ _ __ _   _  ___| |_ _   _ _ __ ___  __| |\ \  / / _  _____      __
#  \___ \| __| '__| | | |/ __| __| | | | '__/ _ \/ _` | \ \/ / | |/ _ \ \ /\ / /
#  ____) | |_| |  | |_| | (__| |_| |_| | | |  __/ (_| |  \  /  | |  __/\ V  V /
# |_____/ \__|_|   \__,_|\___|\__|\__,_|_|  \___|\__,_|   \/   |_|\___| \_/\_/

[docs]class StructuredView(Aggregation):
    """A StructuredView uses ad dict list to store the underlying
    units. It is rather aimed at storing other compartiments.

    """

[docs]    def __init__(self, keep_history=True, **others):
        super().__init__(**others)
        self.keep_history = keep_history
        self.new_population = []
        self._content = OrderedDict()
        self._notifications = {}

def __iter__(self):
        return self._content.values().__iter__()

    def __getitem__(self, name):
        if name not in self._content: # DEBUG: should not happen !
            print(name)
        return self._content[name]

[docs]    def get_content(self):
        """Return the agents contained in the current view.

        """
        print('WARNING, should not enter here !!!')
        return ('agents', list(self._content.values()))


[docs]    def add(self, population):
        """Add the specified population to the current
        compartment. The population is expected to be a dictionary
        with names as keys and compartments as values.

        """
        for key, pop in population.items():
            if key in self._content:
                self._content[key].add(pop._content)
            else:
                self._content[key] = pop


[docs]    def remove(self, population):
        """Remove the specified population from the current
        compartment.  The population is expected to be a dictionary
        with names as keys and compartments as values.

        """
        for key, pop in population.items():
            if key in self._content:
                self._content[key].remove(pop._content)


[docs]    def notify_changed_units(self, source_compartment, units):
        """Receive a change notification concerning several units
        which cannot be stored anymore in the source compartment.

        """
        if source_compartment in self._notifications:
            self._notifications[source_compartment] |= units
        else:
            self._notifications[source_compartment] = SortedSet(units)


[docs]    def handle_notifications(self):
        """Handle all notifications received during the time step."""
        for source_compartment, units in self._notifications.items():
            source_observables = source_compartment.statevars.observables
            new_pop = {}
 #           pop_values = {}
            for unit in units:
                key = tuple(unit.get_information(k) for k in source_observables)
                if key not in new_pop:
                    new_pop[key] = [unit]
#                    pop_values[key] = unit.get_information(source_observable)
                else:
                    new_pop[key].append(unit)
            for key, unit in new_pop.items():
                # print(source_observables, key)
                new_comp = self.get_or_build(key, source=source_compartment)
#                print('Moving to:', new_comp, '\n\t', [u._agid for u in unit])
                source_compartment.move_to(new_comp, agents=unit)
        self._notifications.clear()


[docs]    def get_or_build(self, key, source=None):
        """Return the compartment with the specified key if any, or
        build one by cloning the source if not found.

        """
        if key not in self._content:
            key_infos = dict(zip(self.keys, key))
            # print('Adding new comp:', key, key_infos)
            new_compart = source.clone(host=self, **key_infos)
            new_compart.keys = key
            # if any key for compart is a state marked "autoremove",
            # then the whole compart is marked "autoremove"
            new_compart.autoremove = any(hasattr(value, 'state_machine') and\
                                  value.state_machine.get_property(value.name, 'autoremove')
                                  for value in key)
            # print(new_compart, new_compart.autoremove)
            self.add({key: new_compart})
        return self[key]


[docs]    def evolve(self, machine=None):
        super().evolve(machine=machine)
        self.handle_notifications()


[docs]class StructuredViewWithCounts(StructuredView):
    """Same as StructuredView, plus the capability of handling counts for
    key values.

    """

[docs]    def __init__(self, allowed_values=None, **others):
        super().__init__(**others)
        # if allowed_values:
        self.allowed_values = allowed_values
        self.init_counts()


[docs]    def init_counts(self, index=0):
        """Initialize the counts."""
        self.counts = {}
        if self.allowed_values:
            self.counts = {value: [] if self.keep_history else 0
                           for value in self.allowed_values}
        self.counts['step'] = [] if self.keep_history else 0


[docs]    def update_counts(self, index=0):
        """Update the counts.

        """
        if self.allowed_values:
            total = {value: 0 for value in self.allowed_values}
            for (key, unit) in self._content.items():
                if key[index] is not None and key[index] in self.allowed_values:
                    total[key[index]] += unit.get_information('population')
            if self.keep_history:
                self.counts['step'].append(self.statevars.step)
                for value in self.allowed_values:
                    self.counts[value].append(total[value])
            else:
                self.counts['step'] = self.statevars.step
                self.counts.update(total)

def __contains__(self, keys):
        return keys in self._content

[docs]    def evolve(self):
        super().evolve()
        self.update_counts()

#                _        _____ _                   _                      _
#     /\        | |      / ____| |                 | |                    | |
#    /  \  _   _| |_ ___| (___ | |_ _ __ _   _  ___| |_ _   _ _ __ ___  __| |
#   / /\ \| | | | __/ _ \\___ \| __| '__| | | |/ __| __| | | | '__/ _ \/ _` |
#  / ____ \ |_| | || (_) |___) | |_| |  | |_| | (__| |_| |_| | | |  __/ (_| |
# /_/    \_\__,_|\__\___/_____/ \__|_|   \__,_|\___|\__|\__,_|_|  \___|\__,_|
# __      ___
# \ \    / (_)
#  \ \  / / _  _____      __
#   \ \/ / | |/ _ \ \ /\ / /
#    \  /  | |  __/\ V  V /
#     \/   |_|\___| \_/\_/

[docs]class AutoStructuredView(StructuredView):
    """An AutoStructuredView stores agents in an OrderedDict, using a
    specific statevar as key.

    """

[docs]    def __init__(self, key_variable=None, **others):
        super().__init__(**others)
        self.key_variable = key_variable
        self.current_key = 0    # auto-increment ID if none specified


[docs]    def add(self, population):
        """Add the specified population (SortedSet or list) to the
        current view.

        """
        for agent in population:
            if self.key_variable not in agent.statevars:
                agent.statevars[self.key_variable] = self.current_key
                self.current_key += 1
            else:
                self.current_key = max(1 + agent.statevars[self.key_variable],
                                       self.current_key)
            self._content[agent.statevars[self.key_variable]] = agent


[docs]    def remove(self, population):
        """Remove the specified population from the current view. The
        population is expected to be a SortedSet or list.

        """
        for agent in population:
            key = agent.statevars[self.key_variable]
            if key in self._content:
                del self._content[key]
```

# EMULSION

Epidemiological Multi-Level Simulation Framework

### Navigation

- 1. Installation
- 2. Getting started with EMULSION
- 3. Modelling principles
- 4. Modelling language (basics)
- 5. Modelling language (advanced)
- 6. Feature examples
- 7. Information
- 8. License
- 9. High-level functions for model designers
- 10. emulsion package

### Related Topics

- Documentation overview
  - Module code

### Quick search

©2016, INRA and Univ. Lille.
|
Powered by Sphinx 1.8.5
& Alabaster 0.7.10
