# Supplementary material for: EMULSION: Transparent and flexible multiscale stochastic models in human, animal and plant epidemiology
Source: PLoS Comput Biol. 2019 Sep 13;15(9):e1007342. doi: 10.1371/journal.pcbi.1007342 (PMC6760811; doi:10.1371/journal.pcbi.1007342)
Supplement: S2 File — This zip file is a clone of EMULSION public Git repository. To install from this file rather than from PyPI, go to EMULSION documentation page: https://sourcesup.renater.fr/www/emulsion-public/pages/Install.html and follow Git-based installation instructions. (ZIP) [file pcbi.1007342.s010.zip › S2_file/doc/_modules/emulsion/model/functions.html]

emulsion.model.functions — EMULSION (Epidemiological Multi-Level Simulation framework)

# Source code for emulsion.model.functions

```
"""
.. module:: emulsion.model.functions

.. moduleauthor:: Sébastien Picault <sebastien.picault@inra.fr>

"""

# [HEADER]

from   sympy                   import sympify, lambdify, Symbol
# BEWARE: using lambdify, by default And and Or fall back to numpy's
# *binary* operators, so that more than 3 conditions linked by And or
# Or trigger a TypeError ('return arrays must be of ArrayType')
# see topic addressed there:
# https://stackoverflow.com/questions/42045906/typeerror-return-arrays-must-be-of-arraytype-using-lambdify-of-sympy-in-python
# To avoid problems, please use AND / OR (fully in UPPERCASE) instead
# of And / Or (Capitalized) in the conditions of Emulsion models

from   emulsion.tools.misc     import load_module


#  ______                _   _
# |  ____|              | | (_)
# | |__ _   _ _ __   ___| |_ _  ___  _ __  ___
# |  __| | | | '_ \ / __| __| |/ _ \| '_ \/ __|
# | |  | |_| | | | | (__| |_| | (_) | | | \__ \
# |_|   \__,_|_| |_|\___|\__|_|\___/|_| |_|___/

## Special strings for graphviz/dot formatting
#ACTION_SYMBOL = '&#9881;'
#ACTION_SYMBOL = '&#8623;'
CLOCK_SYMBOL = '&#9719;'
ACTION_SYMBOL = '&#9670;'
WHEN_SYMBOL = 'odot'
ESCAPE_SYMBOL = 'oinv'
COND_SYMBOL = 'tee'
CROSS_SYMBOL = 'diamond'
EDGE_KEYWORDS = ['rate', 'proba', 'amount', 'amount-all-but']


### INFORMATION TO ADD TO LEVEL DESCRIPTION WHEN USING aggregation_type
DEFAULT_LEVEL_INFO = {
    'IBM': {
        'level': {
            'class_name': 'IBMProcessManager',
            'module': 'emulsion.agent.managers',
            'master': {'class_name': 'SimpleView',
                       'module': 'emulsion.agent.views'}
        },
        'sublevels': {
            'class_name': 'EvolvingAtom',
            'module': 'emulsion.agent.atoms'
        }
    },
    'compartment': {
        'level': {
            'module': 'emulsion.agent.managers',
            'class_name': 'CompartProcessManager'
        }
    },
    'hybrid': {
        'level': {
            'module': 'emulsion.agent.managers',
            'class_name': 'MultiProcessManager',
            'master': {'module': 'emulsion.agent.views',
                       'class_name': 'SimpleView'}
        },
        'sublevels': {
            'module': 'emulsion.agent.atoms',
            'class_name': 'AtomAgent'
        }
    },
    'metapopulation': {
        'level': {
            'module': 'emulsion.agent.managers',
            'class_name': 'MetapopProcessManager',
            'master': {
                'module': 'emulsion.agent.views',
                'class_name': 'AutoStructuredView',
                'options': {'key_variable': 'population_id'}
            }
        },
    },
}

### INFORMATION TO ADD TO GROUPING DESCRIPTION WHEN USING aggregation_type
DEFAULT_GROUPING_INFO = {
    'hybrid': {
        'compart_manager': {
            'module': 'emulsion.agent.managers',
            'class_name': 'GroupManager'
        },
        'compart_class': {
            'module': 'emulsion.agent.views',
            'class_name': 'AdaptiveView'
        },
        'fallback_view': {
            'module': 'emulsion.agent.views',
            'class_name': 'StructuredViewWithCounts'
        },
    },
    'compartment': {
        'compart_manager': {
            'module': 'emulsion.agent.managers',
            'class_name': 'GroupManager'
        },
        'compart_class': {
            'module': 'emulsion.agent.comparts',
            'class_name': 'Compartment'
        }
    }
}

[docs]def make_function(expression,
                  dtype=float,
                  modules=['numpy', 'numpy.random', 'math', 'sympy']):
    """Transform the specified sympy expression into a function of an
    agent, which substitutes the specified symbols of the expression
    with an access to either attributes or state variables of the same
    name in the agent (through the ``get_information`` method) and
    returns a value of the specified dtype. The transformation uses
    the `lambdify` sympy function for better performances, with the
    specified modules.

    """
    symbs = tuple(expression.free_symbols)
    mods = [load_module(m) for m in modules]
    # print(expression, '->', symbs)

    lambdified = lambdify(symbs,
                          expression,
                          modules=mods)

    def _func(agent):
        vals = [float(agent.get_information(str(s))) for s in symbs]
        # try:
        return dtype(lambdified(*vals))
        # except TypeError as err:
        #     print(err.args, err.with_traceback, lambdified)
        #     print(expression)
        #     print(symbs)
        #     print(vals, type(vals))
        #     print(lambdified.__doc__)
        #     print(lambdified(7))
        #     import sys
        #     sys.exit(-1)
    return _func


[docs]def make_when_condition(expression,
                        dtype=bool,
                        modules=['numpy', 'numpy.random', 'math', 'sympy']):
    """Transform the specified sympy `expression` into a function of an
    agent, which substitutes the specified symbol of the expression
    with an access to the simulation calendar. The transformation uses
    the `lambdify` sympy function for better performances, with the
    specified modules.

    """
    ## General idea: expression should be a boolean test for a
    ## property in the agent -> simulation -> calendar,
    ## e.g. expressions such as 'breeding_period' or 'Not(vacation)'
    ## call a function associated with 'breeding_period' or 'vacation'
    ## strings in the calendar. The function are applied to simulation
    ## step and generated on the basis of the points or intervals
    ## defined in the 'calendar' section of the model. This implies
    ## that all agents must have access to the whole simulation (or at
    ## least to the calendar). This also means that an actual calendar
    ## is a subclass of a generic calendar, generated automatically to
    ## be endowed with those properties.
    symbs = tuple(expression.free_symbols)
    mods = [load_module(m) for m in modules]
    lambdified = lambdify(symbs,
                          expression,
                          modules=mods)
    def _func(agent):
        vals = [agent.evaluate_event(str(s)) for s in symbs]
        return dtype(lambdified(*vals))
    return _func


[docs]def make_duration_condition(model, machine_name):
    """Build a duration condition associated to the specified state
    machine and add it to the model. A condition duration, which is
    intended to specify when an agent is allowed to leave the current
    state of the state machine, is of the form 'step >=
    _time_to_exit_MACHINE_NAME', each of those variables being stored
    in the state variables.

    """
    # build the name of the state variable from the name of the state machine
    var_name = '_time_to_exit_{}'.format(machine_name)
    # add the association variable name -> Symbol to the namespace of the model
    model._namespace[var_name] = Symbol(var_name)
    # attach a description
    model.statevars[var_name] = {
        'desc': 'time step before which this agent is not allowed to exit current state of state machine {}'.format(machine_name)
    }
    # return the expression corresponding to the duration condition
    return 'GreaterThan(step, {})'.format(var_name)


[docs]def make_duration_init_action(agent, duration, machine_name=None, **_):
    """Action that initializes the 'time to exit' for the state of the
    specified state_machine.

    """
    # the action to execute when entering a state associated with a duration
    agent.update_time_to_exit(machine_name, duration)

# DEPRECATED in >0.9.5
# def make_TTL_increase_action(agent, machine_name=None, **_):
#     """Action that increases the time spent by the agent in the
#     current state of the specified state_machine.

#     """

#     agent.increase_time_spent(machine_name)
```

# EMULSION

Epidemiological Multi-Level Simulation Framework

### Navigation

- 1. Installation
- 2. Getting started with EMULSION
- 3. Modelling principles
- 4. Modelling language (basics)
- 5. Modelling language (advanced)
- 6. Feature examples
- 7. Information
- 8. License
- 9. High-level functions for model designers
- 10. emulsion package

### Related Topics

- Documentation overview
  - Module code

### Quick search

©2016, INRA and Univ. Lille.
|
Powered by Sphinx 1.8.5
& Alabaster 0.7.10
