# Supplementary material for: EMULSION: Transparent and flexible multiscale stochastic models in human, animal and plant epidemiology
Source: PLoS Comput Biol. 2019 Sep 13;15(9):e1007342. doi: 10.1371/journal.pcbi.1007342 (PMC6760811; doi:10.1371/journal.pcbi.1007342)
Supplement: S2 File — This zip file is a clone of EMULSION public Git repository. To install from this file rather than from PyPI, go to EMULSION documentation page: https://sourcesup.renater.fr/www/emulsion-public/pages/Install.html and follow Git-based installation instructions. (ZIP) [file pcbi.1007342.s010.zip › S2_file/doc/_modules/emulsion/model/exceptions.html]

emulsion.model.exceptions — EMULSION (Epidemiological Multi-Level Simulation framework)

# Source code for emulsion.model.exceptions

```
"""
.. module:: emulsion.model.exceptions

.. moduleauthor:: Sébastien Picault <sebastien.picault@inra.fr>

"""

# [HEADER]


#  ______                    _   _
# |  ____|                  | | (_)
# | |__  __  _____ ___ _ __ | |_ _  ___  _ __  ___
# |  __| \ \/ / __/ _ \ '_ \| __| |/ _ \| '_ \/ __|
# | |____ >  < (_|  __/ |_) | |_| | (_) | | | \__ \
# |______/_/\_\___\___| .__/ \__|_|\___/|_| |_|___/
#                     | |
#                     |_|

[docs]class SemanticException(Exception):
    """Exception raised when a semantic error occurs during model parsing.

    """

[docs]    def __init__(self, message):
        super().__init__()
        self.message = message

def __str__(self):
        return self.message
```

# EMULSION

Epidemiological Multi-Level Simulation Framework

### Navigation

- 1. Installation
- 2. Getting started with EMULSION
- 3. Modelling principles
- 4. Modelling language (basics)
- 5. Modelling language (advanced)
- 6. Feature examples
- 7. Information
- 8. License
- 9. High-level functions for model designers
- 10. emulsion package

### Related Topics

- Documentation overview
  - Module code

### Quick search

©2016, INRA and Univ. Lille.
|
Powered by Sphinx 1.8.5
& Alabaster 0.7.10
