# Supplementary material for: EMULSION: Transparent and flexible multiscale stochastic models in human, animal and plant epidemiology
Source: PLoS Comput Biol. 2019 Sep 13;15(9):e1007342. doi: 10.1371/journal.pcbi.1007342 (PMC6760811; doi:10.1371/journal.pcbi.1007342)
Supplement: S2 File — This zip file is a clone of EMULSION public Git repository. To install from this file rather than from PyPI, go to EMULSION documentation page: https://sourcesup.renater.fr/www/emulsion-public/pages/Install.html and follow Git-based installation instructions. (ZIP) [file pcbi.1007342.s010.zip › S2_file/doc/_modules/emulsion/model/state_machines.html]

emulsion.model.state\_machines — EMULSION (Epidemiological Multi-Level Simulation framework)

# Source code for emulsion.model.state\_machines

```
"""
.. module:: emulsion.model.state_machines

.. moduleauthor:: Sébastien Picault <sebastien.picault@inra.fr>

"""

# [HEADER]


from   functools               import partial

import numpy                   as     np
from   sympy                   import sympify

from   sortedcontainers        import SortedSet

import emulsion.tools.graph    as     enx
from   emulsion.agent.action   import AbstractAction
from   emulsion.tools.state    import StateVarDict, EmulsionEnum

from   emulsion.model.functions import ACTION_SYMBOL, WHEN_SYMBOL, ESCAPE_SYMBOL,\
    COND_SYMBOL, CROSS_SYMBOL, EDGE_KEYWORDS, CLOCK_SYMBOL,\
    make_when_condition, make_duration_condition, make_duration_init_action

from   emulsion.model.exceptions     import SemanticException


#   _____ _        _       __  __            _     _
#  / ____| |      | |     |  \/  |          | |   (_)
# | (___ | |_ __ _| |_ ___| \  / | __ _  ___| |__  _ _ __   ___
#  \___ \| __/ _` | __/ _ \ |\/| |/ _` |/ __| '_ \| | '_ \ / _ \
#  ____) | || (_| | ||  __/ |  | | (_| | (__| | | | | | | |  __/
# |_____/ \__\__,_|\__\___|_|  |_|\__,_|\___|_| |_|_|_| |_|\___|

[docs]class StateMachine(object):
    """Class in charge of the description of biological or economical
    processes, modeled as Finite State Machines. The formalism
    implemented here is based on UML state machine diagrams, with
    adaptations to biology.

    """

[docs]    def __init__(self, machine_name, description, model):
        """Build a State Machine within the specified model, based on
        the specified description (dictionary).

        """
        self.model = model
        self.machine_name = machine_name
        self.parse(description)

def _reset_all(self):
        self._statedesc = {}
        self._description = {}
        self.states = None
        self.graph = enx.MultiDiGraph()
        self.stateprops = StateVarDict()
        self.state_actions = {}
#        self.edge_actions = {}

[docs]    def parse(self, description):
        """Build the State Machine from the specified dictionary
        (expected to come from a YAML configuration file).

        """
        self._reset_all()
        # keep an exhaustive description
        self._description = description
        # build the enumeration of the states
        self.build_states()
        # build the graph based on the states and the transitions between them
        self.build_graph()
        # build actions associated with the state machine (states or edges)
        self.build_actions()


[docs]    def get_property(self, state_name, property_name):
        """Return the property associated to the specified state."""
        if state_name not in self.stateprops or\
           property_name not in self.stateprops[state_name]:
            return self.graph.node[state_name][property_name]\
                if property_name in self.graph.node[state_name]\
                   else None
        return self.stateprops[state_name][property_name]


[docs]    def build_states(self):
        """Parse the description of the state machine and extract the existing
        states. States are described as list items, endowed with
        key-value properties. Only one state per list item is allowed
        (to ensure that states are always stored in the same order in
        all executions).

        """
        states = []
        default_state = None
        # retrieve information for each state
        for statedict in self._description['states']:
            for name, value in statedict.items():
                states.append(name)
                # provide a default fillcolor
                if 'fillcolor' not in value:
                    value['fillcolor'] = 'lightgray'
                # if properties are provided, add the corresponding
                # expression to the model
                if 'properties' not in value:
                    value['properties'] = {}
                # store special property: "autoremove: yes"
                value['properties']['autoremove'] = value['autoremove']\
                                                    if 'autoremove' in value else False
                # store special property: "default: yes"
                # if several states are marked "default", take the first one
                value['properties']['default'] = False
                if ('default' in value) and (value['default']) and (default_state is None):
                    value['properties']['default'] = True
                    default_state = name
                self.stateprops[name] = {k: self.model.add_expression(v)
                                         for k, v in value['properties'].items()}
                # store special properties: "next: aState" and
                # "previous: aState" which define
                # successor/predecessor when using "next_state" and
                # "previous_state" in prototype definition (otherwise,
                # next and previous are defined as non-autoremove
                # states after and before in the list)
                if 'next' in value:
                    value['properties']['next'] = value['next']
                    self.stateprops[name]['next'] = value['next']
                if 'previous' in value:
                    value['properties']['previous'] = value['previous']
                    self.stateprops[name]['previous'] = value['previous']
                # store other information
                self._statedesc[name] = value
                # and retrieve available actions if any
                for keyword in ['on_enter', 'on_stay', 'on_exit']:
                    if keyword in value:
                        self._add_state_actions(name, keyword, value[keyword])
        # build the enumeration of the states
        self.states = EmulsionEnum(self.machine_name.capitalize(),
                                   states, module=__name__)
        # link the states to their state machine
        self.states.state_machine = self
        # define the default value for "autoremove" at the enumeration level
        self.states.autoremove = False

        for state in self.states:
            # check that state names are unique
            if state.name in self.model.states:
                other_machine = self.model.states[state.name].__class__.__name__
                raise SemanticException(
                    'Conflict: State %s found in statemachines %s and %s' %
                    (state.name, other_machine, state.__class__.__name__))
            # check that state names are not parameters
            if state.name in self.model.parameters:
                raise SemanticException(
                    'Conflict: State %s of statemachines %s found in parameters'
                    % (state.name, state.__class__.__name__))
            # associate the state with the state name in the model
            self.model.states[state.name] = state
            # update the autoremove value if the state is defined as such
            if self.stateprops[state.name]['autoremove']:
                state.autoremove = True
            # set next and previous to the state itself (to ensure
            # that all states have a predecessor/successor)
            state.predecessor = state
            state.successor = state

        # handle default states
        self.states.is_default = False
        # if a default state is defined, store it and limit the list
        # of "available" states
        if default_state is not None:
            self.states[default_state].is_default = True
            self.states.default = self.states[default_state]
            self.states.available = (self.states.default,)
        else:
        # otherwise the list of available states is simply those which
        # are not autoremove
            self.states.default = None
            self.states.available = tuple(s for s in self.states if not s.autoremove)
        # print(self.states, self.states.default, self.states.available)

        # define "successor" and "predecessor" properties
        # start from the list of non-autoremove states
        usable = [s for s in self.states if not s.autoremove]
        for state in usable:
            # if the state has a "predecessor" state, use it
            if 'previous' in self.stateprops[state.name]:
                state.predecessor = self.states[self.stateprops[state.name]['previous']]
            else:
                # otherwise use the previous state in the list, the
                # first one being its own previous state
                state.predecessor = usable[max(0, usable.index(state) - 1)]
            # if the state has a "successor" state, use it
            if 'next' in self.stateprops[state.name]:
                state.successor = self.states[self.stateprops[state.name]['next']]
            else:
                # otherwise use the next state in the list, the
                # last one being its own next state
                state.successor = usable[min(len(usable)-1, usable.index(state) + 1)]

        # define function used from model to provide a random state
        self.model._values['_random_' + self.machine_name] = self.get_random_state
        # define function used from model to provide the default state
        # if any, otherwise a random state among non-autoremove ones
        if self.states.default:
            self.model._values['_default_' + self.machine_name] = self.get_default_state
        else:
            self.model._values['_default_' + self.machine_name] = self.get_random_state


[docs]    def get_random_state(self, caller=None):
        """Return a random state for this state machine."""
        return np.random.choice([state for state in self.states if not state.autoremove])


[docs]    def get_default_state(self, caller=None):
        """Return the default state for this state machine."""
        return self.states.default

@property
    def state_colors(self):
        """Return a dictionary of state names associated with fill colors."""
        return {state.name: self._statedesc[state.name]['fillcolor']
                for state in self.states
                if not state.autoremove}

[docs]    def build_graph(self):
        """Parse the description of the state machine and extract the
        graph of the transitions between the states. Since a
        MultiDiGraph is used, each pair of nodes can be bound by
        several transitions if needed (beware the associated
        semantics).

        Example of YAML specification:
        ------------------------------
        transitions:
          - {from: S, to: I-, proba: p, cond: not_vaccinated}
          - {from: I-, to: S, proba: m}
          - {from: I-, to: I+m, proba: 'q*plp'}
          - {from: I-, to: I+, proba: 'q*(1-plp)'}

        """
        # add a node for each state
        for state in self.states:
            name = state.name
            self._statedesc[name]['tooltip'] = self.describe_state(name)
            self.graph.add_node(name, **self._statedesc[name])
        # build edges between states according to specified transitions
        if 'transitions' in self._description:
            self._parse_edges(self._description['transitions'],
                              type_id=enx.EdgeTypes.TRANSITION)
        if 'productions' in self._description:
            self._parse_edges(self._description['productions'],
                              type_id=enx.EdgeTypes.PRODUCTION)

def _parse_edges(self, edges, type_id=enx.EdgeTypes.TRANSITION):
        """Parse the description of edges, with the difference
        transitions/productions

        """
        for edge in edges:
            from_ = edge['from']
            to_ = edge['to']
            others = {k: v for (k, v) in edge.items()
                      if k != 'from' and k != 'to'}
            for kwd in EDGE_KEYWORDS:
                if kwd in others:
                    # parm = pretty(sympify(others[kwd], locals=self.model._namespace))
                    parm = others[kwd]
                    label = '{}: {}'.format(kwd, parm)
            # label = ', '.join([pretty(sympify(x, locals=self.model._namespace))
            #                    for x in others.values()])
                    if str(parm) in self.model.parameters:
                        others['labeltooltip'] = self.model.describe_parameter(parm)
                    else:
                        others['labeltooltip'] = label
            # others['labeltooltip'] = ', '.join([self.model.describe_parameter(x)
            #                                     for x in others.values()
            #                                     if str(x) in self.model.parameters])
            # handle conditions if any on the edge
            cond, escape = None, False
            if 'cond' in others:
                cond = others['cond']
                others['truecond'] = others['cond']
            if ('escape' in others) and (type_id == enx.EdgeTypes.TRANSITION):
                cond = others['escape']
                escape = True
            if cond is not None:
                ### WARNING the operation below is not completely
                ### safe... it is done to replace conditions of the form
                ### 'x == y' by 'Eq(x, y)', but it is a simple
                ### substitution instead of parsing the syntax
                ### tree... Thus it is *highly* recommended to express
                ### conditions directly with Eq(x, y)
                if '==' in str(cond):
                    cond = 'Eq({})'.format(','.join(cond.split('==')))
                    # others['label'] = ', '.join(others.values())
            # if duration specified for this state, handle it as an
            # additional condition
            if ('duration' in self._statedesc[from_]) and (type_id == enx.EdgeTypes.TRANSITION):
                duration_cond = make_duration_condition(self.model, self.machine_name)
                if cond is None:
                    cond = duration_cond
                elif escape:
                    cond = 'AND(Not({}),{})'.format(duration_cond, cond)
                else:
                    cond = 'AND({},{})'.format(duration_cond, cond)
                    # print(cond)
                others['cond'] = cond
            if cond is not None:
                ## DEBUG:                print(cond, self.model._namespace)
                self.model.conditions[cond] = sympify(cond,
                                                      locals=self.model._namespace)
            # handle 'when' clause if any on the edge
            self._parse_when(others)
            # handle 'duration', 'escape' and 'condition' clauses if
            # any on the edge
            if type_id == enx.EdgeTypes.TRANSITION:
                self._parse_conditions_durations(from_, others)
            # parse actions on cross if any
            if ('on_cross' in others) and (type_id == enx.EdgeTypes.TRANSITION):
                l_actions = self._parse_action_list(others['on_cross'])
                others['actions'] = l_actions
            others['label'] = label
            others['type_id'] = type_id
            self.graph.add_edge(from_, to_, **others)
            # register rate/proba/amount expressions in the model
            for keyword in EDGE_KEYWORDS:
                if keyword in others:
                    self.model.add_expression(others[keyword])


    def _parse_when(self, edge_desc):
        """Parse the edge description in search for a 'when'
        clause. This special condition is aimed at globally assessing
        a time period within the whole simulation.

        """
        if 'when' in edge_desc:
            expression = sympify(edge_desc['when'],
                                 locals=self.model._event_namespace)
            edge_desc['when'] = str(expression)
            self.model._values[str(expression)] = make_when_condition(
                expression, modules=self.model.modules)

    def _parse_conditions_durations(self, from_, edge_desc):
        """Parse the edge description in search for durations,
        escapement and conditions specifications. Durations
        ('duration' clause )are handled as an additional condition
        (agents entering the state are given a 'time to live' in the
        state, then they are not allowed to leave the state until
        their stay reaches that value). Escapements ('escape' clause)
        are also translated as a condition, allowing the agent to
        leave the state when the expression is true, only while the
        stay duration is below its nominal value.

        """
        cond, escape = None, False
        if 'cond' in edge_desc:
            cond = edge_desc['cond']
        if 'escape' in edge_desc:
            cond = edge_desc['escape']
            escape = True
        if cond is not None:
            ### WARNING the operation below is not completely
            ### safe... it is done to replace conditions of the form
            ### 'x == y' by 'Eq(x, y)', but it is a simple
            ### substitution instead of parsing the syntax
            ### tree... Thus it is *highly* recommended to express
            ### conditions directly with Eq(x, y)
            if '==' in str(cond):
                cond = 'Eq({})'.format(','.join(cond.split('==')))
                # edge_desc['label'] = ', '.join(edge_desc.values()) if
        # duration specified for this state, handle it as an
        # additional condition
        if 'duration' in self._statedesc[from_]:
            duration_cond = make_duration_condition(self.model, self.machine_name)
            if cond is None:
                cond = duration_cond
            elif escape:
                cond = 'AND(Not({}),{})'.format(duration_cond, cond)
            else:
                cond = 'AND({},{})'.format(duration_cond, cond)
            edge_desc['cond'] = cond
        if cond is not None:
            self.model.conditions[cond] = sympify(cond, locals=self.model._namespace)

[docs]    def build_actions(self):
        """Parse the description of the state machine and extract the
        actions that agents running this state machine must have.

        Example of YAML specification:
        ------------------------------
        actions:
          say_hello:
            desc: action performed when entering the S state

        """
        for name, value in self._statedesc.items():
            for keyword in ['on_enter', 'on_stay', 'on_exit']:
                if keyword in value:
                    self._add_state_actions(name, keyword, value[keyword])
            if 'duration' in value:
                val = value['duration']
                self._add_state_duration_actions(name, val)


[docs]    def get_value(self, name):
        """Return the value associated with the specified name."""
        return self.model.get_value(name)

def _add_state_duration_actions(self, state_name, duration_value):
        """Add implicit actions to manage stay duration in the specified state
        name. The `duration_value` can be either a parameter, a
        'statevar' or a distribution.

        """
        # initialize the actions associated to the state if none
        if state_name not in self.state_actions:
            self.state_actions[state_name] = {}
        # retrieve the list of actions on enter for this state, if any
        lenter = self.state_actions[state_name]['on_enter']\
                   if 'on_enter' in self.state_actions[state_name] else []
        # build a partial function based on the current state machine name
        enter_action = partial(make_duration_init_action,
                               machine_name=self.machine_name)
        # set the name of the action
        enter_action.__name__ = 'init_duration'
        # set the action parameters (the expression associated to the duration)
        enter_params = [self.model.add_expression(duration_value)]
        # instantiate the action
        init_action = AbstractAction.build_action('duration',
                                                  function=enter_action,
                                                  l_params=enter_params,
                                                  state_machine=self)
        # and insert it at the beginning of the list of actions
        lenter.insert(0, init_action)
        self.model.add_init_action(self.machine_name,
                                   self.states[state_name],
                                   init_action)
        # lstay = self.state_actions[name]['on_stay']\
        #           if 'on_stay' in self.state_actions[name] else []
        # stay_action = partial(make_TTL_increase_action,
        #                       machine_name=self.machine_name)
        # stay_action.__name__ = '+_time_spent'
        # lstay.insert(0, AbstractAction.build_action('duration',
        #                                             function=stay_action,
        #                                             state_machine=self))
        self.state_actions[state_name]['on_enter'] = lenter
        # self.state_actions[name]['on_stay'] = lstay

    def _add_state_actions(self, name, event, actions):
        """Add the specified actions for the state with the given
        name, associated with the event (e.g. 'on_stay', 'on_enter',
        'on_exit'). Expressions contained in the parameters lists or
        dicts are automatically expanded.

        """
        if name not in self.state_actions:
            self.state_actions[name] = {}
        l_actions = self._parse_action_list(actions)
        self.state_actions[name][event] = l_actions

    def _parse_action_list(self, actions):
        """Parse the list of actions associated with a state."""
        l_actions = []
        for d_action in actions:
            if 'action' in d_action:
                action = d_action['action']
                l_params = [self.model.add_expression(expr)
                            for expr in d_action['l_params']]\
                                if 'l_params' in d_action\
                                else []
                # if 'd_params' in d_action:
                #     print(d_action['d_params'])
                d_params = {key: self.model.add_expression(expr)
                            for key, expr in d_action['d_params'].items()}\
                                if 'd_params' in d_action\
                                else {}
                l_actions.append(
                    AbstractAction.build_action('action',
                                                method=action,
                                                l_params=l_params,
                                                d_params=d_params,
                                                state_machine=self))
            else: #TODO: dispatch through AbstractAction (factory),
                  #make subclasses responsible for parameter parsing
                understood = False
                for keyword in ['increase', 'decrease',
                                'increase_stoch', 'decrease_stoch']:
                    if keyword in d_action:
                        # assume that increase statevar with rate
                        l_actions.append(
                            AbstractAction.build_action(
                                keyword,
                                statevar_name=d_action[keyword],
                                parameter=self.model.add_expression(d_action['rate']),
                                delta_t=self.model.delta_t,
                                state_machine=self
                            )
                        )
                        understood = True
                for keyword in ['set_var']:
                    if keyword in d_action:
                        # assume that increase statevar with rate
                        l_actions.append(
                            AbstractAction.build_action(
                                keyword,
                                statevar_name=d_action[keyword],
                                parameter=self.model.add_expression(d_action['value']),
                                model=self.model
                            )
                        )
                        understood = True
                for keyword in ['become', 'clone', 'produce_offspring']:
                    if keyword in d_action:
                        amount = d_action['amount'] if 'amount' in d_action else None
                        probas = d_action['proba'] if 'proba' in d_action else None
                        l_actions.append(
                            AbstractAction.build_action(
                                keyword,
                                prototypes=d_action[keyword],
                                amount = amount,
                                probas = probas,
                                model = self.model
                            )
                        )
                        understood = True
                for keyword in ['log_vars']:
                    if keyword in d_action:
                        vars = [self.model.add_expression(varname)
                                for varname in d_action[keyword]]
                        l_actions.append(
                            AbstractAction.build_action(
                                keyword,
                                parameter=None,
                                l_params=vars
                            )
                        )
                        understood = True
                for keyword in ['message', 'record_change']:
                    if keyword in d_action:
                        l_actions.append(
                            AbstractAction.build_action(
                                keyword,
                                parameter=d_action[keyword]
                            )
                        )
                        understood = True
                if not understood:
                    print('ERROR !!!!') # but there is certainly a fatal error !
                    print(d_action)
        return l_actions


    #----------------------------------------------------------------
    # Output facilities

[docs]    def describe_state(self, name):
        """Return the description of the state with the specified
        name.

        """
        desc = self._statedesc[name]
        return "{} ({}):\n\t{}".format(name, desc['name'], desc['desc'])


[docs]    def write_dot(self, filename, view_actions=True):
        """Write the graph of the current state machine in the
        specified filename, according to the dot/graphviz format.

        """

        rankdir = "LR" if self.graph.edges() else "TB"
        output = '''digraph {
          charset="utf-8"
        '''
        output += '''\trankdir={};
        '''.format(rankdir)
        output += '''
        \tnode[fontsize=16, fontname=Arial, shape=box, style="filled,rounded"];
        \tedge[minlen=1.5, fontname=Times, penwidth=1.5, tailtooltip="", headtooltip=""];

        '''
        for state in self.states:
            name = state.name
            name_lab = name
            if 'duration' in self._statedesc[name]:
                name_lab += '&nbsp;{}'.format(CLOCK_SYMBOL)
            actions = 'shape="Mrecord", label="{}", '.format(name_lab)
            nodestyle = "filled,rounded"
            if state.is_default:
                nodestyle += ",bold"
            if state.autoremove:
                nodestyle += ",dotted"
            if view_actions:
                onenter = ACTION_SYMBOL+'|'\
                          if 'on_enter' in self._statedesc[name] else ''
                onstay = '|'+ACTION_SYMBOL\
                         if 'on_stay' in self._statedesc[name] else ''
                onexit = '|'+ACTION_SYMBOL\
                         if 'on_exit' in self._statedesc[name] else ''
                if onenter or onstay or onexit:
                    actions = 'shape="Mrecord", label="{%s{\ %s\ %s}%s}", ' % (
                        onenter, name_lab, onstay, onexit)
            output += '\t"{}" [{}tooltip="{}", fillcolor={}, style="{}"] ;\n'.format(
                name, actions,
                self._statedesc[name]['tooltip'],
                # '\n\tON ENTER: {}'.format(self.state_actions[name]['on_enter'])\
                # if onenter else '' +\
                # '\n\tON STAY: {}'.format(self.state_actions[name]['on_stay'])\
                # if onstay else '' +\
                # '\n\tON EXIT: {}'.format(self.state_actions[name]['on_exit'])\
                # if onexit else '',
                self._statedesc[name]['fillcolor'],
                nodestyle)
        for from_, to_ in SortedSet(self.graph.edges()):
            for desc in self.graph.edge[from_][to_].values():
                edgetip = ''
                tail = 'none'
                if 'when' in desc:
                    tail += WHEN_SYMBOL
                    edgetip += 'WHEN: {}'.format(desc['when'])
                if 'escape' in desc:
                    tail += ESCAPE_SYMBOL
                    edgetip += 'ESCAPE: {}'.format(desc['escape'])
                if 'truecond' in desc:
                    tail += COND_SYMBOL
                    edgetip += 'COND: {}'.format(desc['truecond'])
                head = 'normalnone'
                if 'on_cross' in desc:
                    head += CROSS_SYMBOL
                    # edgetip += 'ON CROSS: {}\\n'.format(desc['on_cross'])
                output += ('\t"{}" -> "{}" [label="{}", labeltooltip="{}", '
                           'arrowtail="{}", arrowhead="{}", dir=both, '
                           'tooltip="{}", minlen=3, style="{}"];\n').format(
                               from_, to_, desc['label'], desc['labeltooltip'],
                               tail, head, edgetip, desc['type_id'].linestyle)
        output += '}'
        with open(filename, 'w', encoding="utf8") as f:
            f.write(output)
```

# EMULSION

Epidemiological Multi-Level Simulation Framework

### Navigation

- 1. Installation
- 2. Getting started with EMULSION
- 3. Modelling principles
- 4. Modelling language (basics)
- 5. Modelling language (advanced)
- 6. Feature examples
- 7. Information
- 8. License
- 9. High-level functions for model designers
- 10. emulsion package

### Related Topics

- Documentation overview
  - Module code

### Quick search

©2016, INRA and Univ. Lille.
|
Powered by Sphinx 1.8.5
& Alabaster 0.7.10
