# Supplementary material for: EMULSION: Transparent and flexible multiscale stochastic models in human, animal and plant epidemiology
Source: PLoS Comput Biol. 2019 Sep 13;15(9):e1007342. doi: 10.1371/journal.pcbi.1007342 (PMC6760811; doi:10.1371/journal.pcbi.1007342)
Supplement: S2 File — This zip file is a clone of EMULSION public Git repository. To install from this file rather than from PyPI, go to EMULSION documentation page: https://sourcesup.renater.fr/www/emulsion-public/pages/Install.html and follow Git-based installation instructions. (ZIP) [file pcbi.1007342.s010.zip › S2_file/doc/_modules/emulsion/model/emulsion_model.html]

emulsion.model.emulsion\_model — EMULSION (Epidemiological Multi-Level Simulation framework)

# Source code for emulsion.model.emulsion\_model

```
"""
.. module:: emulsion.model.emulsion_model

.. moduleauthor:: Sébastien Picault <sebastien.picault@inra.fr>

"""

# [HEADER]


from   pathlib                 import Path
from   datetime                import datetime, timedelta
from   copy                    import deepcopy

import yaml
import dateutil.parser         as     dup

from   sympy                   import sympify, Symbol

from   sortedcontainers        import SortedSet, SortedDict
from   jinja2                  import Environment, PackageLoader

import networkx                as     nx

from   emulsion.tools.state    import StateVarDict
from   emulsion.tools.misc     import read_from_file, load_class, create_new_serial, create_weighted_random
from   emulsion.tools.calendar import EventCalendar

from   emulsion.model.functions      import DEFAULT_LEVEL_INFO, DEFAULT_GROUPING_INFO,\
    make_function, make_when_condition
from   emulsion.model.exceptions     import SemanticException
from   emulsion.model.state_machines import StateMachine

#  ______                 _     _             __  __           _      _
# |  ____|               | |   (_)           |  \/  |         | |    | |
# | |__   _ __ ___  _   _| |___ _  ___  _ __ | \  / | ___   __| | ___| |
# |  __| | '_ ` _ \| | | | / __| |/ _ \| '_ \| |\/| |/ _ \ / _` |/ _ \ |
# | |____| | | | | | |_| | \__ \ | (_) | | | | |  | | (_) | (_| |  __/ |
# |______|_| |_| |_|\__,_|_|___/_|\___/|_| |_|_|  |_|\___/ \__,_|\___|_|

[docs]class EmulsionModel(object):
    """Class in charge of the description of a multi-level
    epidemiological model. Such models are composed of several
    processes which may take place at different
    organization/observation levels. Some of those processes are
    reified through a function implemented within an agent, while
    others are represented by a state machine. The model is also in
    charge of handling symbolic expressions and pre-computing their
    values, e.g. concerning parameters, transmission functions,
    etc.

    """

[docs]    def __init__(self, filename=None, description=None):
        """Instantiate the model either from a configuration file or
        from a string. Both must contain a YAML-based description of
        the model.

        """
        if filename:
            self.filename = filename
            self.parse(read_from_file(filename))
        else:
            self.filename = None
            self.parse(description)

def __repr__(self):
        return '%s "%s"' % (self.__class__.__name__, self.model_name)

[docs]    def normalize_format(self):
        """Return a YAML representation of the model, reformatted to to print
        lists and dicts using the flow style (no [], no {}) instead of
        the block style, especially for syntax checking where rules
        are defined only for flow style.

        """
        return yaml.dump(self._description, default_flow_style=False)


[docs]    def copy(self):
        """Return a copy of object (naif method).
        TODO: Find a way to avoid recharging compartment class when
        intentiate a MultiProcessManager class with a old model.

        """
        return deepcopy(self)

def _reset_all(self):
        # namespace for all symbols used in the model
        # dict string -> sympy symbol
        self._namespace = {}
        # namespace for calendar periods in the model
        # dict string -> sympy symbol
        self._event_namespace = {}
        # cache for all expressions encountered in the model
        # dict string -> sympy expression
        self._expressions = {}
        # cache for all values encountered in the model
        # dict string -> value or function
        self._values = {}
        # the original description to parse (dict from YAML document)
        self._description = {}
        # default values for modules used in symbolic computing
        self.modules = ['emulsion.tools.functions', 'numpy', 'numpy.random', 'math', 'sympy']
        # name of the model
        self.model_name = 'New Model'
        # time unit (used to specify parameter values)
        self.time_unit = 'days'
        # duration of a simulation time step (number of time_units)
        self.delta_t = 1
        # duration of a simulation time step ('true' duration)
        self.step_duration = timedelta(days=1)
        # origin date for the simulation
        self.origin_date = datetime.now()
        # dictionary of calendars (keyed by their name)
        self.calendars = SortedDict()
        # reverse dict event -> calendar name
        self._events = {}
        # dict of levels
        self.levels = {}
        # dict of aggregate vars by level
        self.aggregate_vars = {}
        # list of the processes involved in the model
        self.processes = []
        # description of the compartment associated with some of the processes
        self.compartments = {}
        # description of the state machines associated with some of the processes
        self.state_machines = {}
        # dict of all 'parameters' encountered in the model
        self.parameters = StateVarDict()
        # dict of all 'statevars' encountered in the model
        self.statevars = StateVarDict()
        # dict of all conditions encountered in the model
        self.conditions = {}
        # dict of all actions encountered in the model
        self.actions = {}
        # dict of actions to run for state initialization
        self.init_actions = {}
        # dict of all distributions encountered in the model
        self.distributions = {}
        # dict of all prototypes encountered in the model
        self.prototypes = {}
        # dict of all initial conditions encountered in the model
        self.initial_conditions = {}
        # dict of enumerate types used in special variables
        self.types = {}
        # dict of all states existing in state machines
        self.states = StateVarDict()
        # dict of parameters to log when explicitly changed
        self.params_to_log = StateVarDict()

[docs]    def add_init_action(self, machine_name, state, action):
        """Add an action to be performed when initializing agents for
        the specified state of the state machine. Mainly used for
        durations.

        """
        if machine_name not in self.init_actions:
            self.init_actions[machine_name] = {}
        if state not in self.init_actions[machine_name]:
            self.init_actions[machine_name][state] = [action]
        else:
            self.init_actions[machine_name][state].append(action)

def _init_namespace(self):
        """Init the list of all encountered symbols, which have to be
        either parameters or state variables. The association between
        the name and the corresponding Symbol is stored in the
        namespace attribute.

        """
        # name space for 'regular' symbols (i.e. parameters, statevars)
        self._namespace.clear()

        for keyword in ['parameters', 'statevars']:
            if keyword in self._description:
                self._namespace.update({param: Symbol(param)
                                        for param in self._description[keyword]})
            else:
                self._description[keyword] = {}

[docs]    def get_value(self, name):
        """Return the value associated with the specified name. If the method
        is called with a name that is not in the model values, try to
        parse the expression, to prevent failures when called with a
        final value.

        """
        if name == "delta_t":
            return self.delta_t
        try:
            return self._values[name]
        except KeyError:
            try:
                return float(sympify(name, locals=self._namespace))
            except TypeError:
                ### cases of string parameters ?
                return name

# result = sympify(name, locals=self._namespace)
                # print(result, type(result))
                # import sys
                # sys.exit(-1)

[docs]    def change_parameter_values(self, changes, log_params=False):
        """Naive method to change several parameter values at the same time. """
        for name, value in changes.items():
            if name == 'delta_t':
                self._description['time_info']['delta_t'] = value # was int(value) previously
            else:
                self._description['parameters'][name]['value'] = value
        self.parse(self._description)
        self.params_to_log.clear()
        if log_params:
            for name in changes:
                value = self.get_value(name) if name != 'delta_t'\
                                             else self.delta_t
                self.params_to_log[name] = value


[docs]    def set_value(self, name, value):
        """Naif method to change a parameter's value.
        Will be more efficient when treating several parameters at
        the same time.

        """
        self._description['parameters'][name]['value'] = value
        self.parse(self._description)


[docs]    def parse(self, description):
        """Build the EmulsionModel from the specified dictionary
        (expected to come from a YAML configuration file).

        """
        self._reset_all()
        # keep an exhaustive description
        self._description = description
        # retrieve the name of the model
        self.model_name = self._description['model_name']
        # build association between symbols names (all parameter and
        # statevars names) and true sympy symbols
        self._init_namespace()
        # parse time informations
        self.build_timeinfo()
        # parse output options
        self.build_outputs_options()
        #  rapidly check state machines to build is_state and
        #  duration_in_state_machine properties
        self.check_state_machines()
        # parse parameters, state variables and distributions
        self.build_parameters()
        self.build_statevars()
        self.build_levels()
        self.build_distributions()
        # parse processes
        self.build_processes()
        # parse compartment description
        self.build_compartment_desc()
        # parse state machines
        self.build_state_machines()
        # parse prototypes
        self.build_prototypes()
        # parse initial_conditions
        self.build_initial_conds()
        # parse actions
        self.build_actions()
        # calculate expressions from parameters
        self.calculate_compound_params()
        # replace expressions by values or lambdas
        self.compute_values()
        self._description['parameters']['delta_t'] = {
            'desc': 'duration of the simulation time step',
            'value': self.delta_t
        }


[docs]    def build_outputs_options(self):
        """Parse the outputs options of the model.
        The agents will treat extra variables for outputs (TODO), and
        the simulation classes will treat period outputs.

        Example of YAML specification:
        ------------------------------
        outputs:
          # level
          herd:
            period:1
          # level
          metapop:
            period: 7
            extra_vars:
              - step
              - infection_date
        """
        if 'outputs' in self._description:
            self.outputs = self._description['outputs']
        else:
            self.outputs = {}


[docs]    def build_timeinfo(self):
        """Parse the description of the model and extract the
        information related to time management, i.e. time unit,
        duration of a simulation step, origin date, calendars for
        specific events, etc.

        Example of YAML specification:
        ------------------------------
        time_info:
        time_unit: days
        delta_t: 7
        origin: 'May 1'
        calendar:
          name:
          period: {days: 52}
          events:
            spring: {begin: 'April 8', end: 'April 23'}
            summer: {begin: 'July 8', end: 'September 3'}
            national: {date: 'July 14'}
        """
        self._event_namespace.clear()
        if 'time_info' in self._description:
            tinfo = self._description['time_info']
            # compute effective duration of one time step
            self.time_unit = tinfo['time_unit']
            # parse expression for delta_t if any and store its value
            # into the delta_t attribute
            # self.delta_t = tinfo['delta_t']
            self.delta_t = float(sympify(tinfo['delta_t'],
                                         locals=self._namespace))
            timedesc = {self.time_unit: self.delta_t}
            self.step_duration = timedelta(**timedesc)
            # print(self.step_duration)
            # origin date for the simulation
            if 'origin' in tinfo:
                self.origin_date = dup.parse(tinfo['origin'])
            # total duration for the simulation (in time units)
            if 'total_duration' in tinfo:
                self.parameters['total_duration'] =\
                        sympify(tinfo['total_duration'], locals=self._namespace)
            # handle calendars
            if 'calendars' in tinfo:
                self.build_calendar(tinfo['calendars'])


[docs]    def build_calendar(self, calendar_desc):
        """Build a representation of calendars."""
        # init name space for 'event' symbols (periods of
        # time) and handle period definitions
        for cal_name, cal in calendar_desc.items():
            events = {}
            if 'events' in cal:
                cal_period = timedelta(**cal['period']) if 'period' in cal else None
                for event_name, definition in cal['events'].items():
                    # register the event name in the events namespace
                    self._event_namespace[event_name] = Symbol(event_name)
                    # if keyword "date" is used, begin = end
                    if 'date' in definition:
                        events[event_name] = (dup.parse(definition['date']),
                                              dup.parse(definition['date']))
                    else:
                        events[event_name] = (dup.parse(definition['begin']),
                                              dup.parse(definition['end']))
                        # if begin and end date are specified,
                        # register events corresponding to "begin_event"
                        # and "end_event"
                        for keyword in ['begin', 'end']:
                            event_limit = keyword + '_' + event_name
                            self._event_namespace[event_limit] = Symbol(event_limit)
                            events[event_limit] = (dup.parse(definition[keyword]),
                                                   dup.parse(definition[keyword]))
                    # register the durations of each event
                    # compute duration of event
                    begin, end = events[event_name]
                    if end >= begin:
                        dur = end - begin
                    else:
                        dur = end - begin + cal_period
                    # compute duration of 1 time unit
                    unit = {self.time_unit: 1}
                    dur_unit = timedelta(**unit)
                    if (dur_unit > timedelta(days=1)):
                        dur += timedelta(days=1)
                    else: # 1h / 1min / 1s ...
                        dur += dur_unit
                    # convert duration to time units
                    event_duration = dur / dur_unit
                    duration_param = 'duration_of_{}'.format(event_name)
                    self._namespace[duration_param] = Symbol(duration_param)
                    self._values[duration_param] = sympify(event_duration)
                    # cal_name = cal['name']
            self.calendars[cal_name] = EventCalendar(cal_name,
                                                     self.step_duration,
                                                     self.origin_date,
                                                     cal_period,
                                                     **events)
            for event in self.calendars[cal_name].get_events():
                self._events[event] = cal_name
                expression = sympify(event, locals=self._event_namespace)
                self._values[str(expression)] = make_when_condition(
                    expression, modules=self.modules)


[docs]    def get_calendar_for_event(self, name):
        """Return the calendar providing the specified event name."""
        return self.calendars[self._events[name]]


[docs]    def build_parameters(self):
        """Parse the description of the model and extract the
        parameters, either with their value, or with the expression to
        compute them.

        Example of YAML specification:
        ------------------------------
        parameters:
          p:
            desc: infection probability
            value: '(1-exp(-E_total)) * (1 - phi*vaccinated)'
          phi:
            desc: efficiency of vaccination
            value: 0.79

        """
        if 'modules' in self._description:
            self.modules += self._description['modules']
        if 'parameters' in self._description:
            for (key, val) in self._description['parameters'].items():
                self.parameters[key] = sympify(val['value'],
                                               locals=self._namespace)


[docs]    def build_statevars(self):
        """Parse the description of the model and extract the state
        variables that agents running this model must have.

        Example of YAML specification:
        ------------------------------
        statevars:
          E_total:
            desc: total bacteria deposited in the environment
          vaccinated:
            desc: 0/1 value describing the vaccination state

        """
        if 'statevars' in self._description:
            self.statevars = StateVarDict(self._description['statevars'])


[docs]    def build_distributions(self):
        """Parse the description of the model and extract the
        distributions, either with their value, or with the expression
        to compute them. A distribution is a dictionary of the form
        {quantity: probability}. It is stored as a list of tuples
        (quantity, probability) which is more convenient for
        subsequent processing.

        Example of YAML specification:
        ------------------------------
        distributions:
          - shedding_dist1:
              desc: distribution of shedding
              value:
                low_shedding: 0.85
                med_shedding: 0.15

        """
        if 'distributions' in self._description:
            for list_item in self._description['distributions']:
                for (key, val) in list_item.items():
                    self.distributions[key] = [
                        (sympify(quantity, locals=self._namespace),
                         sympify(probability, locals=self._namespace))
                        for quantity, probability in val['value'].items()]


[docs]    def build_initial_conds(self):
        """Parse the description of the model and extract the initial
        conditions for each level, either with their value, of with
        the expression to compute them. An initial condition is a
        dictionary containing a description of the 'content' of the
        level (i.e. the quantity and initial state of sub-levels):
        - either the 'population:' keyword followed by a dict State -> Amount
        - or 'prototype:' and 'amount:' keywords followed by their values

        Example of YAML specification:
        ------------------------------
        initial_conditions:
          metapop:
            - prototype: healthy # defines init_prevalence
              amount: 100
            - prototype: sick
              amount: 1
          herd:
            - population:
                infection:
                  S: 'init_pop * (1 - init_prevalence)'
                  I: 'init_pop * init_prevalence'

        """
        if 'initial_conditions' in self._description:
            for level, desc in self._description['initial_conditions'].items():
                self.initial_conditions[level] = {
                    'total': '0',
                    'populations': [],
                    'prototypes': []
                }
                for list_items in desc:
                    ## build initial conditions for compartment-based model
                    if 'population' in list_items:
                        for pop_spec in list_items['population']:
                            if 'total' in pop_spec:
                                self.initial_conditions[level]['total'] = self.add_expression(pop_spec['total'])
                            else:
                                keys = pop_spec['vars']
                                amount = pop_spec['amount']
                                if not isinstance(keys, list):
                                    keys = [keys]
                                self.initial_conditions[level]['populations'].append(
                                    (keys, self.add_expression(amount))
                                )
                    else:
                        ## build initial conditions for individual-based model
                        protos = list_items['prototype']
                        if not isinstance(protos, list):
                            protos = [protos]
                            list_items['prototype'] = protos
                        amount = list_items['amount']
                        if 'proba' in list_items:
                            probas = list_items['proba']
                            if not isinstance(probas, list):
                                probas = [probas]
                        else:
                            probas = ['1'] * len(protos)
                        list_items['proba'] = probas
                        assert(0 <= len(protos) - len(probas) <= 1)
                        self.initial_conditions[level]['prototypes'].append(
                            (protos, self.add_expression(amount), [self.add_expression(p) for p in probas]))

# print(self._description['initial_conditions'])
        # print(self.initial_conditions)

[docs]    def build_prototypes(self):
        """Parse the description of the model and extract the list of
        prototypes for each level, either with their value, or with
        the expression to compute them. A prototype is a dictionary of
        the form {statevar: value}..

        Example of YAML specification:
        ------------------------------
        prototypes:
          animals:
            - newborn:
                desc: prototype for new calves
                health_state: M
                life_state: NP
                age: 0

        """
        if 'prototypes' in self._description:
            for level, prototypes in self._description['prototypes'].items():
                self.prototypes[level] = {}
                for list_item in prototypes:
                    for (key, val) in list_item.items():
                        self.prototypes[level][key] = {}
                        for variable, value in val.items():
                            result = value
                            if variable != 'desc':
                                if value == 'random':
                                    result = '_random_' + variable
                                elif value == 'default':
                                    result = '_default_' + variable
                                elif value == 'next_state':
                                    result = '_next_state_' + variable
                                    # associate to a function that returns the successor of the state contained in the variable (or a random state if the variable was not defined)
                                    self._values[result] = lambda agent: agent.statevars[variable].successor if variable in agent.statevars else agent.model.state_machines[variable].get_random_state()
                                elif value == 'previous_state':
                                    result = '_previous_state_' + variable
                                    # associate to a function that returns the predecessor of the state contained in the variable (or a random state if the variable was not defined)
                                    self._values[result] = lambda agent: agent.statevars[variable].predecessor if variable in agent.statevars else agent.model.state_machines[variable].get_random_state()
                                elif type(value) == str and value.startswith('random('):
                                    # result = '_weighted_random_' + variable
                                    result = '{}_{}'.format(variable, value)
                                    if value[6] == '(' and value[-1] == ')':
                                        weights = [self.add_expression(e) for e in value[7:-1].split(',')]
                                    self._values[result] = create_weighted_random(variable, weights, model=self)
                                elif type(value) == str and value.startswith('serial'):
                                    result = '_serial_' + variable
                                    if value != 'serial' and value[6] == '(' and value[-1] == ')':
                                        end = self.add_expression(value[7:-1])
                                    else:
                                        start, end = 0, None
                                    self._values[result] = create_new_serial(end=end, model=self)
                                elif value in self.statevars:
                                    self._values[result] = lambda agent: agent.statevars[result]
                                self.prototypes[level][key][variable] = result


[docs]    def get_prototype(self, level, name):
        """Return a ready-to-use prototype, i.e. a StateVarDict corresponding
        to the prototype for the specified level and name, where
        symbols associated to statevariables are already replaced by
        their value in the model. 'Hard-coded' lists are transformed
        into tuples (to be hashable).

        """
        return StateVarDict({
            var: tuple(val) if isinstance(val, list) else self.get_value(val)
            for var, val in self.prototypes[level][name].items()
        })


[docs]    def build_levels(self):
        """Parse the description of different level of simulations.
        Most of time, there are tree differents levels:
        individual, herd, metapopulation.

        Example of YAML specification:
        ------------------------------
        levels:
          individuals:
            super:
              class: AtomAgent
            class_name: Cow
          herd:
            super:
              class: MultiProcessManager
            class_name: QfeverHerd
          metapop:
            super:
              class: MultiProcessManager
              master_class: StructuredView
            class_name: QfeverMetaPop

        """
        if 'levels' in self._description:
            self.levels = self._description['levels']
            self.root_level = None
            self.levels_graph = nx.DiGraph()

        # build levels graph
        for level in self.levels:
            self.levels_graph.add_node(level)
            desc = self.levels[level]
            if 'contains' in desc:
                for sublevel in desc['contains']:
                    self.levels_graph.add_edge(level, sublevel)

        # check consistency
        if nx.is_arborescence(self.levels_graph):
            self.root_level = list(nx.topological_sort(self.levels_graph))[0]
        else:
            print('Warning, levels do not consitute an arborescence!')
            print(self.levels_graph.edges, self.levels_graph.nodes)
        # print(self.levels)

        # add default information if missing
        for level in self.levels:
            desc = self.levels[level]
            if 'module' not in desc:
                # try to build module from file
                if 'file' in desc:
                    path = Path(desc['file'])
                    desc['module'] = '.'.join(path.parent.parts + (path.stem,))
                    # if no class name build one ("DefaultLevelnameClass")
                    if 'class_name' not in desc:
                        desc['class_name'] = "Default{}Class".format(level.capitalize())
                else:
                    # if level built by aggregation use default level class
                    if 'aggregation_type' in desc:
                        desc['module'] = DEFAULT_LEVEL_INFO[desc['aggregation_type']]['level']['module']
                        desc['class_name'] = DEFAULT_LEVEL_INFO[desc['aggregation_type']]['level']['class_name']
                    else:
                        # try to retrieve upper level if any
                        predecessors = list(self.levels_graph.predecessors(level))
                        if not predecessors:
                            desc['module'] = DEFAULT_LEVEL_INFO['IBM']['sublevels']['module']
                            desc['class_name'] = DEFAULT_LEVEL_INFO['IBM']['sublevels']['class_name']
                        else: # take first information regarding aggregation type
                            ## TODO (research topic on multi-level
                            ## design patterns): hierarchize possibly
                            ## different aggregation types from upper
                            ## levels
                            for pred in predecessors:
                                desc_pred = self.levels[pred]
                                if not 'aggregation_type' in desc_pred:
                                    continue
                                pred_info = DEFAULT_LEVEL_INFO[desc_pred['aggregation_type']]
                                if 'sublevels' not in pred_info:
                                    continue
                                desc['module'] = pred_info['sublevels']['module']
                                desc['class_name'] = pred_info['sublevels']['class_name']
                            if 'module' not in desc:
                                desc['module'] = None
                                desc['class_name'] = None
            if 'aggregation_type' in desc:
                default = DEFAULT_LEVEL_INFO[desc['aggregation_type']]
                if 'super' not in desc:
                    desc['super'] = default['level']
                if not desc['module'].startswith('emulsion.agent'):
                    if 'sublevels' in default:
                        if 'contains' in desc:
                            for sublevel in desc['contains']:
                                if ('module' not in self.levels[sublevel] or\
                                    (not self.levels[sublevel]['module'].startswith('emulsion.agent')))\
                                   and ('super' not in self.levels[sublevel]):
                                    self.levels[sublevel]['super'] = default['sublevels']
                if 'aggregate_vars' in desc:
                    if level not in self.aggregate_vars:
                        self.aggregate_vars[level] = []
                    for agg_desc in desc['aggregate_vars']:
                        newvar = agg_desc['name']
                        sourcevar = agg_desc['collect']
                        oper = agg_desc['operator'] if 'operator' in agg_desc else 'sum'
                        self.aggregate_vars[level].append((newvar, sourcevar, oper))

# print(self.aggregate_vars)

[docs]    def get_agent_class_for_level(self, level):
        return load_class(module=self.levels[level]['module'],
                          class_name=self.levels[level]['class_name'])


[docs]    def build_processes(self):
        """Parse the description of the model and retrieve the list of
        processes with different level.

        Example of YAML specification:
        ------------------------------
        processes:
          herd:
            - bacterial_dispersion
            - culling_process
            - infection
          metapop:
            - inbox_distribution
            - outbox_distribution
        """
        if 'processes' in self._description:
            self.processes = self._description['processes']


[docs]    def check_state_machines(self):
        """Rapidly inspect the name of state machines and of their states to
        pre-register automatically built statevars.

        """
        if 'state_machines' in self._description:
            for machine_name, description in self._description['state_machines'].items():
                self.state_machines[machine_name] = None # to build later
                desc = "duration elapsed (time units) in current {} state".format(machine_name)
                prop_name = 'duration_in_{}'.format(machine_name)
                # self._description['statevars'][prop_name] = {'desc': desc}
                self.statevars[prop_name] = {'desc': desc}
                self._description['statevars'][prop_name] = {'desc': desc}
                self._namespace[prop_name] = Symbol(prop_name)
                desc = "time step when agent entered current {} state".format(machine_name)
                prop_name = '_time_entered_{}'.format(machine_name)
                self.statevars[prop_name] = {'desc': desc}
                self._description['statevars'][prop_name] = {'desc': desc}
                self._namespace[prop_name] = Symbol(prop_name)
                desc = "time step after which agent is allowed to leave current {} state".format(machine_name)
                prop_name = '_time_to_exit_{}'.format(machine_name)
                self.statevars[prop_name] = {'desc': desc}
                self._description['statevars'][prop_name] = {'desc': desc}
                self._namespace[prop_name] = Symbol(prop_name)
                for statedict in description['states']:
                    for state_name in statedict:
                        desc = "Test if {} = {}".format(machine_name, state_name)
                        prop_name = 'is_{}'.format(state_name)
                        self._description['statevars'][prop_name] = {'desc': desc}
                        # self.statevars[prop_name] = desc
                        self._namespace[prop_name] = Symbol(prop_name)

# print(self._namespace, self.statevars)

[docs]    def build_state_machines(self):
        """Parse the description of the model and build all the
        required state machines.

        """
        if 'state_machines' in self._description:
            for machine_name, description in self._description['state_machines'].items():
                self.state_machines[machine_name] = StateMachine(machine_name,
                                                                 description,
                                                                 self)


[docs]    def build_compartment_desc(self):
        """Inspect the `grouping` part of the model (if
        any) in order to store the corresponding information.

        """
        if 'grouping' in self._description:
            self.compartments = self._description['grouping']
            for level in self.levels.keys():
                if level not in self.compartments:
                    self.compartments[level] = {}
                if 'aggregation_type' in self.levels[level]:
                    agg_type = self.levels[level]['aggregation_type']
                    if agg_type in DEFAULT_GROUPING_INFO:
                        default = DEFAULT_GROUPING_INFO[agg_type]
                        for desc in self.compartments[level].values():
                            if 'compart_manager' not in desc:
                                desc['compart_manager'] = default['compart_manager']\
                                  if  'machine_name' in desc\
                                  else default['fallback_view']
                            if 'compart_class' not in desc:
                                desc['compart_class'] = default['compart_class']

# print(self.compartments)

[docs]    def calculate_compound_params(self):
        """Inspect all edges of the health states graph and compute
        the actual probabilities associated to expressions.
        ### TODO: check that the ultimate symbols are declared properties.

        """
        for cond, expr in self.conditions.items():
            self.conditions[cond] = self.expand_expression(expr)


[docs]    def add_expression(self, expression):
        """Add the specified expression to the dictionary of known
        expressions.

        """
        if expression not in self.parameters\
           and expression not in self.statevars\
           and expression not in self._expressions:
            self._expressions[expression] = self.expand_expression(expression)
        return expression


[docs]    def expand_expression(self, expression):
        """Transform the specified expression so as to replace all
        parameters by actual values or state variables or
        attributes.

        """
        ### WARNING: expand_expressions should iterate over all
        ### expressions at the same time (halting when no change
        ### occurs) instead of iterating over each expression one by
        ### one
        result = sympify(expression, locals=self._namespace)
        expr = result
        symbs = {s: self.parameters[s.name]
                 for s in expr.free_symbols
                 if s.name in self.parameters}
        while symbs:
            result = expr.subs(symbs)
            expr = sympify(result, locals=self._namespace)
            symbs = {s: self.parameters[s.name]
                     for s in expr.free_symbols
                     if s.name in self.parameters}
        return result


[docs]    def build_actions(self):
        """Parse the description of the model and extract the actions
        that agents must have.

        Example of YAML specification:
        ------------------------------
        actions:
          say_hello:
            desc: action performed when entering the S state

        """
        if 'actions' in self._description:
            self.actions = StateVarDict(self._description['actions'])


[docs]    def compute_values(self):
        """Check parameters and calculated compound parameters, so as
        to make them computable. In the case of parameters, number
        values are left unchanged, but strings (representing an
        expression) are replaced by a function. Regarding calculated
        compound parameters, expressions corresponding to numbers are
        converted to float values, while other expressions are
        replaced by a function.

        """
        # collect 'true' values in the parameters
        for (param, expression) in self.parameters.items():
            value = self.expand_expression(expression)
            try:
                self._values[param] = float(value)
            except:
                self._values[param] = make_function(value,
                                                    modules=self.modules)
        for (param, expression) in self._expressions.items():
            value = self.expand_expression(expression)
            try:
                self._values[param] = float(value)
            except:
                self._values[param] = make_function(value,
                                                    modules=self.modules)
        for (cond, expression) in self.conditions.items():
            value = self.expand_expression(expression)
            if any([str(symb) in self.statevars
                    for symb in expression.free_symbols]):
                self._values[cond] = make_function(value,
                                                   dtype=bool,
                                                   modules=self.modules)
            else:
                self._values[cond] = bool(value)
        for (statename, state) in self.states.items():
            self._values[statename] = state


[docs]    def get_modifiable_parameters(self):
        """Return a dictionary containing all true parameters with their
        value.

        """
        true_params =  {p: self.get_value(p)
                        for p in self.parameters
                        if not callable(self.get_value(p))}
        true_params['delta_t'] = self.delta_t
        return true_params


[docs]    def describe_parameter(self, name):
        """Return the description of the parameter with the specified
        name.

        """
        # pretty_name = pretty(sympify(name, locals=self._namespace))
        # param_name = name if pretty_name == name\
        #              else "{} ({})".format(pretty_name, name)
        param_name = name
        return "{} [parameter]:\n\t{: <72}\n\t{}".format(
            param_name,
            self._description['parameters'][name]['desc'],
            self._description['parameters'][name]['value']
        )


[docs]    def describe_variable(self, name):
        """Return the description of the statevar with the specified
        name.

        """
        # pretty_name = pretty(sympify(name, locals=self._namespace))
        # var_name = name if pretty_name == name\
        #            else "{} ({})".format(pretty_name, name)
        var_name = name
        return "{} [variable]:\n\t{: <72}".format(
            var_name,
            self._description['statevars'][name]['desc'],
        )


[docs]    def describe_name(self, name):
        """Return the description of the specified name in the model
        name.

        """
        if name in self._description['parameters']:
            return self.describe_parameter(name)
        elif name in self._description['statevars']:
            return self.describe_variable(name)
        else:
            return "{}: UNKNOWN NAME".format(name)


[docs]    def write_dot(self, parent_dir):
        """Write the graph of the each state machine in the
        specified directer name, according to the dot/graphviz format.

        """
        for name, statemachine in self.state_machines.items():
            name = self.model_name + '_' + name + '.dot'
            path = str(Path(parent_dir, name))
            statemachine.write_dot(path)


[docs]    def generate_skeleton(self, module):
        """Output a code skeleton to help writing specific pieces of code for
        the specified module to make the model work under Emulsion.

        """
        env = Environment(
            loader=PackageLoader('emulsion', 'templates'),
            extensions=['jinja2.ext.do']
        )
        template = env.get_template('specific_code.py')
        output = template.render(model=self, src_module=module)
        return output

EmulsionModel.get_modifiable_parameters.__USER_METHOD__ = ['Introspection']
EmulsionModel.describe_parameter.__USER_METHOD__ = ['Introspection']
```

# EMULSION

Epidemiological Multi-Level Simulation Framework

### Navigation

- 1. Installation
- 2. Getting started with EMULSION
- 3. Modelling principles
- 4. Modelling language (basics)
- 5. Modelling language (advanced)
- 6. Feature examples
- 7. Information
- 8. License
- 9. High-level functions for model designers
- 10. emulsion package

### Related Topics

- Documentation overview
  - Module code

### Quick search

©2016, INRA and Univ. Lille.
|
Powered by Sphinx 1.8.5
& Alabaster 0.7.10
