# Supplementary material for: EMULSION: Transparent and flexible multiscale stochastic models in human, animal and plant epidemiology
Source: PLoS Comput Biol. 2019 Sep 13;15(9):e1007342. doi: 10.1371/journal.pcbi.1007342 (PMC6760811; doi:10.1371/journal.pcbi.1007342)
Supplement: S2 File — This zip file is a clone of EMULSION public Git repository. To install from this file rather than from PyPI, go to EMULSION documentation page: https://sourcesup.renater.fr/www/emulsion-public/pages/Install.html and follow Git-based installation instructions. (ZIP) [file pcbi.1007342.s010.zip › S2_file/doc/_modules/emulsion/init_emulsion.html]

emulsion.init\_emulsion — EMULSION (Epidemiological Multi-Level Simulation framework)

# Source code for emulsion.init\_emulsion

```
#!/usr/bin/env python3

# [HEADER]

import os
from   pathlib    import Path
from   shutil     import copy

from   colorama   import Style
import emulsion

COMMAND = 'source $HOME/.emulsionrc/emulsion-completion.sh'
CPATH = 'export PYTHONPATH=$PYTHONPATH:.'

[docs]def main():
    # retrieve $HOME and path to EMULSION repository
    parts = Path(emulsion.__file__).parts[:-1] + ("scripts",)
    HOMEDIR = Path.home()
    EMULSION_SCRIPTS = Path(*parts)
    EMULSION_RC = Path(HOMEDIR, '.emulsionrc')
    # create .emulsionrc
    print("Creating $HOME/.emulsionrc with EMULSION init files")
    EMULSION_RC.mkdir(exist_ok=True)
    # copy emulsion-completion.sh
    copy(str(Path(EMULSION_SCRIPTS, 'emulsion-completion.sh')),
         str(EMULSION_RC))
    # change .bashrc
    print("Adding initialization instructions to $HOME/.bashrc")
    with open(Path(HOMEDIR, '.bashrc'), 'a') as f:
        f.write("\n# init EMULSION completion\n")
        f.write(COMMAND + "\n")
        f.write("\n# update PYTHONPATH to allow importing local modules\n")
        f.write(CPATH + "\n")

    print("Initialization finished.")
    print(Style.BRIGHT + """
    To allow completion for EMULSION in current shell, type:
    source $HOME/.bashrc
    """ + Style.RESET_ALL)

if __name__ == '__main__':
    main()
```

# EMULSION

Epidemiological Multi-Level Simulation Framework

### Navigation

- 1. Installation
- 2. Getting started with EMULSION
- 3. Modelling principles
- 4. Modelling language (basics)
- 5. Modelling language (advanced)
- 6. Feature examples
- 7. Information
- 8. License
- 9. High-level functions for model designers
- 10. emulsion package

### Related Topics

- Documentation overview
  - Module code

### Quick search

©2016, INRA and Univ. Lille.
|
Powered by Sphinx 1.8.5
& Alabaster 0.7.10
