# Supplementary material for: EMULSION: Transparent and flexible multiscale stochastic models in human, animal and plant epidemiology
Source: PLoS Comput Biol. 2019 Sep 13;15(9):e1007342. doi: 10.1371/journal.pcbi.1007342 (PMC6760811; doi:10.1371/journal.pcbi.1007342)
Supplement: S2 File — This zip file is a clone of EMULSION public Git repository. To install from this file rather than from PyPI, go to EMULSION documentation page: https://sourcesup.renater.fr/www/emulsion-public/pages/Install.html and follow Git-based installation instructions. (ZIP) [file pcbi.1007342.s010.zip › S2_file/doc/py-modindex.html]

Python Module Index — EMULSION (Epidemiological Multi-Level Simulation framework)

# Python Module Index

**e**

|  |  |  |
| --- | --- | --- |
|  |  |  |
|  | **e** |  |
|  | `emulsion` |  |
|  | `emulsion.agent` |  |
|  | `emulsion.agent.action` |  |
|  | `emulsion.agent.atoms` |  |
|  | `emulsion.agent.comparts` |  |
|  | `emulsion.agent.core` |  |
|  | `emulsion.agent.core.abstract_agent` |  |
|  | `emulsion.agent.core.asbtract_agent` |  |
|  | `emulsion.agent.core.emulsion_agent` |  |
|  | `emulsion.agent.core.groups` |  |
|  | `emulsion.agent.exceptions` |  |
|  | `emulsion.agent.managers` |  |
|  | `emulsion.agent.managers.abstract_process_manager` |  |
|  | `emulsion.agent.managers.compart_process_manager` |  |
|  | `emulsion.agent.managers.functions` |  |
|  | `emulsion.agent.managers.group_manager` |  |
|  | `emulsion.agent.managers.ibm_process_manager` |  |
|  | `emulsion.agent.managers.metapop_process_manager` |  |
|  | `emulsion.agent.managers.multi_process_manager` |  |
|  | `emulsion.agent.meta` |  |
|  | `emulsion.agent.process` |  |
|  | `emulsion.agent.views` |  |
|  | `emulsion.init_emulsion` |  |
|  | `emulsion.model` |  |
|  | `emulsion.model.emulsion_model` |  |
|  | `emulsion.model.exceptions` |  |
|  | `emulsion.model.functions` |  |
|  | `emulsion.model.state_machines` |  |
|  | `emulsion.tools` |  |
|  | `emulsion.tools.calendar` |  |
|  | `emulsion.tools.functions` |  |
|  | `emulsion.tools.graph` |  |
|  | `emulsion.tools.misc` |  |
|  | `emulsion.tools.parallel` |  |
|  | `emulsion.tools.plot` |  |
|  | `emulsion.tools.simulation` |  |
|  | `emulsion.tools.state` |  |
|  | `emulsion.tools.timing` |  |
|  | `emulsion.tools.view` |  |

# EMULSION

Epidemiological Multi-Level Simulation Framework

### Navigation

- 1. Installation
- 2. Getting started with EMULSION
- 3. Modelling principles
- 4. Modelling language (basics)
- 5. Modelling language (advanced)
- 6. Feature examples
- 7. Information
- 8. License
- 9. High-level functions for model designers
- 10. emulsion package

### Related Topics

- Documentation overview

### Quick search

©2016, INRA and Univ. Lille.
|
Powered by Sphinx 1.8.5
& Alabaster 0.7.10
