# Supplementary material for: EMULSION: Transparent and flexible multiscale stochastic models in human, animal and plant epidemiology
Source: PLoS Comput Biol. 2019 Sep 13;15(9):e1007342. doi: 10.1371/journal.pcbi.1007342 (PMC6760811; doi:10.1371/journal.pcbi.1007342)
Supplement: S2 File — This zip file is a clone of EMULSION public Git repository. To install from this file rather than from PyPI, go to EMULSION documentation page: https://sourcesup.renater.fr/www/emulsion-public/pages/Install.html and follow Git-based installation instructions. (ZIP) [file pcbi.1007342.s010.zip › S2_file/doc/emulsion.model.html]

emulsion.model package — EMULSION (Epidemiological Multi-Level Simulation framework)

# emulsion.model package¶

*Module author: Sébastien Picault <sebastien.picault@inra.fr>*

## Submodules¶

## emulsion.model.emulsion\_model module¶

*Module author: Sébastien Picault <sebastien.picault@inra.fr>*

*class* `emulsion.model.emulsion_model.``EmulsionModel`(*filename=None*, *description=None*)[source]¶
:   Bases: `object`

    Class in charge of the description of a multi-level
    epidemiological model. Such models are composed of several
    processes which may take place at different
    organization/observation levels. Some of those processes are
    reified through a function implemented within an agent, while
    others are represented by a state machine. The model is also in
    charge of handling symbolic expressions and pre-computing their
    values, e.g. concerning parameters, transmission functions,
    etc.

    `__init__`(*filename=None*, *description=None*)[source]¶
    :   Instantiate the model either from a configuration file or
        from a string. Both must contain a YAML-based description of
        the model.

    `add_expression`(*expression*)[source]¶
    :   Add the specified expression to the dictionary of known
        expressions.

    `add_init_action`(*machine\_name*, *state*, *action*)[source]¶
    :   Add an action to be performed when initializing agents for
        the specified state of the state machine. Mainly used for
        durations.

    `build_actions`()[source]¶
    :   Parse the description of the model and extract the actions
        that agents must have.

        actions:
        :   say\_hello:
            :   desc: action performed when entering the S state

    `build_calendar`(*calendar\_desc*)[source]¶
    :   Build a representation of calendars.

    `build_compartment_desc`()[source]¶
    :   Inspect the grouping part of the model (if
        any) in order to store the corresponding information.

    `build_distributions`()[source]¶
    :   Parse the description of the model and extract the
        distributions, either with their value, or with the expression
        to compute them. A distribution is a dictionary of the form
        {quantity: probability}. It is stored as a list of tuples
        (quantity, probability) which is more convenient for
        subsequent processing.

        distributions:
        :   - shedding\_dist1:
              :   desc: distribution of shedding
                  value:

                  > low\_shedding: 0.85
                  > med\_shedding: 0.15

    `build_initial_conds`()[source]¶
    :   Parse the description of the model and extract the initial
        conditions for each level, either with their value, of with
        the expression to compute them. An initial condition is a
        dictionary containing a description of the ‘content’ of the
        level (i.e. the quantity and initial state of sub-levels):
        - either the ‘population:’ keyword followed by a dict State -> Amount
        - or ‘prototype:’ and ‘amount:’ keywords followed by their values

        initial\_conditions:
        :   metapop:
            :   - prototype: healthy # defines init\_prevalence
                  amount: 100
                - prototype: sick
                  amount: 1

            herd:
            :   - population:
                  :   infection:
                      :   S: ‘init\_pop \* (1 - init\_prevalence)’
                          I: ‘init\_pop \* init\_prevalence’

    `build_levels`()[source]¶
    :   Parse the description of different level of simulations.
        Most of time, there are tree differents levels:
        individual, herd, metapopulation.

        levels:
        :   individuals:
            :   super:
                :   class: AtomAgent

                class\_name: Cow

            herd:
            :   super:
                :   class: MultiProcessManager

                class\_name: QfeverHerd

            metapop:
            :   super:
                :   class: MultiProcessManager
                    master\_class: StructuredView

                class\_name: QfeverMetaPop

    `build_outputs_options`()[source]¶
    :   Parse the outputs options of the model.
        The agents will treat extra variables for outputs (TODO), and
        the simulation classes will treat period outputs.

        outputs:
        :   # level
            herd:

            > period:1

            # level
            metapop:

            > period: 7
            > extra\_vars:
            >
            > > - step
            > > - infection\_date

    `build_parameters`()[source]¶
    :   Parse the description of the model and extract the
        parameters, either with their value, or with the expression to
        compute them.

        |  |  |
        | --- | --- |
        | Parameters: | - **p** – desc: infection probability   value: ‘(1-exp(-E\_total)) \* (1 - phi\*vaccinated)’ - **phi** – desc: efficiency of vaccination   value: 0.79 |

    `build_processes`()[source]¶
    :   Parse the description of the model and retrieve the list of
        processes with different level.

        processes:
        :   herd:
            :   - bacterial\_dispersion
                - culling\_process
                - infection

            metapop:
            :   - inbox\_distribution
                - outbox\_distribution

    `build_prototypes`()[source]¶
    :   Parse the description of the model and extract the list of
        prototypes for each level, either with their value, or with
        the expression to compute them. A prototype is a dictionary of
        the form {statevar: value}..

        prototypes:
        :   animals:
            :   - newborn:
                  :   desc: prototype for new calves
                      health\_state: M
                      life\_state: NP
                      age: 0

    `build_state_machines`()[source]¶
    :   Parse the description of the model and build all the
        required state machines.

    `build_statevars`()[source]¶
    :   Parse the description of the model and extract the state
        variables that agents running this model must have.

        statevars:
        :   E\_total:
            :   desc: total bacteria deposited in the environment

            vaccinated:
            :   desc: 0/1 value describing the vaccination state

    `build_timeinfo`()[source]¶
    :   Parse the description of the model and extract the
        information related to time management, i.e. time unit,
        duration of a simulation step, origin date, calendars for
        specific events, etc.

        time\_info:
        time\_unit: days
        delta\_t: 7
        origin: ‘May 1’
        calendar:

        > name:
        > period: {days: 52}
        > events:
        >
        > > spring: {begin: ‘April 8’, end: ‘April 23’}
        > > summer: {begin: ‘July 8’, end: ‘September 3’}
        > > national: {date: ‘July 14’}

    `calculate_compound_params`()[source]¶
    :   Inspect all edges of the health states graph and compute
        the actual probabilities associated to expressions.
        ### TODO: check that the ultimate symbols are declared properties.

    `change_parameter_values`(*changes*, *log\_params=False*)[source]¶
    :   Naive method to change several parameter values at the same time.

    `check_state_machines`()[source]¶
    :   Rapidly inspect the name of state machines and of their states to
        pre-register automatically built statevars.

    `compute_values`()[source]¶
    :   Check parameters and calculated compound parameters, so as
        to make them computable. In the case of parameters, number
        values are left unchanged, but strings (representing an
        expression) are replaced by a function. Regarding calculated
        compound parameters, expressions corresponding to numbers are
        converted to float values, while other expressions are
        replaced by a function.

    `copy`()[source]¶
    :   Return a copy of object (naif method).
        TODO: Find a way to avoid recharging compartment class when
        intentiate a MultiProcessManager class with a old model.

    `describe_name`(*name*)[source]¶
    :   Return the description of the specified name in the model
        name.

    `describe_parameter`(*name*)[source]¶
    :   Return the description of the parameter with the specified
        name.

    `describe_variable`(*name*)[source]¶
    :   Return the description of the statevar with the specified
        name.

    `expand_expression`(*expression*)[source]¶
    :   Transform the specified expression so as to replace all
        parameters by actual values or state variables or
        attributes.

    `generate_skeleton`(*module*)[source]¶
    :   Output a code skeleton to help writing specific pieces of code for
        the specified module to make the model work under Emulsion.

    `get_agent_class_for_level`(*level*)[source]¶

    `get_calendar_for_event`(*name*)[source]¶
    :   Return the calendar providing the specified event name.

    `get_modifiable_parameters`()[source]¶
    :   Return a dictionary containing all true parameters with their
        value.

    `get_prototype`(*level*, *name*)[source]¶
    :   Return a ready-to-use prototype, i.e. a StateVarDict corresponding
        to the prototype for the specified level and name, where
        symbols associated to statevariables are already replaced by
        their value in the model. ‘Hard-coded’ lists are transformed
        into tuples (to be hashable).

    `get_value`(*name*)[source]¶
    :   Return the value associated with the specified name. If the method
        is called with a name that is not in the model values, try to
        parse the expression, to prevent failures when called with a
        final value.

    `normalize_format`()[source]¶
    :   Return a YAML representation of the model, reformatted to to print
        lists and dicts using the flow style (no [], no {}) instead of
        the block style, especially for syntax checking where rules
        are defined only for flow style.

    `parse`(*description*)[source]¶
    :   Build the EmulsionModel from the specified dictionary
        (expected to come from a YAML configuration file).

    `set_value`(*name*, *value*)[source]¶
    :   Naif method to change a parameter’s value.
        Will be more efficient when treating several parameters at
        the same time.

    `write_dot`(*parent\_dir*)[source]¶
    :   Write the graph of the each state machine in the
        specified directer name, according to the dot/graphviz format.

## emulsion.model.exceptions module¶

*Module author: Sébastien Picault <sebastien.picault@inra.fr>*

*exception* `emulsion.model.exceptions.``SemanticException`(*message*)[source]¶
:   Bases: `Exception`

    Exception raised when a semantic error occurs during model parsing.

    `__init__`(*message*)[source]¶
    :   Initialize self. See help(type(self)) for accurate signature.

## emulsion.model.functions module¶

*Module author: Sébastien Picault <sebastien.picault@inra.fr>*

`emulsion.model.functions.``make_duration_condition`(*model*, *machine\_name*)[source]¶
:   Build a duration condition associated to the specified state
    machine and add it to the model. A condition duration, which is
    intended to specify when an agent is allowed to leave the current
    state of the state machine, is of the form ‘step >=
    \_time\_to\_exit\_MACHINE\_NAME’, each of those variables being stored
    in the state variables.

`emulsion.model.functions.``make_duration_init_action`(*agent*, *duration*, *machine\_name=None*, *\*\*\_*)[source]¶
:   Action that initializes the ‘time to exit’ for the state of the
    specified state\_machine.

`emulsion.model.functions.``make_function`(*expression, dtype=<class 'float'>, modules=['numpy', 'numpy.random', 'math', 'sympy']*)[source]¶
:   Transform the specified sympy expression into a function of an
    agent, which substitutes the specified symbols of the expression
    with an access to either attributes or state variables of the same
    name in the agent (through the `get_information` method) and
    returns a value of the specified dtype. The transformation uses
    the lambdify sympy function for better performances, with the
    specified modules.

`emulsion.model.functions.``make_when_condition`(*expression, dtype=<class 'bool'>, modules=['numpy', 'numpy.random', 'math', 'sympy']*)[source]¶
:   Transform the specified sympy expression into a function of an
    agent, which substitutes the specified symbol of the expression
    with an access to the simulation calendar. The transformation uses
    the lambdify sympy function for better performances, with the
    specified modules.

## emulsion.model.state\_machines module¶

*Module author: Sébastien Picault <sebastien.picault@inra.fr>*

*class* `emulsion.model.state_machines.``StateMachine`(*machine\_name*, *description*, *model*)[source]¶
:   Bases: `object`

    Class in charge of the description of biological or economical
    processes, modeled as Finite State Machines. The formalism
    implemented here is based on UML state machine diagrams, with
    adaptations to biology.

    `__init__`(*machine\_name*, *description*, *model*)[source]¶
    :   Build a State Machine within the specified model, based on
        the specified description (dictionary).

    `build_actions`()[source]¶
    :   Parse the description of the state machine and extract the
        actions that agents running this state machine must have.

        actions:
        :   say\_hello:
            :   desc: action performed when entering the S state

    `build_graph`()[source]¶
    :   Parse the description of the state machine and extract the
        graph of the transitions between the states. Since a
        MultiDiGraph is used, each pair of nodes can be bound by
        several transitions if needed (beware the associated
        semantics).

        transitions:
        :   - {from: S, to: I-, proba: p, cond: not\_vaccinated}
            - {from: I-, to: S, proba: m}
            - {from: I-, to: I+m, proba: ‘q\*plp’}
            - {from: I-, to: I+, proba: ‘q\*(1-plp)’}

    `build_states`()[source]¶
    :   Parse the description of the state machine and extract the existing
        states. States are described as list items, endowed with
        key-value properties. Only one state per list item is allowed
        (to ensure that states are always stored in the same order in
        all executions).

    `describe_state`(*name*)[source]¶
    :   Return the description of the state with the specified
        name.

    `get_default_state`(*caller=None*)[source]¶
    :   Return the default state for this state machine.

    `get_property`(*state\_name*, *property\_name*)[source]¶
    :   Return the property associated to the specified state.

    `get_random_state`(*caller=None*)[source]¶
    :   Return a random state for this state machine.

    `get_value`(*name*)[source]¶
    :   Return the value associated with the specified name.

    `parse`(*description*)[source]¶
    :   Build the State Machine from the specified dictionary
        (expected to come from a YAML configuration file).

    `state_colors`¶
    :   Return a dictionary of state names associated with fill colors.

    `write_dot`(*filename*, *view\_actions=True*)[source]¶
    :   Write the graph of the current state machine in the
        specified filename, according to the dot/graphviz format.

# EMULSION

Epidemiological Multi-Level Simulation Framework

### Navigation

- 1. Installation
- 2. Getting started with EMULSION
- 3. Modelling principles
- 4. Modelling language (basics)
- 5. Modelling language (advanced)
- 6. Feature examples
- 7. Information
- 8. License
- 9. High-level functions for model designers
- 10. emulsion package
  - 10.1. Subpackages
    - emulsion.agent package
    - emulsion.model package
    - emulsion.tools package
  - 10.2. Submodules
  - 10.3. emulsion.init\_emulsion module

### Related Topics

- Documentation overview
  - 10. emulsion package
    - Previous: emulsion.agent.managers package
    - Next: emulsion.tools package

### Quick search

©2016, INRA and Univ. Lille.
|
Powered by Sphinx 1.8.5
& Alabaster 0.7.10
|
Page source
