# Supplementary material for: EMULSION: Transparent and flexible multiscale stochastic models in human, animal and plant epidemiology
Source: PLoS Comput Biol. 2019 Sep 13;15(9):e1007342. doi: 10.1371/journal.pcbi.1007342 (PMC6760811; doi:10.1371/journal.pcbi.1007342)
Supplement: S2 File — This zip file is a clone of EMULSION public Git repository. To install from this file rather than from PyPI, go to EMULSION documentation page: https://sourcesup.renater.fr/www/emulsion-public/pages/Install.html and follow Git-based installation instructions. (ZIP) [file pcbi.1007342.s010.zip › S2_file/doc/genindex.html]

Index — EMULSION (Epidemiological Multi-Level Simulation framework)

# Index

**\_**
| **A**
| **B**
| **C**
| **D**
| **E**
| **F**
| **G**
| **H**
| **I**
| **J**
| **L**
| **M**
| **N**
| **O**
| **P**
| **R**
| **S**
| **T**
| **U**
| **V**
| **W**

## \_

- \_\_init\_\_() (emulsion.agent.action.AbstractAction method)
  - (emulsion.agent.action.BecomeAction method)
  - (emulsion.agent.action.CloneAction method)
  - (emulsion.agent.action.FunctionAction method)
  - (emulsion.agent.action.InvalidActionException method)
  - (emulsion.agent.action.MethodAction method)
  - (emulsion.agent.action.RateAdditiveAction method)
  - (emulsion.agent.action.RateDecreaseAction method)
  - (emulsion.agent.action.RateIncreaseAction method)
  - (emulsion.agent.action.RecordChangeAction method)
  - (emulsion.agent.action.SetVarAction method)
  - (emulsion.agent.action.StochAdditiveAction method)
  - (emulsion.agent.action.StochDecreaseAction method)
  - (emulsion.agent.action.StochIncreaseAction method)
  - (emulsion.agent.action.StringAction method)
  - (emulsion.agent.action.ValueAction method)
  - (emulsion.agent.atoms.AtomAgent method)
  - (emulsion.agent.atoms.EvolvingAtom method)
  - (emulsion.agent.comparts.Compartment method)
  - (emulsion.agent.core.abstract\_agent.AbstractAgent method), [1], [2]
  - (emulsion.agent.core.emulsion\_agent.EmulsionAgent method)
  - (emulsion.agent.core.groups.Aggregation method)
  - (emulsion.agent.core.groups.GroupAgent method)
  - (emulsion.agent.exceptions.InvalidCompartmentOperation method)
  - (emulsion.agent.exceptions.LevelException method)
  - (emulsion.agent.exceptions.StateVarNotFoundException method)
  - (emulsion.agent.managers.abstract\_process\_manager.AbstractProcessManager method)
  - (emulsion.agent.managers.group\_manager.GroupManager method)
  - (emulsion.agent.managers.ibm\_process\_manager.IBMProcessManager method)
  - (emulsion.agent.managers.multi\_process\_manager.MultiProcessManager method)
  - (emulsion.agent.process.AbstractProcess method)
  - (emulsion.agent.process.MethodProcess method)
  - (emulsion.agent.process.StateMachineProcess method)
  - (emulsion.agent.views.AdaptiveView method)
  - (emulsion.agent.views.AutoStructuredView method)
  - (emulsion.agent.views.SimpleView method)
  - (emulsion.agent.views.StructuredView method)
  - (emulsion.agent.views.StructuredViewWithCounts method)
  - (emulsion.model.emulsion\_model.EmulsionModel method), [1]
  - (emulsion.model.exceptions.SemanticException method)
  - (emulsion.model.state\_machines.StateMachine method)
  - (emulsion.tools.calendar.EventCalendar method)
  - (emulsion.tools.calendar.InvalidIntervalException method)
  - (emulsion.tools.graph.MultiDiGraph method)
  - (emulsion.tools.simulation.AbstractSimulation method)
  - (emulsion.tools.simulation.MultiSimulation method)
  - (emulsion.tools.simulation.OutputManager method)
  - (emulsion.tools.simulation.SensitivitySimulation method)
  - (emulsion.tools.simulation.Simulation method)
  - (emulsion.tools.state.StateVarDict method)

## A

|  |  |
| --- | --- |
| - AbstractAction (class in emulsion.agent.action) - AbstractAgent (class in emulsion.agent.core.abstract\_agent), [1], [2] - AbstractProcess (class in emulsion.agent.process) - AbstractProcessManager (class in emulsion.agent.managers.abstract\_process\_manager) - AbstractSimulation (class in emulsion.tools.simulation) - AdaptiveView (class in emulsion.agent.views) - add() (emulsion.agent.comparts.Compartment method)   - (emulsion.agent.core.groups.Aggregation method)   - (emulsion.agent.core.groups.GroupAgent method)   - (emulsion.agent.views.AutoStructuredView method)   - (emulsion.agent.views.SimpleView method)   - (emulsion.agent.views.StructuredView method) - add\_all\_test\_properties() (in module emulsion.tools.misc) - add\_atoms() (emulsion.agent.managers.ibm\_process\_manager.IBMProcessManager method)   - (emulsion.agent.managers.multi\_process\_manager.MultiProcessManager method) - add\_compart\_process() (emulsion.agent.managers.abstract\_process\_manager.AbstractProcessManager method)   - (emulsion.agent.managers.compart\_process\_manager.CompartProcessManager method)   - (emulsion.agent.managers.multi\_process\_manager.MultiProcessManager method) - add\_edge() (emulsion.tools.graph.MultiDiGraph method) - add\_event() (emulsion.tools.calendar.EventCalendar method) - add\_expression() (emulsion.model.emulsion\_model.EmulsionModel method), [1] - add\_host() (emulsion.agent.atoms.AtomAgent method)   - (emulsion.agent.managers.compart\_process\_manager.CompartProcessManager method)   - (emulsion.agent.managers.multi\_process\_manager.MultiProcessManager method) - add\_inbox() (emulsion.agent.core.emulsion\_agent.EmulsionAgent method) - add\_init\_action() (emulsion.model.emulsion\_model.EmulsionModel method), [1] - add\_method\_process() (emulsion.agent.atoms.EvolvingAtom method)   - (emulsion.agent.managers.abstract\_process\_manager.AbstractProcessManager method) - add\_new\_population() (emulsion.agent.managers.abstract\_process\_manager.AbstractProcessManager method)   - (emulsion.agent.managers.compart\_process\_manager.CompartProcessManager method)   - (emulsion.agent.managers.multi\_process\_manager.MultiProcessManager method) - add\_new\_property() (in module emulsion.tools.misc) - add\_node() (emulsion.tools.graph.MultiDiGraph method) - add\_outbox() (emulsion.agent.core.emulsion\_agent.EmulsionAgent method) - add\_population() (emulsion.agent.managers.compart\_process\_manager.CompartProcessManager method) - add\_statemachine\_process() (emulsion.agent.managers.abstract\_process\_manager.AbstractProcessManager method) - agcount (emulsion.agent.atoms.AtomAgent attribute)   - (emulsion.agent.atoms.EvolvingAtom attribute)   - (emulsion.agent.comparts.Compartment attribute)   - (emulsion.agent.core.abstract\_agent.AbstractAgent attribute)   - (emulsion.agent.core.emulsion\_agent.EmulsionAgent attribute)   - (emulsion.agent.core.groups.Aggregation attribute)   - (emulsion.agent.core.groups.GroupAgent attribute)   - (emulsion.agent.managers.abstract\_process\_manager.AbstractProcessManager attribute)   - (emulsion.agent.managers.compart\_process\_manager.CompartProcessManager attribute)   - (emulsion.agent.managers.group\_manager.GroupManager attribute)   - (emulsion.agent.managers.ibm\_process\_manager.IBMProcessManager attribute)   - (emulsion.agent.managers.metapop\_process\_manager.MetapopProcessManager attribute)   - (emulsion.agent.managers.multi\_process\_manager.MultiProcessManager attribute)   - (emulsion.agent.views.AdaptiveView attribute)   - (emulsion.agent.views.AutoStructuredView attribute)   - (emulsion.agent.views.SimpleView attribute)   - (emulsion.agent.views.StructuredView attribute)   - (emulsion.agent.views.StructuredViewWithCounts attribute) | - agdict (emulsion.agent.atoms.AtomAgent attribute)   - (emulsion.agent.atoms.EvolvingAtom attribute)   - (emulsion.agent.comparts.Compartment attribute)   - (emulsion.agent.core.abstract\_agent.AbstractAgent attribute)   - (emulsion.agent.core.emulsion\_agent.EmulsionAgent attribute)   - (emulsion.agent.core.groups.Aggregation attribute)   - (emulsion.agent.core.groups.GroupAgent attribute)   - (emulsion.agent.managers.abstract\_process\_manager.AbstractProcessManager attribute)   - (emulsion.agent.managers.compart\_process\_manager.CompartProcessManager attribute)   - (emulsion.agent.managers.group\_manager.GroupManager attribute)   - (emulsion.agent.managers.ibm\_process\_manager.IBMProcessManager attribute)   - (emulsion.agent.managers.metapop\_process\_manager.MetapopProcessManager attribute)   - (emulsion.agent.managers.multi\_process\_manager.MultiProcessManager attribute)   - (emulsion.agent.views.AdaptiveView attribute)   - (emulsion.agent.views.AutoStructuredView attribute)   - (emulsion.agent.views.SimpleView attribute)   - (emulsion.agent.views.StructuredView attribute)   - (emulsion.agent.views.StructuredViewWithCounts attribute) - AGENTS (in module emulsion.tools.misc) - aggregate\_probabilities() (in module emulsion.tools.misc), [1] - aggregate\_probability() (in module emulsion.tools.misc), [1] - Aggregation (class in emulsion.agent.core.groups) - AND() (in module emulsion.tools.functions), [1] - apply\_changes() (emulsion.agent.managers.group\_manager.GroupManager method) - apply\_initial\_conditions() (emulsion.agent.managers.abstract\_process\_manager.AbstractProcessManager method)   - (emulsion.agent.managers.compart\_process\_manager.CompartProcessManager method)   - (emulsion.agent.managers.multi\_process\_manager.MultiProcessManager method) - apply\_initial\_prototype() (emulsion.agent.core.abstract\_agent.AbstractAgent method), [1], [2]   - (emulsion.agent.managers.abstract\_process\_manager.AbstractProcessManager method) - apply\_prototype() (emulsion.agent.core.abstract\_agent.AbstractAgent method), [1], [2], [3] - AtomAgent (class in emulsion.agent.atoms) - AutoStructuredView (class in emulsion.agent.views) |

## B

|  |  |
| --- | --- |
| - BecomeAction (class in emulsion.agent.action) - build\_action() (emulsion.agent.action.AbstractAction class method) - build\_actions() (emulsion.model.emulsion\_model.EmulsionModel method), [1]   - (emulsion.model.state\_machines.StateMachine method) - build\_animation() (in module emulsion.tools.view) - build\_calendar() (emulsion.model.emulsion\_model.EmulsionModel method), [1] - build\_compartment\_desc() (emulsion.model.emulsion\_model.EmulsionModel method), [1] - build\_distributions() (emulsion.model.emulsion\_model.EmulsionModel method), [1] - build\_graph() (emulsion.model.state\_machines.StateMachine method) - build\_initial\_conds() (emulsion.model.emulsion\_model.EmulsionModel method), [1] | - build\_levels() (emulsion.model.emulsion\_model.EmulsionModel method), [1] - build\_machine\_plot() (in module emulsion.tools.plot) - build\_outputs\_options() (emulsion.model.emulsion\_model.EmulsionModel method), [1] - build\_parameters() (emulsion.model.emulsion\_model.EmulsionModel method), [1] - build\_processes() (emulsion.model.emulsion\_model.EmulsionModel method), [1] - build\_prototypes() (emulsion.model.emulsion\_model.EmulsionModel method), [1] - build\_state\_machines() (emulsion.model.emulsion\_model.EmulsionModel method), [1] - build\_state\_plot() (in module emulsion.tools.plot) - build\_states() (emulsion.model.state\_machines.StateMachine method) - build\_statevars() (emulsion.model.emulsion\_model.EmulsionModel method), [1] - build\_timeinfo() (emulsion.model.emulsion\_model.EmulsionModel method), [1] |

## C

|  |  |
| --- | --- |
| - calculate\_compound\_params() (emulsion.model.emulsion\_model.EmulsionModel method), [1] - change\_parameter\_values() (emulsion.model.emulsion\_model.EmulsionModel method), [1] - change\_state() (emulsion.agent.core.abstract\_agent.AbstractAgent method), [1], [2] - check\_consistency() (emulsion.agent.views.AdaptiveView method) - check\_state\_machines() (emulsion.model.emulsion\_model.EmulsionModel method), [1] - checkout\_inbox() (emulsion.agent.core.emulsion\_agent.EmulsionAgent method) - clean\_inbox() (emulsion.agent.core.emulsion\_agent.EmulsionAgent method) - clone() (emulsion.agent.atoms.AtomAgent method)   - (emulsion.agent.comparts.Compartment method)   - (emulsion.agent.views.AdaptiveView method) - CloneAction (class in emulsion.agent.action) - Compartment (class in emulsion.agent.comparts) - CompartProcessManager (class in emulsion.agent.managers.compart\_process\_manager) - complement\_population() (emulsion.agent.managers.compart\_process\_manager.CompartProcessManager method) - compute\_values() (emulsion.model.emulsion\_model.EmulsionModel method), [1] - copy() (emulsion.model.emulsion\_model.EmulsionModel method), [1] - count\_population() (in module emulsion.tools.misc) | - counts (emulsion.agent.managers.abstract\_process\_manager.AbstractProcessManager attribute)   - (emulsion.agent.managers.compart\_process\_manager.CompartProcessManager attribute)   - (emulsion.agent.managers.ibm\_process\_manager.IBMProcessManager attribute)   - (emulsion.agent.managers.metapop\_process\_manager.MetapopProcessManager attribute)   - (emulsion.tools.simulation.MultiSimulation attribute)   - (emulsion.tools.simulation.SensitivitySimulation attribute)   - (emulsion.tools.simulation.Simulation attribute) - create\_aggregator() (in module emulsion.tools.misc) - create\_atoms\_aggregator() (in module emulsion.tools.misc) - create\_count\_properties\_for\_state() (emulsion.agent.managers.abstract\_process\_manager.AbstractProcessManager method) - create\_counter\_getter() (in module emulsion.tools.misc) - create\_duration\_getter() (in module emulsion.tools.misc) - create\_group\_aggregator() (in module emulsion.tools.misc) - create\_new\_serial() (in module emulsion.tools.misc) - create\_population\_getter() (in module emulsion.tools.misc) - create\_properties\_for\_groups() (emulsion.agent.managers.abstract\_process\_manager.AbstractProcessManager method) - create\_state\_tester() (in module emulsion.tools.misc) - create\_weighted\_random() (in module emulsion.tools.misc) |

## D

|  |  |
| --- | --- |
| - date\_in() (in module emulsion.tools.calendar) - date\_to\_step() (emulsion.tools.calendar.EventCalendar method) - delta\_t (emulsion.agent.core.abstract\_agent.AbstractAgent attribute), [1], [2] - describe\_name() (emulsion.model.emulsion\_model.EmulsionModel method), [1] - describe\_parameter() (emulsion.model.emulsion\_model.EmulsionModel method), [1], [2] | - describe\_state() (emulsion.model.state\_machines.StateMachine method) - describe\_variable() (emulsion.model.emulsion\_model.EmulsionModel method), [1] - die() (emulsion.agent.core.abstract\_agent.AbstractAgent method), [1], [2] - do\_edge\_actions() (emulsion.agent.core.emulsion\_agent.EmulsionAgent method) - do\_state\_actions() (emulsion.agent.core.emulsion\_agent.EmulsionAgent method) - duration\_in\_current\_state() (emulsion.agent.core.abstract\_agent.AbstractAgent method), [1], [2], [3] |

## E

|  |  |
| --- | --- |
| - edges() (emulsion.tools.graph.MultiDiGraph method) - edges\_from() (emulsion.tools.graph.MultiDiGraph method) - EdgeTypes (class in emulsion.tools.graph) - emulsion (module), [1] - emulsion.agent (module), [1] - emulsion.agent.action (module) - emulsion.agent.atoms (module) - emulsion.agent.comparts (module) - emulsion.agent.core (module), [1] - emulsion.agent.core.abstract\_agent (module) - emulsion.agent.core.asbtract\_agent (module) - emulsion.agent.core.emulsion\_agent (module), [1] - emulsion.agent.core.groups (module), [1] - emulsion.agent.exceptions (module) - emulsion.agent.managers (module), [1] - emulsion.agent.managers.abstract\_process\_manager (module) - emulsion.agent.managers.compart\_process\_manager (module) - emulsion.agent.managers.functions (module), [1], [2], [3], [4], [5], [6], [7] - emulsion.agent.managers.group\_manager (module) - emulsion.agent.managers.ibm\_process\_manager (module) - emulsion.agent.managers.metapop\_process\_manager (module) - emulsion.agent.managers.multi\_process\_manager (module) - emulsion.agent.meta (module) - emulsion.agent.process (module) - emulsion.agent.views (module) - emulsion.init\_emulsion (module) - emulsion.model (module), [1] - emulsion.model.emulsion\_model (module), [1] - emulsion.model.exceptions (module), [1] - emulsion.model.functions (module), [1] - emulsion.model.state\_machines (module), [1] - emulsion.tools (module) - emulsion.tools.calendar (module), [1] - emulsion.tools.functions (module), [1] - emulsion.tools.graph (module) - emulsion.tools.misc (module), [1] - emulsion.tools.parallel (module) - emulsion.tools.plot (module) - emulsion.tools.simulation (module), [1] - emulsion.tools.state (module), [1] | - emulsion.tools.timing (module) - emulsion.tools.view (module) - EmulsionAgent (class in emulsion.agent.core.emulsion\_agent) - EmulsionEnum (class in emulsion.tools.state) - EmulsionModel (class in emulsion.model.emulsion\_model), [1] - evaluate\_condition() (emulsion.agent.core.emulsion\_agent.EmulsionAgent method)   - (emulsion.agent.views.AdaptiveView method) - evaluate\_event() (emulsion.agent.core.emulsion\_agent.EmulsionAgent method) - EventCalendar (class in emulsion.tools.calendar) - evolve() (emulsion.agent.atoms.EvolvingAtom method)   - (emulsion.agent.core.emulsion\_agent.EmulsionAgent method)   - (emulsion.agent.core.groups.Aggregation method)   - (emulsion.agent.managers.abstract\_process\_manager.AbstractProcessManager method)   - (emulsion.agent.managers.group\_manager.GroupManager method)   - (emulsion.agent.managers.ibm\_process\_manager.IBMProcessManager method)   - (emulsion.agent.process.AbstractProcess method)   - (emulsion.agent.process.MethodProcess method)   - (emulsion.agent.process.StateMachineProcess method)   - (emulsion.agent.views.AdaptiveView method)   - (emulsion.agent.views.StructuredView method)   - (emulsion.agent.views.StructuredViewWithCounts method)   - (emulsion.tools.simulation.AbstractSimulation method)   - (emulsion.tools.simulation.MultiSimulation method)   - (emulsion.tools.simulation.Simulation method) - evolve\_productions() (emulsion.agent.process.StateMachineProcess method) - evolve\_states() (emulsion.agent.atoms.EvolvingAtom method)   - (emulsion.agent.managers.group\_manager.GroupManager method) - evolve\_transitions() (emulsion.agent.process.StateMachineProcess method) - EvolvingAtom (class in emulsion.agent.atoms) - execute\_action() (emulsion.agent.action.AbstractAction method)   - (emulsion.agent.action.BecomeAction method)   - (emulsion.agent.action.CloneAction method)   - (emulsion.agent.action.FunctionAction method)   - (emulsion.agent.action.LogVarsAction method)   - (emulsion.agent.action.MessageAction method)   - (emulsion.agent.action.MethodAction method)   - (emulsion.agent.action.RateAdditiveAction method)   - (emulsion.agent.action.RecordChangeAction method)   - (emulsion.agent.action.SetVarAction method)   - (emulsion.agent.action.StochAdditiveAction method) - expand\_expression() (emulsion.model.emulsion\_model.EmulsionModel method), [1] |

## F

|  |  |
| --- | --- |
| - families (emulsion.agent.atoms.AtomAgent attribute)   - (emulsion.agent.atoms.EvolvingAtom attribute)   - (emulsion.agent.comparts.Compartment attribute)   - (emulsion.agent.core.abstract\_agent.AbstractAgent attribute)   - (emulsion.agent.core.emulsion\_agent.EmulsionAgent attribute)   - (emulsion.agent.core.groups.Aggregation attribute)   - (emulsion.agent.core.groups.GroupAgent attribute)   - (emulsion.agent.managers.abstract\_process\_manager.AbstractProcessManager attribute)   - (emulsion.agent.managers.compart\_process\_manager.CompartProcessManager attribute)   - (emulsion.agent.managers.group\_manager.GroupManager attribute)   - (emulsion.agent.managers.ibm\_process\_manager.IBMProcessManager attribute)   - (emulsion.agent.managers.metapop\_process\_manager.MetapopProcessManager attribute)   - (emulsion.agent.managers.multi\_process\_manager.MultiProcessManager attribute)   - (emulsion.agent.views.AdaptiveView attribute)   - (emulsion.agent.views.AutoStructuredView attribute)   - (emulsion.agent.views.SimpleView attribute)   - (emulsion.agent.views.StructuredView attribute)   - (emulsion.agent.views.StructuredViewWithCounts attribute) | - finalize\_level() (emulsion.agent.managers.abstract\_process\_manager.AbstractProcessManager method) - find\_operator() (in module emulsion.tools.misc) - find\_sublevel\_statemachines() (emulsion.agent.managers.ibm\_process\_manager.IBMProcessManager method) - from\_dict() (emulsion.agent.core.abstract\_agent.AbstractAgent class method), [1], [2] - FunctionAction (class in emulsion.agent.action) |

## G

|  |  |
| --- | --- |
| - generate\_skeleton() (emulsion.model.emulsion\_model.EmulsionModel method), [1] - get\_agent\_class\_for\_level() (emulsion.model.emulsion\_model.EmulsionModel method) - get\_agent\_class\_for\_sublevel() (emulsion.agent.managers.multi\_process\_manager.MultiProcessManager method) - get\_calendar\_for\_event() (emulsion.model.emulsion\_model.EmulsionModel method), [1] - get\_content() (emulsion.agent.atoms.AtomAgent method)   - (emulsion.agent.comparts.Compartment method)   - (emulsion.agent.core.emulsion\_agent.EmulsionAgent method)   - (emulsion.agent.views.SimpleView method)   - (emulsion.agent.views.StructuredView method) - get\_default\_state() (emulsion.model.state\_machines.StateMachine method) - get\_default\_sublevel() (emulsion.agent.managers.multi\_process\_manager.MultiProcessManager method) - get\_events() (emulsion.tools.calendar.EventCalendar method) - get\_group\_atoms() (emulsion.agent.managers.multi\_process\_manager.MultiProcessManager method) - get\_group\_population() (emulsion.agent.managers.abstract\_process\_manager.AbstractProcessManager method) - get\_host() (emulsion.agent.atoms.AtomAgent method)   - (emulsion.agent.core.emulsion\_agent.EmulsionAgent method) | - get\_information() (emulsion.agent.core.abstract\_agent.AbstractAgent method), [1], [2] - get\_machine() (emulsion.agent.atoms.EvolvingAtom method) - get\_model\_value() (emulsion.agent.core.abstract\_agent.AbstractAgent method), [1], [2] - get\_modifiable\_parameters() (emulsion.model.emulsion\_model.EmulsionModel method), [1], [2] - get\_or\_build() (emulsion.agent.views.StructuredView method) - get\_outbox() (emulsion.agent.core.emulsion\_agent.EmulsionAgent method) - get\_populations() (emulsion.agent.managers.metapop\_process\_manager.MetapopProcessManager method) - get\_property() (emulsion.model.state\_machines.StateMachine method) - get\_prototype() (emulsion.model.emulsion\_model.EmulsionModel method), [1] - get\_random\_state() (emulsion.model.state\_machines.StateMachine method) - get\_sublevels() (emulsion.agent.managers.ibm\_process\_manager.IBMProcessManager method) - get\_value() (emulsion.model.emulsion\_model.EmulsionModel method), [1]   - (emulsion.model.state\_machines.StateMachine method) - group\_and\_split\_populations() (in module emulsion.agent.managers.functions) - GroupAgent (class in emulsion.agent.core.groups) - GroupManager (class in emulsion.agent.managers.group\_manager) |

## H

- handle\_notifications() (emulsion.agent.views.StructuredView method)

## I

|  |  |
| --- | --- |
| - IBMProcessManager (class in emulsion.agent.managers.ibm\_process\_manager) - IfThenElse() (in module emulsion.tools.functions), [1] - increment() (emulsion.tools.calendar.EventCalendar method) - init\_agent() (emulsion.tools.simulation.Simulation method) - init\_counts() (emulsion.agent.managers.group\_manager.GroupManager method)   - (emulsion.agent.views.StructuredViewWithCounts method) - init\_level\_processes() (emulsion.agent.atoms.EvolvingAtom method) | - init\_processes() (emulsion.agent.managers.abstract\_process\_manager.AbstractProcessManager method) - init\_time\_entered() (emulsion.agent.core.abstract\_agent.AbstractAgent method), [1], [2] - initialize\_level() (emulsion.agent.managers.abstract\_process\_manager.AbstractProcessManager method) - InvalidActionException - InvalidCompartmentOperation - InvalidIntervalException - is\_in\_state() (emulsion.agent.core.emulsion\_agent.EmulsionAgent method) |

## J

|  |  |
| --- | --- |
| - job() (in module emulsion.tools.parallel) | - job\_dist() (in module emulsion.tools.parallel) |

## L

|  |  |
| --- | --- |
| - LevelException - linestyle (emulsion.tools.graph.EdgeTypes attribute) - load\_class() (in module emulsion.tools.misc) - load\_module() (in module emulsion.tools.misc) | - load\_state\_from\_file() (emulsion.agent.managers.abstract\_process\_manager.AbstractProcessManager method) - log\_path() (emulsion.agent.core.emulsion\_agent.EmulsionAgent method)   - (emulsion.tools.simulation.Simulation method) - LogVarsAction (class in emulsion.agent.action) |

## M

|  |  |
| --- | --- |
| - main() (in module emulsion.init\_emulsion) - make\_all\_consistent() (emulsion.agent.managers.multi\_process\_manager.MultiProcessManager method) - make\_consistent() (emulsion.agent.managers.multi\_process\_manager.MultiProcessManager method) - make\_duration\_condition() (in module emulsion.model.functions) - make\_duration\_init\_action() (in module emulsion.model.functions) - make\_function() (in module emulsion.model.functions) - make\_when\_condition() (in module emulsion.model.functions) - members (emulsion.agent.atoms.AtomAgent attribute)   - (emulsion.agent.atoms.EvolvingAtom attribute)   - (emulsion.agent.comparts.Compartment attribute)   - (emulsion.agent.core.abstract\_agent.AbstractAgent attribute)   - (emulsion.agent.core.emulsion\_agent.EmulsionAgent attribute)   - (emulsion.agent.core.groups.Aggregation attribute)   - (emulsion.agent.core.groups.GroupAgent attribute)   - (emulsion.agent.managers.abstract\_process\_manager.AbstractProcessManager attribute)   - (emulsion.agent.managers.compart\_process\_manager.CompartProcessManager attribute)   - (emulsion.agent.managers.group\_manager.GroupManager attribute)   - (emulsion.agent.managers.ibm\_process\_manager.IBMProcessManager attribute)   - (emulsion.agent.managers.metapop\_process\_manager.MetapopProcessManager attribute)   - (emulsion.agent.managers.multi\_process\_manager.MultiProcessManager attribute)   - (emulsion.agent.views.AdaptiveView attribute)   - (emulsion.agent.views.AutoStructuredView attribute)   - (emulsion.agent.views.SimpleView attribute)   - (emulsion.agent.views.StructuredView attribute)   - (emulsion.agent.views.StructuredViewWithCounts attribute) | - MessageAction (class in emulsion.agent.action) - MetaAgent (class in emulsion.agent.meta) - MetapopProcessManager (class in emulsion.agent.managers.metapop\_process\_manager) - MethodAction (class in emulsion.agent.action) - MethodProcess (class in emulsion.agent.process) - move\_to() (emulsion.agent.comparts.Compartment method)   - (emulsion.agent.core.groups.GroupAgent method) - moving\_average() (in module emulsion.tools.misc), [1] - MultiDiGraph (class in emulsion.tools.graph) - MultiProcessManager (class in emulsion.agent.managers.multi\_process\_manager) - MultiSimulation (class in emulsion.tools.simulation) |

## N

|  |  |
| --- | --- |
| - name (emulsion.agent.core.emulsion\_agent.EmulsionAgent attribute) - new\_atom() (emulsion.agent.managers.multi\_process\_manager.MultiProcessManager method) - next\_states() (emulsion.agent.comparts.Compartment method)   - (emulsion.agent.views.AdaptiveView method)   - (emulsion.agent.views.SimpleView method) | - next\_states\_from() (emulsion.agent.core.emulsion\_agent.EmulsionAgent method) - normalize\_format() (emulsion.model.emulsion\_model.EmulsionModel method), [1] - notify\_changed\_units() (emulsion.agent.views.StructuredView method) |

## O

|  |  |
| --- | --- |
| - OR() (in module emulsion.tools.functions), [1] | - OutputManager (class in emulsion.tools.simulation) |

## P

|  |  |
| --- | --- |
| - parallel\_multi() (in module emulsion.tools.parallel) - parallel\_sensi() (in module emulsion.tools.parallel) - parse() (emulsion.model.emulsion\_model.EmulsionModel method), [1]   - (emulsion.model.state\_machines.StateMachine method) - plot\_outputs() (in module emulsion.tools.plot) - population (emulsion.agent.comparts.Compartment attribute)   - (emulsion.agent.core.groups.Aggregation attribute)   - (emulsion.agent.managers.abstract\_process\_manager.AbstractProcessManager attribute) | - POPULATION (in module emulsion.tools.misc) - probabilities\_to\_rates() (in module emulsion.tools.misc), [1] - PRODUCTION (emulsion.tools.graph.EdgeTypes attribute) - production\_from() (emulsion.agent.core.emulsion\_agent.EmulsionAgent method) |

## R

|  |  |
| --- | --- |
| - random\_beta() (in module emulsion.tools.functions), [1] - random\_bool() (in module emulsion.tools.functions), [1] - random\_choice() (in module emulsion.tools.functions), [1] - random\_choice\_weighted() (in module emulsion.tools.functions), [1] - random\_exponential() (in module emulsion.tools.functions), [1] - random\_gamma() (in module emulsion.tools.functions), [1] - random\_integers() (in module emulsion.tools.functions), [1] - random\_multinomial() (in module emulsion.tools.functions), [1] - random\_normal() (in module emulsion.tools.functions), [1] - random\_poisson() (in module emulsion.tools.functions), [1] - random\_uniform() (in module emulsion.tools.functions), [1] - RateAdditiveAction (class in emulsion.agent.action) - RateDecreaseAction (class in emulsion.agent.action) - RateIncreaseAction (class in emulsion.agent.action) - rates\_to\_probabilities() (in module emulsion.tools.misc), [1] - read\_from\_file() (in module emulsion.tools.misc) - RecordChangeAction (class in emulsion.agent.action) - remove() (emulsion.agent.comparts.Compartment method)   - (emulsion.agent.core.groups.Aggregation method)   - (emulsion.agent.core.groups.GroupAgent method)   - (emulsion.agent.managers.abstract\_process\_manager.AbstractProcessManager method)   - (emulsion.agent.managers.compart\_process\_manager.CompartProcessManager method)   - (emulsion.agent.managers.multi\_process\_manager.MultiProcessManager method)   - (emulsion.agent.views.AutoStructuredView method)   - (emulsion.agent.views.SimpleView method)   - (emulsion.agent.views.StructuredView method) | - remove\_atoms() (emulsion.agent.managers.multi\_process\_manager.MultiProcessManager method) - remove\_host() (emulsion.agent.atoms.AtomAgent method) - remove\_population() (emulsion.agent.managers.compart\_process\_manager.CompartProcessManager method) - remove\_randomly() (emulsion.agent.managers.abstract\_process\_manager.AbstractProcessManager method)   - (emulsion.agent.managers.compart\_process\_manager.CompartProcessManager method)   - (emulsion.agent.managers.ibm\_process\_manager.IBMProcessManager method)   - (emulsion.agent.managers.multi\_process\_manager.MultiProcessManager method) - reset\_outbox() (emulsion.agent.core.emulsion\_agent.EmulsionAgent method) - retrieve\_value() (in module emulsion.tools.misc) - rewrite\_keys() (in module emulsion.tools.misc) - run() (emulsion.tools.simulation.AbstractSimulation method)   - (emulsion.tools.simulation.MultiSimulation method)   - (emulsion.tools.simulation.SensitivitySimulation method)   - (emulsion.tools.simulation.Simulation method) |

## S

|  |  |
| --- | --- |
| - save\_state\_to\_file() (emulsion.agent.managers.abstract\_process\_manager.AbstractProcessManager method) - select\_atoms() (emulsion.agent.managers.multi\_process\_manager.MultiProcessManager method) - select\_random() (in module emulsion.tools.misc), [1] - select\_randomly() (emulsion.agent.managers.multi\_process\_manager.MultiProcessManager method) - SemanticException - SensitivitySimulation (class in emulsion.tools.simulation) - serial() (in module emulsion.tools.misc) - set\_information() (emulsion.agent.core.abstract\_agent.AbstractAgent method), [1], [2] - set\_statemachines() (emulsion.agent.atoms.EvolvingAtom method) - set\_value() (emulsion.model.emulsion\_model.EmulsionModel method), [1] - SetVarAction (class in emulsion.agent.action) - show\_contour() (in module emulsion.tools.view) - show\_histo() (in module emulsion.tools.view) - show\_img() (in module emulsion.tools.view) | - SimpleView (class in emulsion.agent.views) - Simulation (class in emulsion.tools.simulation) - state\_colors (emulsion.model.state\_machines.StateMachine attribute) - StateMachine (class in emulsion.model.state\_machines) - StateMachineProcess (class in emulsion.agent.process) - StateVarDict (class in emulsion.tools.state) - StateVarNotFoundException - step\_to\_date() (emulsion.tools.calendar.EventCalendar method) - StochAdditiveAction (class in emulsion.agent.action) - StochDecreaseAction (class in emulsion.agent.action) - StochIncreaseAction (class in emulsion.agent.action) - StringAction (class in emulsion.agent.action) - StructuredView (class in emulsion.agent.views) - StructuredViewWithCounts (class in emulsion.agent.views) |

## T

|  |  |
| --- | --- |
| - time (emulsion.agent.core.abstract\_agent.AbstractAgent attribute), [1], [2] - timethis() (in module emulsion.tools.timing) | - top\_level() (emulsion.agent.core.emulsion\_agent.EmulsionAgent method) - TRANSITION (emulsion.tools.graph.EdgeTypes attribute) |

## U

|  |  |
| --- | --- |
| - update\_counts() (emulsion.agent.managers.group\_manager.GroupManager method)   - (emulsion.agent.managers.ibm\_process\_manager.IBMProcessManager method)   - (emulsion.agent.views.StructuredViewWithCounts method) - update\_csv\_counts() (emulsion.tools.simulation.AbstractSimulation method) | - update\_output\_information() (emulsion.tools.simulation.OutputManager method) - update\_output\_type() (emulsion.tools.simulation.OutputManager method) - update\_outputs() (emulsion.tools.simulation.OutputManager method) - update\_time\_to\_exit() (emulsion.agent.core.abstract\_agent.AbstractAgent method), [1], [2], [3] - upper\_level() (emulsion.agent.core.emulsion\_agent.EmulsionAgent method) |

## V

- ValueAction (class in emulsion.agent.action)

## W

- write\_dot() (emulsion.model.emulsion\_model.EmulsionModel method), [1]
  - (emulsion.model.state\_machines.StateMachine method)
  - (emulsion.tools.simulation.MultiSimulation method)
  - (emulsion.tools.simulation.SensitivitySimulation method)

# EMULSION

Epidemiological Multi-Level Simulation Framework

### Navigation

- 1. Installation
- 2. Getting started with EMULSION
- 3. Modelling principles
- 4. Modelling language (basics)
- 5. Modelling language (advanced)
- 6. Feature examples
- 7. Information
- 8. License
- 9. High-level functions for model designers
- 10. emulsion package

### Related Topics

- Documentation overview

### Quick search

©2016, INRA and Univ. Lille.
|
Powered by Sphinx 1.8.5
& Alabaster 0.7.10
