# Supplementary material for: EMULSION: Transparent and flexible multiscale stochastic models in human, animal and plant epidemiology
Source: PLoS Comput Biol. 2019 Sep 13;15(9):e1007342. doi: 10.1371/journal.pcbi.1007342 (PMC6760811; doi:10.1371/journal.pcbi.1007342)
Supplement: S2 File — This zip file is a clone of EMULSION public Git repository. To install from this file rather than from PyPI, go to EMULSION documentation page: https://sourcesup.renater.fr/www/emulsion-public/pages/Install.html and follow Git-based installation instructions. (ZIP) [file pcbi.1007342.s010.zip › S2_file/doc/emulsion.html]

10. emulsion package — EMULSION (Epidemiological Multi-Level Simulation framework)

# 10. emulsion package¶

EMULSION: Epidemiologic Multi-Level Simulation framework

## 10.1. Subpackages¶

- emulsion.agent package
  - Subpackages
    - emulsion.agent.core package
      - Submodules
      - emulsion.agent.core.abstract\_agent module
      - emulsion.agent.core.emulsion\_agent module
      - emulsion.agent.core.groups module
    - emulsion.agent.managers package
      - Submodules
      - emulsion.agent.managers.abstract\_process\_manager module
      - emulsion.agent.managers.compart\_process\_manager module
      - emulsion.agent.managers.functions module
      - emulsion.agent.managers.group\_manager module
      - emulsion.agent.managers.ibm\_process\_manager module
      - emulsion.agent.managers.metapop\_process\_manager module
      - emulsion.agent.managers.multi\_process\_manager module
  - Submodules
  - emulsion.agent.action module
  - emulsion.agent.atoms module
  - emulsion.agent.comparts module
  - emulsion.agent.exceptions module
  - emulsion.agent.meta module
  - emulsion.agent.process module
  - emulsion.agent.views module
- emulsion.model package
  - Submodules
  - emulsion.model.emulsion\_model module
  - emulsion.model.exceptions module
  - emulsion.model.functions module
  - emulsion.model.state\_machines module
- emulsion.tools package
  - Submodules
  - emulsion.tools.calendar module
  - emulsion.tools.functions module
  - emulsion.tools.graph module
  - emulsion.tools.misc module
  - emulsion.tools.parallel module
  - emulsion.tools.plot module
  - emulsion.tools.simulation module
  - emulsion.tools.state module
  - emulsion.tools.timing module
  - emulsion.tools.view module

## 10.2. Submodules¶

## 10.3. emulsion.init\_emulsion module¶

`emulsion.init_emulsion.``main`()[source]¶

# EMULSION

Epidemiological Multi-Level Simulation Framework

### Navigation

- 1. Installation
- 2. Getting started with EMULSION
- 3. Modelling principles
- 4. Modelling language (basics)
- 5. Modelling language (advanced)
- 6. Feature examples
- 7. Information
- 8. License
- 9. High-level functions for model designers
- 10. emulsion package
  - 10.1. Subpackages
    - emulsion.agent package
    - emulsion.model package
    - emulsion.tools package
  - 10.2. Submodules
  - 10.3. emulsion.init\_emulsion module

### Related Topics

- Documentation overview
  - Previous: 9. High-level functions for model designers
  - Next: emulsion.agent package

### Quick search

©2016, INRA and Univ. Lille.
|
Powered by Sphinx 1.8.5
& Alabaster 0.7.10
|
Page source
