# Supplementary material for: EMULSION: Transparent and flexible multiscale stochastic models in human, animal and plant epidemiology
Source: PLoS Comput Biol. 2019 Sep 13;15(9):e1007342. doi: 10.1371/journal.pcbi.1007342 (PMC6760811; doi:10.1371/journal.pcbi.1007342)
Supplement: S2 File — This zip file is a clone of EMULSION public Git repository. To install from this file rather than from PyPI, go to EMULSION documentation page: https://sourcesup.renater.fr/www/emulsion-public/pages/Install.html and follow Git-based installation instructions. (ZIP) [file pcbi.1007342.s010.zip › S2_file/doc/emulsion.agent.core.html]

emulsion.agent.core package — EMULSION (Epidemiological Multi-Level Simulation framework)

# emulsion.agent.core package¶

*Module author: Sébastien Picault <sebastien.picault@inra.fr>*

Part of this code was adapted from the PADAWAN framework (S. Picault,
Univ. Lille).

## Submodules¶

## emulsion.agent.core.abstract\_agent module¶

*Module author: Sébastien Picault <sebastien.picault@inra.fr>*

Part of this code was adapted from the PADAWAN framework (S. Picault,
Univ. Lille).

*class* `emulsion.agent.core.abstract_agent.``AbstractAgent`(*envt=None*, *content=None*, *\*\*others*)[source]¶
:   Bases: `object`

    The Superclass for any multi-level agent. Due to the MetaAgent
    metaclass, all agents of the same class can be accessed through
    their ID, using the agdict class attribute. Agents are endowed
    with an automatic ID and possibly with a label. Agents also belong
    to families which can be chosen arbitrarily. By default, each
    agent belongs to the family named afer its own class.

    An agent is situated in one or more environments. Besides, agents
    can encapsulate environments where other agents can be
    situated. Agents are also endowed with State Variables, which can
    represent either properties of their own, or properties perceived
    from their inner environment or from the environments where they
    are situated.

    `__init__`(*envt=None*, *content=None*, *\*\*others*)[source]¶
    :   Instantiate an agent. The instance is automatically added
        to the agentset of its own class. An arbitrary label can be
        specified to give the agent a label (otherwise a label is
        automatically computed using the class name and the agent
        ID).

    `agcount` *= 0*¶

    `agdict` *= {}*¶

    `apply_initial_prototype`(*name=None*, *prototype=None*, *execute\_actions=False*)[source]¶
    :   Apply prototype to a newly created agent. This function is called
        by new\_atom`in `MultiProcessManager and redefined to do nothing in
        this class, in order to ensure that the prototype of a level is
        applied before creating entities for the sublevels.

    `apply_prototype`(*name=None*, *prototype=None*, *execute\_actions=False*)[source]¶
    :   Apply the prototype with the specified name to this agent. A
        prototype is basically a dictionary of statevars associated to
        values. Thus this method changes specific statevars to new
        values as required in the prototype. Other statevars are kept
        unchanged. If a statevar was no amongst those of this agent,
        it is added. When statevars hold the state of a state machine,
        the state is changed with correct initialization of
        corresponding attributes (mainly \_time\_entered) ; if
        execute\_actions is True, on\_enter/on\_exit actions are
        performed.

        TAG: USER

    `change_state`(*machine\_name*, *new\_state*, *do\_actions=False*)[source]¶
    :   Change the state of this agent for the specified state machine to
        new\_state. The \_time\_entered\_MACHINE value for this state machine
        is initialized. If do\_actions is True, perform the actions to
        do on\_exit from the previous state (if any) and those to do
        on\_enter in the new state (if any).

        TAG: USER?

    `delta_t`¶
    :   A shortcut to self.model.delta\_t.

    `die`()[source]¶
    :   Operation performed when the agent is removed from the
        simulation. Recursively destroy agents contained in the
        current agent if any, and remove the current agent from the
        agdict attribute of its class.

    `duration_in_current_state`(*machine\_name: str*) → float[source]¶
    :   Return the duration (in time units) this agent has spent in the
        current state of the specified state machine (*machine\_name*).

        |  |  |
        | --- | --- |
        | Parameters: | **machine\_name** – the name of the state machine for which the duration in the current state must be computed |
        | Returns: | A duration (expressed in time units) corresponding to the duration spent in the current state for this state machine. |

    `families` *= SortedSet(['AbstractAgent'], key=None, load=1000)*¶

    *classmethod* `from_dict`(*dct*)[source]¶
    :   Instantiate an agent using the specified dictionary.

        TAG: USER

    `get_information`(*name*)[source]¶
    :   Return the value corresponding to the specified name in the
        agent. This value can be stored either as an attribute or a
        property-like descriptor (and thus accessed through an
        attribute-like syntax), or as a State Variable using a
        StateVarDict attribute named `statevars`.

        Example:
        class Cow(AtomAgent):

        > …
        > @property
        > def age(self):
        >
        > > return self.\_age

        c = Cow()
        c.get\_information(‘age’)
        # -> access through property ‘age’
        c.get\_information(‘health\_state’)
        # -> access through statevar ‘health\_state’ (present in any Unit),
        # unless a ‘health\_state’ attribute or property is explicitly
        # redefined to override the state variable

        TAG: USER

    `get_model_value`(*name*)[source]¶
    :   Return the value corresponding to the specified name in the model
        of the agent. If the name refers to a function, apply this
        function to the agent.

        TAG: USER

    `init_time_entered`(*machine\_name*, *advance=0*, *nb\_timesteps=0*)[source]¶
    :   Initialize the time step value when this agent is entering a new
        state of the specified state machine. A (positive) advance
        value can be provided to mimick an earlier entering in the
        state. By default, a \_time\_to\_exit value is set according to
        the value of nb\_timesteps.

    `members` *= ('agcount', 'agdict', 'families', '\_\_module\_\_', '\_\_qualname\_\_', '\_\_doc\_\_', 'from\_dict', '\_register\_instance', '\_\_init\_\_', '\_reset\_mbr\_cache', '\_\_hash\_\_', '\_\_len\_\_', '\_\_str\_\_', '\_\_repr\_\_', '\_\_eq\_\_', '\_\_lt\_\_', 'get\_model\_value', 'delta\_t', 'time', 'die', 'get\_information', 'set\_information', 'init\_time\_entered', 'change\_state', 'update\_time\_to\_exit', 'duration\_in\_current\_state', 'apply\_prototype', 'apply\_initial\_prototype', '\_\_classcell\_\_')*¶

    `set_information`(*name*, *value*)[source]¶
    :   Set the specified value for the statevar/attribute.

        TAG: USER

    `time`¶
    :   The time elapsed since the beginning of simulation (in time
        units).

    `update_time_to_exit`(*machine\_name*, *duration*)[source]¶
    :   Update the *duration* this agent is expected to stay in the current
        state of the specified state machine (*machine\_name*).

        The time step value after which the agent is allowed to leave
        the current state is stored in a statevar called
        `_time_to_exit_machine_name`, is computed from the
        current time (`step`) plus the *duration*.

        |  |  |
        | --- | --- |
        | Parameters: | - **machine\_name** (*str*) – name of the state machine for which   the duration in the current state must be updated - **duration** (*datetime.timedelta*) – the new duration for the   agent to stay in the current state, calculated from the   current time in the simulation |

## emulsion.agent.core.emulsion\_agent module¶

*Module author: Sébastien Picault <sebastien.picault@inra.fr>*

Part of this code was adapted from the PADAWAN framework (S. Picault,
Univ. Lille).

*class* `emulsion.agent.core.emulsion_agent.``EmulsionAgent`(*model=None*, *level=None*, *name=None*, *host=None*, *simulation=None*, *\*\*others*)[source]¶
:   Bases: `emulsion.agent.core.abstract_agent.AbstractAgent`

    The EmulsionAgent is the base class for multi-level
    epidemiologic agents. An EmulsionAgent can represent any entity
    involved in the epidemiologic model (individual, compartment,
    herd, [meta]population, etc.).

    Each agent contains an exchange box (in & out) which is composed
    of a list of messages. A message is generally a dictionary of
    Source/Destination (In/Out) and content of exchange with
    Source/Destination agent.

    `__init__`(*model=None*, *level=None*, *name=None*, *host=None*, *simulation=None*, *\*\*others*)[source]¶
    :   Initialize the unit with a health state and a name.

    `add_inbox`(*messages=[]*)[source]¶
    :   Add the specified list of messages in the inbox.

        TAG: USER

    `add_outbox`(*message*)[source]¶
    :   Appendd a message to the outbox.

        TAG: USER

    `agcount` *= 0*¶

    `agdict` *= {}*¶

    `checkout_inbox`()[source]¶
    :   Pick up agent’s inbox content.

        TAG: USER

    `clean_inbox`()[source]¶
    :   Remove non sticky messages from the inbox.

        TAG: USER

    `do_edge_actions`(*actions=None*, *population=None*, *agents=None*)[source]¶
    :   Perform the actions associated to a transition between
        states. If the unit is a ViewCompartment, actions are actually
        performed by each unit of the specified agents list, in
        turn. Otherwise, the actions are performed according to the
        population, which is expected to be a number.

    `do_state_actions`(*event*, *state\_machine*, *state\_name*, *population=None*, *agents=None*, *\*\*\_*)[source]¶
    :   Perform the actions associated to the current state. If the
        unit is a ViewAgent, actions are actually performed by each
        unit of the specified agents list, in turn. Otherwise, the
        actions are performed according to the population, which is
        expected to be a number.

    `evaluate_condition`(*condition*)[source]¶
    :   Return the content (tuple either (‘population’, qty) or
        (‘agents’, list)) if the condition is fulfilled, () otherwise.

    `evaluate_event`(*name*)[source]¶
    :   Evaluate if the specified event name is fulfilled, using a
        calendar. The calendar is determined dynamically according to
        the name of the event.

        TAG: USER

    `evolve`(*machine=None*)[source]¶
    :   This method is aimed at defining what has systematically to
        be done in the unit at each time step (e.g. age change…). It
        has to be overriden if needed in subclasses.

    `families` *= SortedSet(['EmulsionAgent'], key=None, load=1000)*¶

    `get_content`()[source]¶
    :   Return either the population (number) of the current unit,
        or the list of agents contained in the current unit. The
        output is a tuple with either (‘population’, qty) or (‘agents’, list).

    `get_host`(*key=None*)[source]¶
    :   Return the host of the current unit.

        TAG: USER

    `get_outbox`()[source]¶
    :   Return the content of the outbox.

        TAG: USER

    `is_in_state`(*state\_name*)[source]¶
    :   Evaluate if the agent is in the specified state. The state name is
        expected to be a valid state of one of the state machines. If
        so, the name of the state machine is expected to be one of the
        statevars of the agent.

    `log_path`()[source]¶
    :   Return the path to the log file for the current simulation

    `members` *= ('agcount', 'agdict', 'families', '\_\_module\_\_', '\_\_qualname\_\_', '\_\_doc\_\_', '\_\_init\_\_', 'upper\_level', 'top\_level', 'log\_path', 'name', '\_\_repr\_\_', 'evolve', 'get\_host', 'get\_content', 'do\_state\_actions', 'do\_edge\_actions', 'next\_states\_from', 'production\_from', '\_compute\_production', '\_compute\_values\_for\_unique\_population', '\_compute\_values\_for\_multiple\_populations', 'evaluate\_condition', 'evaluate\_event', 'is\_in\_state', 'get\_outbox', 'add\_outbox', 'reset\_outbox', 'add\_inbox', 'clean\_inbox', 'checkout\_inbox', '\_\_classcell\_\_')*¶

    `name`¶
    :   Return the name of the unit. If no name was provided during
        instantiation, the class name is returned.

        TAG: USER

    `next_states_from`(*initial\_state*, *state\_machine*)[source]¶
    :   Return a list of tuples composed of:

        - each of the possible states reachable from the specified

        initial state (some depending on a condition)

        - the transition rate, probability or amount to each state in

        the specified state\_machine (possible clauses: ‘rate’,
        ‘proba’, ‘amount’, ‘amount-all-but’ with the actual value)

        - a tuple indicating who can cross the edge, depending on

        conditions (either (‘population’, qty) or (‘agents’, list))

        - the list of actions to perform when crossing the edge (if

        any).

        The conditions, rates and probabilities may depend on the state
        variables or properties of the current unit.

    `production_from`(*initial\_state*, *state\_machine*)[source]¶
    :   Return a list of tuples composed of:

        - each of the possible states that can be produced from the specified

        initial state (some depending on a condition)

        - the transition rate, probability or amount to each state in

        the specified state\_machine (possible clauses: ‘rate’,
        ‘proba’, ‘amount’, ‘amount-all-but’ with the actual value)

        - a tuple indicating who can produce cross the edge, depending on

        conditions (either (‘population’, qty) or (‘agents’, list))

        - the prototypes of agents produced through the edge (if

        any).

        The conditions, rates and probabilities may depend on the state
        variables or properties of the current unit.

    `reset_outbox`()[source]¶
    :   Reset the content of the outbox.

        TAG: USER

    `top_level`()[source]¶
    :   Return the highest level used in the simulation

    `upper_level`(*init=True*)[source]¶
    :   Return the ‘upper level’ for this agent, i.e. the first host with
        a not-None level attribute.

        TAG: USER

## emulsion.agent.core.groups module¶

*Module author: Sébastien Picault <sebastien.picault@inra.fr>*

Part of this code was adapted from the PADAWAN framework (S. Picault,
Univ. Lille).

*class* `emulsion.agent.core.groups.``Aggregation`(*recursive=True*, *\*\*others*)[source]¶
:   Bases: `emulsion.agent.core.groups.GroupAgent`

    An Aggregation is aimed at grouping agents from the underlying
    level. Thus, aggregate information such as the total number of
    agents in the compartment (population) is calculated from the
    actual content of the compartment. The evolution of a
    ViewCompartment, by default, consists in making the units
    contained in the compartment evolve themselves, unless
    recursive=False is specified during instantiation.

    `__init__`(*recursive=True*, *\*\*others*)[source]¶
    :   Initialize the unit with a health state and a name.

    `add`(*population*)[source]¶
    :   Add the specified population to the current compartment.

    `agcount` *= 0*¶

    `agdict` *= {}*¶

    `evolve`(*machine=None*)[source]¶
    :   Ask each unit in the current compartment to make its content evolve
        according to its own capabilities. A specific state machine
        can be specified if needed.

    `families` *= SortedSet(['Aggregation'], key=None, load=1000)*¶

    `members` *= ('agcount', 'agdict', 'families', '\_\_module\_\_', '\_\_qualname\_\_', '\_\_doc\_\_', '\_\_init\_\_', 'population', 'add', 'remove', '\_base\_move', '\_\_iter\_\_', 'evolve', '\_\_classcell\_\_')*¶

    `population`¶
    :   Return the total population of the compartment. It is
        calculated either using a true ‘population’ statevar if any,
        or as the sum of the population of each unit contained in the
        compartment.

        TAG: USER

    `remove`(*population*)[source]¶
    :   Remove the specified population from the current
        compartment.

*class* `emulsion.agent.core.groups.``GroupAgent`(*keys=()*, *\*\*others*)[source]¶
:   Bases: `emulsion.agent.core.emulsion_agent.EmulsionAgent`

    A GroupAgent is aimed at representing a group of agents. The
    underlying level may be either explicitly represented (using a
    ViewCompartment) or aggregated (using an Compartment). Each
    compartment is associated with keys, i.e. a tuple of state
    variables (possibly empty) which play a crucial role in this
    compartment.

    `__init__`(*keys=()*, *\*\*others*)[source]¶
    :   Initialize the unit with a health state and a name.

    `add`(*population*)[source]¶
    :   Add the specified population to the current compartment.

    `agcount` *= 0*¶

    `agdict` *= {}*¶

    `families` *= SortedSet(['GroupAgent'], key=None, load=1000)*¶

    `members` *= ('agcount', 'agdict', 'families', '\_\_module\_\_', '\_\_qualname\_\_', '\_\_doc\_\_', '\_\_init\_\_', 'add', 'remove', '\_base\_move', '\_before\_move', '\_after\_move', 'move\_to', '\_\_classcell\_\_')*¶

    `move_to`(*other\_unit*, *state\_machine=None*, *\*\*others*)[source]¶
    :   Move the specified population from the current population
        of the compartment to the other unit. If a state machine is
        provided, executes the corresponding actions when
        entering/exiting nodes and crossing edges if needed.

    `remove`(*population*)[source]¶
    :   Remove the specified population from the current
        compartment.

# EMULSION

Epidemiological Multi-Level Simulation Framework

### Navigation

- 1. Installation
- 2. Getting started with EMULSION
- 3. Modelling principles
- 4. Modelling language (basics)
- 5. Modelling language (advanced)
- 6. Feature examples
- 7. Information
- 8. License
- 9. High-level functions for model designers
- 10. emulsion package
  - 10.1. Subpackages
    - emulsion.agent package
    - emulsion.model package
    - emulsion.tools package
  - 10.2. Submodules
  - 10.3. emulsion.init\_emulsion module

### Related Topics

- Documentation overview
  - 10. emulsion package
    - emulsion.agent package
      - Previous: emulsion.agent package
      - Next: emulsion.agent.managers package

### Quick search

©2016, INRA and Univ. Lille.
|
Powered by Sphinx 1.8.5
& Alabaster 0.7.10
|
Page source
