# Supplementary material for: EMULSION: Transparent and flexible multiscale stochastic models in human, animal and plant epidemiology
Source: PLoS Comput Biol. 2019 Sep 13;15(9):e1007342. doi: 10.1371/journal.pcbi.1007342 (PMC6760811; doi:10.1371/journal.pcbi.1007342)
Supplement: S2 File — This zip file is a clone of EMULSION public Git repository. To install from this file rather than from PyPI, go to EMULSION documentation page: https://sourcesup.renater.fr/www/emulsion-public/pages/Install.html and follow Git-based installation instructions. (ZIP) [file pcbi.1007342.s010.zip › S2_file/doc/emulsion_for_modellers.html]

9. High-level functions for model designers — EMULSION (Epidemiological Multi-Level Simulation framework)

# 9. High-level functions for model designers¶

## 9.1. Functions Available for Models¶

`emulsion.tools.functions.``AND`(*\*values*)[source]¶
:   Return a logical AND (conjunction) between all the values.

    Example

    To define symptomatic individuals as infected for at least 5 days:

    ```
    is_symptomatic:
      desc: 'test if individuals has symptoms'
      value: 'AND(is_I, duration_in_health_state >= 5)'
    ```

    |  |  |
    | --- | --- |
    | Parameters: | **\*values** (*list*) – boolean values separated by commas |
    | Returns: | *bool* – True if **all** values are True, False otherwise. |

`emulsion.tools.functions.``IfThenElse`(*condition*, *val\_if\_true*, *val\_if\_false*)[source]¶
:   Ternary conditional function: return either *val\_if\_true* or
    *val\_if\_false* depending on *condition*.

    Example

    Here in a parameter definition (assuming that `summer_period` is
    defined e.g. in the *calendars* section of the model):

    ```
    average_temperature:
      desc: 'average temperature for the season'
      value: 'IfThenElse(summer_period, 25, 8)'
    ```

    |  |  |
    | --- | --- |
    | Parameters: | - **condition** (*bool*) – a boolean expression - **val\_if\_true** (*number*) – value to return if the expression is True - **val\_if\_false** (*number*) – value to return if the expression is False |
    | Returns: | *number* – One of the two values, depending on *condition*. |

`emulsion.tools.functions.``OR`(*\*values*)[source]¶
:   Return a logical OR (disjunction) between all the values.

    Example

    To sell animals depending on either their weight or their age:

    ```
    transitions:
      ...
      - from: Fattening
        to: Sold
        proba: 1
        cond: 'OR(weight >= weight_thr, age >= age_thr)'
    ```

    |  |  |
    | --- | --- |
    | Parameters: | **\*values** (*list*) – boolean values separated by commas |
    | Returns: | *bool* – True if **at least one** of the values is True, False otherwise. |

`emulsion.tools.functions.``random_beta`(*a: float*, *b: float*) → float[source]¶
:   Return a random value drawn from a beta distribution of parameters
    *a* and *b*.

    Example

    In a prototype definition:

    ```
    age: 'random_beta(2, 5) * age_max'
    ```

    |  |  |
    | --- | --- |
    | Parameters: | - **a** (*float**,* *positive* *(**>0**)*) – first shape parameter of the beta distribution - **b** (*float**,* *positive* *(**>0**)*) – second shape parameter of the beta distribution |
    | Returns: | *float* – a random value sampled according to a beta distribution of parameters *a* and *b* |

    See also

    `numpy.random.beta()`

`emulsion.tools.functions.``random_bool`(*proba\_success: float*) → int[source]¶
:   Return a random boolean value (actually, 0 or 1) depending on
    *proba\_success*.

    Example

    Set the value of a state variable `has_symptoms` when entering
    in the infectious state:

    ```
    states:
      ...
      - I:
        ...
        on_enter:
          - set_var: has_symptoms
            value: 'random_bool(proba_symptomatic)'
    ```

    |  |  |
    | --- | --- |
    | Parameters: | **proba\_success** (*float in* *[**0**,**1**]*) – probability of returning 1 (True) |
    | Returns: | *int* – either 1 with probability *proba\_success*, or 0 with probability 1-*proba\_success* |

`emulsion.tools.functions.``random_choice`(*\*values*)[source]¶
:   Return a value chosen randomly among those provided (equiprobable
    sampling).

    Example

    To init `age` among three typical values:

    ```
    prototypes:
      individuals:
        init_individual:
          age: 'random_choice(10, 50, 200)'
    ```

    |  |  |
    | --- | --- |
    | Parameters: | **\*values** (*list*) – the possible values, separated by commas |
    | Returns: | *val* – one of the values (equiprobable choice) |

`emulsion.tools.functions.``random_choice_weighted`(*\*values*)[source]¶
:   Return a value chosen randomly among those provided (but not
    equiprobably).

    Example

    To init `age` among three typical values which respectively
    represent 10%, 20% and 70% of the population:

    ```
    prototypes:
      individuals:
        init_individual:
          age: 'random_choice_weighted(10, 0.1, 50, 0.2, 200, 0.7)'
    ```

    |  |  |
    | --- | --- |
    | Parameters: | **values** (*list*) – possibles choices and their weight, alternatively, separated by commas. Weights are normalized to be used as probabilities. |
    | Returns: | *One of the choices* |

`emulsion.tools.functions.``random_exponential`(*scale: float*) → float[source]¶
:   Return a random value drawn from an exponential distribution of
    rate 1/*scale* (thus of mean *scale*).

    Example

    In a prototype definition:

    ```
    time_to_live: random_exponential(mean_duration)
    ```

    |  |  |
    | --- | --- |
    | Parameters: | **scale** (*float*) – the scale parameter of the distribution, i.e. the inverse of the rate |
    | Returns: | *float* – a random value sampled according to an exponential distribution of rate 1/*scale* |

    See also

    `numpy.random.exponential()`

`emulsion.tools.functions.``random_gamma`(*shape: float*, *scale: float*) → float[source]¶
:   Return a random value drawn from a gamma distribution of parameters
    *shape* and *scale*.

    Example

    In a prototype definition:

    ```
    age: 'random_gamma(3, 2)'
    ```

    |  |  |
    | --- | --- |
    | Parameters: | - **shape** (*float**,* *positive* *(**>0**)*) – the shape of the gamma distribution - **scale** (*float**,* *positive* *(**>0**)*) – the scale of the gamma distribution |
    | Returns: | *float* – a random value sampled according to a gamma distribution of parameters *shape* and *scale* |

    See also

    `numpy.random.gamma()`

`emulsion.tools.functions.``random_integers`(*low: int*, *high: int*) → int[source]¶
:   Return a random integer value drawn from a discrete uniform
    distribution between *low* and *high* (both inclusive).

    Example

    In a prototype definition:

    ```
    age: random_integers(min_age, max_age)
    ```

    |  |  |
    | --- | --- |
    | Parameters: | - **low** (*int*) – lower boundary of the sample interval (inclusive) - **high** (*int*) – upper boundary of the sample interval (inclusive) |
    | Returns: | *int* – a random integer value sampled according to a discrete uniform distribution between *low* and *high* |

    See also

    `numpy.random.random_integers()`

`emulsion.tools.functions.``random_multinomial`(*number\_of\_samples*, *\*probas*)[source]¶
:   Return a multinomial sample based on the specified probabilities.

    |  |  |
    | --- | --- |
    | Parameters: | - **number\_of\_samples** (*int*) – number of experiments - **probas** (*list*) – list of probabilities |
    | Returns: | *list* – the drawn samples |

`emulsion.tools.functions.``random_normal`(*mn: float*, *sd: float*) → float[source]¶
:   Return a random value drawn from a normal distribution of mean *mn*
    and standard deviation *sd*.

    Example

    In a prototype definition:

    ```
    age: 'random_normal(100, 5)'
    ```

    |  |  |
    | --- | --- |
    | Parameters: | - **mn** (*float*) – the mean of the normal distribution - **sd** (*float**,* *positive* *(**>=0**)*) – the standard deviation of the normal distribution |
    | Returns: | *float* – a random value sampled according to a normal distribution of mean *mn* and standard deviation *sd* |

    See also

    `numpy.random.normal()`

`emulsion.tools.functions.``random_poisson`(*lam: float*) → int[source]¶
:   Return an integer random value drawn from a Poisson distribution of
    mean *lam*.

    Example

    In an action, e.g. here when computing how many newborn
    individuals will be produced:

    ```
    - from: Gestating
      to: NonGestating
      on_cross:
        - produce_offspring: newborn
          amount: 'random_poisson(1.05)'
    ```

    |  |  |
    | --- | --- |
    | Parameters: | **lam** (*float*) – the mean of the Poisson distribution |
    | Returns: | *int* – a random integer value sampled according to a Poisson distribution of mean *lam* |

    See also

    `numpy.random.poisson()`

`emulsion.tools.functions.``random_uniform`(*low: float*, *high: float*) → float[source]¶
:   Return a random value drawn from a uniform distribution between
    *low* (inclusive) and *high* (exclusive).

    Example

    In a prototype definition:

    ```
    age: random_uniform(min_age, max_age)
    ```

    |  |  |
    | --- | --- |
    | Parameters: | - **low** (*float*) – lower boundary of the sample interval (inclusive) - **high** (*float*) – upper boundary of the sample interval (exclusive) |
    | Returns: | *float* – a random value sampled according to a uniform distribution between *low* and *high* |

    See also

    `numpy.random.uniform()`

## 9.2. Rates / probabilities¶

`emulsion.tools.misc.``aggregate_probabilities`(*probability\_values: Iterable[float], delta\_t: float*) → Iterable[float][source]¶
:   From the specified *probability\_values*, intended to represent
    probabilities of events duting one time unit, compute the
    probabilities for the specified time step (*delta\_t*).

    |  |  |
    | --- | --- |
    | Parameters: | - **probability\_values** (*list*) – a list of probability values for several events (during 1 time unit) - **delta\_t** (*float*) – the value of the time step (expressed in time units) |
    | Returns: | *list* – the probabilities of the same events during *delta\_t* time units. |

`emulsion.tools.misc.``aggregate_probability`(*probability: float*, *delta\_t: float*) → float[source]¶
:   Transform the specified *probability* value, intended to represent
    a probability for events tested each time unit, into the
    probability for the specified time step (*delta\_t*).

    |  |  |
    | --- | --- |
    | Parameters: | - **probability** (*float*) – the probability value of an event (during 1 time unit) - **delta\_t** (*float*) – the value of the time step (expressed in time units) |
    | Returns: | *float* – the probability of the event during *delta\_t* time units. |

`emulsion.tools.misc.``probabilities_to_rates`(*probability\_values: Iterable[float]*) → List[float][source]¶
:   Transform a list of probabilities into a list of rates. The
    last value is expected to represent the probability of staying in
    the current state.

    |  |  |
    | --- | --- |
    | Parameters: | **probability\_values** (*list*) – a list of probabilities, the last one representing the probability to stay in the current state |
    | Returns: | *list* – a list of rates corresponding to those probabilities. |

`emulsion.tools.misc.``rates_to_probabilities`(*total\_rate: float, rate\_values: List[float], delta\_t: float = 1*) → List[float][source]¶
:   Transform the specified list of *rate\_values*, interpreted as
    outgoing rates, into probabilities, according to the specified
    time step (*delta\_t*) and normalized by *total\_rate*.

    For exit rates \(\rho\_i\) (one of the *rate\_values*), the
    probability to stay in current state is given by:

    \[p\_0 = e^{-\delta t.\sum\_i \rho\_i}\]

    Thus, each rate \(\rho\_i\) corresponds to a probability

    \[p\_i = \frac{\rho\_i}{\sum\_i \rho\_i} (1 - p\_0)\]

    |  |  |
    | --- | --- |
    | Parameters: | - **total\_rate** (*float*) – the total exit rate, used for normalization purposes. If   *rate\_values* represent all possible exit rates, *total\_rate*   is their sum. - **rate\_values** (*list*) – the list of rates to transform - **delta\_t** (*float*) – the value of the time step (expressed in time units) |
    | Returns: | *list* – the list of probabilities corresponding to the *rate\_values*. |

## 9.3. Computations¶

`emulsion.tools.misc.``moving_average`(*values*, *window\_size*, *mode='same'*)[source]¶
:   Compute a moving average of the specified *values* with respect to
    the *window\_size* on which the average is calculated. The return
    moving average has the same size as the original values. To avoid
    boundary effects, use `mode='valid'`, which produce a result of
    size `len(values) - window_size + 1`.

    |  |  |
    | --- | --- |
    | Parameters: | - **values** (*array-like*) – contains the values for moving average computation - **window\_size** (*int*) – width of the moving average window - **mode** (*str*) – a parameter for numpy.convolve |
    | Returns: | *nd\_array* – a numpy `nd_array` containing the values of the moving average. |

## 9.4. Selecting agents¶

`emulsion.tools.misc.``select_random`(*origin: Iterable*, *quantity: int*, *exclude: sortedcontainers.sortedset.SortedSet = SortedSet([]*, *key=None*, *load=1000)*) → List[source]¶
:   Return a random selection of *quantity* agents from the *origin*
    group, avoiding those explicitly in the *exclude* set. If the
    *origin* population proves too small, all available agents are
    taken, irrespective to the *quantity*.

    |  |  |
    | --- | --- |
    | Parameters: | - **origin** (*iterable*) – the population where agents must be selected - **quantity** (*int*) – the number of agents to select in the population - **exclude** (*set*) – agents which are not available for the selection |
    | Returns: | *list* – a list of randomly selected agents according to the above constraints. |

## 9.5. Durations¶

*class* `emulsion.agent.core.abstract_agent.``AbstractAgent`(*envt=None*, *content=None*, *\*\*others*)[source]¶
:   Bases: `object`

    The Superclass for any multi-level agent. Due to the MetaAgent
    metaclass, all agents of the same class can be accessed through
    their ID, using the agdict class attribute. Agents are endowed
    with an automatic ID and possibly with a label. Agents also belong
    to families which can be chosen arbitrarily. By default, each
    agent belongs to the family named afer its own class.

    An agent is situated in one or more environments. Besides, agents
    can encapsulate environments where other agents can be
    situated. Agents are also endowed with State Variables, which can
    represent either properties of their own, or properties perceived
    from their inner environment or from the environments where they
    are situated.

    `duration_in_current_state`(*machine\_name: str*) → float[source]¶
    :   Return the duration (in time units) this agent has spent in the
        current state of the specified state machine (*machine\_name*).

        |  |  |
        | --- | --- |
        | Parameters: | **machine\_name** – the name of the state machine for which the duration in the current state must be computed |
        | Returns: | A duration (expressed in time units) corresponding to the duration spent in the current state for this state machine. |

    `update_time_to_exit`(*machine\_name*, *duration*)[source]¶
    :   Update the *duration* this agent is expected to stay in the current
        state of the specified state machine (*machine\_name*).

        The time step value after which the agent is allowed to leave
        the current state is stored in a statevar called
        `_time_to_exit_machine_name`, is computed from the
        current time (`step`) plus the *duration*.

        |  |  |
        | --- | --- |
        | Parameters: | - **machine\_name** (*str*) – name of the state machine for which   the duration in the current state must be updated - **duration** (*datetime.timedelta*) – the new duration for the   agent to stay in the current state, calculated from the   current time in the simulation |

    `__init__`(*envt=None*, *content=None*, *\*\*others*)[source]¶
    :   Instantiate an agent. The instance is automatically added
        to the agentset of its own class. An arbitrary label can be
        specified to give the agent a label (otherwise a label is
        automatically computed using the class name and the agent
        ID).

    `apply_initial_prototype`(*name=None*, *prototype=None*, *execute\_actions=False*)[source]¶
    :   Apply prototype to a newly created agent. This function is called
        by new\_atom`in `MultiProcessManager and redefined to do nothing in
        this class, in order to ensure that the prototype of a level is
        applied before creating entities for the sublevels.

    `apply_prototype`(*name=None*, *prototype=None*, *execute\_actions=False*)[source]¶
    :   Apply the prototype with the specified name to this agent. A
        prototype is basically a dictionary of statevars associated to
        values. Thus this method changes specific statevars to new
        values as required in the prototype. Other statevars are kept
        unchanged. If a statevar was no amongst those of this agent,
        it is added. When statevars hold the state of a state machine,
        the state is changed with correct initialization of
        corresponding attributes (mainly \_time\_entered) ; if
        execute\_actions is True, on\_enter/on\_exit actions are
        performed.

        TAG: USER

    `change_state`(*machine\_name*, *new\_state*, *do\_actions=False*)[source]¶
    :   Change the state of this agent for the specified state machine to
        new\_state. The \_time\_entered\_MACHINE value for this state machine
        is initialized. If do\_actions is True, perform the actions to
        do on\_exit from the previous state (if any) and those to do
        on\_enter in the new state (if any).

        TAG: USER?

    `delta_t`¶
    :   A shortcut to self.model.delta\_t.

    `die`()[source]¶
    :   Operation performed when the agent is removed from the
        simulation. Recursively destroy agents contained in the
        current agent if any, and remove the current agent from the
        agdict attribute of its class.

    `duration_in_current_state`(*machine\_name: str*) → float[source]
    :   Return the duration (in time units) this agent has spent in the
        current state of the specified state machine (*machine\_name*).

        |  |  |
        | --- | --- |
        | Parameters: | **machine\_name** – the name of the state machine for which the duration in the current state must be computed |
        | Returns: | A duration (expressed in time units) corresponding to the duration spent in the current state for this state machine. |

    *classmethod* `from_dict`(*dct*)[source]¶
    :   Instantiate an agent using the specified dictionary.

        TAG: USER

    `get_information`(*name*)[source]¶
    :   Return the value corresponding to the specified name in the
        agent. This value can be stored either as an attribute or a
        property-like descriptor (and thus accessed through an
        attribute-like syntax), or as a State Variable using a
        StateVarDict attribute named `statevars`.

        Example:
        class Cow(AtomAgent):

        > …
        > @property
        > def age(self):
        >
        > > return self.\_age

        c = Cow()
        c.get\_information(‘age’)
        # -> access through property ‘age’
        c.get\_information(‘health\_state’)
        # -> access through statevar ‘health\_state’ (present in any Unit),
        # unless a ‘health\_state’ attribute or property is explicitly
        # redefined to override the state variable

        TAG: USER

    `get_model_value`(*name*)[source]¶
    :   Return the value corresponding to the specified name in the model
        of the agent. If the name refers to a function, apply this
        function to the agent.

        TAG: USER

    `init_time_entered`(*machine\_name*, *advance=0*, *nb\_timesteps=0*)[source]¶
    :   Initialize the time step value when this agent is entering a new
        state of the specified state machine. A (positive) advance
        value can be provided to mimick an earlier entering in the
        state. By default, a \_time\_to\_exit value is set according to
        the value of nb\_timesteps.

    `set_information`(*name*, *value*)[source]¶
    :   Set the specified value for the statevar/attribute.

        TAG: USER

    `time`¶
    :   The time elapsed since the beginning of simulation (in time
        units).

    `update_time_to_exit`(*machine\_name*, *duration*)[source]
    :   Update the *duration* this agent is expected to stay in the current
        state of the specified state machine (*machine\_name*).

        The time step value after which the agent is allowed to leave
        the current state is stored in a statevar called
        `_time_to_exit_machine_name`, is computed from the
        current time (`step`) plus the *duration*.

        |  |  |
        | --- | --- |
        | Parameters: | - **machine\_name** (*str*) – name of the state machine for which   the duration in the current state must be updated - **duration** (*datetime.timedelta*) – the new duration for the   agent to stay in the current state, calculated from the   current time in the simulation |

## 9.6. Agent State and Variable Changes¶

*class* `emulsion.agent.core.abstract_agent.``AbstractAgent`(*envt=None*, *content=None*, *\*\*others*)[source]
:   Bases: `object`

    The Superclass for any multi-level agent. Due to the MetaAgent
    metaclass, all agents of the same class can be accessed through
    their ID, using the agdict class attribute. Agents are endowed
    with an automatic ID and possibly with a label. Agents also belong
    to families which can be chosen arbitrarily. By default, each
    agent belongs to the family named afer its own class.

    An agent is situated in one or more environments. Besides, agents
    can encapsulate environments where other agents can be
    situated. Agents are also endowed with State Variables, which can
    represent either properties of their own, or properties perceived
    from their inner environment or from the environments where they
    are situated.

    `apply_prototype`(*name=None*, *prototype=None*, *execute\_actions=False*)[source]
    :   Apply the prototype with the specified name to this agent. A
        prototype is basically a dictionary of statevars associated to
        values. Thus this method changes specific statevars to new
        values as required in the prototype. Other statevars are kept
        unchanged. If a statevar was no amongst those of this agent,
        it is added. When statevars hold the state of a state machine,
        the state is changed with correct initialization of
        corresponding attributes (mainly \_time\_entered) ; if
        execute\_actions is True, on\_enter/on\_exit actions are
        performed.

        TAG: USER

    `__init__`(*envt=None*, *content=None*, *\*\*others*)[source]
    :   Instantiate an agent. The instance is automatically added
        to the agentset of its own class. An arbitrary label can be
        specified to give the agent a label (otherwise a label is
        automatically computed using the class name and the agent
        ID).

    `apply_initial_prototype`(*name=None*, *prototype=None*, *execute\_actions=False*)[source]
    :   Apply prototype to a newly created agent. This function is called
        by new\_atom`in `MultiProcessManager and redefined to do nothing in
        this class, in order to ensure that the prototype of a level is
        applied before creating entities for the sublevels.

    `apply_prototype`(*name=None*, *prototype=None*, *execute\_actions=False*)[source]
    :   Apply the prototype with the specified name to this agent. A
        prototype is basically a dictionary of statevars associated to
        values. Thus this method changes specific statevars to new
        values as required in the prototype. Other statevars are kept
        unchanged. If a statevar was no amongst those of this agent,
        it is added. When statevars hold the state of a state machine,
        the state is changed with correct initialization of
        corresponding attributes (mainly \_time\_entered) ; if
        execute\_actions is True, on\_enter/on\_exit actions are
        performed.

        TAG: USER

    `change_state`(*machine\_name*, *new\_state*, *do\_actions=False*)[source]
    :   Change the state of this agent for the specified state machine to
        new\_state. The \_time\_entered\_MACHINE value for this state machine
        is initialized. If do\_actions is True, perform the actions to
        do on\_exit from the previous state (if any) and those to do
        on\_enter in the new state (if any).

        TAG: USER?

    `delta_t`
    :   A shortcut to self.model.delta\_t.

    `die`()[source]
    :   Operation performed when the agent is removed from the
        simulation. Recursively destroy agents contained in the
        current agent if any, and remove the current agent from the
        agdict attribute of its class.

    `duration_in_current_state`(*machine\_name: str*) → float[source]
    :   Return the duration (in time units) this agent has spent in the
        current state of the specified state machine (*machine\_name*).

        |  |  |
        | --- | --- |
        | Parameters: | **machine\_name** – the name of the state machine for which the duration in the current state must be computed |
        | Returns: | A duration (expressed in time units) corresponding to the duration spent in the current state for this state machine. |

    *classmethod* `from_dict`(*dct*)[source]
    :   Instantiate an agent using the specified dictionary.

        TAG: USER

    `get_information`(*name*)[source]
    :   Return the value corresponding to the specified name in the
        agent. This value can be stored either as an attribute or a
        property-like descriptor (and thus accessed through an
        attribute-like syntax), or as a State Variable using a
        StateVarDict attribute named `statevars`.

        Example:
        class Cow(AtomAgent):

        > …
        > @property
        > def age(self):
        >
        > > return self.\_age

        c = Cow()
        c.get\_information(‘age’)
        # -> access through property ‘age’
        c.get\_information(‘health\_state’)
        # -> access through statevar ‘health\_state’ (present in any Unit),
        # unless a ‘health\_state’ attribute or property is explicitly
        # redefined to override the state variable

        TAG: USER

    `get_model_value`(*name*)[source]
    :   Return the value corresponding to the specified name in the model
        of the agent. If the name refers to a function, apply this
        function to the agent.

        TAG: USER

    `init_time_entered`(*machine\_name*, *advance=0*, *nb\_timesteps=0*)[source]
    :   Initialize the time step value when this agent is entering a new
        state of the specified state machine. A (positive) advance
        value can be provided to mimick an earlier entering in the
        state. By default, a \_time\_to\_exit value is set according to
        the value of nb\_timesteps.

    `set_information`(*name*, *value*)[source]
    :   Set the specified value for the statevar/attribute.

        TAG: USER

    `time`
    :   The time elapsed since the beginning of simulation (in time
        units).

    `update_time_to_exit`(*machine\_name*, *duration*)[source]
    :   Update the *duration* this agent is expected to stay in the current
        state of the specified state machine (*machine\_name*).

        The time step value after which the agent is allowed to leave
        the current state is stored in a statevar called
        `_time_to_exit_machine_name`, is computed from the
        current time (`step`) plus the *duration*.

        |  |  |
        | --- | --- |
        | Parameters: | - **machine\_name** (*str*) – name of the state machine for which   the duration in the current state must be updated - **duration** (*datetime.timedelta*) – the new duration for the   agent to stay in the current state, calculated from the   current time in the simulation |

## 9.7. Introspection¶

*class* `emulsion.model.emulsion_model.``EmulsionModel`(*filename=None*, *description=None*)[source]¶
:   Bases: `object`

    Class in charge of the description of a multi-level
    epidemiological model. Such models are composed of several
    processes which may take place at different
    organization/observation levels. Some of those processes are
    reified through a function implemented within an agent, while
    others are represented by a state machine. The model is also in
    charge of handling symbolic expressions and pre-computing their
    values, e.g. concerning parameters, transmission functions,
    etc.

    `describe_parameter`(*name*)[source]¶
    :   Return the description of the parameter with the specified
        name.

    `get_modifiable_parameters`()[source]¶
    :   Return a dictionary containing all true parameters with their
        value.

    `__init__`(*filename=None*, *description=None*)[source]¶
    :   Instantiate the model either from a configuration file or
        from a string. Both must contain a YAML-based description of
        the model.

    `add_expression`(*expression*)[source]¶
    :   Add the specified expression to the dictionary of known
        expressions.

    `add_init_action`(*machine\_name*, *state*, *action*)[source]¶
    :   Add an action to be performed when initializing agents for
        the specified state of the state machine. Mainly used for
        durations.

    `build_actions`()[source]¶
    :   Parse the description of the model and extract the actions
        that agents must have.

        actions:
        :   say\_hello:
            :   desc: action performed when entering the S state

    `build_calendar`(*calendar\_desc*)[source]¶
    :   Build a representation of calendars.

    `build_compartment_desc`()[source]¶
    :   Inspect the grouping part of the model (if
        any) in order to store the corresponding information.

    `build_distributions`()[source]¶
    :   Parse the description of the model and extract the
        distributions, either with their value, or with the expression
        to compute them. A distribution is a dictionary of the form
        {quantity: probability}. It is stored as a list of tuples
        (quantity, probability) which is more convenient for
        subsequent processing.

        distributions:
        :   - shedding\_dist1:
              :   desc: distribution of shedding
                  value:

                  > low\_shedding: 0.85
                  > med\_shedding: 0.15

    `build_initial_conds`()[source]¶
    :   Parse the description of the model and extract the initial
        conditions for each level, either with their value, of with
        the expression to compute them. An initial condition is a
        dictionary containing a description of the ‘content’ of the
        level (i.e. the quantity and initial state of sub-levels):
        - either the ‘population:’ keyword followed by a dict State -> Amount
        - or ‘prototype:’ and ‘amount:’ keywords followed by their values

        initial\_conditions:
        :   metapop:
            :   - prototype: healthy # defines init\_prevalence
                  amount: 100
                - prototype: sick
                  amount: 1

            herd:
            :   - population:
                  :   infection:
                      :   S: ‘init\_pop \* (1 - init\_prevalence)’
                          I: ‘init\_pop \* init\_prevalence’

    `build_levels`()[source]¶
    :   Parse the description of different level of simulations.
        Most of time, there are tree differents levels:
        individual, herd, metapopulation.

        levels:
        :   individuals:
            :   super:
                :   class: AtomAgent

                class\_name: Cow

            herd:
            :   super:
                :   class: MultiProcessManager

                class\_name: QfeverHerd

            metapop:
            :   super:
                :   class: MultiProcessManager
                    master\_class: StructuredView

                class\_name: QfeverMetaPop

    `build_outputs_options`()[source]¶
    :   Parse the outputs options of the model.
        The agents will treat extra variables for outputs (TODO), and
        the simulation classes will treat period outputs.

        outputs:
        :   # level
            herd:

            > period:1

            # level
            metapop:

            > period: 7
            > extra\_vars:
            >
            > > - step
            > > - infection\_date

    `build_parameters`()[source]¶
    :   Parse the description of the model and extract the
        parameters, either with their value, or with the expression to
        compute them.

        |  |  |
        | --- | --- |
        | Parameters: | - **p** – desc: infection probability   value: ‘(1-exp(-E\_total)) \* (1 - phi\*vaccinated)’ - **phi** – desc: efficiency of vaccination   value: 0.79 |

    `build_processes`()[source]¶
    :   Parse the description of the model and retrieve the list of
        processes with different level.

        processes:
        :   herd:
            :   - bacterial\_dispersion
                - culling\_process
                - infection

            metapop:
            :   - inbox\_distribution
                - outbox\_distribution

    `build_prototypes`()[source]¶
    :   Parse the description of the model and extract the list of
        prototypes for each level, either with their value, or with
        the expression to compute them. A prototype is a dictionary of
        the form {statevar: value}..

        prototypes:
        :   animals:
            :   - newborn:
                  :   desc: prototype for new calves
                      health\_state: M
                      life\_state: NP
                      age: 0

    `build_state_machines`()[source]¶
    :   Parse the description of the model and build all the
        required state machines.

    `build_statevars`()[source]¶
    :   Parse the description of the model and extract the state
        variables that agents running this model must have.

        statevars:
        :   E\_total:
            :   desc: total bacteria deposited in the environment

            vaccinated:
            :   desc: 0/1 value describing the vaccination state

    `build_timeinfo`()[source]¶
    :   Parse the description of the model and extract the
        information related to time management, i.e. time unit,
        duration of a simulation step, origin date, calendars for
        specific events, etc.

        time\_info:
        time\_unit: days
        delta\_t: 7
        origin: ‘May 1’
        calendar:

        > name:
        > period: {days: 52}
        > events:
        >
        > > spring: {begin: ‘April 8’, end: ‘April 23’}
        > > summer: {begin: ‘July 8’, end: ‘September 3’}
        > > national: {date: ‘July 14’}

    `calculate_compound_params`()[source]¶
    :   Inspect all edges of the health states graph and compute
        the actual probabilities associated to expressions.
        ### TODO: check that the ultimate symbols are declared properties.

    `change_parameter_values`(*changes*, *log\_params=False*)[source]¶
    :   Naive method to change several parameter values at the same time.

    `check_state_machines`()[source]¶
    :   Rapidly inspect the name of state machines and of their states to
        pre-register automatically built statevars.

    `compute_values`()[source]¶
    :   Check parameters and calculated compound parameters, so as
        to make them computable. In the case of parameters, number
        values are left unchanged, but strings (representing an
        expression) are replaced by a function. Regarding calculated
        compound parameters, expressions corresponding to numbers are
        converted to float values, while other expressions are
        replaced by a function.

    `copy`()[source]¶
    :   Return a copy of object (naif method).
        TODO: Find a way to avoid recharging compartment class when
        intentiate a MultiProcessManager class with a old model.

    `describe_name`(*name*)[source]¶
    :   Return the description of the specified name in the model
        name.

    `describe_parameter`(*name*)[source]
    :   Return the description of the parameter with the specified
        name.

    `describe_variable`(*name*)[source]¶
    :   Return the description of the statevar with the specified
        name.

    `expand_expression`(*expression*)[source]¶
    :   Transform the specified expression so as to replace all
        parameters by actual values or state variables or
        attributes.

    `generate_skeleton`(*module*)[source]¶
    :   Output a code skeleton to help writing specific pieces of code for
        the specified module to make the model work under Emulsion.

    `get_calendar_for_event`(*name*)[source]¶
    :   Return the calendar providing the specified event name.

    `get_modifiable_parameters`()[source]
    :   Return a dictionary containing all true parameters with their
        value.

    `get_prototype`(*level*, *name*)[source]¶
    :   Return a ready-to-use prototype, i.e. a StateVarDict corresponding
        to the prototype for the specified level and name, where
        symbols associated to statevariables are already replaced by
        their value in the model. ‘Hard-coded’ lists are transformed
        into tuples (to be hashable).

    `get_value`(*name*)[source]¶
    :   Return the value associated with the specified name. If the method
        is called with a name that is not in the model values, try to
        parse the expression, to prevent failures when called with a
        final value.

    `normalize_format`()[source]¶
    :   Return a YAML representation of the model, reformatted to to print
        lists and dicts using the flow style (no [], no {}) instead of
        the block style, especially for syntax checking where rules
        are defined only for flow style.

    `parse`(*description*)[source]¶
    :   Build the EmulsionModel from the specified dictionary
        (expected to come from a YAML configuration file).

    `set_value`(*name*, *value*)[source]¶
    :   Naif method to change a parameter’s value.
        Will be more efficient when treating several parameters at
        the same time.

    `write_dot`(*parent\_dir*)[source]¶
    :   Write the graph of the each state machine in the
        specified directer name, according to the dot/graphviz format.

# EMULSION

Epidemiological Multi-Level Simulation Framework

### Navigation

- 1. Installation
- 2. Getting started with EMULSION
- 3. Modelling principles
- 4. Modelling language (basics)
- 5. Modelling language (advanced)
- 6. Feature examples
- 7. Information
- 8. License
- 9. High-level functions for model designers
  - 9.1. Functions Available for Models
  - 9.2. Rates / probabilities
  - 9.3. Computations
  - 9.4. Selecting agents
  - 9.5. Durations
  - 9.6. Agent State and Variable Changes
  - 9.7. Introspection
- 10. emulsion package

### Related Topics

- Documentation overview
  - Previous: 8. License
  - Next: 10. emulsion package

### Quick search

©2016, INRA and Univ. Lille.
|
Powered by Sphinx 1.8.5
& Alabaster 0.7.10
|
Page source
