# Supplementary material for: EMULSION: Transparent and flexible multiscale stochastic models in human, animal and plant epidemiology
Source: PLoS Comput Biol. 2019 Sep 13;15(9):e1007342. doi: 10.1371/journal.pcbi.1007342 (PMC6760811; doi:10.1371/journal.pcbi.1007342)
Supplement: S2 File — This zip file is a clone of EMULSION public Git repository. To install from this file rather than from PyPI, go to EMULSION documentation page: https://sourcesup.renater.fr/www/emulsion-public/pages/Install.html and follow Git-based installation instructions. (ZIP) [file pcbi.1007342.s010.zip › S2_file/doc/pages/Install.html]

1. Installation — EMULSION (Epidemiological Multi-Level Simulation framework)

# 1. Installation¶

## 1.1. Requirements¶

System
:   EMULSION has been designed under **MacOS** and **Linux**, and
    works (with minor limitations) with **Windows 10**. See the
    installation procedure specific to your system.

    Also, in what follows, we assume that MacOS and Linux users are
    working with a **bash** shell. If you are not sure, typing: `echo
    $SHELL` in a terminal should print `/bin/bash`. Otherwise, please refer to
    the documentation of your shell to adapt the commands below.

Language
:   EMULSION is written in **Python 3** (version 3.6 or higher). In what
    follows, we assume that `python3` and `pip3` refer to your
    Python3 installation. If not, replace them by your own
    configuration in the commands below (e.g. with Windows:
    respectively `python.exe` and `pip.exe`).

    To install Python on your system, please consult
    Python website. When
    installing on Windows, check the box “Add Python 3.x to PATH” to
    make python commands available from the terminal.

## 1.2. Install with `pip` (recommended)¶


### Linux and MacOS:¶

```
sudo pip3 install emulsion
init_emulsion
source $HOME/.bashrc
```

Depending on how you installed Python, `sudo` is sometimes
optional.

The second command (`init_emulsion`) initializes command-line
completion (available with a bash shell, under Linux or MacOS), which allows
to use `TAB` key to get suggestions on what is expected (options,
files, parameters…) in the command. It also creates (or modifies)
the environment variable named `PYTHONPATH` to make EMULSION able to
find code add-ons located in the same directory as your models.

EMULSION documentation
is provided on-line.

### Windows¶

Open a terminal (“Command Prompt” or “Windows Power Shell”), then
type:

```
pip.exe install emulsion
```

To make EMULSION able to find code add-ons in the directory where your model
files are located, you must declare (or modify) an environment
variable named `PYTHONPATH` to add the current directory (“`.`”).

To do so, you can use the graphical interface (from System
Preferences), or the terminal. The syntax depends on the kind of
terminal you are using:

> - with the “Command Prompt” (`cmd.exe`), type:
>
>   ```
>   setx PYTHONPATH ".;%PYTHONPATH%"
>   ```
> - with “Windows Power Shell”, type:
>
>   ```
>   setx PYTHONPATH ".;$Env:PYTHONPATH"
>   ```

## 1.3. Install third-party software¶

Graphviz is highly recommended
(otherwise, simulations will run but you will not be able to get the
nice diagrams representing model structure).

### Linux¶

```
sudo apt install graphviz
```

### MacOS¶

To install on MacOS, use for instance homebrew

```
brew install graphviz
```

### Windows¶

To install on Windows, download the latest stable version from
Graphviz website
and update the `PATH` environment variable according to where you
installed the executable files (especially `dot.exe`).
For instance, if `dot.exe` is located in
`C:\Program Files (x86)\Graphviz\bin`, just type:

> - in the “Command Prompt”:
>
>   ```
>   setx PATH "C:\Program Files (x86)\Graphviz\bin;%PATH%"
>   ```
> - in “Windows Power Shell”:
>
>   ```
>   setx PATH "C:\Program Files (x86)\Graphviz\bin;$Env:PATH"
>   ```

## 1.4. Test your installation¶

Download **model examples** here.

Extract the archive, open a terminal in the `models` directory, then type:

```
cd quickstart
emulsion run --plot quickstart.yaml --view-model --silent
```

This should produce the following output:

```
Simulation level:population
Generated state machine diagram img/Quickstart_age_group_machine.svg
Generated state machine diagram img/Quickstart_life_cycle_machine.svg
Generated state machine diagram img/Quickstart_health_state_machine.svg
100%|*************************************************************| 10/10
Simulation finished in 12.37 s
Outputs stored in outputs/counts.csv
Outputs plot in file: img/Quickstart.html
```

and the following figures should appear in your navigator:

digraph {
rankdir=LR;
node[fontsize=16, fontname=Arial, shape=box, style="filled,rounded"];
edge[minlen=1.5, penwidth=1.5, tailtooltip="", headtooltip=""];
"J" [shape="Mrecord", label="{{\ J\ |&#9670;}}", tooltip="J (Juvenile):
juvenile animals", fillcolor=orange, style="filled,rounded,bold"] ;
"A" [shape="Mrecord", label="{{\ A\ |&#9670;}}", tooltip="A (Adult):
adult animals", fillcolor=brown, style="filled,rounded"] ;
"J" -> "A" [label="proba: 1", labeltooltip="proba: 1", arrowtail="nonetee", arrowhead="normalnone", dir=both, tooltip="COND: GreaterThan(age, adult\_age)", minlen=3, style="solid"];
}

digraph {
rankdir=LR;
node[fontsize=16, fontname=Arial, shape=box, style="filled,rounded"];
edge[minlen=1.5, penwidth=1.5, tailtooltip="", headtooltip=""];
"G" [shape="Mrecord", label="G&nbsp;&#9719;", tooltip="G (Gestating):
state for gestating animals", fillcolor=green, style="filled,rounded"] ;
"NG" [shape="Mrecord", label="NG&nbsp;&#9719;", tooltip="NG (NonGestating):
state for empty animals", fillcolor=cyan, style="filled,rounded,bold"] ;
"G" -> "NG" [label="proba: 1", labeltooltip="proba: 1", arrowtail="none", arrowhead="normalnonediamond", dir=both, tooltip="", minlen=3, style="solid"];
"G" -> "NG" [label="proba: daily\_abortion", labeltooltip="daily\_abortion [parameter]:
daily probability that an infected animal aborts during gestation
1 - (1 - global\_abortion)\*\*(1/gestation\_duration)", arrowtail="noneoinv", arrowhead="normalnonediamond", dir=both, tooltip="ESCAPE: is\_I", minlen=3, style="solid"];
"NG" -> "G" [label="rate: reproduction", labeltooltip="reproduction [parameter]:
reproduction rate of the animals
0.05", arrowtail="nonetee", arrowhead="normalnone", dir=both, tooltip="COND: is\_A", minlen=3, style="solid"];
}

digraph {
charset="utf-8"
rankdir=LR;
node[fontsize=16, fontname=Arial, shape=box, style="filled,rounded"];
edge[minlen=1.5, fontname=Times, penwidth=1.5, tailtooltip="", headtooltip=""];
"M" [shape="Mrecord", label="M&nbsp;&#9719;", tooltip="M (Maternal protection):
animal protected by maternal antibodies", fillcolor=cyan, style="filled,rounded"] ;
"S" [shape="Mrecord", label="S", tooltip="S (Susceptible):
suceptible of becoming infectious", fillcolor=wheat, style="filled,rounded,bold"] ;
"E" [shape="Mrecord", label="E&nbsp;&#9719;", tooltip="E (Exposed):
already infected but not yet able to transmit the disease", fillcolor=lightcoral, style="filled,rounded"] ;
"I" [shape="Mrecord", label="I", tooltip="I (Infectious):
sick and able to transmit the disease", fillcolor=maroon, style="filled,rounded"] ;
"R" [shape="Mrecord", label="R", tooltip="R (Resistant):
healthy again and resistant to infection", fillcolor=deepskyblue, style="filled,rounded"] ;
"Q" [shape="Mrecord", label="Q&nbsp;&#9719;", tooltip="Q (Quarantine):
sick animals that are put aside for a while", fillcolor=green, style="filled,rounded"] ;
"D" [shape="Mrecord", label="D", tooltip="D (Dead):
dead animals", fillcolor=white, style="filled,rounded,dotted"] ;
"E" -> "D" [label="rate: mortality\_sick", labeltooltip="mortality\_sick [parameter]:
mortality rate for sick animals (E / I / Q)
0.004", arrowtail="noneoinv", arrowhead="normalnone", dir=both, tooltip="ESCAPE: True", minlen=3, style="solid"];
"E" -> "I" [label="rate: alpha", labeltooltip="alpha [parameter]:
rate for E->I transition
0.3", arrowtail="none", arrowhead="normalnone", dir=both, tooltip="", minlen=3, style="solid"];
"I" -> "D" [label="rate: mortality\_sick", labeltooltip="mortality\_sick [parameter]:
mortality rate for sick animals (E / I / Q)
0.004", arrowtail="none", arrowhead="normalnone", dir=both, tooltip="", minlen=3, style="solid"];
"I" -> "Q" [label="rate: detection", labeltooltip="detection [parameter]:
rate at which animals are detected sick (falls to 0 if quarantine zone is full)
0.01\*room\_in\_quarantine", arrowtail="noneodot", arrowhead="normalnone", dir=both, tooltip="WHEN: quarantine\_period", minlen=3, style="solid"];
"I" -> "R" [label="rate: gamma", labeltooltip="gamma [parameter]:
recovery rate
0.01", arrowtail="none", arrowhead="normalnone", dir=both, tooltip="", minlen=3, style="solid"];
"M" -> "D" [label="rate: mortality\_healthy", labeltooltip="mortality\_healthy [parameter]:
mortality rate for healthy animals
0.002", arrowtail="noneoinv", arrowhead="normalnone", dir=both, tooltip="ESCAPE: True", minlen=3, style="solid"];
"M" -> "S" [label="rate: epsilon", labeltooltip="epsilon [parameter]:
maternal antibodies loss rate
0.04", arrowtail="none", arrowhead="normalnone", dir=both, tooltip="", minlen=3, style="solid"];
"Q" -> "D" [label="rate: mortality\_sick", labeltooltip="mortality\_sick [parameter]:
mortality rate for sick animals (E / I / Q)
0.004", arrowtail="noneoinv", arrowhead="normalnone", dir=both, tooltip="ESCAPE: True", minlen=3, style="solid"];
"Q" -> "R" [label="proba: 1", labeltooltip="proba: 1", arrowtail="none", arrowhead="normalnone", dir=both, tooltip="", minlen=3, style="solid"];
"R" -> "D" [label="rate: mortality\_healthy", labeltooltip="mortality\_healthy [parameter]:
mortality rate for healthy animals
0.002", arrowtail="none", arrowhead="normalnone", dir=both, tooltip="", minlen=3, style="solid"];
"R" -> "S" [label="rate: delta", labeltooltip="delta [parameter]:
resistance loss rate
0.01", arrowtail="none", arrowhead="normalnone", dir=both, tooltip="", minlen=3, style="solid"];
"S" -> "D" [label="rate: mortality\_healthy", labeltooltip="mortality\_healthy [parameter]:
mortality rate for healthy animals
0.002", arrowtail="none", arrowhead="normalnone", dir=both, tooltip="", minlen=3, style="solid"];
"S" -> "E" [label="rate: f", labeltooltip="f [parameter]:
force of infection
beta \* total\_I / total\_herd", arrowtail="none", arrowhead="normalnone", dir=both, tooltip="", minlen=3, style="solid"];
}

## 1.5. Alternative: install with `git`¶

This procedure assumes that you have `git` already
installed on your system.
It is provided here for Linux or MacOS.

1. Install (or update) required packages

   ```
   sudo pip3 install numpy scipy matplotlib pandas 'sympy==1.1.1' mpmath
   sudo pip3 install networkx docopt jinja2 textx
   sudo pip3 install sortedcontainers tqdm pyyaml colorama
   sudo pip3 install bokeh sqlalchemy utm
   ```
2. Clone the EMULSION repository

   ```
   git clone https://git.renater.fr/anonscm/git/emulsion-public/emulsion-public.git
   cd emulsion-public
   ```
3. Add the local directory and `src` directory to your `PYTHONPATH` environment
   variable.

   ```
   echo "export PYTHONPATH=$(pwd)/src:." >>$HOME/.bashrc
   ```
4. Install command-line completion (optional but very convenient).

   ```
   echo "source $(pwd)/src/emulsion/scripts/emulsion-completion.sh" >>$HOME/.bashrc
   ```

   Command-line completion (available with a bash shell, under Linux
   or MacOS) allows to use `TAB` key to get suggestions on what is
   expected (options, files, parameters…) in the command.
5. Create command `emulsion`:

   ```
   echo "alias emulsion='python3 -m emulsion'" >>$HOME/.bashrc
   ```
6. Force the shell to update your init file:

   ```
   source $HOME/.bashrc
   ```

# EMULSION

Epidemiological Multi-Level Simulation Framework

### Navigation

- 1. Installation
  - 1.1. Requirements
  - 1.2. Install with `pip` (recommended)
    - Linux and MacOS:
    - Windows
  - 1.3. Install third-party software
    - Linux
    - MacOS
    - Windows
  - 1.4. Test your installation
  - 1.5. Alternative: install with `git`
- 2. Getting started with EMULSION
- 3. Modelling principles
- 4. Modelling language (basics)
- 5. Modelling language (advanced)
- 6. Feature examples
- 7. Information
- 8. License
- 9. High-level functions for model designers
- 10. emulsion package

### Related Topics

- Documentation overview
  - Previous: EMULSION Manual
  - Next: 2. Getting started with EMULSION

### Quick search

©2016, INRA and Univ. Lille.
|
Powered by Sphinx 1.8.5
& Alabaster 0.7.10
|
Page source
