# Supplementary material for: EMULSION: Transparent and flexible multiscale stochastic models in human, animal and plant epidemiology
Source: PLoS Comput Biol. 2019 Sep 13;15(9):e1007342. doi: 10.1371/journal.pcbi.1007342 (PMC6760811; doi:10.1371/journal.pcbi.1007342)
Supplement: S2 File — This zip file is a clone of EMULSION public Git repository. To install from this file rather than from PyPI, go to EMULSION documentation page: https://sourcesup.renater.fr/www/emulsion-public/pages/Install.html and follow Git-based installation instructions. (ZIP) [file pcbi.1007342.s010.zip › S2_file/doc/pages/Modelling_principles.html]

3. Modelling principles — EMULSION (Epidemiological Multi-Level Simulation framework)

# 3. Modelling principles¶

First, a few words on key concepts used in EMULSION.

## 3.1. Individuals, populations, metapopulations¶

EMULSION models can represent the dynamics of several organization
levels (or scales), through classical paradigms used in epidemiological modelling:

- **Compartment-based models** represent aggregate populations sharing a
  same state (e.g. same health state, same age group…). Individuals,
  thus, are not represented explicitly. The population is the mapping
  between states and the amount of individuals in those states
  (Fig. 3.1, right).
- **Individual-based models (IBM)**, on the contrary, focus on an
  explicit depiction of individual specificities (in states, actions,
  processes…) to account for finer grained assumptions, or represent
  specific control measures. The population is a collection of
  individuals (Fig. 3.1, left).
- EMULSION also provides **hybrid models**, which combine the
  capability of representing detailed information through individuals,
  with an adaptive grouping of individuals based on their state, to
  optimize computation (Fig. 3.1, center).

  In addition, EMULSION can handle **metapopulations**, i.e. a
  collection of interconnected populations (for instance through
  movements of individuals, airborne diffusion of pathogens,
  etc.). Metapopulations can aggregate populations built on
  compartment-based, individual-based or hybrid modelling paradigms.

Fig. 3.1 From individuals to aggregate populations in EMULSION

## 3.2. From flow diagrams to state machines¶

### Flow diagrams¶

Flow diagrams are a broadly adopted representation in epidemiological
modelling. Possible states are represented as boxes. Plain arrows
specify at which rate individuals move from one state to another. For
instance, a classical flow diagram to represent a SIR model (assuming
frequency-dependent force of infection) will look as:

digraph {
rankdir=LR;
node[fontsize=16, fontname="Arial", shape="box", style="bold"];
edge[penwidth=1.5];
"S" [shape="box", label="S", tooltip="S (Susceptible): not infected"] ;
"I" [shape="box", label="I", tooltip="I (Infectious):
infected and able to transmit the disease"] ;
"R" [shape="box", label="R", tooltip="R (Resistant):
healthy again and resistant to infection"] ;
"I" -> "R" [label="γ", labeltooltip="rate: γ", arrowtail="none", arrowhead="normalnone", dir=both, tooltip="I individuals become R at rate γ", minlen=2, style="solid"];
"S" -> "I" [label="βI/N", labeltooltip="rate: βI/N", arrowtail="none", arrowhead="normalnone", dir=both, tooltip="S individuals become I at rate βI/N", minlen=2, style="solid"];
}

Under the classical assumption that durations in each state follow an
exponential distribution, this graphical representation can be easily
translated into either an ODE system which provides a mathematical
representation of a deterministic SIR model, or a system of stochastic
difference equations.

However, flow diagrams have two main drawbacks:

1. Dealing with several concerns at the same time (e.g. infectious
   process and age groups) implies that all states must be subdivided
   according to each concern (e.g. infected juveniles vs. infected
   adults), hence a multiplication of boxes and arrows;
2. With more complex models which do not stand classical assumptions
   or intend to represent individual-based conditions or actions ,
   many model features cannot be represented explicitly.

### State machines¶

State machines (Fig. 3.2) is a formalism which
completely encompasses flow diagrams but can also support more complex
features while making them appear explicitly. Especially:

- each process involved in the system dynamics (e.g. infection, age
  groups, demography, treatment protocol, etc.) has to be
  represented by its own state machine: hence, instead of a large,
  complicated diagram, EMULSION fosters a separation between concerns
  through several smaller diagrams
- state machines represent the dynamics of individual situations
  instead of the evolution of populations
- each state can be endowed with additional properties:
  - an explicit duration
  - actions to perform when entering, staying in, or leaving the state
- each transition is labelled with either a **rate**, or a
  **probability**, or an absolute **amount** to better control the
  flow from one state to the other, and can also be associated with:
  - individual conditions to filter which individuals are allowed to
    cross the transition
  - temporal conditions to enable/disable transitions at targeted
    periods of time
  - escape conditions to allow some individuals to leave their state
    before the expected duration
  - actions to perform for individuals crossing the transition

Fig. 3.2 State machines are similar to flow diagrams but can represent much
more explicit details (here all features are represented at the
same time)

Note

In a state machine, each possible pathway from one state to another
must be represented by one transition, with its own features
(rate/proba/amount, conditions, actions).

# EMULSION

Epidemiological Multi-Level Simulation Framework

### Navigation

- 1. Installation
- 2. Getting started with EMULSION
- 3. Modelling principles
  - 3.1. Individuals, populations, metapopulations
  - 3.2. From flow diagrams to state machines
    - Flow diagrams
    - State machines
- 4. Modelling language (basics)
- 5. Modelling language (advanced)
- 6. Feature examples
- 7. Information
- 8. License
- 9. High-level functions for model designers
- 10. emulsion package

### Related Topics

- Documentation overview
  - Previous: 2. Getting started with EMULSION
  - Next: 4. Modelling language (basics)

### Quick search

©2016, INRA and Univ. Lille.
|
Powered by Sphinx 1.8.5
& Alabaster 0.7.10
|
Page source
