# Supplementary material for: EMULSION: Transparent and flexible multiscale stochastic models in human, animal and plant epidemiology
Source: PLoS Comput Biol. 2019 Sep 13;15(9):e1007342. doi: 10.1371/journal.pcbi.1007342 (PMC6760811; doi:10.1371/journal.pcbi.1007342)
Supplement: S2 File — This zip file is a clone of EMULSION public Git repository. To install from this file rather than from PyPI, go to EMULSION documentation page: https://sourcesup.renater.fr/www/emulsion-public/pages/Install.html and follow Git-based installation instructions. (ZIP) [file pcbi.1007342.s010.zip › S2_file/doc/pages/paradigms.html]

<no title> — EMULSION (Epidemiological Multi-Level Simulation framework)

| EMULSION section | section keyword | Modelling paradigm | | |
| --- | --- | --- | --- | --- |
|  |  | compartment | hybrid | IBM |
| `levels` | `aggregation_type` | `compartment` | `hybrid` | `IBM` |
|  | `contains` | *not used* | *sublevels* | |
| `grouping` | `key_variables` | *list of variables* | | *not used* |
|  | `state_machine` | *machine name* (optional) | |  |
| `processes` | *level* | *population* | | *individuals* |
|  | *list of processes* | *names of groupings* | | *machine name* |
| `state_machines` | `productions` |  | *based on prototypes* | |
| `prototypes` |  | *not used* | *for individuals/populations* | |
| `initial_conditions` |  | *per state* | *based on prototypes* | |

# EMULSION

Epidemiological Multi-Level Simulation Framework

### Navigation

- 1. Installation
- 2. Getting started with EMULSION
- 3. Modelling principles
- 4. Modelling language (basics)
- 5. Modelling language (advanced)
- 6. Feature examples
- 7. Information
- 8. License
- 9. High-level functions for model designers
- 10. emulsion package

### Related Topics

- Documentation overview

### Quick search

©2016, INRA and Univ. Lille.
|
Powered by Sphinx 1.8.5
& Alabaster 0.7.10
|
Page source
