# Supplementary material for: EMULSION: Transparent and flexible multiscale stochastic models in human, animal and plant epidemiology
Source: PLoS Comput Biol. 2019 Sep 13;15(9):e1007342. doi: 10.1371/journal.pcbi.1007342 (PMC6760811; doi:10.1371/journal.pcbi.1007342)
Supplement: S2 File — This zip file is a clone of EMULSION public Git repository. To install from this file rather than from PyPI, go to EMULSION documentation page: https://sourcesup.renater.fr/www/emulsion-public/pages/Install.html and follow Git-based installation instructions. (ZIP) [file pcbi.1007342.s010.zip › S2_file/doc/pages/Modelling_language_basics.html]

4. Modelling language (basics) — EMULSION (Epidemiological Multi-Level Simulation framework)

# 4. Modelling language (basics)¶

## 4.1. YAML Syntax in a nutshell¶

EMULSION models must respect the YAML format, which is based
on lists and key-value mappings. Data structures are delimited by
2-space indentation.

Comments
:   Whatever is put after `#` is not interpreted.

Values
:   Numbers (`3`, `3.14`), strings (`'some text'`), booleans
    (`yes`/`no`), lists or key-value mappings.

Lists
:   A succession of values, e.g.

    ```
    [value1, value2, value3]
    ```

    which is equivalent to:

    ```
    - value1
    - value2
    - value3
    ```

Key-value mappings
:   An (unordered) set of associations between unique indentifiers
    (keys) and any value, e.g.

    ```
    {key1: value1, key2: value2, key3: value3}
    ```

    which is equivalent to:

    ```
    key1: value1
    key2: value2
    key3: value3
    ```

All elements above can be combined and nested to build complex
structures, for instance:

```
# Here a key mapped to a list
key1: [v1, v2, v3]

# Here a key mapped to another mapping
key2:
  subkey1:
    # the value associated with subkey1 is a list
    - item1
    - item2
  subkey2: 'an important message'
  subkey3:
    # and each element of the list below is a mapping
    - another: value1
      withother: value2
    - another: value3
      withother: value4
```

## 4.2. Model structure¶

An EMULSION model is divided in several “sections”, corresponding to
the main components of a model. Each section corresponds to a
first-level key (i.e. put directly at the beginning of a line without
any indentation).

Below is a short description of their nature. This is just an overview
of what can be found in a typical EMULSION model. To go further, dive
into the next chapter!

### `model_name`¶

The name of the model. Used to name figures and diagrams.

Example

```
model_name: compart_SIR
```

### `model_info`¶

Several optional information on the model, such as an abstract to
describe the model principles and purpose, the authors, references, a
license if any, etc.

Example

```
model_info:
  abstract: 'A very long description of the model'
  authors:
    - First Author
    - Another Colleague
  DOI: my_doi/10.10.10.
```

This part is only intended to provide information to the reader. All
subsections can be freely defined according to the modeller’s needs.

### `time_info`¶

This section defines the **time unit** used in the whole model for
parameter values (e.g. hours, days, weeks) and the **duration of one time
step** in the simulation. Optionally, it can specify:

- the date where the simulation starts (`origin_date`)
- the total duration of the simulation (`total_duration`)
- calendars with events (see Regulate time)

Example

```
time_info:
  # all paramter values are expressed in days/per day
  time_unit: 'days'
  # the simulation step is 1 day
      delta_t: 1
  # simulations start on 01/01 (default: current year)
  origin: 'January 1'
  # simulations run for 100 days
  total_duration: '100'
```

### `state_machines`¶

State machines are the main way to define processes involved in
an EMULSION model. A state machine is defined by a list of states and
a list of transitions between the states. It can also define a list of
productions links between states, to specify which states can produce
new individuals.

An EMULSION model can contain several state machines, the only
constraint being that all state names must be different.

Example of a typical state machine

```
state_machines:
  health_state:  # the name of the state machine
        desc: 'The state machine which defines the evolution of health
        states'
        # Below, the list of states with their attributes.
        states:
          - S:
              name: 'Susceptible'
              desc: 'suceptible of becoming infected'
              fillcolor: 'deepskyblue'
          - I:
              name: 'Infectious'
              desc: 'infected and able to transmit the disease'
              fillcolor: 'red'
          - R:
              name: 'Resistant'
              desc: 'healthy again and resistant to infection'
              fillcolor: 'limegreen'
        # Below, a list of transitions between states
        transitions:
          - {from: S, to: I, rate: 'force_of_infection'}
          - {from: I, to: R, rate: 'recovery'}
```

### `levels`¶

In EMULSION, a **level** is an organization level which is explicitly
represented in the model. For instance, a compartment model has only
one level, the population, since individuals are only considered
implicitly. A level can contain other sub-levels, base on a specific
aggregation type (`compartment`, `IBM`, `hybrid` or
`metapopulation`). A level can also define aggregated variables
calculated from the values of another variable at the sublevel.

Examples

A typical level specification in a compartment-based/hybrid
model:

```
levels:
  population:
    desc: 'level of the population'
    aggregation_type: 'compartment'
```

A typical levels specification in an individual-based model:

```
levels:
  population:
    desc: 'level of the population'
    aggregation_type: 'IBM'
    contains:
      - individuals  # the sublevel
  individuals:
    desc: 'level of the individuals'
```

### `grouping`¶

This section only concerns compartment-based and hybrid models. It
specifies upon which individual variables a population is partitioned,
and optionnally the name of a state machine which drives the evolution
of the corresponding groups.

For instance, the infection process is driven by the `health_state`
state machine. The value of variable `health_state` is thus the key
to partition the population (all S individuals together, all I
together, etc.)

Example

```
grouping:
  population:
    infection:
      machine_name: health_state
      key_variables: [health_state]
```

### `processes`¶

This section specifies the list of major processes that take place at
each level during the simulation. A process name can be:

- the name of a grouping in compartment-based or hybrid models (hence
  taking place at the population level)
- the name of a state machine in individual-based models (hence taking
  place at the individual level)
- the name of a Python method developed specifically in a code add-on

Examples

A typical processes specification in a compartment-based/hybrid
model:

```
processes:
  population:
    - infection
```

A typical processes specification in an individual-based model:

```
processes:
  individuals:
    - health_state
```

### `parameters`¶

This section is intended to define:

- model parameters (*stricto sensu*), i.e. numerical values coming
  from experts, data or assumptions and driving the dynamics of the
  model
- configuration parameters, i.e. numerical values used in initial
  conditions or scenario definition
- distributions expressed by functions and returning a new sample each
  time they are “used” in a computation
- expressions which can combine other parameters or variables

Each entry must be endowed with a full description of its role
(`desc:`) and can also provide information on where the value (or
expression) comes from (`source:`).

Example

```
parameters:
  # a model parameter
  transmission_I:
    desc: 'transmission rate from infectious individuals (/day)'
    value: 0.5
  # an expression (of another parameter and variables)
  force_of_infection:
    desc: 'infection function'
    value: 'transmission_I * total_I / total_population'
    source: 'classical function assuming frequency dependence'
  # a distribution
  initial_age:
    desc: 'distribution of ages when initializing individuals'
    value: 'random_integers(0, 20)'
```

### `prototypes`¶

Prototypes are used for IBM/hybrid models and for
metapopulations. They are intended to specify typical individuals or
populations which are characterized by specific values of their
variables.

Prototypes are used mainly in two occasions:

- in initial conditions (see below), to specify how many individuals
  of each kind must be created
- in production links of state machines, to indicate the nature of
  individuals produced
- in the built-in action `become`, to make a state machine induce
  changes in another state machine

Example

```
prototypes:
  # here the level for which the prototypes are defined
  individuals:
    - healthy:  # the name of the prototype
        desc: 'healthy individuals'
        health_state: S
        # variable age_group is one of the existing states
        age_group: random
    - infected:
        desc: 'infected individuals'
        health_state: I
        # here we intend to start with infected juveniles
        age_group: J
```

See also

To go further: Design prototypes for typical individuals or populations

### `initial_conditions`¶

Initial conditions specify how to initialize each level.

In compartment-based models, initial conditions give the total
population and the repartition in each compartment.

In IBM, hybrid models and metapopulations, initial conditions rely on
prototypes.

Examples

A typical specification of initial conditions in a
compartment-based model:

```
initial_conditions:
  # the level for which the initial conditions are defined
  population:
    - population:
        - total: 'initial_population_size'
        - vars: [S]  # amount of individuals in state S
          amount: 'initial_population_size - initial_infected'
        - vars: [I] # amount of individuals in state I
          amount: 'initial_infected'
        # the amount of individuals in state R is computed as:
        # (total - S - I)
```

A typical specification of initial conditions in an IBM:

```
initial_conditions:
  population:
    # a list of prototypes with the number of individuals
    # to create with each prototype
    - prototype: healthy
      amount: 'initial_population_size - initial_infected'
    - prototype: infected
      amount: 'initial_infected'
```

### `outputs`¶

When running an EMULSION model, the amounts of individuals in each
state for all state machines are computed automatically at each time
step. The outputs section specifies how the output data are stored
(CSV file, database…) and at which period (in time steps).
Additional variables can also be logged (`extra_vars`).

Using command-line option `--plot`, one plot per state machine is
automatically produced, as well as one plot for all extra variables.

Example

```
outputs:
  type: csv  # produces counts.csv in output directory
  population:      # outputs for level population
    period: 1  # at each time step
    extra_vars:
      # add an expression (from 'parameters')
      - 'prevalence (%)'
      # add a population variable
      - total_population
```

### `actions`¶

This section appears when a level requires actions which are not
provided in EMULSION generic engine. In that case, the section
contains a simple description of the meaning of the action, which has
to be defined in a separate Python file.

Example

```
actions:
  read_mailbox:
    desc: 'Check if messages were received from other
    individuals and modify contact network accordingly.'
```

### `statevars`¶

This section appears when a level requires variables which are not
defined automatically by their state machines, nor defined as
expressions in the parameters section, nor computed by aggregating
variables from a sublevel. In that case, the section contains a simple
description of the meaning of the variable, which has to be defined in
a separate Python file.

Example

```
statevars:
  nb_mothers_of_infected_calf:
    desc: 'identify and counts cows which gave birth to an
    infected calf'
```

# EMULSION

Epidemiological Multi-Level Simulation Framework

### Navigation

- 1. Installation
- 2. Getting started with EMULSION
- 3. Modelling principles
- 4. Modelling language (basics)
  - 4.1. YAML Syntax in a nutshell
  - 4.2. Model structure
    - `model_name`
    - `model_info`
    - `time_info`
    - `state_machines`
    - `levels`
    - `grouping`
    - `processes`
    - `parameters`
    - `prototypes`
    - `initial_conditions`
    - `outputs`
    - `actions`
    - `statevars`
- 5. Modelling language (advanced)
- 6. Feature examples
- 7. Information
- 8. License
- 9. High-level functions for model designers
- 10. emulsion package

### Related Topics

- Documentation overview
  - Previous: 3. Modelling principles
  - Next: 5. Modelling language (advanced)

### Quick search

©2016, INRA and Univ. Lille.
|
Powered by Sphinx 1.8.5
& Alabaster 0.7.10
|
Page source
