# Supplementary material for: EMULSION: Transparent and flexible multiscale stochastic models in human, animal and plant epidemiology
Source: PLoS Comput Biol. 2019 Sep 13;15(9):e1007342. doi: 10.1371/journal.pcbi.1007342 (PMC6760811; doi:10.1371/journal.pcbi.1007342)
Supplement: S2 File — This zip file is a clone of EMULSION public Git repository. To install from this file rather than from PyPI, go to EMULSION documentation page: https://sourcesup.renater.fr/www/emulsion-public/pages/Install.html and follow Git-based installation instructions. (ZIP) [file pcbi.1007342.s010.zip › S2_file/doc/pages/Feature_examples.html]

6. Feature examples — EMULSION (Epidemiological Multi-Level Simulation framework)

# 6. Feature examples¶

Directory `models/features` provides a collection of EMULSION models
which demonstrate EMULSION features in their simplest usage.

## 6.1. SIR model¶

Files

- compartment-based: compart\_SIR.yaml
- individual-based: IBM\_SIR.yaml
- hybrid model: hybrid\_SIR.yaml

**Move cursor over diagram states and transitions to see the tooltip**

digraph {
rankdir=LR;
node[fontsize=16, fontname=Arial, shape=box, style="filled,rounded"];
edge[minlen=1.5, penwidth=1.5, tailtooltip="", headtooltip=""];
"S" [shape="Mrecord", label="S", tooltip="S (Susceptible):
suceptible of becoming infected", fillcolor=wheat, style="filled,rounded"] ;
"I" [shape="Mrecord", label="I", tooltip="I (Infectious):
infected and able to transmit the disease", fillcolor=maroon, style="filled,rounded"] ;
"R" [shape="Mrecord", label="R", tooltip="R (Resistant):
healthy again and resistant to infection", fillcolor=deepskyblue, style="filled,rounded"] ;
"I" -> "R" [label="rate: recovery", labeltooltip="recovery [parameter]:
recovery rate (/day)
0.1", arrowtail="none", arrowhead="normalnone", dir=both, tooltip="", minlen=3, style="solid"];
"S" -> "I" [label="rate: force\_of\_infection", labeltooltip="force\_of\_infection [parameter]:
infection function
transmission\_I \* total\_I / total\_herd", arrowtail="none", arrowhead="normalnone", dir=both, tooltip="", minlen=3, style="solid"];
}

Note

Variables `total_I` and `total_population` used in force of infection
(see tooltip when moving your mouse above `force_of_infection` in
the diagram above) are computed automatically because of the
existence of a level `population` and a state `I`.

See also

Model structure,
Compartments, IBM or hybrid models?,
Automatic variables

## 6.2. SEIRS model¶

Files

- compart\_SEIRS.yaml
- IBM\_SEIRS.yaml
- hybrid\_SEIRS.yaml

digraph {
rankdir=LR;
node[fontsize=16, fontname=Arial, shape=box, style="filled,rounded"];
edge[minlen=1.5, penwidth=1.5, tailtooltip="", headtooltip=""];
"S" [shape="Mrecord", label="S", tooltip="S (Susceptible):
suceptible of becoming infected", fillcolor=wheat, style="filled,rounded"] ;
"E" [shape="Mrecord", label="E", tooltip="E (Latent):
infected but not yet able to transmit the disease", fillcolor=orange, style="filled,rounded"] ;
"I" [shape="Mrecord", label="I", tooltip="I (Infectious):
infected and able to transmit the disease", fillcolor=maroon, style="filled,rounded"] ;
"R" [shape="Mrecord", label="R", tooltip="R (Resistant):
healthy again and resistant to infection", fillcolor=deepskyblue, style="filled,rounded"] ;
"E" -> "I" [label="rate: 1
──────────
incubation", labeltooltip="rate: 1
──────────
incubation", arrowtail="none", arrowhead="normalnone", dir=both, tooltip="", minlen=3, style="solid"];
"I" -> "R" [label="rate: recovery", labeltooltip="recovery [parameter]:
recovery rate (/day)
0.1", arrowtail="none", arrowhead="normalnone", dir=both, tooltip="", minlen=3, style="solid"];
"R" -> "S" [label="rate: waning", labeltooltip="waning [parameter]:
rate at which individuals lose immunity (/day)
0.05", arrowtail="none", arrowhead="normalnone", dir=both, tooltip="", minlen=3, style="solid"];
"S" -> "E" [label="rate: force\_of\_infection", labeltooltip="force\_of\_infection [parameter]:
infection function
transmission\_I \* total\_I / total\_herd", arrowtail="none", arrowhead="normalnone", dir=both, tooltip="", minlen=3, style="solid"];
}

Differences with previous model (SIR):

```
state_machines:
  health_state:
    states:
    ...
      - E:
          name: 'Latent'
          desc: 'infected but not yet able to transmit the disease'
          fillcolor: 'orange'
    transitions:
      - {from: S, to: E, rate: 'force_of_infection'}
      - {from: E, to: I, rate: '1/incubation'}
      - {from: I, to: R, rate: 'recovery'}
      - {from: R, to: S, rate: 'waning'}
...
parameters:
  ...
    incubation:
    desc: 'mean duration of latent state (days, assuming exponential
      distribution of durations in the state)'
      value: 5
    waning:
      desc: 'rate at which individuals lose immunity (/day)'
      value: 0.05
    'prevalence (%)':
      desc: 'proportion of infected+infectious individuals'
      value: '100 * (total_I + total_E) / total_population'
```

See also

Model structure,
Compartments, IBM or hybrid models?

## 6.3. SIRS model with periodic external risk¶

Files

- compart\_SIRS\_periodic\_risk.yaml
- hybrid\_SIRS\_periodic\_risk.yaml
- IBM\_SIRS\_periodic\_risk.yaml

digraph {
rankdir=LR;
node[fontsize=16, fontname=Arial, shape=box, style="filled,rounded"];
edge[minlen=1.5, penwidth=1.5, tailtooltip="", headtooltip=""];
"S" [shape="Mrecord", label="S", tooltip="S (Susceptible):
suceptible of becoming infected", fillcolor=wheat, style="filled,rounded"] ;
"I" [shape="Mrecord", label="I", tooltip="I (Infectious):
infected and able to transmit the disease", fillcolor=maroon, style="filled,rounded"] ;
"R" [shape="Mrecord", label="R", tooltip="R (Resistant):
healthy again and resistant to infection", fillcolor=deepskyblue, style="filled,rounded"] ;
"I" -> "R" [label="rate: recovery", labeltooltip="recovery [parameter]:
recovery rate (/day)
0.1", arrowtail="none", arrowhead="normalnone", dir=both, tooltip="", minlen=3, style="solid"];
"R" -> "S" [label="rate: waning", labeltooltip="waning [parameter]:
rate at which individuals lose immunity (/day)
0.01", arrowtail="none", arrowhead="normalnone", dir=both, tooltip="", minlen=3, style="solid"];
"S" -> "I" [label="rate: force\_of\_infection", labeltooltip="force\_of\_infection [parameter]:
infection function
(external\_risk + transmission\_I \* total\_I) / total\_herd", arrowtail="none", arrowhead="normalnone", dir=both, tooltip="", minlen=3, style="solid"];
}

Note

- this model is initialized with a healthy population only
- external risk is modelled through a periodic forcing variable:

  ```
  transmission_I * (1 + sin(time * 2 * pi / external_risk_period))
  ```

  where `time` represents the time elapsed since the beginning of
  simulation (in time units - here days)

See also

Automatic variables,
Built-in functions

## 6.4. Custom state durations¶

Files

- hybrid\_duration.yaml
- IBM\_duration.yaml

digraph {
rankdir=LR;
node[fontsize=16, fontname=Arial, shape=box, style="filled,rounded"];
edge[minlen=1.5, penwidth=1.5, tailtooltip="", headtooltip=""];
"N" [shape="Mrecord", label="N&nbsp;&#9719;", tooltip="N (Newborn):
very young animals", fillcolor=deepskyblue, style="filled,rounded,bold"] ;
"J" [shape="Mrecord", label="J&nbsp;&#9719;", tooltip="J (Juvenile):
juvenile animals", fillcolor=orange, style="filled,rounded"] ;
"A" [shape="Mrecord", label="A", tooltip="A (Adult):
adult animals (producing new juveniles)", fillcolor=brown, style="filled,rounded"] ;
"J" -> "A" [label="proba: 1", labeltooltip="proba: 1", arrowtail="none", arrowhead="normalnone", dir=both, tooltip="", minlen=3, style="solid"];
"N" -> "J" [label="proba: 1", labeltooltip="proba: 1", arrowtail="none", arrowhead="normalnone", dir=both, tooltip="", minlen=3, style="solid"];
}

```
state_machines:
  health_state:
    states:
      - N:
          name: 'Newborn'
          duration: 'juvenile_age'
      - J:
          name: 'Juvenile'
          duration: 'adult_age - juvenile_age'
      ...
parameters:
  juvenile_age:
    value: 20
  adult_age:
    value: 'random_uniform(50, 70)'
```

Note

- Duration in `N` is constant (20 days)
- Duration in `J` depends on a distribution (parameter
  `adult_age`), hence each individual gets a duration based on a
  random sample
- Transitions use `proba: 1` to ensure that all individuals that
  have spent the required duration in their state move to the next
  one.
- On state machine diagrams, a clock appears next to the state name
  when a duration is explicitly specified.

See also

Set states attributes,
Built-in functions

## 6.5. SIR model with basic demography (births/deaths)¶

Files

- compart\_SIR\_demo.yaml
- hybrid\_SIR\_demo.yaml
- IBM\_SIR\_demo.yaml

digraph {
rankdir=LR;
node[fontsize=16, fontname=Arial, shape=box, style="filled,rounded"];
edge[minlen=1.5, penwidth=1.5, tailtooltip="", headtooltip=""];
"S" [shape="Mrecord", label="S", tooltip="S (Susceptible):
suceptible of becoming infected", fillcolor=wheat, style="filled,rounded"] ;
"I" [shape="Mrecord", label="I", tooltip="I (Infectious):
infected and able to transmit the disease", fillcolor=maroon, style="filled,rounded"] ;
"R" [shape="Mrecord", label="R", tooltip="R (Resistant):
healthy again and resistant to infection", fillcolor=deepskyblue, style="filled,rounded"] ;
"D" [shape="Mrecord", label="D", tooltip="D (Dead):
compartment to put dead animals", fillcolor=white, style="filled,rounded,dotted"] ;
"I" -> "D" [label="rate: death", labeltooltip="death [parameter]:
the death rate (/day)
birth", arrowtail="none", arrowhead="normalnone", dir=both, tooltip="", minlen=3, style="solid"];
"I" -> "R" [label="rate: recovery", labeltooltip="recovery [parameter]:
recovery rate (/day)
0.1", arrowtail="none", arrowhead="normalnone", dir=both, tooltip="", minlen=3, style="solid"];
"I" -> "S" [label="rate: birth", labeltooltip="birth [parameter]:
the birth rate (/day)
0.01", arrowtail="none", arrowhead="normalnone", dir=both, tooltip="", minlen=3, style="dashed"];
"R" -> "D" [label="rate: death", labeltooltip="death [parameter]:
the death rate (/day)
birth", arrowtail="none", arrowhead="normalnone", dir=both, tooltip="", minlen=3, style="solid"];
"R" -> "S" [label="rate: birth", labeltooltip="birth [parameter]:
the birth rate (/day)
0.01", arrowtail="none", arrowhead="normalnone", dir=both, tooltip="", minlen=3, style="dashed"];
"S" -> "D" [label="rate: death", labeltooltip="death [parameter]:
the death rate (/day)
birth", arrowtail="none", arrowhead="normalnone", dir=both, tooltip="", minlen=3, style="solid"];
"S" -> "I" [label="rate: force\_of\_infection", labeltooltip="force\_of\_infection [parameter]:
infection function
transmission\_I \* total\_I / total\_herd", arrowtail="none", arrowhead="normalnone", dir=both, tooltip="", minlen=3, style="solid"];
"S" -> "S" [label="rate: birth", labeltooltip="birth [parameter]:
the birth rate (/day)
0.01", arrowtail="none", arrowhead="normalnone", dir=both, tooltip="", minlen=3, style="dashed"];
}

```
state_machines:
  health_state:
    states:
      ...
      - D:
          name: 'Dead'
          desc: 'compartment to put dead individuals'
          fillcolor: white
          autoremove: yes
  transitions:
  ...
    - {from: S, to: D, rate: death}
    - {from: I, to: D, rate: death}
    - {from: R, to: D, rate: death}
  productions:
    - {from: S, to: S, rate: birth, prototype: newborn}
    - {from: I, to: S, rate: birth, prototype: newborn}
    - {from: R, to: S, rate: birth, prototype: newborn}

...
parameters:
  juvenile_age:
    value: 20
  adult_age:
    value: 'random_uniform(50, 70)'
```

Note

- State `D` with `autoremove: yes` represents the state of
  deceased individuals: all individuals sent to `D` are
  automatically removed from simulation. On the state machine
  diagram, this state appears as a dotted box.
- Section `productions` in `state_machines`: production links
  specify from which state groups new individuals are
  produced. Here for instance all health states are able to produce
  newborns. On the diagram, productions links appears as dashed
  arrows.

See also

Set states attributes,
Produce new individuals

## 6.6. SIR model with age groups¶

Files

- compart\_SIR\_JA\_demo.yaml
- hybrid\_SIR\_JA\_demo.yaml
- IBM\_SIR\_JA\_demo.yaml

digraph {
rankdir=LR;
node[fontsize=16, fontname=Arial, shape=box, style="filled,rounded"];
edge[minlen=1.5, penwidth=1.5, tailtooltip="", headtooltip=""];
"S" [shape="Mrecord", label="S", tooltip="S (Susceptible):
suceptible of becoming infected", fillcolor=wheat, style="filled,rounded,bold"] ;
"I" [shape="Mrecord", label="I", tooltip="I (Infectious):
infected and able to transmit the disease", fillcolor=maroon, style="filled,rounded"] ;
"R" [shape="Mrecord", label="R", tooltip="R (Resistant):
healthy again and resistant to infection", fillcolor=deepskyblue, style="filled,rounded"] ;
"I" -> "R" [label="rate: recovery", labeltooltip="recovery [parameter]:
recovery rate (/day)
0.1", arrowtail="none", arrowhead="normalnone", dir=both, tooltip="", minlen=3, style="solid"];
"S" -> "I" [label="rate: force\_of\_infection", labeltooltip="force\_of\_infection [parameter]:
infection function
transmission\_I \* total\_I / total\_herd", arrowtail="none", arrowhead="normalnone", dir=both, tooltip="", minlen=3, style="solid"];
}

digraph {
rankdir=LR;
node[fontsize=16, fontname=Arial, shape=box, style="filled,rounded"];
edge[minlen=1.5, penwidth=1.5, tailtooltip="", headtooltip=""];
"J" [shape="Mrecord", label="J", tooltip="J (Juvenile):
juvenile animals", fillcolor=orange, style="filled,rounded"] ;
"A" [shape="Mrecord", label="A", tooltip="A (Adult):
adult animals (producing new juveniles)", fillcolor=brown, style="filled,rounded"] ;
"D" [shape="Mrecord", label="D", tooltip="D (Dead):
compartment to put dead animals", fillcolor=white, style="filled,rounded,dotted"] ;
"A" -> "D" [label="rate: death", labeltooltip="death [parameter]:
the death rate (/day)
birth", arrowtail="none", arrowhead="normalnone", dir=both, tooltip="", minlen=3, style="solid"];
"A" -> "J" [label="rate: birth", labeltooltip="birth [parameter]:
the birth rate (/day)
0.01", arrowtail="none", arrowhead="normalnone", dir=both, tooltip="", minlen=3, style="dashed"];
"J" -> "A" [label="rate: maturation", labeltooltip="maturation [parameter]:
rate at which juveniles become adults (/day)
1/20", arrowtail="none", arrowhead="normalnone", dir=both, tooltip="", minlen=3, style="solid"];
"J" -> "D" [label="rate: death", labeltooltip="death [parameter]:
the death rate (/day)
birth", arrowtail="none", arrowhead="normalnone", dir=both, tooltip="", minlen=3, style="solid"];
}

Note

- **Two state machines** and corresponding groupings/processes are
  defined: one for the progression of infection (`health_state`),
  the other for the maturation of individuals and demography
  (`age_group`).
- State `S` with `default: yes` (hence, in bold on the diagram)
  is now chosen without further indication:
  - in compart\_SIR\_JA\_demo.yaml new individuals produced by
    adults are automatically considered `S`: conversely, since no
    default state was defined for `age_group` and initial
    conditions only specify initial repartition in health states,
    individuals are automatically distributed in `J` and `A` (not
    in `D` since it is an autoremove state) with equal
    probability.
  - in hybrid\_SIR\_JA\_demo.yaml and IBM\_SIR\_JA\_demo.yaml,
    prototype `newborn` uses `default` for initializing
    `health_state` when creating new individuals.
- In hybrid\_SIR\_JA\_demo.yaml and IBM\_SIR\_JA\_demo.yaml,
  prototypes now initialize the values for both state
  machines. Especially, for prototypes used in initial conditions,
  age groups are set to `random`, which means an equiprobable
  choice between states of state machine `age_group` (except
  autoremove states).

See also

Set states attributes

## 6.7. SIR model with age groups and random initialization¶

Files

- hybrid\_SIR\_JA\_demo\_random.yaml
- IBM\_SIR\_JA\_demo\_random.yaml

Note

- Prototypes `healthy` and `infected` define the `age_group`
  using a multinomial sampling between possible states (`J` and
  `A`), to ensure a given proportion of juveniles among healthy
  individuals in average:

  ```
  prototypes:
      individuals:
        - healthy:
            desc: 'healthy individuals'
            health_state: S
            age_group: 'random(init_prop_juveniles, 1-init_prop_juveniles)'
  ```
- Similarly, initial conditions use a multinomial sampling between
  two prototypes (`healthy` and `indfected`) to make the
  initial prevalence vary stochastically according to the initial
  probability of being infected (`initial_proba_infection`):

  ```
  initial_conditions:
    population:
      - prototype: [infected, healthy]
        # two possible prototypes for individual initially in the population
        amount: initial_population_size
        # drawn among a population of size initial_population_size
        proba: [initial_proba_infection, 1-initial_proba_infection]
        # with respective probabilities initial_proba_infection and
        # 1-initial_proba_infection
  ```

See also

Design prototypes for typical individuals or populations

## 6.8. SIR model with cumulative incidence¶

Files

- compart\_SIR\_cumul\_inc.yaml
- hybrid\_SIR\_cumul\_inc.yaml
- IBM\_SIR\_cumul\_inc.yaml

Note

Cumulative incidence is obtained through the built-in action
`record_change` executed when entering infecious state. This
action adds the number of individuals currently executing the action
to a variable, here `cumulative_incidence`.

See also

Built-in actions

## 6.9. SIR model with individual actions and variable aggregation¶

Files

- hybrid\_SIR\_aggreg.yaml
- IBM\_SIR\_aggreg.yaml

digraph {
rankdir=LR;
node[fontsize=16, fontname=Arial, shape=box, style="filled,rounded"];
edge[minlen=1.5, penwidth=1.5, tailtooltip="", headtooltip=""];
"S" [shape="Mrecord", label="{&#9670;|{\ S\ }}", tooltip="S (Susceptible):
suceptible of becoming infected", fillcolor=wheat, style="filled,rounded"] ;
"I" [shape="Mrecord", label="{&#9670;|{\ I\ |&#9670;}}", tooltip="I (Infectious):
infected and able to transmit the disease", fillcolor=maroon, style="filled,rounded"] ;
"R" [shape="Mrecord", label="R", tooltip="R (Resistant):
healthy again and resistant to infection", fillcolor=deepskyblue, style="filled,rounded"] ;
"I" -> "R" [label="rate: recovery", labeltooltip="recovery [parameter]:
recovery rate (/day)
0.1", arrowtail="none", arrowhead="normalnone", dir=both, tooltip="", minlen=3, style="solid"];
"S" -> "I" [label="rate: force\_of\_infection", labeltooltip="force\_of\_infection [parameter]:
infection function
transmission\_I \* total\_I / total\_herd", arrowtail="none", arrowhead="normalnone", dir=both, tooltip="", minlen=3, style="solid"];
}

Note

- On state machine diagrams, black lozenges (left to, below or
  right to the state name) mark the specification of actions
  (respectively on enter, on stay and on exit).
- Look at messages in the terminal (beware, printing messages
  increases computation time!)
- Variables at population scale are created by aggregating
  variables at individual scale.

See also

Built-in actions,
Aggregate variables

## 6.10. SIR model with age groups and explicit age¶

Files

- hybrid\_SIR\_age\_demo.yaml
- IBM\_SIR\_age\_demo.yaml

digraph {
rankdir=LR;
node[fontsize=16, fontname=Arial, shape=box, style="filled,rounded"];
edge[minlen=1.5, penwidth=1.5, tailtooltip="", headtooltip=""];
"S" [shape="Mrecord", label="S", tooltip="S (Susceptible):
suceptible of becoming infected", fillcolor=wheat, style="filled,rounded,bold"] ;
"I" [shape="Mrecord", label="I", tooltip="I (Infectious):
infected and able to transmit the disease", fillcolor=maroon, style="filled,rounded"] ;
"R" [shape="Mrecord", label="R", tooltip="R (Resistant):
healthy again and resistant to infection", fillcolor=deepskyblue, style="filled,rounded"] ;
"I" -> "R" [label="rate: recovery", labeltooltip="recovery [parameter]:
recovery rate (/day)
0.1", arrowtail="none", arrowhead="normalnone", dir=both, tooltip="", minlen=3, style="solid"];
"S" -> "I" [label="rate: force\_of\_infection", labeltooltip="force\_of\_infection [parameter]:
infection function
transmission\_I \* total\_I / total\_herd", arrowtail="none", arrowhead="normalnone", dir=both, tooltip="", minlen=3, style="solid"];
}

digraph {
rankdir=LR;
node[fontsize=16, fontname=Arial, shape=box, style="filled,rounded"];
edge[minlen=1.5, penwidth=1.5, tailtooltip="", headtooltip=""];
"J" [shape="Mrecord", label="{{\ J\ |&#9670;}}", tooltip="J (Juvenile):
juvenile animals", fillcolor=orange, style="filled,rounded"] ;
"A" [shape="Mrecord", label="{{\ A\ |&#9670;}}", tooltip="A (Adult):
adult animals (producing new juveniles)", fillcolor=brown, style="filled,rounded"] ;
"D" [shape="Mrecord", label="D", tooltip="D (Dead):
compartment to put dead animals", fillcolor=white, style="filled,rounded,dotted"] ;
"A" -> "D" [label="rate: 1
───────────────────
avg\_adult\_death\_age", labeltooltip="rate: 1
───────────────────
avg\_adult\_death\_age", arrowtail="none", arrowhead="normalnone", dir=both, tooltip="", minlen=3, style="solid"];
"A" -> "J" [label="rate: birth", labeltooltip="birth [parameter]:
the birth rate (/day)
0.01", arrowtail="none", arrowhead="normalnone", dir=both, tooltip="", minlen=3, style="dashed"];
"J" -> "A" [label="proba: 1", labeltooltip="proba: 1", arrowtail="nonetee", arrowhead="normalnone", dir=both, tooltip="COND: GreaterThan(age, adult\_age)", minlen=3, style="solid"];
}

```
state_machines:
  age_group:
    - J:
        ...
        on_stay:
          - set_var: age
            value: 'age + delta_t'
    - A:
        ...
        on_stay:
          - set_var: age
            value: 'age + delta_t'
    ...
  transitions:
    - from: J
      to: A
      cond: 'GreaterThan(age, adult_age)'
      proba: 1
```

Note

- `cond` in transition from J to A: since ages are distributed
  randomly within the limits of J group, becoming adult does not rely
  on a duration but on an explicit verification of age. (An
  alternative would be to define a duration as the difference
  between adult age and individual age.)
- `statevars` section to declare variable `age`, which is
  initialized in prototypes, modified by actions in J and A states,
  and used to define the condition in transition from J to A.
- `max_age_A_I` automatically provides the greatest age in
  infected adults in hybrid model (due to grouping `details`) but
  is not available in IBM (due to the absence of grouping).

See also

Customize transitions,
Built-in actions,
Aggregate variables

## 6.11. SIR model with explicit gestation¶

Files

- hybrid\_gest.yaml
- IBM\_gest.yaml

digraph {
node[fontsize=16, fontname=Arial, shape=box, style="filled,rounded"];
edge[minlen=1.5, penwidth=1.5, tailtooltip="", headtooltip=""];
"Female" [shape="Mrecord", label="Female", tooltip="Female (Female animals):
", fillcolor=orange, style="filled,rounded,bold"] ;
"Male" [shape="Mrecord", label="Male", tooltip="Male (Male animals):
", fillcolor=deepskyblue, style="filled,rounded"] ;
}

digraph {
charset="utf-8"
rankdir=TB;
node[fontsize=16, fontname=Arial, shape=box, style="filled,rounded"];
edge[minlen=1.5, fontname=Times, penwidth=1.5, tailtooltip="", headtooltip=""];
"P0" [shape="Mrecord", label="P0", tooltip="P0 (Nulliparous):
Nulliparous animal (no previous farrowing)", fillcolor=orange, style="filled,rounded"] ;
"P1" [shape="Mrecord", label="P1", tooltip="P1 (Parity 1):
Animal with parity 1 (first farrowing)", fillcolor=pink, style="filled,rounded"] ;
"P2" [shape="Mrecord", label="P2", tooltip="P2 (Parity 2):
Animal with parity 2", fillcolor=red, style="filled,rounded"] ;
"P3" [shape="Mrecord", label="P3", tooltip="P3 (Parity 3+):
Animal with parity 3 and more", fillcolor=purple, style="filled,rounded"] ;
}

digraph {
charset="utf-8"
rankdir=LR;
node[fontsize=16, fontname=Arial, shape=box, style="filled,rounded"];
edge[minlen=1.5, fontname=Times, penwidth=1.5, tailtooltip="", headtooltip=""];
"J" [shape="Mrecord", label="{{\ J\ |&#9670;}}", tooltip="J (Juvenile):
juvenile animals", fillcolor=orange, style="filled,rounded"] ;
"A" [shape="Mrecord", label="{{\ A\ |&#9670;}}", tooltip="A (Non-gestating Adult):
adult animals", fillcolor=brown, style="filled,rounded"] ;
"G" [shape="Mrecord", label="{{\ G&nbsp;&#9719;\ |&#9670;}}", tooltip="G (Gestating Adult):
adult animals (producing new juveniles)", fillcolor=deepskyblue, style="filled,rounded"] ;
"D" [shape="Mrecord", label="D", tooltip="D (Dead):
compartment to put dead animals", fillcolor=white, style="filled,rounded,dotted"] ;
"A" -> "D" [label="rate: 1 / avg\_adult\_death\_age", labeltooltip="rate: 1 / avg\_adult\_death\_age", arrowtail="none", arrowhead="normalnone", dir=both, tooltip="", minlen=3, style="solid"];
"A" -> "G" [label="rate: reproduction", labeltooltip="reproduction [parameter]:
reproduction rate (/days)
1/20", arrowtail="nonetee", arrowhead="normalnone", dir=both, tooltip="COND: is\_Female", minlen=3, style="solid"];
"G" -> "A" [label="proba: 1", labeltooltip="proba: 1", arrowtail="none", arrowhead="normalnonediamond", dir=both, tooltip="", minlen=3, style="solid"];
"G" -> "D" [label="rate: 1 / avg\_adult\_death\_age", labeltooltip="rate: 1 / avg\_adult\_death\_age", arrowtail="noneoinv", arrowhead="normalnone", dir=both, tooltip="ESCAPE: True", minlen=3, style="solid"];
"J" -> "A" [label="proba: 1", labeltooltip="proba: 1", arrowtail="nonetee", arrowhead="normalnone", dir=both, tooltip="COND: GreaterThan(age, adult\_age)", minlen=3, style="solid"];
}

Note

- the use of state machines `sex` and `parity` which have only
  states (without any transition) to represent discrete values
- condition `is_Female` to allow only females to move to
  gestating state
- built-in action `produce_offspring` used when returning from
  gestating (G) to non-gestating (A) state
- keyword `next_state` for variable `parity` in prototype
  `next_parity`, applied when returning from G to A, to make the
  value change automatically to the “next” one (from P0 to P1, from
  P1 to P2, etc.)
- `escape` condition on transition from G to D to allow mortality
  while being in gestating state (otherwise the `duration` would
  forbid to leave gestating state before the end!)
- `total_G_Female` automatically provides the number of females
  in gestating state (which should be exactly `total_G`) in
  hybrid model, but is not available in IBM (due to the absence of
  grouping).

See also

Customize transitions,
State machines without transitions,
Produce new individuals,
Design prototypes for typical individuals or populations

A very complete example is also provided in the Quickstart model
(`quickstart/quickstart.yaml`) used to Test your installation.

Especially:

- action `produce_offspring` used to model vertical transmission
- `escape` condition to allow abortions before gestation has ended

## 6.12. SIR model with structured population¶

Files

hybrid\_SIR\_struct.yaml

digraph {
rankdir=LR;
node[fontsize=16, fontname=Arial, shape=box, style="filled,rounded"];
edge[minlen=1.5, penwidth=1.5, tailtooltip="", headtooltip=""];
"S" [shape="Mrecord", label="S", tooltip="S (Susceptible):
suceptible of becoming infected", fillcolor=wheat, style="filled,rounded,bold"] ;
"I" [shape="Mrecord", label="I", tooltip="I (Infectious):
infected and able to transmit the disease", fillcolor=maroon, style="filled,rounded"] ;
"R" [shape="Mrecord", label="R", tooltip="R (Resistant):
healthy again and resistant to infection", fillcolor=deepskyblue, style="filled,rounded"] ;
"I" -> "R" [label="rate: recovery", labeltooltip="recovery [parameter]:
recovery rate (/day)
0.1", arrowtail="none", arrowhead="normalnone", dir=both, tooltip="", minlen=3, style="solid"];
"S" -> "I" [label="rate: force\_of\_infection", labeltooltip="force\_of\_infection [parameter]:
infection function
is\_J \* force\_of\_infection\_juveniles + is\_A \* force\_of\_infection\_adults ", arrowtail="none", arrowhead="normalnone", dir=both, tooltip="", minlen=3, style="solid"];
}

digraph {
rankdir=LR;
node[fontsize=16, fontname=Arial, shape=box, style="filled,rounded"];
edge[minlen=1.5, penwidth=1.5, tailtooltip="", headtooltip=""];
"J" [shape="Mrecord", label="J&nbsp;&#9719;", tooltip="J (Juvenile):
Young animals", fillcolor=orange, style="filled,rounded,bold"] ;
"A" [shape="Mrecord", label="A", tooltip="A (Adult):
Adult animals", fillcolor=brown, style="filled,rounded"] ;
"D" [shape="Mrecord", label="D", tooltip="D (Dead):
compartment to represent deceased animals", fillcolor=white, style="filled,rounded,dotted"] ;
"A" -> "D" [label="rate: mortality", labeltooltip="mortality [parameter]:
density-dependent mortality rate
b \* total\_herd / carrying\_capacity", arrowtail="none", arrowhead="normalnone", dir=both, tooltip="", minlen=3, style="solid"];
"A" -> "J" [label="rate: birth", labeltooltip="birth [parameter]:
birth rate (/day)
b \* 2", arrowtail="none", arrowhead="normalnone", dir=both, tooltip="", minlen=3, style="dashed"];
"J" -> "A" [label="proba: 1", labeltooltip="proba: 1", arrowtail="none", arrowhead="normalnone", dir=both, tooltip="", minlen=3, style="solid"];
"J" -> "D" [label="rate: mortality", labeltooltip="mortality [parameter]:
density-dependent mortality rate
b \* total\_herd / carrying\_capacity", arrowtail="noneoinv", arrowhead="normalnone", dir=both, tooltip="ESCAPE: True", minlen=3, style="solid"];
}

```
grouping:
  population:
    infection:
      machine_name: health_state
      # grouping for infection now relies on two variables: the health
      # state but also the age_group since the value of variables
      # is_A/is_J used in the force of infection is determined by the
      # age group
      key_variables: [health_state, age_group]
    aging:
      machine_name: age_group
      key_variables: [age_group]
...
parameters:
  force_of_infection:
    desc: 'infection function'
    value: 'is_J * force_of_infection_juveniles + is_A * force_of_infection_adults'
  force_of_infection_juveniles:
    desc: 'infection function experience by juveniles'
    value: 'transJJ * total_I_J / (total_J + 1e-9) + transAJ * total_I_A / (total_A + 1e-9)'
  force_of_infection_adults:
    desc: 'infection function experience by adults'
    value: 'transAA * total_I_A / (total_A + 1e-9) + transJA * total_I_J / (total_J + 1e-9)'
```

Note

- The grouping used in infection process
- The composite definition of the force of infection, based on
  booleans (`is_J`, `is_A`) and on the force of infection
  experienced by each group

See also

Complexify grouping,
Automatic variables

## 6.13. SIR model with metapopulation¶

Files

- hybrid\_SIR\_metapop.yaml
- compart\_SIR\_metapop.yaml

```
levels:
  population:
  ...
  metapop:
    desc: 'level of the metapopulation'
    contains:
      - population
    aggregation_type: metapopulation
...
prototypes:
  population:
    - healthy_population:
        desc: 'population initially without infected individuals'
        initial_prevalence: 0
    - infected_population:
        desc: 'population with initially infected individuals'
        initial_prevalence: 0.1
...
initial_conditions:
  population:
  ...
  metapop:
    - prototype: infected_population
      amount: 1
    - prototype: healthy_population
      amount: 'nb_populations - 1'
```

Note

- The additional level with aggregation type `metapopulation`
- Additional prototypes at population level
- Initial conditions now apply not only at population level, but also at
  metapop level

See also

Changing scale: metapopulations

## 6.14. SIR model with metapopulation and data-driven movements¶

Files

hybrid\_SIR\_metapop\_data.yaml +
hybrid\_SIR\_metapop\_data.py

```
levels:
  ...
  metapop:
    desc: 'level of the metapopulation'
    contains:
      - herd
    aggregation_type: metapopulation
    # The metapopulation is explicitly linked to a specific class in a
    # Python code add-on
    file: hybrid_SIR_metapop_data.py
    class_name: Metapopulation
...
processes:
  herd:
    ...
    metapop:
      # here the process is the name of a procedure defined in the
      # Python code
      - exchange_animals
```

```
class Metapopulation(MetapopProcessManager):
    """ level of the metapop. """

    def initialize_level(self, **others):
         """Initialize an instance of Metapopulation. Here, load
         data from CSV file."""
         ...

    def exchange_animals(self):
         """ Definition of process 'exchange_animals'. """
         ...
```

Note

- Reference to Python file and class name in the definition of
  level `metapop`
- Class name `Metapopulation` which links level `metapop` from
  YAML file to actual code in Python file.
- New prototype `imported_movement` for creating animals coming
  from outside the metapopulation
- Variables `purchased` and `sold` which are updated in Python
  code by process `exchange_animals`

See also

Changing scale: metapopulations,
Connecting to Python code add-ons

# EMULSION

Epidemiological Multi-Level Simulation Framework

### Navigation

- 1. Installation
- 2. Getting started with EMULSION
- 3. Modelling principles
- 4. Modelling language (basics)
- 5. Modelling language (advanced)
- 6. Feature examples
  - 6.1. SIR model
  - 6.2. SEIRS model
  - 6.3. SIRS model with periodic external risk
  - 6.4. Custom state durations
  - 6.5. SIR model with basic demography (births/deaths)
  - 6.6. SIR model with age groups
  - 6.7. SIR model with age groups and random initialization
  - 6.8. SIR model with cumulative incidence
  - 6.9. SIR model with individual actions and variable aggregation
  - 6.10. SIR model with age groups and explicit age
  - 6.11. SIR model with explicit gestation
  - 6.12. SIR model with structured population
  - 6.13. SIR model with metapopulation
  - 6.14. SIR model with metapopulation and data-driven movements
- 7. Information
- 8. License
- 9. High-level functions for model designers
- 10. emulsion package

### Related Topics

- Documentation overview
  - Previous: 5. Modelling language (advanced)
  - Next: 7. Information

### Quick search

©2016, INRA and Univ. Lille.
|
Powered by Sphinx 1.8.5
& Alabaster 0.7.10
|
Page source
