# Supplementary material for: EMULSION: Transparent and flexible multiscale stochastic models in human, animal and plant epidemiology
Source: PLoS Comput Biol. 2019 Sep 13;15(9):e1007342. doi: 10.1371/journal.pcbi.1007342 (PMC6760811; doi:10.1371/journal.pcbi.1007342)
Supplement: S2 File — This zip file is a clone of EMULSION public Git repository. To install from this file rather than from PyPI, go to EMULSION documentation page: https://sourcesup.renater.fr/www/emulsion-public/pages/Install.html and follow Git-based installation instructions. (ZIP) [file pcbi.1007342.s010.zip › S2_file/doc/pages/Getting_started.html]

2. Getting started with EMULSION — EMULSION (Epidemiological Multi-Level Simulation framework)

# 2. Getting started with EMULSION¶

We assume in what follows that you have downloaded and unzipped the
models directory provided
on EMULSION website.

## 2.1. Running EMULSION¶

To simulate an EMULSION model (named e.g. `MODEL`), open a terminal
in the directory where your files are located, and type the following
command:

```
emulsion run --plot MODEL.yaml
```

Option `--plot` automatically opens your web browser to show
simulation outcomes. To view diagrams representing model structure,
append option `--view-model` to the previous line.

As an example, open a terminal in the directory `models/features` and type:

```
emulsion run --plot compart_SIR.yaml --view-model
```

The simulation results will pop up in your web browser:

digraph {
rankdir=LR;
node[fontsize=16, fontname=Arial, shape=box, style="filled,rounded"];
edge[minlen=1.5, penwidth=1.5, tailtooltip="", headtooltip=""];
"S" [shape="Mrecord", label="S", tooltip="S (Susceptible):
suceptible of becoming infected", fillcolor=wheat, style="filled,rounded"] ;
"I" [shape="Mrecord", label="I", tooltip="I (Infectious):
infected and able to transmit the disease", fillcolor=maroon, style="filled,rounded"] ;
"R" [shape="Mrecord", label="R", tooltip="R (Resistant):
healthy again and resistant to infection", fillcolor=deepskyblue, style="filled,rounded"] ;
"I" -> "R" [label="rate: recovery", labeltooltip="recovery [parameter]:
recovery rate (/day)
0.1", arrowtail="none", arrowhead="normalnone", dir=both, tooltip="", minlen=3, style="solid"];
"S" -> "I" [label="rate: force\_of\_infection", labeltooltip="force\_of\_infection [parameter]:
infection function
transmission\_I \* total\_I / total\_herd", arrowtail="none", arrowhead="normalnone", dir=both, tooltip="", minlen=3, style="solid"];
}

You can also check the results of a more complex model, e.g. `quickstart`,
tested at install.

When running a model, EMULSION:

> 1. Reads file `MODEL.yaml` which describes the model
> 2. With options `--view-model`, builds the diagrams representing
>    model structure and stores them in the figure directory
>    (default: `img/`)
> 3. Runs *N* (default: 10) stochastic repetitions of model simulation during
>    *T* (default: 100) time steps
> 4. Stores simulation outputs in a CSV file `counts.csv` located
>    in the output directory (default: `outputs/`)
> 5. With option `--plot`, produces a web page with a basic
>    representation of the outputs (and model structure if
>    `--view-model` was also set) which is stored in the figure
>    directory, under the name `My_model_name.html`
>    (`My_model_name` being the name of the model as defined in
>    `MODEL.yaml` by the instruction: `model_name:
>    My_model_name`)

Most frequently used options are:

`-r N`
:   produce *N* stochastic repetitions

`-t T`
:   run the simulation for *T* time steps

`--silent`
:   show a progress bar only for the progression of repetitions
    (instead of a progress bar per repetition).

`--output-dir OUTPUT`
:   change output directory (the location of `counts.csv` produced
    by the simulation) to `OUTPUT`

`--figure-dir FIG`
:   change figure directory (the location of model diagrams and
    outputs web page) to `FIG`

(Other options are available with `emulsion -h`.)

## 2.2. Producing model diagrams¶

When running a model, you can use options `--plot` and
`--view-model` to plot simulation outcomes and model diagrams,
respectively.

To generate model diagrams without having to run the model, just type
for instance:

```
emulsion diagrams compart_SIR.yaml
```

By default, diagrams are generated in SVG format (in the figure
directory, by default `img`). You can change format to PNG or PDF
with option `--format`, e.g.:

```
emulsion diagrams compart_SIR.yaml --format pdf
```

## 2.3. Viewing parameters¶

EMULSION command can also show the actual values of model parameters:

```
emulsion show compart_SIR.yaml
```

in the following list (“MODEL parameters”):

```
                      EmulsionModel "compart_SIR"
            AVAILABLE PARAMETERS (with their current value)
------------------------------------------------------------------------
MODEL PARAMETERS
------------------------------------------------------------------------
  total_duration.................................................100.0
  initial_herd_size..............................................100.0
  initial_infected.................................................1.0
  transmission_I...................................................0.5
  recovery.........................................................0.1
  delta_t..........................................................1.0
------------------------------------------------------------------------
```

You can also ask for the role of the parameters:

```
emulsion describe compart_SIR.yaml recovery delta_t transmission_I
```

results in the following list (“MODEL parameters”):

```
                      EmulsionModel "compart_SIR"
              ROLE OF PARAMETERS (AND CURRENT DEFINITION)
------------------------------------------------------------------------
recovery [parameter]:
	recovery rate (/day)
	0.1
delta_t [parameter]:
	duration of the simulation time step
	1.0
transmission_I [parameter]:
	transmission rate from infectious animals (/day)
	0.5
------------------------------------------------------------------------
```

## 2.4. Changing parameters¶

Now, to run the model with other parameter values without changing the
model file, you can specify the new values with option `-p
PARAM=VALUE` (one for each parameter to change), for instance:

```
emulsion run --plot compart_SIR.yaml -p transmission_I=0.3 -p recovery=0.01
```

which produces:

## 2.5. Changing the model¶

To change model structure and assumptions, open the file in a text
editor. For instance, to change the above SIR model into a SEIRS
model, open `compart_SIR.yaml` and save it as a new file,
e.g. `compart_SEIRS.yaml`. You have to add a state (E), modify
existing transitions and add new ones, and introduce new parameters
(to represent the incubation and the waning of immunity).

1. Add a latent (or exposed) state: search for the `health_state`
   state machines and change the states and transitions as follows:

   ```
      state_machines:
        health_state:
          desc: 'The state machine which defines the evolution of health states'
          states:
            - S:
              name: 'Susceptible'
              desc: 'suceptible of becoming infected'
              fillcolor: 'deepskyblue'
            - E:
              name: 'Latent'
              desc: 'infected but not yet able to transmit the disease'
              fillcolor: 'orange'
            - I:
              name: 'Infectious'
              desc: 'infected and able to transmit the disease'
              fillcolor: 'red'
            - R:
              name: 'Resistant'
              desc: 'healthy again and resistant to infection'
              fillcolor: 'limegreen'
          transitions:
            - {from: S, to: E, rate: 'force_of_infection'}
            - {from: E, to: I, rate: '1/incubation'}
            - {from: I, to: R, rate: 'recovery'}
   ```
2. Add the waning of immunity simply by adding the following
   transition:

   ```
   - {from: R, to: S, rate: 'waning'}
   ```
3. Add the new parameters to the `parameters` section:

   ```
   parameters:
     ...
     incubation:
       desc: 'mean duration of latent state (days, assuming exponential
       distribution of durations in the state)
       '
       value: 5
     waning:
       desc: 'rate at which individuals lose immunity (/day)'
       value: 0.05
   ```
4. Do not forget to update the calculation of prevalence, which is an
   expression also defined in the `parameters` section:

   ```
   parameters:
     ...
     'prevalence (%)':
       desc: 'proportion of infected+infectious individuals'
       value: '100 * (total_I + total_E) / total_population'
   ```

You’re done ! Test your model with `emulsion` command as in previous
examples:

```
emulsion run --plot compart_SEIRS.yaml --view-model --silent
```

which produces also a new diagram:

digraph {
rankdir=LR;
node[fontsize=16, fontname=Arial, shape=box, style="filled,rounded"];
edge[minlen=1.5, penwidth=1.5, tailtooltip="", headtooltip=""];
"S" [shape="Mrecord", label="S", tooltip="S (Susceptible):
suceptible of becoming infected", fillcolor=wheat, style="filled,rounded"] ;
"E" [shape="Mrecord", label="E", tooltip="E (Latent):
infected but not yet able to transmit the disease", fillcolor=orange, style="filled,rounded"] ;
"I" [shape="Mrecord", label="I", tooltip="I (Infectious):
infected and able to transmit the disease", fillcolor=maroon, style="filled,rounded"] ;
"R" [shape="Mrecord", label="R", tooltip="R (Resistant):
healthy again and resistant to infection", fillcolor=deepskyblue, style="filled,rounded"] ;
"E" -> "I" [label="rate: 1
──────────
incubation", labeltooltip="rate: 1
──────────
incubation", arrowtail="none", arrowhead="normalnone", dir=both, tooltip="", minlen=3, style="solid"];
"I" -> "R" [label="rate: recovery", labeltooltip="recovery [parameter]:
recovery rate (/day)
0.1", arrowtail="none", arrowhead="normalnone", dir=both, tooltip="", minlen=3, style="solid"];
"R" -> "S" [label="rate: waning", labeltooltip="waning [parameter]:
rate at which individuals lose immunity (/day)
0.05", arrowtail="none", arrowhead="normalnone", dir=both, tooltip="", minlen=3, style="solid"];
"S" -> "E" [label="rate: force\_of\_infection", labeltooltip="force\_of\_infection [parameter]:
infection function
transmission\_I \* total\_I / total\_herd", arrowtail="none", arrowhead="normalnone", dir=both, tooltip="", minlen=3, style="solid"];
}

## 2.6. Going further…¶

Check our examples

EMULSION provides a large number of modelling features. To go
further on using EMULSION to design models according to your needs
(even complex), jump to next sections:

- Modelling principles to get insights on key EMULSION concepts
- Modelling language (basics) to have an overview on how
  EMULSION models are structured and to be able to write your first
  models
- Modelling language (advanced) to learn more on EMULSION
  features
- Feature examples to discover representative
  epidemiological models and how they are handled with EMULSION.

# EMULSION

Epidemiological Multi-Level Simulation Framework

### Navigation

- 1. Installation
- 2. Getting started with EMULSION
  - 2.1. Running EMULSION
  - 2.2. Producing model diagrams
  - 2.3. Viewing parameters
  - 2.4. Changing parameters
  - 2.5. Changing the model
  - 2.6. Going further…
- 3. Modelling principles
- 4. Modelling language (basics)
- 5. Modelling language (advanced)
- 6. Feature examples
- 7. Information
- 8. License
- 9. High-level functions for model designers
- 10. emulsion package

### Related Topics

- Documentation overview
  - Previous: 1. Installation
  - Next: 3. Modelling principles

### Quick search

©2016, INRA and Univ. Lille.
|
Powered by Sphinx 1.8.5
& Alabaster 0.7.10
|
Page source
