# Supplementary material for: EMULSION: Transparent and flexible multiscale stochastic models in human, animal and plant epidemiology
Source: PLoS Comput Biol. 2019 Sep 13;15(9):e1007342. doi: 10.1371/journal.pcbi.1007342 (PMC6760811; doi:10.1371/journal.pcbi.1007342)
Supplement: S2 File — This zip file is a clone of EMULSION public Git repository. To install from this file rather than from PyPI, go to EMULSION documentation page: https://sourcesup.renater.fr/www/emulsion-public/pages/Install.html and follow Git-based installation instructions. (ZIP) [file pcbi.1007342.s010.zip › S2_file/doc/pages/Modelling_language_advanced.html]

5. Modelling language (advanced) — EMULSION (Epidemiological Multi-Level Simulation framework)

# 5. Modelling language (advanced)¶

EMULSION modelling language allows model designers to specify complex
assumptions in a readable way, to foster interactions with other
scientists and facilitate model revision throughout the design process
without having to dive into the simulation code.

Most language features documented below are associated with example
files, located in the `models/features` directory.

## 5.1. Compartments, IBM or hybrid models?¶

EMULSION helps to transform compartment-based models into
invidual-based models (or vice-versa, assuming the individual-based
model can get rid of individual specificities). An intermediate
approach is the hybrid model, which preserves individual
characteristics but drives system evolution through compartment-like
groupings (see: Individuals, populations, metapopulations).

The table below summarises the main differences between each kind of
models, regarding the sections and keywords to modify in an EMULSION
model file.

| EMULSION section | section keyword | Modelling paradigm | | |
| --- | --- | --- | --- | --- |
|  |  | compartment | hybrid | IBM |
| `levels` | `aggregation_type` | `compartment` | `hybrid` | `IBM` |
|  | `contains` | *not used* | *sublevels* | |
| `grouping` | `key_variables` | *list of variables* | | *not used* |
|  | `state_machine` | *machine name* (optional) | |  |
| `processes` | *level* | *population* | | *individuals* |
|  | *list of processes* | *names of groupings* | | *machine name* |
| `state_machines` | `productions` |  | *based on prototypes* | |
| `prototypes` |  | *not used* | *for individuals/populations* | |
| `initial_conditions` |  | *per state* | *based on prototypes* | |

See also

Also, in `model/features`, many examples are provided with the
three modelling paradigms when possible, especially the simplest
SIR model (`compart_SIR.yaml`, `hybrid_SIR.yaml`,
`IBM_SIR.yaml`) and the corresponding version with demographic
processes (`compart_SIR_demo.yaml`, `hybrid_SIR_demo.yaml`,
`IBM_SIR_demo.yaml`).

See SIR model, SEIRS model, SIR model with basic demography (births/deaths) and many others in Feature examples.

## 5.2. Master state machines¶

### Set states attributes¶

States can be endowed with *attributes*. For instance, `fillcolor`
defines the color of the boxes on state machine diagrams, which is the
same than the color of plots in outputs.

A state can define three main properties:

`autoremove: yes`
:   Autoremove states are intended as “sink”, so that all individuals
    that reach such a state are removed from the system. This is a
    very convenient way to represent deaths or outgoing commercial
    movements.

    See also

    SIR model with basic demography (births/deaths)

`default: yes`
:   A state can be labelled as “default state”. Default state plays
    the same role but the way to use them differs in compartment-based
    models and in others:

    - in compartment-based models, when no indication is provided in
      initial conditions or production links regarding new
      individuals, they will be put in the default state for each of
      their state machines
    - in IBM/hybrid models and metapopulations, `default` is a valid
      value for prototypes definition. For instance, writing
      `health_state: default` in a prototype will set the health
      state to the default state of the `health_state` state machine

      See also

      Default state, SIR model with age groups, Design prototypes for typical individuals or populations

`next: AnotherState` / `previous: AnotherState`
:   A state, especially when used in a state machine without
    transitions (e.g. representing a set of discrete states which are
    driven by another process, such as animal parities) can specify
    explicitly a predecessor/successor relation with other states from
    the same state machine. This can be quite useful to induce
    incremental changes in a state without knowing its value, by using
    the `next_state` and `previous_state` keywords in prototypes.

    See also

    SIR model with explicit gestation

`duration` (valid for **IBM**/**hybrid** models)
:   This keyword allows to assign a specific duration to a
    state. Durations can be constant values or arbitrary expressions,
    including a random distribution. When an individual enters the
    state, it receives a value calculated from this expression
    (possibly involving a random sample) and cannot leave this state
    until the duration is over (with the exception of escape
    conditions).

    See also

    Custom state durations

`on_enter`, `on_stay`, `on_exit` (valid for **IBM**/**hybrid** models)
:   These keywords are used to specify lists of **actions** that
    individuals have to perform when, respectively, entering, staying
    in, or leaving the state.

    Actions can be either built-in actions (listed there) or the name of a function provided in a code
    add-on (e.g. `action: my_custom_function`).

    See also

    SIR model with individual actions and variable aggregation

### Customize transitions¶

In state machines, transitions are composed of at least three items:

- `from` followed by the origin state
- `to` followed by the destination state
- a **quantifier** to indicate the flow from origin to destination
  state, which can be:
  - `rate` with a transition rate per individual i.e. a number per
    time unit. In stochastic models , rates are automatically
    converted into probabilities, assuming that durations in the
    origin state follow an exponential distribution
  - `proba` with the probabiliy that, during *one time unit*, an
    individual moves from origin to destination state
  - `amount` which indicates an absolute number of individuals,
    (bounded by the number of individuals actually in origin state)
  - `amount-all-but` which indicates an absolute number of
    individuals which have to *stay* in origin state while the others
    move to destination state. Hence, writing `amount-all-but: 0`
    means “all individuals in origin state”

Warning

In a state machine, all quantifiers may be used. However, from a
given origin state, all transitions that can be used simultaneously
must have the same quantifiers (i.e. only `rate`, or only
`proba`, or only `amount`/`amount-all-but`).

Transitions can also incorporate additional elements:

- `cond` followed with a expression (logical or numeric, non-zero
  values being considered true) specifies which individuals are
  allowed to cross the transition
- `escape` is a special condition for origin states endowed with a
  specific duration, which allows individuals that fulfil the
  expression to exit their state *before* the normal term. For
  instance, a gestating state has a duration before which individuals should
  not be able to leave the state, but if abortions are possible, the
  corresponding condition can be used as escape condition to permit a
  premature exit of gestation.
- `when` is a special condition which refers to *events* from a
  calendar (useful to handle seasonality)
- `on_cross` is used to specify a list of actions that indivuals
  moving from origin state to destination state through this
  transition have to perform

### Produce new individuals¶

- Section `productions` in state machines allow to define how some
  states can produce new individuals. Production links are very
  similar to transitions in their syntax, except that:

  - `escape` conditions and `amount-all-but` quantifier are not allowed
  - in IBM/hybrid models, a `prototype` must be specified for the new
    individuals

  Besides, the computation of actual flows uses sampling in Poisson
  distribution rather than binomial/multinomial sampling.

  See also

  SIR model with basic demography (births/deaths), SIR model with age groups, Design prototypes for typical individuals or populations
- Another way to produce new individuals is using an action: `clone`
  (aka `produce_offspring`) associated with a transition or
  with a state (see Set states attributes and Built-in actions).

  See also

  SIR model with explicit gestation

### State machines without transitions¶

State machines can also be used only for defining states, e.g. to
differentiate discrete values such as categories (male/female
individuals) or enumerations (e.g. female parities). In that case, the
state machine does not require any transition at all.

See also

SIR model with explicit gestation

## 5.3. Design prototypes for typical individuals or populations¶

In EMULSION hybrid and IBM models, prototypes are meant to represent
typical individuals (within populations) or populations (within
metapopulations), characterized by their state variables, either
regarding the state of a state machine (e.g. health state, age
group…), or specific features (e.g. age, temperature… of an
individual; initial health status, location, carrying capacity… of a
population).

Prototypes are defined in the section named `prototypes` (see
Model structure).

- Variables corresponding to regular values can be set using any valid expression:

  ```
  newborn:
    age: 0
  ```
- Variables corresponding to state machines can be set using:

  - the name of a state:

    ```
    healthy_individual:
      health_state: S
    ```
  - keyword `default` if a state was marked as default state in the
    state machine:

    ```
    healthy_individual:
      health_state: S
      age_group: default
    ```
  - keywords `next_state` or `previous_state` to select the
    “successor” or “predecessor” state of the current value (see
    Set state attributes on how to specify
    predecessor/successor relations between states - by default, they
    correspond to the order in which states are defined):

    ```
    next_parity:
      parity: next_state
    ```
  - keyword `random` possibly with parameters. Without parameters,
    one state is chosen randomly with equal probability among the
    states of the state machine (except for those marked as
    `autoremove`). To provide parameters:

    - if *N* (non-autoremove) states are defined in the state machine,
      either *N* or *N-1* values can be provided
    - values are interpreted in the same order as the definition of
      states in the state machine
    - if *N-1* values \(p\_i\) are given, they are interpreted as
      probabilities, the probability for the last state being:

      \[p\_N = 1 - \sum\_{i=1}^{N-1} p\_i\]
    - if *N* values \(v\_i\) are given, they are interpreted as
      weights and normalized to compute probabilities as follows:

      \[\forall i=1..N, p\_i = \frac{v\_i}{\sum\_{j=1}^N v\_j}\]

See also

SIR model with age groups, SIR model with basic demography (births/deaths),
SIR model with age groups and random initialization, SIR model with metapopulation,
SIR model with explicit gestation

## 5.4. Regulate time¶

- The simulation time step can be changed either in the model file
  (section `time_info`), or at runtime with option `-p
  delta_t=value`
- Section `time_info` allows the definition of calendars with
  events, for instance a pasture period:

  Example

  > ```
  > calendars:
  >   pasture:
  >     period: {days: 365}
  >        events:
  >          pasture_period: {begin: 'April 1', end: 'October 1'}
  >          open_days: {date: 'May 1'}
  > ```

  A calendar can be periodic or not, and define events that span from
  a `begin` date to and `end` date, or that take place at a
  specific `date`. Each event (here `pasture_period` and
  `open_days`) can be used in conditions, especially in the
  `when` clause of transition or productions. A transition with
  e.g. `Not(pasture_period)` will not be considered at all when the
  condition is not fulfilled.

  Besides, events with a begin and an end date automatically generate
  two other events, here for instance `begin_pasture_period` and
  `end_pasture_period`.

  See also

  model Quickstart provided to test
  EMULSION installation

## 5.5. Complexify grouping¶

Grouping can be based on several variables, especially when the
dynamics of one process is affected by another one. For instance, if
infection is driven by age groups, the grouping section should be
rewritten as follows:

Example

```
grouping:
  population:
    infection:
      state_machine: health_state
      key_variables: [health_state, age_group]
    age_group:
      state_machine: age_group
      key_variables: [age_group]
```

See also

SIR model with structured population

Grouping are especially useful in hybrid models, either to accelerate
simulation time, or to benefit from automatic variables.

A grouping can also be designed just to have access to specific
subgroups in the population, and thus indicate no state machine, nor
rely on variables related to state machines.

Warning

In any case, the name of the grouping must appear in the list of
processes: otherwise the grouping is not updated at each time step
(individuals are not distributed in the proper groups).

## 5.6. Aggregate variables¶

When defining levels, variables that aggregate other variables from a
sublevel can be defined, for instance:

Example

```
levels:
  population:
    ...
    aggregate_vars:
      - name: pop_affected_over_time
        collect: nb_episodes
        operator: 'sum'
      - name: 'avg_inf_duration'
        collect: duration_infected
        operator: 'mean'
```

Aggregated variables (here, `pop_affected_over_time` and
`avg_inf_duration`) defined by a level (here, `population`) consist in
collecting the values of other variables (`nb_episodes`,
`duration_infected`) in the sublevels (`individuals`) and applying
an operator (`sum`, `mean`) to compute the resulting value.

Operators can be any classical usual operation operating on lists:
sum, prod, min, max, mean, var, std, median, all, any. Shortcuts have
been defined so that e.g. `percentile20` computes the 20th
percentile.

See also

SIR model with individual actions and variable aggregation

## 5.7. Automatic variables¶

EMULSION automatically provides variables in relation to model
components.

- `step` represents the current time step, `delta_t` the duration
  of one time step (in time units), and `time` the time elapsed
  since the beginning of simulation (in time units)
- When defining a level, e.g. `population`: `total_population` gives the total
  population of this level.
- When defining a state machine, e.g. `health_state`, and its
  states, e.g. `S`:
  - `is_S` tests whether or not an individual is in state `S`
  - `duration_in_health_state` contains the duration elapsed since
    the individual entered the current state of state machine
    `health_state`
  - `total_S` is the number of individuals in state `S`
- When defining complex groupings, e.g. `[age_group, health_state]`:
  - `total_J_I` is the number of individuals in age group `J` and
    in health state `I`
  - if aggregated variables (e.g. `mean_age`) were also defined,
    their counterpart is automatically defined for the grouping
    (e.g. `mean_age_J_I` etc.)

See also

SIRS model with periodic external risk,
SIR model with structured population

## 5.8. Built-in functions¶

Expressions used in EMULSION models can refer to classical Python
mathematical operators (`+`, `-`, `*`, `/`, `**` for
exponentiation, etc. ) and functions (e.g. `sqrt`, `cos`, `sin`,
`exp`, `log`, `log10`, etc.).

The following functions are also available:

`AND(cond1, cond2)`
:   Logical conjunction: return true (1) if `cond1` and `cond2` are
    both true, false (0) otherwise

`OR(cond1, cond2)`
:   Logical disjunction: return true (1) if `cond1` is true or
    `cond2` is true, false (0) otherwise

`Not(cond)`
:   Logical negation: return true (1) if `cond1` and `cond2` are
    both true, false (0) otherwise

`IfThenElse(condition, val_if_true, val_if_false)`
:   Ternary conditional function: return either `val_if_true` or
    `val_if_false` depending on `condition`.

`random_bool(proba_success)`
:   Return a random boolean value (0 or 1) depending on
    `proba_success`

Shortcuts to distributions from package `numpy.random` are also
available:

```
random_uniform(a, b)
random_integers(a, b)
random_exponential(m)
random_poisson(m)
random_beta(a, b)
random_normal(m, sd)
random_gamma(a, b)
```

See also

Custom state durations,
SIRS model with periodic external risk

## 5.9. Built-in actions¶

`{action: action_name, d_params: dict_of_parameters}`
:   The generic keyword to call an external actions, requires Python code.

    - `action_name` has to be described (`desc: ...`) explicitly in
      main section `actions`
    - `action_name` must be provided in a separate Python file, as a
      *method* in a class associated with the level at which the action
      will be performed. This method will look as (assuming that
      `d_params` contains two keys, `param1` and `param2`):

      Example

      ```
      def action_name(self, param1, param2):
          """Do something with *param1* and *param2* as specified in
          section ``actions``"""
          # here the Python code for the action
      ```

    Whenever possible, prefer other built-in actions described below.

`{message: 'Some important information'}`
:   Print the specified character string on the standard output while
    running the model, preceded by the simulation ID, the time step
    and the identifier of the individual.

    Example

    ```
    states:
    ...
      - I:
        ...
        on_enter:
          - message: 'now infected !'
    ```

    will produce outputs like this:

    ```
    $0,@2,AtomAgent #55,now infected !
    $0,@5,AtomAgent #49,now infected !
    ```

`{log_vars: list_of_variables_or_expressions}`

> Store the specified list of variable to a log file named
> `log.txt` in the output directory (by default, `outputs/`).
> Each line starts with the simulation ID, the time step, the class and
> identifier of the individual, then each variable is associated
> with its value.
>
> Example
>
> ```
> states:
> ...
>   - I:
>     ...
>     on_enter:
>       - log_vars: [is_S, is_I, is_R]
> ```
>
> will produce a log file `outputs/log.txt` containing something like this:
>
> ```
> 0,0,AtomAgent,100,is_S=False,is_I=True,is_R=False
> 0,4,AtomAgent,57,is_S=False,is_I=True,is_R=False
> 0,5,AtomAgent,8,is_S=False,is_I=True,is_R=False
> ```

`{set_var: varname, value: expression}`
:   Change the value of `varname` according to what is calculated in
    `expression`.

    See also

    SIR model with individual actions and variable aggregation

`{record_change: varname}`
:   Add the number of individuals performing the action to the
    specified state variable, assumed to be defined at population
    level. This can be used for instance to calculate the cumulative
    incidence (e.g. as an action `on_enter` for the infectious
    state).

    See also

    SIR model with cumulative incidence

`{clone: a_prototype_or_list, amount: value, proba: value_or_list}`
:   Clone the individual into new ones. The newly produced individuals
    follow the specified prototypes, according the given amount and
    probabilities.

    - default amount is 1
    - if no probabilities are given, prototypes are considered
      equiprobable
    - a single prototype can be given as a single value instead of a
      list
    - the list or probabilites must be of same size of prototypes
      list, only the last value may be omitted to ensure that the sum
      of probabilities is 1. When N prototypes are associated with N
      probability values which sum to S < 1, then there is a
      probability 1-S to produce no offspring. Once probabilies are
      actualized, a multinomial sampling is performed to determine the
      number of new individuals in each category

    Example

    A model with explicit gestation states and possibly vertical disease
    transmission:

    ```
    - from: G
      to: NG
      proba: 1
      on_cross:
        - clone: [infected, healthy]
          amount: 1
          proba: [proba_vertical_transmission]
          # prototype 'healthy' has proba: 1-proba_vertical_transmission
    ```

    See also

    SIR model with explicit gestation, Design prototypes for typical individuals or populations

`{produce_offspring: a_prototype_or_list, amount: value, proba: value_or_list}`
:   Synonym of `clone`.

`{become: a_prototype_or_list, proba: value_or_list}`
:   Force the individual to adopt the specified prototype(s), possibly
    with probabilities (works like `clone` above). Useful to couple states
    from distinct state machines.

## 5.10. Changing scale: metapopulations¶

In EMULSION, `metapopulation` is an aggregation type which allows to
handle multiple populations without regard to how this population is
built (compartment-based/hybrid/IBM).

It consists in defining a new level and completing initial conditions
through new population prototypes.

It may be also necessary to transform within-population parameters
into variables to allow for heterogeneous populations.

See also

SIR model with metapopulation

## 5.11. Connecting to Python code add-ons¶

EMULSION is aimed at providing *generic* features required for
designing epidemiological models. Thus, some very specific operations
are not available in the generic engine. In such cases, a small code
add-on is required to provide additional functionalities.

Code add-ons are mainly used for specific requirements regarding:

- variables which are complicated to change through expressions and
  actions
- actions which are not realizable through built-in ones
- processes which are not realizable through state machines
- complex intial conditions
- data loading

To link a level with a code add-on, two elements are required:

- `file: my_code_add_on.py` which specified the Python source code to use
- `class_name: AClassName` which defines the name of the Python
  class associated with the level

EMULSION provides an (experimental) code generator (`emulsion
generate MODEL.yaml`) which builds a code skeletton for files and
classes mentioned in the level definition. All actions and variables
listed respectively in `actions` and `statevars` sections, as well
as processes not associated with state machines or groupings, are
assumed to be defined in the Python file. All you need then is to fill
the relevant methods with the corresponding code, or remove
unnecessary parts.

See also

SIR model with metapopulation and data-driven movements

# EMULSION

Epidemiological Multi-Level Simulation Framework

### Navigation

- 1. Installation
- 2. Getting started with EMULSION
- 3. Modelling principles
- 4. Modelling language (basics)
- 5. Modelling language (advanced)
  - 5.1. Compartments, IBM or hybrid models?
  - 5.2. Master state machines
    - Set states attributes
    - Customize transitions
    - Produce new individuals
    - State machines without transitions
  - 5.3. Design prototypes for typical individuals or populations
  - 5.4. Regulate time
  - 5.5. Complexify grouping
  - 5.6. Aggregate variables
  - 5.7. Automatic variables
  - 5.8. Built-in functions
  - 5.9. Built-in actions
  - 5.10. Changing scale: metapopulations
  - 5.11. Connecting to Python code add-ons
- 6. Feature examples
- 7. Information
- 8. License
- 9. High-level functions for model designers
- 10. emulsion package

### Related Topics

- Documentation overview
  - Previous: 4. Modelling language (basics)
  - Next: 6. Feature examples

### Quick search

©2016, INRA and Univ. Lille.
|
Powered by Sphinx 1.8.5
& Alabaster 0.7.10
|
Page source
