# Supplementary material for: EMULSION: Transparent and flexible multiscale stochastic models in human, animal and plant epidemiology
Source: PLoS Comput Biol. 2019 Sep 13;15(9):e1007342. doi: 10.1371/journal.pcbi.1007342 (PMC6760811; doi:10.1371/journal.pcbi.1007342)
Supplement: S2 File — This zip file is a clone of EMULSION public Git repository. To install from this file rather than from PyPI, go to EMULSION documentation page: https://sourcesup.renater.fr/www/emulsion-public/pages/Install.html and follow Git-based installation instructions. (ZIP) [file pcbi.1007342.s010.zip › S2_file/doc/pages/Information.html]

7. Information — EMULSION (Epidemiological Multi-Level Simulation framework)

# 7. Information¶

## 7.1. Contributors and contact¶

- Sébastien Picault (sebastien.picault@inra.fr)
- Yu-Lin Huang
- Vianney Sicard
- Gaël Beaunée
- Sandie Arnoux
- Pauline Ezanno (pauline.ezanno@inra.fr)

BIOEPAR, INRA, Oniris, Atlanpôle La Chantrerie, route de Gachet CS
40706, 44307 Nantes, France

## 7.2. How to cite¶

S. Picault, Y.-L. Huang, V. Sicard, P. Ezanno (2017). “Enhancing
Sustainability of Complex Epidemiological Models through a Generic
Multilevel Agent-based Approach”
in: C. Sierra (ed.), *26th International Joint Conference on
Artificial Intelligence (IJCAI)*, AAAI, p. 374-380.

DOI: 10.24963/ijcai.2017/53

```
@InProceedings{ijcai2017-emulsion,
  author =	 {Picault, Sébastien and Huang, Yu-Lin and Sicard,
                  Vianney and Ezanno, Pauline},
  title =	 {Enhancing Sustainability of Complex Epidemiological
                  Models through a Generic Multilevel Agent-based
                  Approach},
  booktitle =	 {Proceedings of the 26th International Joint
                  Conference on Artificial Intelligence (IJCAI'2017)},
  year =	 {2017},
  editor =	 {Sierra, Carles},
  publisher =	 {AAAI},
  pages =	 {374--380},
  DOI =		 {10.24963/ijcai.2017/53},
  ISBN =	 {978-0-9992411-0-3},
}
```

## 7.3. Selected publications¶

- On bovine respiratory disease (BRD) in feedlots:

  S. Picault, P. Ezanno, S. Assié (2019). “Combining early
  hyperthermia detection with metaphylaxis for reducing antibiotics
  usage in newly received beef bulls at fattening operations: a
  simulation-based approach”, in: *SVEPM Annual Conference and General
  Meeting* (in press)
- On Q fever (Coxiellosis) in dairy herds:

  - S. Picault, Y.-L. Huang, V. Sicard, F. Beaudeau, P. Ezanno
    (2017). “A Multi-Level Multi-Agent Simulation Framework in Animal
    Epidemiology”, in: Demazeau *et al.* (eds.), *15th International
    Conference on Practical Applications of Agents and Multi-Agent
    Systems (PAAMS)*, Lecture Notes in Computer Science
    10349, p. 209-221, Springer.

    DOI: 10.1007/978-3-319-59930-4\_17
- On Artificial Intelligence research involved in EMULSION:

  - P. Mathieu, G. Morvan, S. Picault (2018). “Multi-level agent-based
    simulations: Four design patterns”, *Simulation Modelling Practice
    and Theory* 83, p. 51-64, Agent-Based Modelling and Simulation
    special issue Elsevier.

    DOI: 10.1016/j.simpat.2017.12.015
  - S. Picault, P. Mathieu (2011). “An Interaction-Oriented Model for
    Multi-Scale Simulation”, in: T. Walsh (ed.), *22nd International
    Joint Conference on Artificial Intelligence (IJCAI)*,
    AAAI, p. 332-337.

    DOI: 10.5591/978-1-57735-516-8/IJCAI11-065

## 7.4. Acknowledgements¶

This work was carried out with the financial support of:

> - the French Research Agency (ANR), Program Investments for the
>   Future, project ANR-10-BINF-07 (MIHMES)
> - the European Union through the European fund for the regional
>   development (FEDER) of Pays-de-la-Loire
> - the French Research Agency (ANR), project ANR-16-CE32-0007-01
>   (CADENCE)
> - INRA, the French National Institute for Agricultural Research
>   (Animal Health Division)
> - the SANT’Innov project of the For and On Regional Development
>   (PSDR) French research programme, which is funded by INRA, the
>   National Research Institute of Science and Technology for
>   Environment and Agriculture (IRSTEA), and the French regions of
>   Bretagne, Pays de la Loire, Normandie and Nouvelle Aquitaine.

# EMULSION

Epidemiological Multi-Level Simulation Framework

### Navigation

- 1. Installation
- 2. Getting started with EMULSION
- 3. Modelling principles
- 4. Modelling language (basics)
- 5. Modelling language (advanced)
- 6. Feature examples
- 7. Information
  - 7.1. Contributors and contact
  - 7.2. How to cite
  - 7.3. Selected publications
  - 7.4. Acknowledgements
- 8. License
- 9. High-level functions for model designers
- 10. emulsion package

### Related Topics

- Documentation overview
  - Previous: 6. Feature examples
  - Next: 8. License

### Quick search

©2016, INRA and Univ. Lille.
|
Powered by Sphinx 1.8.5
& Alabaster 0.7.10
|
Page source
